# Environmentally driven extinction and opportunistic origination explain fern diversification patterns

**DOI:** 10.1038/s41598-017-05263-7

**Published:** 2017-07-06

**Authors:** Samuli Lehtonen, Daniele Silvestro, Dirk Nikolaus Karger, Christopher Scotese, Hanna Tuomisto, Michael Kessler, Carlos Peña, Niklas Wahlberg, Alexandre Antonelli

**Affiliations:** 1 Herbarium, Biodiversity Unit, University of Turku, 20014 Turku, Finland; 2 Department of Biology, University of Turku, 20014 Turku, Finland; 3 Department of Biological and Environmental Sciences, University of Gothenburg, Carl Skottsbergs gata 22B, Gothenburg, 413 19 Sweden; 4 Gothenburg Global Biodiversity Centre, Box 461, SE-405 30, Gothenburg, Sweden; 5 Department of Ecology and Evolution, University of Lausanne, 1015 Lausanne, Switzerland; 6 Swiss Institute of Bioinformatics, Quartier Sorge, 1015 Lausanne, Switzerland; 7 Department of Systematic and Evolutionary Botany, University of Zurich, 8008 Zurich, Switzerland; 8 Earth and Planetary Sciences, Northwestern University, Evanston, IL USA; 9 Department of Biology, Lund University, Lund, Sweden; 10 Gothenburg Botanical Garden, Carl Skottsbergs gata 22 A, Gothenburg, 413 19 Sweden

## Abstract

Combining palaeontological and neontological data offers a unique opportunity to investigate the relative roles of biotic and abiotic controls of species diversification, and the importance of origination versus extinction in driving evolutionary dynamics. Ferns comprise a major terrestrial plant radiation with an extensive evolutionary history providing a wealth of modern and fossil data for modelling environmental drivers of diversification. Here we develop a novel Bayesian model to simultaneously estimate correlations between diversification dynamics and multiple environmental trajectories. We estimate the impact of different factors on fern diversification over the past 400 million years by analysing a comprehensive dataset of fossil occurrences and complement these findings by analysing a large molecular phylogeny. We show that origination and extinction rates are governed by fundamentally different processes: originations depend on within-group diversity but are largely unaffected by environmental changes, whereas extinctions are strongly affected by external factors such as climate and geology. Our results indicate that the prime driver of fern diversity dynamics is environmentally driven extinction, with origination being an opportunistic response to diminishing ecospace occupancy.

## Introduction

The world’s biodiversity is the result of a complex interplay between biotic and abiotic drivers and their changes over time and space^1, 2^. Recent advances in paleontology and molecular phylogenetics have led to a renaissance in macroevolutionary research, but inherent biases in the fossil record and phylogenetic data often compromise inferences based on a single type of data^3^. Hence, the relative roles of different factors affecting species diversification, as well as the importance of origination versus extinction, remain contested^1–3^. Ferns are an unusually well-suited group to investigate these questions because of their high diversity throughout most of the history of terrestrial life^4, 5^, their rich fossil record^4–6^, and the well understood phylogenetic relationships among their extant members^7–11^.

Earlier, ferns were seen as a relict group with their heyday in the Palaeozoic and diminished importance towards the present, as they were gradually replaced by gymnosperms and angiosperms^4^. This view was challenged by molecular phylogenies revealing that the most diverse, predominantly epiphytic fern lineages diversified simultaneously with the angiosperms^12^. This has been taken as evidence for adaptive radiation of epiphytic ferns in angiosperm-dominated forests^12, 13^. Although these alternative views suggest contrasting interactions between ferns and angiosperms, they emphasize the importance of biotic factors in driving diversification as predicted by the “Red Queen” model of evolution^1, 14^. Other studies have linked fern diversification with the physical environment, by suggesting that warm and wet climates induced the simultaneous radiation of angiosperms and ferns^15^, that initial fern diversification was associated with volcanism^16^, or that increased drought allowed seed plants to replace ferns towards the end of the Palaeozoic^16^. These hypotheses are more congruent with the “Court Jester” model of evolution, in which changes in the physical environment are considered to be the main drivers of species diversification^1, 17^. The multiple and complex variables underlying these evolutionary models remain to be disentangled, especially while taking into account both fossil and molecular data in a statistically robust framework.

**Figure 1:**
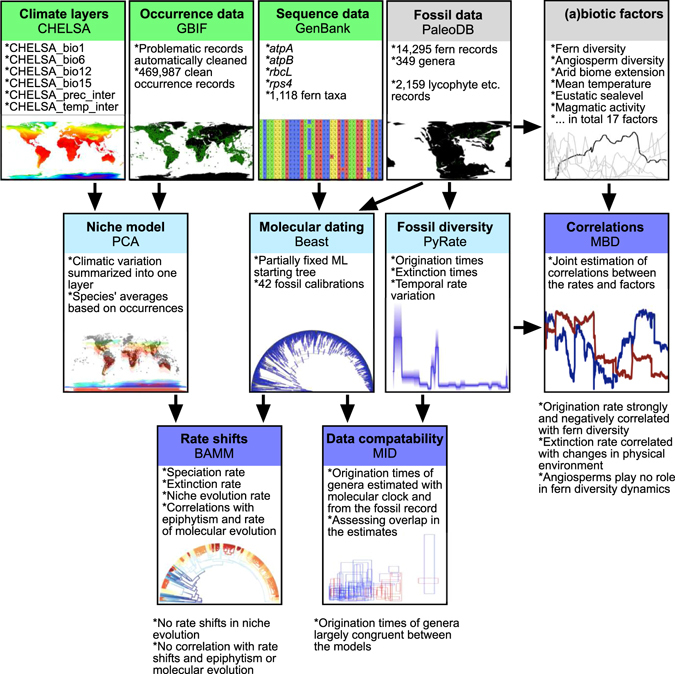
Analytical workflow developed for this study. We used neontological (green) and palaeontological (grey) data sources to model fern diversification dynamics. In the first round of analyses (pale blue) we summarized climatic layer information for each species, built a time-calibrated molecular phylogeny, and estimated origination and extinction times of fern genera using the fossil record. These analyses provided data for the final analyses (blue) where we tested whether the rate variation observed in the phylogeny and fossil record were correlated with various candidate biotic and abiotic factors, niche and molecular evolution, and life form. We also estimated data compatability between the neontological and palaeontological models. Maps of modern world were created using QGIS v. 2.0.1 (http://www.qgis.org) and the statistical programming language R^84^, the paleomap is based on PALEOMAP Global Plate Tectonic Model^85^ and was created using Adobe Photoshop CC 2015, PointTracker^86^ and QGIS v. 2.0.1 (http://www.qgis.org).

Here we develop a Bayesian Multivariate Birth-Death (MBD) model to simultaneously estimate the impact of multiple environmental and biotic factors on the genus-level origination and extinction rates of ferns. We model the temporal variation in fern diversity for a fossil dataset comprising 14,295 fern occurrences (Supplementary Figure 1, Supplementary Data 1) encompassing 349 extinct and extant genera. We test whether rate variation could be the result of the interaction of ferns with various biotic and abiotic factors, including several climatic, environmental and geologic factors as well as diversities of major plant groups (Supplementary Figure 2). We complement these inferences with the analysis of a large molecular dataset of modern taxa, by modelling fern diversification dynamics on the basis of a large time-calibrated molecular phylogeny and correlating the inferred diversification rates with molecular and ecological evolution. Our combined, multi-variate approach allows us to test the drivers of origination and extinction throughout the history of the terrestrial flora (Fig. 1).

## Results

### Successive replacements of fern clades

**Figure 2:**
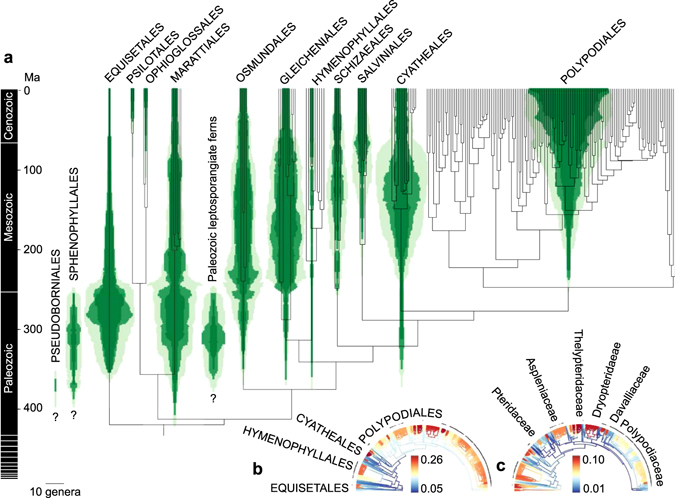
Diversification dynamics of ferns through time. (**a**) The green diagrams show the diversity dynamics of fern genera that were assigned to higher taxa (orders) inferred from the fossil record (Supplementary Table 1), plotted above a time-calibrated molecular phylogeny collapsed to the genus level. Pale green indicates the estimated maximum diversity (95% HPD), dark green the estimated minimum (95% HPD), and intermediate green the estimated mean diversity. (**b**) Diversification rate shifts identified from a species-level phylogeny for speciation and (**c**) extinction rates. Some important higher taxa (orders in **b** and families in **c**) are labelled and epiphytic species indicated by dots.

**Figure 3:**
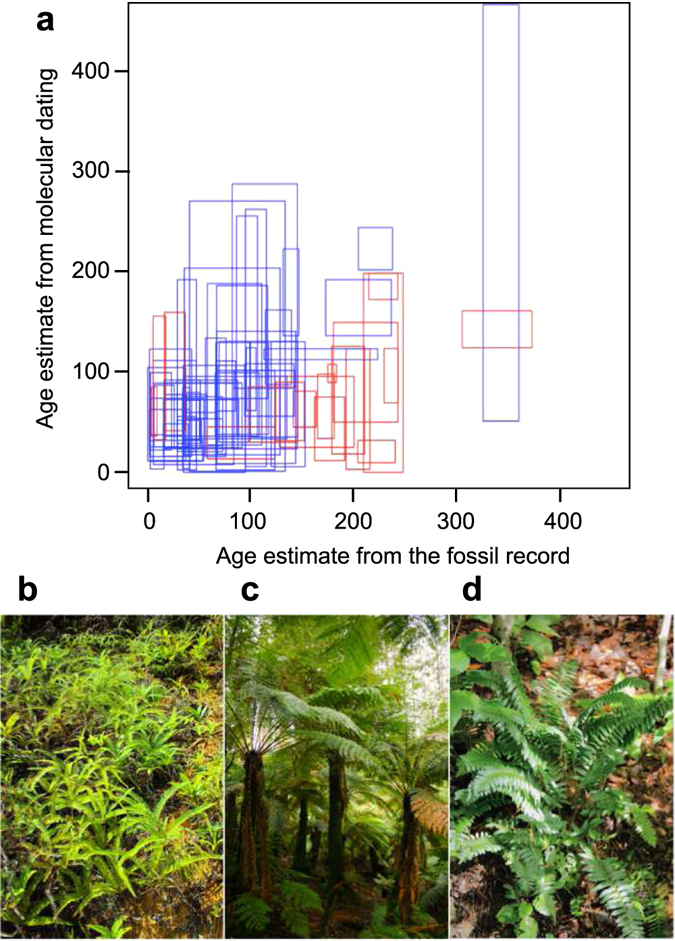
Characterisation of the studied fern genera. (**a**), Estimated ages of fern genera present in both molecular and fossil data sets (*n* = 76). Molecular age interval represents 95% HPD combining the stem and crown group nodes. Genera with congruent age estimates between the two methods are shown in blue and non-overlapping estimates in red. (**b**) Representative taxa from the analyses that include both living and extinct relatives: Gleicheniales: *Dicranopteris flexuosa*. (**c**) Cyatheales: *Dicksonia antarctica*. (**d**) Polypodiales: *Polystichum acrostichoides*.

**Figure 4:**
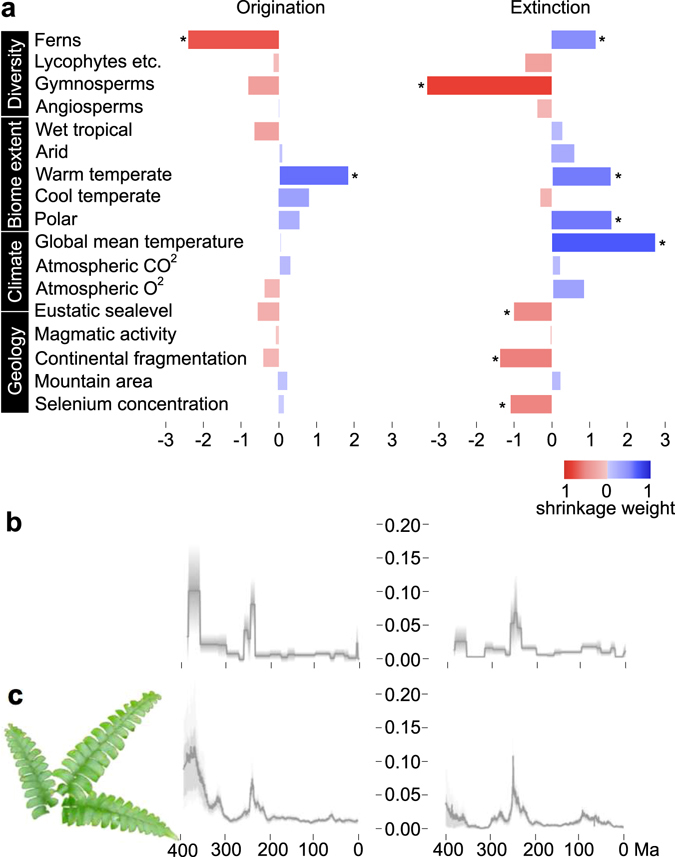
Paleoenvironmental correlates and diversification dynamics of ferns. (**a**) Correlation parameters (G_*i*_) for fern originations and extinctions with darker colours indicating higher shrinkage weight (red for negative, blue for positive correlation). Significant correlations (shrinkage weight >0.5) are indicated by asterisks. (**b**) Origination (left panel) and extinction (right panel) rates through time as estimated from the fossil record using the BDS model and (**c**) by the MBD model. The clear similarity between origination and extinction dynamics estimated under the two models indicate that the MBD model and the set of variables tested here can adequately recover the rates inferred under the BDS model disregarding potential correlates.

Our time-calibrated phylogeny is generally congruent with the current understanding of fern relationships^7–11, 18^. We resolved Equisetales as sister to all other ferns, Marattiales as sister to the Ophioglossales-Psilotales clade, and Hymenophyllales as sister to Gleicheniales (Fig. 2, Supplementary Data 2). These deep lineages have remained controversially resolved and poorly supported; an emerging consensus agrees with our position of Equisetales but not with that of Marattiales^9, 10, 18^. We estimated the crown age for ferns as 421.30 Ma (95% CI: 466.97–379.16) based on molecular dating and this was congruent with the 411.06 Ma (95% CI: 424.45–392.88) directly modelled from the fossil record. These Silurian-Devonian ages are comparable with some recent molecular dates^7, 9, 15, 19^ and fossil evidence^20^, although older than has often been assumed^12, 21, 22^. The estimated ages of fern genera present both in the palaeontological and neontological datasets are largely congruent between the fossil and molecular models (with credible intervals overlapping in 72% of cases, *n* = 76; Fig. 3, Supplementary Tables 1–3). When age estimates were incongruent, the fossil-based estimates were more often older (*n* = 18, 55.97 Ma) than younger (*n* = 3, 15.46 Ma) in comparison to molecular estimates. This may reflect taxonomic errors and a tendency to lump unrelated fossils into broad living genera (e.g. *Asplenium* and *Cyathea*). Both models also support high extinction rates for all major fern lineages except the Polypodiales (Fig. 2). Our model captures the previously demonstrated fern mass extinction at the Permo-Triassic (P-T) boundary at 251 Ma^6^, but reveals that this led to accelerated origination rate and massive taxonomic turnover at generic level rather than an overall diversity collapse (Fig. 4b, Supplementary Figure 3).

### Originations and extinctions are controlled differently

Our MBD analysis indicates that origination is a largely diversity-dependent process (Fig. 4a), wherein the origination rate slows down as ferns’ own diversity increases (shrinkage weight *w* = 0.76, correlation parameter G_*i*_ = −2.41; where *w* > 0.5 indicates significant correlation). In contrast, extinctions are strongly correlated with external environmental factors (Fig. 4a), which thus stand out as the prime drivers of diversity dynamics. The strongest interaction in our model is a negative correlation between the fern extinction rate and gymnosperm diversity (*w* = 0.84, G_*i*_ = −3.31). The second most important factor is global mean temperature with a strong positive correlation with fern extinction rate (*w* = 0.78, G_*i*_ = 2.74). Other factors that are positively and significantly correlated with fern extinctions include fern diversity (*w* = 0.52, G_*i*_ = 1.17) and the extent of warm temperate (*w* = 0.61, G_*i*_ = 1.54) and polar biomes (*w* = 0.63, G_*i*_ = 1.59), while significant negative correlations include continental fragmentation (*w* = 0.58, G_*i*_ = −1.37), selenium concentration in marine sediments (*w* = 0.54, G_*i*_ = −1.09), and eustatic sea level (*w* = 0.51, G_*i*_ = −1.00; Fig. 4a).

To test whether the expanding and diminishing fern clades show different response to these factors we repeated the MBD model for Polypodiales and non-Polypodiales, and included the diversities of non-Polypodiales and Polypodiales, respectively, as an additional factor into the models. We did not find significant correlations between the origination or extinction rates of the Polypodiales clade with any of the explanatory variables. For the non-Polypodiales we observed generally similar patterns as for the full data, with the origination rate mainly explained by their diversity (diversity dependence *w* = 0.72, G_*i*_ = −2.73; warm temperate biome *w* = 0.71, G_*i*_ = 2.09; gymnosperm diversity *w* = 0.53, G_*i*_ = −1.18; eustatic sealevel *w* = 0.50, G_*i*_ = −0.72) and the extinction rate explained by external factors (gymnosperm diversity *w* = 0.85, G_*i*_ = −3.43, global mean temperature *w* = 0.79, G_*i*_ = 2.80, warm temperate biome *w* = 0.58, G_*i*_ = 1.45, cool temperate biome *w* = 0.58, G_*i*_ = 1.30, diversity dependence *w* = 0.54, G_*i*_ = 1.30, selenium concentration in marine sediments *w* = 0.53, G_*i*_ = −1.06, continental fragmentation *w* = 51, G_*i*_ = −1.17).

### Diversification is decoupled from niche shifts

Some of the early diverging fern lineages are predominantly tropical^6^ while others show apparent phenotypic and genomic stasis^6, 23^. This could reflect evolutionary stagnation of these once prolific clades, possibly explaining their low modern diversity. We tested this hypothesis by modelling the rate of niche evolution through the fern phylogeny as a continuous phenotypic trait but found no support for rate changes (93% of the samples in the posterior distribution supported zero rate shifts; Supplementary Figure 4), nor correlations between speciation-extinction dynamics and either rate of molecular evolution (Mann–Whitney *U*-test: speciation *P* = 0.032, extinction *P* = 0.243) or epiphytism (speciation *P* = 0.812, extinction *P* = 0.687) in structured permutation tests (STRAPP)^24^.

## Discussion

The inferred diversity dynamics reveal waxing and waning of fern orders, with a peak of diversity in most orders followed by a declining phase (Fig. 2a). These successive clade replacements reflect complementary aspects of massive taxonomic turnover through time and provide a more nuanced evolutionary scenario than the alternative views that ferns are either an entirely relict group^4^, or a group with mostly recent active evolution^12^. Our results provide compelling evidence that this turnover is the result of fundamentally differently controlled originations and extinctions^2^. Most diversification appears decoupled from major niche shifts, as indicated by a uniform niche shift rate and lack of significant correlation between epiphytism and diversification. Although epiphytism has been generally considered as a key adaptation allowing rapid fern radiations^12, 13^, other recent studies have also failed to find support for this hypothesis and requested alternative explanations^7, 25^. All these studies have estimated diversification-extinction dynamics from molecular phylogenies and may therefore be compromised, because of the inherent difficulties in estimating extinction rates from the living diversity only^26^. We complemented our phylogeny-based inferences with analyses of fossil record to achieve a more robust estimate of extinction rate. Due to the nature of the plant fossil record these analyses had to be run at genus-level, but this is generally considered as acceptable proxy for species diversity^27^.

The limited role of adaptive pressure in diversification was also supported in our analyses of the fossil record, in which the intrinsic diversity dependence was detected as the primary driver of origination rate. Our MBD model restricted to the currently expansive Polypodiales clade revealed that their radiation is consistent with a roughly constant birth-death model without significant correlations with the available environmental variables. This supports the view that lack of environmental effect on fern origination indicates environmentally neutral diversification rate rather than failure to detect the possibly unique and contrasting environmental drivers of ecologically and phylogenetically distinct fern lineages. It should be noted, however, that our MBD model correlates genus level origination rate with coarse environmental variables at global level and the applicability of these conclusions on the adaptive processes that take place at the species level remains to be tested.

A neutral, clock-like speciation model has been proposed to rule across the tree of life^28^, but neither the rapid diversity rebound after the P-T mass extinction event, nor the general diversity-dependent slowdown in diversification rate fit the expectations of that model. We also find no evidence for the neutral biodiversity theory^29^ as an explanation of the observed pattern, because that model predicts increasing taxonomic turnover with increasing diversity, which we do not observe (Spearman correlation, *r* = −0.46, *P* = 1; Supplementary Figure 3b). The species selection^30^ hypothesis might explain ecologically neutral radiations and their later collapse to marginality^30, 31^. Under this scenario, speciation and extinction rates are affected by lineage-specific traits that determine the likelihood of speciation or ability to resist extinction, while environmental changes alter the adaptive landscape for entire clades^30^. Alternatively, a neutral pattern may emerge from stochastic subdivision of available ecospace^1, 32, 33^. This model resembles the Simpsonian ecospace model^3, 34^, but with originations seen as passive, stochastic responses to increased ecospace availability, rather than actively driven by adaptation^32^. Lower diversity may allow species to occupy larger geographic areas^35^ and wider parts of ecological gradients^36^, both of which may increase the probability of lineage splitting by chance^32, 37^. Under the neutral origination model, the rate at which ecospace becomes subdivided will decrease as it becomes more finely divided, thus resulting in diversity dependence^32^. This model further predicts that closely related species occupy relatively similar niches, due to either non-adaptive allopatric speciation or subdivision of the ancestral niche, resulting in a pattern resembling widely observed phylogenetic niche conservatism^38^, which has been documented also among ferns^39^. Complex host-pathogen interactions could result in diversification patterns that incorrectly suggests ecological neutrality^40^ but neither methods nor sufficient data are currently available for modelling such interactions through evolutionary history.

In contrast to diversity-dependent origination rates, our results suggest that extinction rates are primarily driven by perturbations in the physical environment, thus supporting the Court Jester model^1, 14^. We interpret the strong interaction in our model indicating an increase in fern extinction rate with decreasing gymnosperm diversity as the result of an external forcing that affects both groups simultaneously. This external factor may have been connected to temperature, as we found that global mean temperature was strongly and positively correlated with fern extinction rate. High temperatures have been linked with extinction dynamics in the marine realm^2^ and uncontrolled water loss in ferns^41^. The observed association between increasing extinction rate and expansion of extra-tropical biomes is consistent with tropically centred modern fern distributions. However, we failed to observe direct correlation between the extension of wet tropical biome with fern extinctions, but this may be because of the strong sampling bias against tropical regions in the Mesozoic-Cenozoic fossil data (Supplementary Figure 1). The increased extinction rate in less fragmented continental settings may reflect effects not captured by the other factors in our model, such as more pronounced monsoon climate^42^, or reduced opportunities to support endemic floras, two poorly quantifiable variables. Modern fern diversity is strongly related to soil fertility with long-term evolutionary implications^39^. We used selenium as a proxy for overall substrate fertility and observed increased fern extinctions in relation to decrease in fertility. Severe selenium depletions have been associated with marine extinction events^43^ and our results suggest that fertility may have played a role in controlling diversity also in the terrestrial ecosystems.

Although competition with angiosperms is an often cited explanation for the decrease in the diversity of ferns towards modern times^4, 12, 16^, angiosperm diversity remained a negligible factor in our model. This result also hold for the models considering Polypodiales and non-Polypodiales separately. Thus, we failed to find support for the hypothesis that the rise of angiosperms drove taxonomic replacement of other fern orders with Polypodiales^12, 13, 15, 44^. The hypothesis that angiosperms triggered adaptive radiation in epiphytic ferns has been recently questioned and the need for alternative explanations acknowledged^7, 25^. Our approach combining palaeontological data and molecular phylogenies fulfils this need and provides a novel view on the possible drivers of this major radiation.

Taken together, our results indicate that fern diversity dynamics are primarily driven by environmentally induced extinctions, with origination being an opportunistic response to diminishing ecospace occupancy. We hypothesize that these conclusions also hold for many other taxonomic lineages.

## Methods

### Modelling temporal variation in fossil diversity

The phylogenetic relationships of the early ferns and fern-like plants are poorly understood, making taxonomical assignment of the fossil species difficult^45^. We excluded Lycopsida, Trimerophytopsida, Rhacophytales, Stauropteridales, Cladoxylopsida and Zygopteridales from ferns, but included Sphenophytes (Pseudoborniales, Sphenophyllales and Equisetales)^45^.

Fossil occurrence data were compiled at genus level to avoid taxonomic problems and scarcity of data at species level^46^. We excluded genera described solely on spores, but included records of fossil spores assigned to extant genera. The fossil dataset is largely based on previous compilation^46^ with additional records queried from Paleobiology Database (PBDB; https://paleobiodb.org/#/), Global Biodiversity Information Facility (GBIF; http://www.gbif.org), the paleobiology database of the Swedish Museum of Natural History (http://www.nrm.se/english/researchandcollections/palaeobiology/collections.851_en.html), and the literature (Supplementary Data 3). The stratigraphic ages reported for the fossils were translated into a numeric timescale^47^. The final dataset is composed of 14,295 fossil fern occurrences representing 349 genera, of which 308 were assigned to higher taxa (order) and 76 are still extant (Supplementary Data 1). The age of fossil occurrences ranged from 405.66 Ma (+/−7.59 Ma) to 0.03 Ma (+/−0.02 Ma) and most of the occurrences (99.8%) were provided with a minimum and maximum age, reflecting the estimated boundaries of stratigraphic units. We treated these time ranges (of average length = 16.24 Ma) as dating uncertainties^48^ and randomly resampled the ages of each fossil occurrence from uniform distribution spanning the time ranges, generating 100 datasets. Thus, all the fossil analyses were replicated over 100 randomized datasets to incorporate dating uncertainties in our parameter estimates.

We jointly modelled fossil preservation and the dynamics of origination and extinction within a hierarchical Bayesian framework using PyRate^49^. As in previous analyses^46^, we used a uniform Poisson process of preservation to estimate the expected number of fossil occurrences per lineage/Ma and allowed for rate heterogeneity across lineages using the Gamma model with eight rate categories. We inferred temporal dynamics of diversification by setting a birth-death model with rate shifts (BDS) defined by the epochs of the stratigraphic geological timescale^47^ and estimated origination and extinction rates within these time intervals. To control for overparameterization, we assumed a single half Cauchy prior distribution for all origination rates (*C* ^*+*^[0, *s*_*1*_]) and one for all extinction rates (*C* ^*+*^[0, *s*_*2*_]), where the scale parameters *s*_*1*_ and *s*_*2*_ were themselves assigned a uniform hyper-prior (*U*[0, 20]) and estimated from the data^46^.

In summary, the main parameters estimated under the BDS model were (a) preservation rate and its degree of heterogeneity, (b) times of origination and extinction for all genera in the dataset, (c) origination and extinction rates through time. We approximated the posterior distributions of all parameters through Markov Chain Monte Carlo (MCMC) and ran 25,000,000 iterations (sampling every 10,000) to achieve convergence. We combined posterior samples from 100 randomized datasets and summarized the parameters by calculating their mean and 95% credible intervals (95% CI). We assessed convergence and burnin fraction by inspecting the log files in Tracer^50^ and considered effective sample sizes exceeding 100 as a sufficient sample from the posterior distribution.

We found a high degree of heterogeneity in preservation rates across lineages, as indicated by a small value of the estimated shape parameter of the gamma distribution describing rate heterogeneity, alpha = 0.33 (95% CI: 0.22–0.36)^48^. The median preservation rate across lineages was 0.73 (95% CI: 0.29–0.81). Estimated origination and extinction times of genera are given in Supplementary Table 1. Based on the results of the PyRate analyses run under the BDS model, we plotted the marginal rates of origination and extinction through time (Fig. 4b), and summarized the estimated times of origination and extinction using the –SE_stats command in PyRate (Supplementary Figure 3).

### Phylogenetic inference and molecular dating

We updated a dataset underlying a large-scale fern phylogeny^8^ by querying GenBank release 184 (June 15 2011) with PhyLoTA^51^ browser and supplementing additional sequence data^39^ not included in the queried release. We concatenated the aligned sequence data representing four plastid genes (*atpA*, *atpB*, *rbcL*, *rps4*) and excluded all taxa without the *rbcL* gene and at least one other marker, or with less than 1,000 base pairs of sequence data. In order to allow time calibration within reasonable time the most similar taxa (defined by the uncorrected pairwise changes of aligned sequences) were removed until no pair of taxa had pairwise distance less than 0.5%. During the pairwise comparison the taxon with more sequence data was retained, or selection was randomly done if both pairs had equal amount of data. This resulted in a dataset of 1,118 taxa (1,116 species). The GenBank taxon names were updated to follow the linear fern classification^52^ with some modifications following the Pteridophyte Phylogeny Group classification^53^.

Sequences were aligned using default parameters in Mafft 6.864b^54^ with manual exclusion of ambiguously aligned segment in *rps4* gene, and a maximum likelihood (ML) tree was produced for concatenated data without partitioning in RAxML 7.3.0^55^ using the GTRCAT approximation and performing 500 rapid bootstrap replications followed by a thorough ML search. This tree was used as starting tree with birth-death model of diversification in exponential relaxed-clock divergence time estimation using BEAST 1.7.3^56^. All the nodes with >90% ML bootstrap support were constrained to be monophyletic in order to decrease the computational burden. The dataset was partitioned by the genes and the site, clock, and tree models were unlinked. Exponential prior distribution with a hard minimum and soft maximum age (allowing 5% probability to exceed the constraint) were assigned to 42 fossil calibration points (Supplementary Information). Whenever possible, we used fossils that have been included in phylogenetic analyses for time calibration. The fern crown group was calibrated with a minimum age of 359 Ma and a soft maximum age of 407 Ma. Eight independent chains were run until effective sample sizes exceeded 200 for the parameters sampled; this took 170–177 million generations sampling every 2,000 generations. The BEAST input file with all the parameter specifications is given as Supplementary Data 4. Effective sample sizes and burnin fraction were determined from log files in Tracer^50^. Stationarity was reached after the first 10% of the trees were discarded as burnin. The remaining trees were resampled to allow calculation of posterior probabilities from the retained 88,831 trees.

### Compilation and justification of palaeoenvironmental variables

We identified 17 factors that may have impacted fern diversification (Supplementary Figure 2). These include four biotic factors, namely (a) the diversity of ferns as estimated from the BDS analysis, (b) the diversity of free sporing vascular plants that are not part of the fern lineage (lycophytes etc.), (c) the diversity of gymnosperms and (d) angiosperms as modeled in a previous study^46^. Variable (b) was modeled on the basis of 2,159 fossil records from the Paleobiology Database (PBDB; https://paleobiodb.org/; Supplementary Data 5) using PyRate^49^. These data included 116 genera (114 of which are extinct) and ranged from 439.36 (+/−3.51) to 0.006 (+/−0.003) Ma. To model environmental effects, we extracted from the available paleobiome reconstructions^57^ the estimated Phanerozoic area of (e) wet tropical, (f) arid, (g) warm temperate, (h) cool temperate and (i) polar biome.

Our model also included (j) selenium (Se) concentration in marine sediments^43, 58^. Marine Se is derived from weathering and erosion of continental rocks and Se concentration in the ocean have been suggested to reflect variation in nutrient availability in continental systems^58^. The remaining variables are as follows: (k) continental fragmentation (scatter-index)^59^; (l) variation in the global mean temperature^60^; (m) eustatic sea level^61^; (n) the level of atmospheric CO_2_^62^; (o) the level of atmospheric O_2_^63^; (p) magmatic activity^64, 65^; and (q) mountain area. The Phanerozoic mountain area was extracted from the paleogeographic digital elevation models (paleoDEM, Supplementary Information). The paleoDEMs had a resolution of 1° cell and, after correcting for variation in sea level^61^, we calculated the total global surface area of grid cells with elevation >999 meters. Using this model and definition of mountain, the global mountain area of the present day world is measured as 22% of the terrestrial area. This is within the 12–25% mountain cover estimated using various roughness criteria^66, 67^.

The environmental variables were compiled from the raw values whenever possible, or if not available, the values were extracted from the published graphs using GraphClick (Arizona Software, http://www.arizona-software.ch/graphclick/). All the trajectories were rescaled to vary between 0–1 before analyses.

### Multivariate Birth-Death model

We developed a new birth-death model named Multivariate Birth-Death model (MBD) to assess to what extent biotic and abiotic factors can explain temporal variation in origination and extinction rates. Under the MBD model, origination and extinction rates can change through time (but equally across all lineages as in the BDS model) through correlations with time-continuous variables and the strength and sign (positive or negative) of the correlations are jointly estimated for each variable. We implemented two models, one with linear and the other with exponential correlations. The model with linear correlations is similar to the recently described Multiple Clade Diversity Dependence model (Equation 9 in Silvestro *et al*.^68^), where origination and extinction rates were modeled through linear correlations with the diversity trajectories of several clades. Here, we replace clade trajectories with our 17 variables, so that the origination and extinction rates at time t, λ(t) and μ(t) respectively, are:

1 $$\lambda (t)=max\{0,{\lambda }_{0}+{\lambda }_{0}\sum _{i=1}^{N}{G}_{i}{C}_{i}(t)\}$$

and

2 $$\mu (t)=max\{0,{\mu }_{0}+{\mu }_{0}\sum _{i=1}^{N}{H}_{i}{C}_{i}(t)\}$$

where, λ_0_ and μ_0_ are estimated baseline rates, C_1_, …, C_N_ are the 17 variables (i.e. N = 17), and G_1_, …, G_N_ and H_1_, …, H_N_ are the correlation parameters between each variable and origination and extinction rates, respectively. The correlation parameters can take negative values indicating negative correlation and positive values for positive correlations. When their value is estimated to be approximately zero, no correlation is estimated.

In the exponential correlation model we assume that origination and extinction rates may correlate exponentially with the variables (see Equation 7 in Silvestro *et al*.^68^ for a univariate example). Thus, the origination and extinction rates are based on the following transformations:

3 $$\lambda (t)=({\lambda }_{0}\exp \,\sum _{i=1}^{N}{G}_{i}{C}_{i}(t))$$

and

4 $$\mu (t)=({\mu }_{0}\exp \,\sum _{i=1}^{N}{H}_{i}{C}_{i}(t))$$

where G_1_, …, G_N_ and H_1_, …, H_N_ are the correlation parameters associated with each variable.

The MBD model with 17 variables includes 36 parameters, and assessing the strength and significance of each correlation or combination of correlations by explicit model testing is unfeasible. Instead, we implemented an MCMC algorithm to jointly estimate the baseline origination and extinction rates and all correlation parameters using a horseshoe prior^69^ to control for overparameterization and for the potential effects of multiple testing. The horseshoe prior provides an efficient approach to distinguish correlation parameters that should be treated as noise (and therefore shrunk around 0) from those that are significantly different from 0 and represent true signal. Under the horseshoe prior, the prior on the correlation parameters is a normal distribution with mean 0 and variance determined by two hyper-parameters *ε*_*i*_ (or ζ_*i*_) and τ. Thus, the prior for the given parameters G_*i*_ and H_*i*_ are:

5 $$P({G}_{i}|{{\epsilon }}_{i},\tau )\sim {\mathscr{N}}(0,{{\epsilon }}_{i}^{2}{\tau }^{2})$$

and

6 $$P({H}_{i}|{\zeta }_{i},\tau )\sim {\mathscr{N}}(0,{\zeta }_{i}^{2}{\tau }^{2})$$

Where the hyper-parameters ε_*i*_ (or ζ_*i*_) and τ control the shrinkage or release of each correlation parameter and are assigned half Cauchy hyper-prior distributions:

7 $${{\epsilon }}_{i}\sim {{\mathscr{C}}}^{+}(0,1){\zeta }_{i}\sim {{\mathscr{C}}}^{+}(0,1)$$

and

8 $$\tau \sim {{\mathscr{C}}}^{+}(0,1)$$

This parameterization combines local (ε_*i*_, *ζ*_*i*_) and global (τ) Bayesian shrinkage parameters^69^ and was recently shown by extensive simulations to yield accurate results under the Multiple Clade Diversity Dependence birth-death model^70^. Based on the estimated local and global shrinkage parameters we calculated the shrinkage weights for each correlation parameter, e.g.

9 $$w(({G}_{i})=1-1/(1+{\tau }^{2}{{\epsilon }}^{2})$$

and

10 $$w({H}_{i})=1-1/(1+{\tau }^{2}{\zeta }^{2})$$

Although shrinkage weights do not represent actual probabilities, they provide a robust measure to distinguish between noise and signal^69^. In particular, estimated shrinkage weights exceeding 0.5 indicate significant support for the corresponding correlation parameter (as also demonstrated through simulations^70^). For instance if *w*(G_*i*_) > 0.5, then G_*i*_ significantly differs from the background noise and represents a positive or negative correlation. Thus, with the MBD model and the horseshoe prior algorithm we can infer in a single analysis which and how many variables can significantly explain variations in origination and extinction rates and their sign (positive or negative) and intensity.

We ran the MBD model using 55,000,000 MCMC iterations and sampling every 50,000 to approximate the posterior distribution of all parameters (Supplementary Tables 4 and 5). We summarized the results of the MBD analyses by calculating the posterior mean and 95% HPD of all correlation parameters and the mean of the respective shrinkage weights (across 100 replicates), as well as the mean and 95% HPD of the baseline origination and extinction rates. Although the current implementation of the MBD model does not allow for formal estimation of marginal likelihoods to compare the fit linear against exponential model (e.g. using path sampling^71^), we used harmonic means of the log likelihoods sampled through MCMC under the two models to approximate their relative support^72^. We calculated log Bayes factors as two times the difference between the log marginal likelihood of the linear versus the exponential model and compared it against the threshold described by Kass and Raftery^72^. Bayes factors indicated strong support in favour of exponential correlation against linear correlation (mean log BF = 22.16 st. dev. 10.22). Finally, based on the best fitting MBD models, we generated a rates-through-time plots depicting how origination and extinction rates are inferred to change through time as a function of the joint effects of strong and weak correlations with all 17 variables (Fig. 4c). We calculated the correlation coefficient (R^2^) between the rates through time estimated under BDS (within fixed time bins, but without any explicit assumptions about their dynamics) and the two MBD models. We interpreted a good match between estimates (hence a high R^2^ value) as an indication that the MBD model and the set of variables included in the analysis provided an adequate framework to estimate rate variation and were able to capture the main trends in origination and extinction rates. The correlation coefficients were 0.74 for origination rates and 0.65 for extinction rates under the exponential MBD model, whereas they were 0.68 and 0.57 under the linear MBD model. While these values indicate that both models could capture most of the rate variation estimated under the BDS model, they also suggest that the exponential model might be the most appropriate here, thus confirming the results of Bayes factors.

### Macroevolutionary modelling of extant species

We performed a BAMM v2.5.0 (http://bamm-project.org/)^73^ analysis on the phylogeny to investigate fern diversification dynamics in relation to their modern ecology. The incomplete sampling was accounted for by estimating the sampling percentage of each genus. We ran four chains of 100 million generations of reversible-jump Metropolis coupled MCMC with samples drawn from the posterior every 100,000 generations and swaps between chains every 1,000 generations. Although recent concerns have been raised concerning the use of BAMM is such analyses^74^, these may only apply to the use of non-standard analytical settings^75^. Post-run analyses were performed in the R package ‘BAMMtools’ version 2.1.0^76^. The estimated speciation and extinction rates are shown in Fig. 2.

To model the rate of ecological adaptation we created a global climatic niche layer on the basis of six climatic layers, CHELSA_bio1_1979-2013_v1_1 (annual mean temperature), CHELSA_bio6_1979-2013_v1_1 (min temperature of coldest month), CHELSA_bio12_1979-2013_v1_1 (annual precipitation), CHELSA_bio15_1979-2013_v1_1 (precipitation seasonality), CHELSA_prec_interannual_1979-2013_v1_1 (interannual precipitation variation), and CHELSA_temp_interannual_1979-2013_V1_1 (interannual temperature variation)^77^. Temperature and precipitation with their seasonal variations are among the most relevant climatic parameters determining fern occurrences^78^. The climatic variation was summarized into a single layer with the resolution of 10 arc-minutes by performing a principal component analysis (PCA) on global data after rescaling all the values, and mapping the first principal component (with 47% explained proportion of variance) in R package ‘raster’^79^ (Supplementary Figure 5). We then downloaded occurrence data of fern species represented in our phylogeny from GBIF (Global Biodiversity Information Facility; http://www.gbif.org) and automatically cleaned the data by removing erroneous or suspicious geographic coordinates (coordinates that were non-valid, in the sea, zero, not located in the reported country of origin, or corresponded to country capitals, country centroids, or GBIF headquarter) using R packages ‘rgbif’^80^ and ‘speciesgeocodeR’^81^. The taxonomy between GBIF and our tree was cross-checked resulting in 935 species with occurrence data; for these we calculated a mean value of their climatic niche from the PCA layer. We then modelled the rate of niche evolution through the fern phylogeny as a continuous phenotypic trait in BAMM. Species without niche data were pruned from the tree and analysis was run as above. The most frequent rate shift configuration (93% of the samples in the posterior distribution) supported zero rate shifts (Supplementary Figure 4).

We applied a structured permutation test (STRAPP) in BAMMtools^24^ to identify whether epiphytic life strategy or rate of molecular evolution is associated with estimated speciation and extinction rate changes. All the terminal taxa represented in the phylogeny were coded as ‘epiphytic’ or ‘non-epiphytic’ based on various botanical literature sources or our field experience. A large number of species can grow terrestrially or epiphytically depending on the local conditions and were coded according to our best knowledge of the prevailing growth mode. This coding resulted in 488 epiphytic and 630 non-epiphytic terminal taxa in the permutation test. To correlate the rate of molecular evolution the median substitution rates for each tip of the tree were extracted using the R package ‘phytools’^82^ and coded as continuous character for the test. In all cases we estimated the significance of correlation by performing 10,000 STRAPP^24^ replications. We did not find any significant correlations, but it should be noted that our tree is barely large enough to detect significant correlations even if they exist^24^.

### Comparison of molecular and fossil age estimates

We calculated the minimum divergence incongruence (MID)^83^ metric for each genus represented in both the palaeontological and neontologial datasets (*n* = 76; Supplementary Table 3). For the origination of fossil taxa we obtained 95% highest posterior density (HPD) intervals directly from our PyRate runs, but in the molecular phylogeny the corresponding origination may have occurred at any point between the stem and crown group nodes. We therefore compared the fit of fossil-based 95% HPD interval with the molecular-based interval from 95% maximum stem age to 95% minimum crown age.

### Code availability

The MBD implementation was built within the PyRate software package^49, 70^ and is available at https://github.com/dsilvestro/PyRate.

### Data availability

Data available from Zenodo DOI: 10.5281/zenodo.345670. Fossil occurrences, input file for phylogenetic analysis, and the resulting time calibrated phylogeny are also available as Supplementary Data, and phylogenies, sequence alignments and input file for dating analysis in TreeBASE (http://purl.org/phylo/treebase/phylows/study/TB2:S20675).

## Electronic supplementary material

Supplementary Information

Dataset 1

Dataset 2

Dataset 3

Dataset 4

Dataset 5

## References

[1] Benton MJ (2009). The Red Queen and the Court Jester: species diversity and the role of biotic and abiotic factors through time. Science.

[2] Ezard THG, Aze T, Pearson PN, Purvis A (2011). Interplay between changing climate and species’ ecology drives macroevolutionary dynamics. Science.

[3] Benton, M. J. Exploring macroevolution using modern and fossil data. *Proc R Soc B* **282**, 20150569 (2015). 10.1098/rspb.2015.0569 PMC4590474 26063844

[4] Niklas KJ, Tiffney BH, Knoll AH (1983). Patterns in vascular land plant diversification. Nature.

[5] Rothwell GW (1996). Pteridophytic evolution: an often underappreciated phytological success story. Rev. Palaeobot. Palynol..

[6] Tidwell WD, Ash SR (1994). A review of selected triassic to Early Cretaceous ferns. J. Plant Res..

[7] Testo W, Sundue M (2016). A 4000-species dataset provides new insight into the evolution of ferns. Mol. Phylogenet. Evol..

[8] Lehtonen S (2011). Towards resolving the complete fern tree of life. PLoS ONE.

[9] Rothfels CJ (2015). The evolutionary history of ferns inferred from 25 low-copy nuclear genes. Am. J. Bot..

[10] Rai HS, Graham SW (2010). Utility of a large, multigene plastid data set in inferring higher-order relationships in ferns and relatives (monilophytes). Am. J. Bot..

[11] Schuettpelz E, Pryer KM (2007). Fern phylogeny inferred from 400 leptosporangiate species and three plastid genes. Taxon.

[12] Schneider H (2004). Ferns diversified in the shadow of angiosperms. Nature.

[13] Schuettpelz E, Pryer KM (2009). Evidence for a Cenozoic radiation of ferns in an angiosperm-dominated canopy. Proc. Natl. Acad. Sci..

[14] Van Valen L (1973). A new evolutionary law. Evol. Theory.

[15] Fiz-Palacios O, Schneider H, Heinrichs J, Savolainen V (2011). Diversification of land plants: insights from a family-level phylogenetic analysis. BMC Evol. Biol..

[16] Scott AC, Galtier J (1985). Distribution and ecology of early ferns. Proc. R. Soc. Edinb. Sect. B Biol. Sci..

[17] Barnosky AD (2001). Distinguishing the effects of the Red Queen and Court Jester on Miocene mammal evolution in the northern Rocky Mountains. J. Vertebr. Paleontol..

[18] Knie N, Fischer S, Grewe F, Polsakiewicz M, Knoop V (2015). Horsetails are the sister group to all other monilophytes and Marattiales are sister to leptosporangiate ferns. Mol. Phylogenet. Evol..

[19] Magallón S, Hilu KW, Quandt D (2013). Land plant evolutionary timeline: gene effects are secondary to fossil constraints in relaxed clock estimation of age and substitution rates. Am. J. Bot..

[20] Gensel PG (2008). The earliest land plants. Annu. Rev. Ecol. Evol. Syst..

[21] Rothwell GW (1987). Complex Paleozoic Filicales in the evolutionary radiation of ferns. Am. J. Bot..

[22] Galtier J, Scott AC (1985). Diversification of early ferns. Proc. R. Soc. Edinb. Sect. B Biol. Sci..

[23] Bomfleur B, McLoughlin S, Vajda V (2014). Fossilized nuclei and chromosomes reveal 180 million years of genomic stasis in royal ferns. Science.

[24] Rabosky DL, Huang H (2016). A robust semi-parametric test for detecting trait-dependent diversification. Syst. Biol..

[25] Sundue MA, Testo WL, Ranker TA (2015). Morphological innovation, ecological opportunity, and the radiation of a major vascular epiphyte lineage. Evolution.

[26] Rabosky DL (2010). Extinction rates should not be estimated from molecular phylogenies. Evolution.

[27] Roy K, Jablonski D, Valentine J (1996). Higher taxa in biodiversity studies: patterns from eastern Pacific marine molluscs. Philos. Trans. R. Soc. Lond. B Biol. Sci..

[28] Hedges SB, Marin J, Suleski M, Paymer M, Kumar S (2015). Tree of life reveals clock-like speciation and diversification. Mol. Biol. Evol..

[29] Hubbell, S. P. The Unified Neutral Theory of Biodiversity and Biogeography. (Princeton University Press, 2001). 10.1016/j.tree.2011.03.024 21561679

[30] Jablonski D (2008). Species selection: theory and data. Annu. Rev. Ecol. Evol. Syst..

[31] Cardoso GC, Cortesão M, García C (2015). Ecological marginalization facilitated diversification in conifers. Evol. Biol..

[32] Moen D, Morlon H (2014). Why does diversification slow down?. Trends Ecol. Evol..

[33] Venditti C, Meade A, Pagel M (2010). Phylogenies reveal new interpretation of speciation and the Red Queen. Nature.

[34] Simpson, G. G. *Tempo and mode in evolution*. (Columbia University Press, 1944).

[35] Pigot AL, Tobias JA (2013). Species interactions constrain geographic range expansion over evolutionary time. Ecol. Lett..

[36] Karger DN (2015). The importance of species pool size for community composition. Ecography.

[37] Price TD (2014). Niche filling slows the diversification of Himalayan songbirds. Nature.

[38] Wiens JJ (2010). Niche conservatism as an emerging principle in ecology and conservation biology. Ecol. Lett..

[39] Lehtonen S, Jones MM, Zuquim G, Prado J, Tuomisto H (2015). Phylogenetic relatedness within Neotropical fern communities increases with soil fertility. Glob. Ecol. Biogeogr..

[40] Ricklefs RE (2010). Host–pathogen coevolution, secondary sympatry and species diversification. Philos. Trans. R. Soc. Lond. B Biol. Sci..

[41] Brodribb TJ, McAdam SAM (2011). Passive origins of stomatal control in vascular plants. Science.

[42] Parrish JT (1993). Climate of the supercontinent Pangea. J. Geol..

[43] Long JA (2015). Severe selenium depletion in the Phanerozoic oceans as a factor in three global mass extinction events. Gondwana Res..

[44] Watkins JE, Cardelús CL (2012). Ferns in an angiosperm world: Cretaceous radiation into the epiphytic niche and diversification on the forest floor. Int. J. Plant Sci..

[45] Rothwell GW, Nixon KC (2006). How does the inclusion of fossil data change our conclusions about the phylogenetic history of euphyllophytes?. Int. J. Plant Sci..

[46] Silvestro D, Cascales-Miñana B, Bacon CD, Antonelli A (2015). Revisiting the origin and diversification of vascular plants through a comprehensive Bayesian analysis of the fossil record. New Phytol..

[47] Walker JD, Geissman JW (2009). Geologic time scale. GSA Today.

[48] Silvestro D, Schnitzler J, Liow LH, Antonelli A, Salamin N (2014). Bayesian estimation of speciation and extinction from incomplete fossil occurrence data. Syst. Biol..

[49] Silvestro D, Salamin N, Schnitzler J (2014). PyRate: a new program to estimate speciation and extinction rates from incomplete fossil data. Methods Ecol. Evol..

[50] Rambaut, A. & Drummond, A. J. http://beast.bio.ed.ac.uk/Tracer. (2007).

[51] Sanderson MJ, Boss D, Chen D, Cranston KA, Wehe A (2008). The PhyLoTA Browser: processing GenBank for molecular phylogenetics research. Syst. Biol..

[52] Christenhusz M, Zhang X, Schneider H (2011). A linear sequence of extant families and genera of lycophytes and ferns. Phytotaxa.

[53] PPG I. (2016). A community-derived classification for extant lycophytes and ferns. J. Syst. Evol..

[54] Katoh K, Misawa K, Kuma K, Miyata T (2002). MAFFT: a novel method for rapid multiple sequence alignment based on fast Fourier transform. Nucleic Acids Res..

[55] Stamatakis A (2006). RAxML-VI-HPC: maximum likelihood-based phylogenetic analyses with thousands of taxa and mixed models. Bioinformatics.

[56] Drummond AJ, Suchard MA, Xie D, Rambaut A (2012). Bayesian phylogenetics with BEAUti and the BEAST 1.7. Mol. Biol. Evol..

[57] Boucot, A. J., Chen, X. & Scotese, C. R. *Phanerozoic paleoclimate: an atlas of lithologic indicators of climate*. (Society for Sedimentary Geology, 2013).

[58] Large RR (2015). Cycles of nutrient trace elements in the Phanerozoic ocean. Gondwana Res..

[59] Cogné J-P, Humler E (2008). Global scale patterns of continental fragmentation: Wilson’s cycles as a constraint for long-term sea-level changes. Earth Planet. Sci. Lett..

[60] Scotese, C. R. Some thoughts on global climate change: the transition from icehouse to hothouse, in the Earth history: the evolution of the Earth system. https://www.academia.edu/12082909/. (PALEOMAP Project, Evanston, IL., 2016).

[61] Snedden, J. W. & Liu, C. A compilation of Phanerozoic sea-level change, coastal onlaps and recommended sequence designations. *Search and Discovery Article* 40594 (2010).

[62] Berner RA, Kothavala Z (2001). Geocarb III: a revised model of atmospheric CO2 over Phanerozoic time. Am. J. Sci..

[63] Berner RA (2009). Phanerozoic atmospheric oxygen: new results using the GEOCARBSULF model. Am. J. Sci..

[64] Gastil RG (1960). The distribution of mineral dates in time and space. Am. J. Sci..

[65] Brink H-J (2015). Periodic signals of the Milky Way concealed in terrestrial sedimentary basin fills and in planetary magmatism?. Int. J. Geosci..

[66] Körner C, Paulsen J, Spehn EM (2011). A definition of mountains and their bioclimatic belts for global comparisons of biodiversity data. Alp. Bot..

[67] Meybeck M, Green P, Vörösmarty C (2001). A new typology for mountains and other relief classes. Mt. Res. Dev..

[68] Silvestro D, Antonelli A, Salamin N, Quental TB (2015). The role of clade competition in the diversification of North American canids. Proc. Natl. Acad. Sci..

[69] Carvalho CM, Polson NG, Scott JG (2010). The horseshoe estimator for sparse signals. Biometrika.

[70] Silvestro D, Pires MM, Quental TB, Salamin N (2017). Bayesian estimation of multiple clade competition from fossil data. Evol. Ecol. Res..

[71] Xie W, Lewis PO, Fan Y, Kuo L, Chen M-H (2010). Improving marginal likelihood estimation for Bayesian phylogenetic model selection. Syst. Biol..

[72] Kass RE, Raftery AE (1995). Bayes Factors. J. Am. Stat. Assoc..

[73] Rabosky DL (2014). Automatic detection of key innovations, rate shifts, and diversity-dependence on phylogenetic trees. PLoS ONE.

[74] Moore, B. R., Höhna, S., May, M. R., Rannala, B. & Huelsenbeck, J. P. Critically evaluating the theory and performance of Bayesian analysis of macroevolutionary mixtures. *Proc. Natl. Acad. Sci*. 201518659 (2016). 10.1073/pnas.1518659113 PMC5003228 27512038

[75] Rabosky, D. L., Mitchell, J. S. & Chang, J. Is BAMM flawed? Theoretical and practical concerns in the analysis of multi-rate diversification models. *Syst. Biol*. doi:https://doi.org/10.1093/sysbio/syx037 (2017). 10.1093/sysbio/syx037 PMC5790138 28334223

[76] Rabosky DL (2014). BAMMtools: an R package for the analysis of evolutionary dynamics on phylogenetic trees. Methods Ecol. Evol..

[77] Karger, D. N. *et al*. Climatologies at high resolution for the earth’s land surface areas. *arXiv:1607.00217v2.1*. (2016). 10.1038/sdata.2017.122 PMC5584396 28872642

[78] Kreft H, Jetz W, Mutke J, Barthlott W (2010). Contrasting environmental and regional effects on global pteridophyte and seed plant diversity. Ecography.

[79] Hijmans, R. J. *et al*. raster: geographic data analysis and modeling. (2015).

[80] Chamberlain, S., Boettiger, C., Ram, K., Barve, V. & Mcglinn, D. rgbif: Interface to the Global Biodiversity Information Facility API. R package version 0.9.3. (2016).

[81] Töpel M (2017). SpeciesGeoCoder: Fast Categorization of Species Occurrences for Analyses of Biodiversity, Biogeography, Ecology, and Evolution. Syst. Biol..

[82] Revell L (2012). J. phytools: an R package for phylogenetic comparative biology (and other things). Methods Ecol. Evol..

[83] Clarke JA, Boyd CA (2015). Methods for the quantitative comparison of molecular estimates of clade age and the fossil record. Syst. Biol..

[84] R Core Team. *R: A language and environment for statistical computing*. (R Foundation for Statistical Computing, 2015).

[85] Scotese, C. R. PALEOMAP PaleoAtlas for GPlates and the PaleoData Plotter Program, PALEOMAP Project, http://www.earthbyte.org/paleomap-paleoatlas-for-gplates/. (2016).

[86] Scotese, C. R. PointTracker, PALEOMAP Project, University of Texas at Arlington. (2004).

