# Supplementary material for: Environmentally driven extinction and opportunistic origination explain fern diversification patterns
Source: Sci Rep. 2017 Jul 6;7:4831. doi: 10.1038/s41598-017-05263-7 (PMC5500532; doi:10.1038/s41598-017-05263-7)
Supplement: Supplementary file 1 — Supplementary Information [file 41598_2017_5263_MOESM1_ESM.pdf]

Environmentally driven extinction and opportunistic origination explain fern diversification patterns

Samuli Lehtonen*[1,2†], Daniele Silvestro*[3,4,5,6†], Dirk Nikolaus Karger[2,7], Christopher Scotese[8], Hanna Tuomisto[2], Michael Kessler[7], Carlos Peña[2], Niklas Wahlberg[2,9], Alexandre Antonelli[3,4,10]

[1]Herbarium, Biodiversity Unit, University of Turku, 20014 Turku, Finland

[2]Department of Biology, University of Turku, 20014 Turku, Finland

[3]Department of Biological and Environmental Sciences, University of Gothenburg, Carl Skottsbergs gata 22B, Gothenburg 413 19, Sweden

[4]Gothenburg Global Biodiversity Centre, Box 461, SE-405 30 Göteborg, Sweden

[5]Department of Ecology and Evolution, University of Lausanne, 1015 Lausanne, Switzerland

[6]Swiss Institute of Bioinformatics, Quartier Sorge, 1015 Lausanne, Switzerland

[7]Department of Systematic and Evolutionary Botany, University of Zurich, 8008 Zurich, Switzerland

[8]Earth and Planetary Sciences, Northwestern University, Evanston, IL, USA

[9]Department of Biology, Lund University, Lund, Sweden

[10]Gothenburg Botanical Garden, Carl Skottsbergs gata 22A, Gothenburg 413 19, Sweden

[†]These authors contributed equally to this work.

***Correspondence to:** samuli.lehtonen@utu.fi or daniele.silvestro@bioenv.gu.se

Supplementary Information

Supplementary Materials and Methods

PaleoDEMs

The paleogeographic digital elevation models[1] were built on the basis of digital topographic and bathymetric datasets of the modern world[2–4]. These data were combined into a global dataset with 6-minute resolution. The individual grid cells were rotated to their paleopositions using the PALEOMAP Global Plate Tectonic Model[5], and the modern digital topographic and bathymetric values were corrected and modified using the lithofacies and paleoenvironmental data. These data were constructed using the database of Paleogeographic Atlas Project[6–8] supplemented by additional lithological and paleoenvironmental records for the Permian and Jurassic[9,10] in combination with numerous regional and global paleogeographic atlases. The plate tectonic reconstructions[11] were used to model the expected changes in topography and bathymetry caused by plate tectonic events, such as sea floor spreading, continental rifting, subduction, and continental collision, as well as other isostatic events such as glacial rebound[12].

Fossil calibration priors for molecular dating

1. Ferns

Hard minimum age constraint: 359 Ma. *Archaeocalamites* from Upper Devonian to Lower Permian[13,14] is considered as a member of the horsetail lineage[15] and used in several studies to constrain the first divergence within ferns[16–18]. We follow this practice to constrain the minimum age of crown group ferns.

Soft maximum age constraint: 407 Ma. Oldest known Euphyllophytina are from Lower Devonian[19], and we used this age as the soft maximum age for the ferns.

2. ***Equisetum***

Hard minimum age constraint: 359 Ma. *Archaeocalamites* is considered a member of the horsetail lineage[15] and we used their first appearance to constrain the minimum age of the *Equisetum* stem lineage.

Soft maximum age constraint: 407 Ma. We used the oldest known Euphyllophytina[19] as the soft maximum for the stem lineage.

3. ***Tmesipteris***

Hard minimum age constraint: 23 Ma. The Oligocene fossil *Tmesipteris tasmanica* is lacking reproductive structures, but represents the distinctive vegetative characteristics of *Tmesipteris*[20]. We assigned the constraint for the crown group *Tmesipteris* because the fossil is associated with extant species having indefinite growth type[20].

Soft maximum age constraint: 145.5 Ma. Whisk ferns have extremely poor fossil record (Collinson 1996), but are among the first diverging fern lineages[15,17,21]. In the lack of better fossil constraint we applied the beginning of Cretaceous as the soft maximum age for this node.

4. ***Botrychium***

Hard minimum age constraint: 55.8 Ma. Fossil trophophores, sporophores and spores were assigned to the genus *Botrychium* with high confidence[22]. The fossil was considered remarkably similar with extant *B. virginianum*[22], a species that was resolved together with *B. strictum* as the sister lineage of all other living *Botrychium*[21]. A detailed cladistic analysis incorporating morphological characters would be needed to verify whether *B. wrightonii* represents a crown group *Botrychium*, or a stem group. We took a conservative approach and allowed the fossil to represent the stem group.

Soft maximum age constraint: 201.6 Ma. Ophioglossales have extremely poor fossil record[22], but are among the first diverging fern lineages[21]. The split between *Botrychium* and *Ophioglossum* have

previously been estimated to have occurred ca. 162 Mya[17]. Without a better judgement we applied the Triassic-Jurassic boundary as the soft maximum age for the stem lineage.

5. Marattiaceae

Hard minimum age constraint: 318 Ma. Marattioid fossils are abundant from Pennsylvanian onwards, but poorly preserved specimens are known already from the Mississippian[13]. We assigned the constraint to the Marattiaceae stem lineage.

Soft maximum age constraint: 407 Ma. We used the oldest known Euphyllophytina[19] as the soft maximum for the stem lineage, as the ancestors of marattioids remain unknown[23].

6. *Ptisana*

Hard minimum age constraint: 176 Ma. *Marattia asiatica* is here considered as a member of segregate genus *Ptisana*[24]. The species has sessile, deeply cut bivalve synangia with thick outer walls[25], characters typical of *Ptisana*[24]. These characters may represent plesiomorphies rather than apomorphies[26]. However, Middle Jurassic *Angiopteris blackii* has free synangia and trilete spores[27], which are considered to be apomorphic states[26]. Therefore, it appears that *Ptisana* and *Angiopteris* were separated by the end of the Early Jurassic. We constrained the *Ptisana* stem lineage to have originated by then.

Soft maximum age constraint: 318 Ma. A cladistic analysis based on morphological data suggested that the Early Permian *Millaya* may belong to crown group marattioids[23]. We applied the beginning of Pennsylvanian as the soft maximum age for the stem lineage, as marattioids became common during this period, but modern lineages apparently had not evolved yet[23].

7. *Angiopteris*

Hard minimum age constraint: 172 Ma. We use the Middle Jurassic *Angiopteris blackii*[27] to constrain the age of the stem lineage.

Soft maximum age constraint: 318 Ma. We applied the same soft maximum age constraint for *Angiopteris* as for *Ptisana*.

8. Leptosporangiate ferns

Hard minimum age constraint: 345 Ma. Tournaisian sporangia of *Senftenbergia*-type are placed in Tedeleaceae[28], a group of ferns resolved as stem group leptosporangiates[15]. We assigned the constraint to the leptosporangiate stem lineage.

Soft maximum age constraint: 407 Ma. Oldest known Euphyllophytina are from Lower Devonian[19], and we used this age as the soft maximum age for leptosporangiate stem lineage.

9. Osmundaceae

Hard minimum age constraint: 271 Ma. Early Permian *Grammatopteris* is generally considered as an early member of osmundaceous ferns[16,17,29–31]. We assigned the constraint to the Osmundaceae stem lineage.

Soft maximum age constraint: 345 Ma. The hard minimum age of leptosporangiates was used as the soft maximum age for Osmundaceae stem lineage.

10. *Osmunda*

Hard minimum age constraint: 201.6 Ma. The Triassic fossil *Osmunda claytoniites* was described as remarkably similar to modern *Osmunda*[32]. The fossil shows medially positioned hemidimorphic leaves which are characteristic to *Osmunda* subgenus *Claytosmunda*, thus providing a minimum age for the genus[32,33]. We used this fossil to constrain the crown group age.

Soft maximum age constraint: 299 Ma. Members of Osmundaceae appeared first time in the Permian[34,35].

11. *Osmundastrum*

Hard minimum age constraint: 65.5 Ma. Fossils indistinguishable from the modern *Osmundastrum cinnamomeum* are known from the Upper Cretaceous[36]. We assigned the constraint to the stem lineage of the clade representing two *O. cinnamomeum* varieties in our analysis.

Soft maximum age constraint: 299 Ma. The soft maximum age of *Osmunda* was applied to the stem lineage of the subgenus.

12. *Osmunda* subg. *Plenasium*

Hard minimum age constraint: 55.8 Ma. The Paleocene *Osmunda arnoldii* is very similar to extant species of subgenus *Plenasium*[34,37]. We assigned the constraint to the stem lineage of subgenus *Plenasium*.

Soft maximum age constraint: 299 Ma. The soft maximum age of *Osmunda* was applied to the stem lineage of the subgenus.

13. *Todea*

Hard minimum age constraint: 136 Ma. Phylogenetic analysis combining morphological data and *rbcL* sequences resolved the fossil species *Todea tidwellii* as a member of *Todea*[35]. *Todea* and *Leptopteris* are morphologically readily distinguishable and strongly supported as each others closest relatives in molecular phylogeny[33], and we used this fossil to constrain the *Todea-Leptopteris* node excluding the stem.

Soft maximum age constraint: 299 Ma. Members of Osmundaceae appeared first time in the Permian[34,35].

14. Hymenophyllaceae

Hard minimum age constraint: 228 Ma. *Hopetedia praetermissa* is the oldest known member of Hymenophyllaceae based on its general frond morphology, indirect evidence of a filmy habit, and soral

position and morphology[38]. This fossil has been used to constrain filmy fern origin previously as well[16]. We assigned the constraint to the Hymenophyllaceae stem lineage.

Soft maximum age constraint: 318 Ma. *Hymenophyllites quadridactylites* from the Upper Carboniferous is the oldest known putative Hymenophyllaceae[38], and the beginning of Pennsylvanian is used here as the soft maximum age for the stem lineage.

15. Matoniaceae

Hard minimum age constraint: 235 Ma. Structurally preserved sori and sporangia of *Tomaniopteris katonii* has a set of characters unique to Matoniaceae[39]. This constraint has been applied also in previous studies[31]. We assigned the constraint to the Matoniaceae stem lineage.

Soft maximum age constraint: 299 Ma. *Oligocarpia* and *Szea* from Permian to Triassic have been considered as possible stem group members of Gleicheniaceae, Matoniaceae, Dipteridaceae, or Hymenophyllaceae[16]. The Carboniferous-Permian boundary was used here as soft maximum age for the stem lineage.

16. Gleicheniaceae

Hard minimum age constraint: 99.6 Ma. Morphology based cladistic analysis resolved *Gleichenia chaloneri* as the sister of extant *Diplopterygium glaucum* (=*Gleichenia glauca*)[40]. However, that analysis suffered from poor taxon sampling and failed to cover the extant diversity within Gleicheniaceae. As well, their results are different from those of a previous study[41] and neither one of these studies is supported by the molecular results[21]. Better sampled morphological data matrices would be needed to verify the exact position of *Gleichenia chaloneri* and another fossil taxa, *Boodlepteris turoniana*[41], within the family. However, it seems apparent that both of these fossil taxa represent members of crown group Gleicheniaceae, and following a previous study[31] we used the older one of them (*G. chaloneri*) to constrain the Gleicheniaceae crown group.

Soft maximum age constraint: 299 Ma. Justification as for the Matoniaceae.

17. *Stromatopteris*

Hard minimum age constraint: 89.3 Ma. Morphology based cladistic analyses[40,41] resolved *Boodlepteris* as the sister of extant *Stromatopteris*. Both of these studies suffered from poor taxon sampling and failed to cover the extant diversity within Gleicheniaceae and their topology is not supported by the molecular results. However, *Boodlepteris* and *Stromatopteris* share anatomical apomorphies not observed in other members of the family, and we follow a previous study[31] in using this calibration point. We applied the constraint for the *Stromatopteris moniliformis–Gleichenella pectinata* node.

Soft maximum age constraint: 299 Ma. The soft maximum age of the family was applied to this node.

18. Lygodiaceae

Hard minimum age constraint: 168 Ma. It has been suggested that mid-Jurassic *Stachypteris* provides a minimum age for the divergence between *Lygodium* and *Anemia*-*Schizaea*[42]. Similarities in fertile structures suggests affinity with modern *Lygodium*, despite the highly different frond morphology and habit[42]. We assigned the constraint to the Lygodiaceae stem lineage following a previous study[16].

Soft maximum age constraint: 299 Ma. Fossil evidence suggests that the second filicalean radiation, into which Lygodiaceae belongs, began in Permian time[43]. Carboniferous-Permian boundary is applied here as the soft maximum age for the stem lineage.

19. *Schizaea*

Hard minimum age constraint: 112 Ma. Fossil *Schizaeopsis* is very similar to living *Schizaea* in frond morphology, but differs in its spore morphology[42,44,45]. *Schizaeopsis* has been considered as the sister lineage of *Schizaea*[42]. The oldest known fossils are from the Lower Cretaceous[45]. We assigned the constraint to the *Schizaea* stem lineage.

Soft maximum age constraint: 251 Ma. Spores with a possible affinity with *Schizaea* first appear in the Triassic[42] and consequently we applied the beginning of Triassic as the soft maximum age for the stem lineage.

20. *Anemia*

Hard minimum age constraint: 136 Ma. The anatomically preserved fossils were assigned to the genus *Anemia*, and compared most closely to subgenus *Anemiorrhiza*[46]. *Anemiorrhiza* is the sister group to a clade comprising *Mohria* and the rest of the *Anemia*[42]. Because the fossil differed from *Mohria* and presented characters suggesting affinity with *Anemiorrhiza*[46], we used it to constrain the basal split of *Anemiorrhiza* from the remaining clade. Further evidence to support this calibration point is provided by Cretaceous *Pelletixia*, *Ruffordia* and *Anemia* fossils which have been variously resolved within the modern *Anemia* subgroups in phylogenetic analyses[42,47,48]. This node was constrained as Valanginian also in a previous study[31]. We assigned the constraint to the *Anemia* stem lineage.

Soft maximum age constraint: 251 Ma. *Anemia* and *Schizaea* are sister groups[42] and we apply the same soft maximum age for both of them.

21. Marsileaceae

Hard minimum age constraint: 140 Ma. Late Jurassic-Early Cretaceous *Regnellites* was resolved as a member of Marsileaceae in a cladistic analysis of morphological data[49]. This calibration has also been used before[31]. We assigned the constraint to the Marsileaceae stem lineage.

Soft maximum age constraint: 201.6 Ma. Oldest fossils possible related to Marsileaceae are Late Jurassic microspores[50,51]. We therefore applied Triassic-Jurassic boundary as the soft maximum age for the stem lineage.

22. *Marsilea*

Hard minimum age constraint: 99.6 Ma. *Marsileaceaephyllum mahisensis* was nested within *Marsilea* in cladistic analysis of morphological data[51]. Taxon sampling was poor in that analysis, but the

relationships within *Marsilea* were in accordance with the molecular hypothesis[52]. We assigned the constraint to the *Marsilea* stem lineage.

Soft maximum age constraint: 145.5 Ma. Based on fossil record it has been estimated that modern Marsileaceae genera started to differentiate at mid-Cretaceous[50]. We used the beginning of Cretaceous as the soft maximum age for the stem lineage.

23. *Regnellidium*

Hard minimum age constraint: 83.5 Ma. Fossils from the Late Cretaceous were assigned to *Regnellidium* based on preserved sporocarp, megaspore and microspore characters[50]. This calibration point has been used also in a previous study[31]. We applied this constraint to the *Regnellidium diphyllum–Pilularia globulifera* node.

Soft maximum age constraint: 145.5 Ma. Based of fossil record it has been estimated that modern Marsileaceae genera started to differentiate at mid-Cretaceous[50] and we assigned the beginning of Cretaceous as the soft maximum age for this node.

24. *Azolla*

Hard minimum age constraint: 83.5 Ma. Earliest fossil *Azolla* megaspores appear in the Santonian[53]. We applied this constraint for the *Azolla* stem lineage.

Soft maximum age constraint: 201.6 Ma. The soft maximum age of Marsileaceae (sister family of Salviniaceae, in which *Azolla* belongs) was applied as the maximum age for *Azolla* stem lineage.

25. *Azolla* section *Azolla*

Hard minimum age constraint: 13.8 Ma. The minimum age of this section was constrained following a previous study[54]. The age constraint was applied to the section including the stem.

Soft maximum age constraint: 33.9 Ma. All modern *Azolla* share a collared megaspore, a character that is observed in the fossil record first time in the Oligocene[54]. The age constraint was applied to the stem lineage.

26. Loxsomataceae

Hard minimum age constraint: 112 Ma. Relationship of the Aptian *Loxsomopteris anasilla* rhizome with Loxsomataceae was suggested by similarity of the hairs covering the rhizome[55]. We assigned the constraint to the Loxsomataceae stem lineage.

Soft maximum age constraint: 251 Ma. Triassic fossils have often been associated with tree ferns[56], although it is possible that they represent extinct stem lineages of tree ferns or tree ferns and Polypodiales[17]. Molecular clock suggested that the divergence between Polypodiales and tree ferns occured in Late Triassic[16]. We apply the beginning of Triassic as the soft maximum age for the tree ferns.

27. *Lophosoria*

Hard minimum age constraint: 112 Ma. *Lophosoria cupulata* from the Aptian of Antarctica is placed in the extant genus[57] and was used here to constrain the split between *Lophosoria* and *Dicksonia*.

Soft maximum age constraint: 251 Ma. We apply the beginning of Triassic as the soft maximum age for the tree ferns.

28. Cyatheaceae

Hard minimum age constraint: 125 Ma. Permineralized fertiled structures have diagnostic characters of modern *Cyathea*, including abaxially borne sori with globose indusia, annulate sporangia with multiseriate stalks and vertical annuli that are not interrupted by the stalk, and 64 trilete spores[58]. The Barremian *Cyathea cranhamii* represents the oldest unequivocal member of modern Cyatheaceae[58] and is used here as the minimum age constraint, although the permineralized stem genus *Cyathocaulis* appears to be slightly older (Hauterivian)[59] and was resolved as a member of Cyatheaceae in a cladistic analysis[60]. We assigned the constraint to the Cyatheaceae stem lineage.

Soft maximum age constraint: 251 Ma. We apply the beginning of Triassic as the soft maximum age for the tree ferns.

29. Lindsaeaceae

Hard minimum age constraint: 99.6 Ma. Cretaceous fossil roots display sclerenchymatous outer cortex, parenchymatous inner cortex, and an innermost layer of six large cells[61]. This root anatomy is only known in the family Lindsaeaceae[61]. We assigned the constraint to the Lindsaeaceae stem lineage.

Soft maximum age constraint: 201.6 Ma. Some Jurassic fossils have been associated with Lindsaeaceae, although their affinity is highly uncertain[61,62]. Oldest fossils possibly associated with Polypodiales are also Jurassic[17,56,62] and we used the beginning of the Jurassic as the soft maximum age for the stem lineage.

30. Pteridaceae

Hard minimum age constraint: 93.5 Ma. The shape of the pinnae and the presence of pseudoindusia in a Cenomanian fossil[63] closely resemble pteridoid ferns, and this fossil has been used to constrain the origin of pteridoid lineage[16,17,31]. We assigned the constraint to the Pteridaceae stem lineage.

Soft maximum age constraint: 201.6 Ma. Oldest known putative members of Polypodiales are Jurassic[17,56,62] and therefore, we used the beginning of Jurassic as the soft maximum age constrain for the stem lineage.

31. *Acrostichum-Ceratopteris* –clade

Hard minimum age constraint: 65.5 Ma. Macrofossils from Maastrichtian were assigned to *Acrostichum*[64], but more conservative views consider this fossil as putative stem group member[17,31]. This more conservative approach is also followed here.

Soft maximum age constraint: 201.6 Ma. The soft maximum age of Pteridaceae was applied to the stem lineage.

32. *Ceratopteris*

Hard minimum age constraint: 37.2 Ma. Diagnostic *Ceratopteris* spores are known from the Bartonian[47]. This calibration has also been used in previous studies[17,31]. We assigned the constraint to the *Ceratopteris* stem lineage.

Soft maximum age constraint: 145.5 Ma. If the Late Cretaceous *Acrostichum*[64] indeed represents crown group *Acrostichum* instead of stem group, the divergence between *Acrostichum* and *Ceratopteris* must have occured earlier during the Cretaceous if not before. We apply the beginning of Cretaceous as the soft maximum age for the stem lineage.

33. *Dennstaedtia-Microlepia* –clade

Hard minimum age constraint: 70.6 Ma. The rhizome and stipe anatomy of the genus *Microlepiopsis* from Campanian are characteristic for the clade containing living species of *Microlepia* and *Dennstaedtia*[65]. This calibration was also applied in a previous study[31]. We assigned the constraint to the stem lineage of this clade.

Soft maximum age constraint: 201.6 Ma. It remains uncertain whether Dennstaedtiaceae or Pteridaceae diverged earlier. For this early divergence within Dennstaedtiaceae we apply the same soft maximum as for the family Pteridaceae.

34. *Pteridium*

Hard minimum age constraint: 23 Ma. Fossil evidence indicates worldwide distribution of *Pteridium* by Oligocene[66]. This constraint was assigned to the *Pteridium* stem lineage.

Soft maximum age constraint: 145.5 Ma. We apply the beginning of Cretaceous as the soft maximum age for the stem lineage in lack of good fossil record, but it has been estimated that *Pteridium* likely originated during Cretaceous-Paleogene[67].

35. *Drynaria*

Hard minimum age constraint: 33.9 Ma. Phylogenetic analysis resolved *Protodrynaria takhtajanii* from the Eocene-Oligocene boundary as a member of *Drynaria*[68]. The constraint was assigned to the *Drynaria* stem lineage.

Soft maximum age constraint: 145.5 Ma. Lower Cretaceous deposits of North America and China have yielded fossils with eupolypod characteristics[69–71]. Molecular studies have also suggested Cretaceous origin for the main eupolypod groups[16,17,31]. We apply the beginning of Cretaceous as the soft maximum age for the eupolypod groups.

36. *Drynaria mollis*

Hard minimum age constraint: 2.6 Ma. The morphology-based and total-evidence analyses resolved Pliocene *D. callispora* as sister to the extant species *D. mollis*, and these two formed sister lineage to *D. sinica*[68]. Although the morphological results somewhat differed from the molecular results (e.g. *Drynaria* monophyletic vs. paraphyletic), the nodes critical to place *D. callispora* in the phylogeny remained the same and we constrained the *D. mollis–D. sinica* split to have occured before 2.6 Ma.

Soft maximum age constraint: 65.5 Ma. We applied the beginning of Paleogene as the soft maximum age for this phylogenetically recent split.

37. Cyclosoroids

Hard minimum age constraint: 33.9 Ma. Eocene fossils can be assigned as members of cyclosoroids[72], which form a clade within Thelypteridaceae[73]. This calibration has also been used in previous studies[17,31]. We assigned the constraint to the cyclosoroid stem lineage.

Soft maximum age constraint: 145.5 Ma. We apply the beginning of Cretaceous as the soft maximum age for the eupolypod groups (see above).

38. *Onoclea sensibilis*

Hard minimum age constraint: 55.8 Ma. A large number of relatively complete Paleocene fossils conform to the living species in all recognizable features[74]. This calibration has been accepted in previous studies[17,31], although in these more sparsely sampled analyses the calibration was assigned at deeper phylogenetic level. We assigned the constraint to stem lineage of the clade of two *Onoclea sensibilis* varieties.

Soft maximum age constraint: 145.5 Ma. We apply the beginning of Cretaceous as the soft maximum age for the eupolypod groups (see above).

39. *Stenochlaena*

Hard minimum age constraint: 23 Ma. Distinctive spores of *Stenochlaenidites* are essentially identical with the spores of modern *Stenochlaena* and first appear in the late Oligocene[72]. The constraint was assigned to the *Stenochlaena* stem lineage.

Soft maximum age constraint: 145.5 Ma. We apply the beginning of Cretaceous as the soft maximum age for the eupolypod groups (see above).

40. *Woodwardia*

Hard minimum age constraint: 55.8 Ma. Several fossil *Woodwardia* species are known from the Paleocene onwards[72], including *W. bureiensis* from Wuyun Formation of China[75]. This calibration has been used previously as well[31]. The constraint was assigned to the *Woodwardia* stem lineage.

Soft maximum age constraint: 145.5 Ma. We apply the beginning of Cretaceous as the soft maximum age for the eupolypod groups (see above).

41. *Woodwardia virginica*

Hard minimum age constraint: 15.4 Ma. Combination of vegetative pinnules, rhizome and stipe anatomy, and fertile pinnules with sori and sporangia from the Middle Miocene were described to be

similar with the extant *Woodwardia virginica*[76]. The fossil bed was dated 15.6 ± 0.2 Ma by Ar/Ar dating technique[76,77]. We used the lower estimate as the hard minimum age to constrain the crown group *Woodwardia*.

Soft maximum age constraint: 145.5 Ma. We apply the soft maximum age of the genus as the soft maximum age for this early diverging species.

42. Athyriaceae

Hard minimum age constraint: 37.2 Ma. The Middle Eocene *Makopteris princetonensis* is the oldest fossil assigned to the family[78] and has been used to calibrate Athyriaceae in other studies as well[17,31]. The constraint was assigned to the Athyriaceae stem lineage.

Soft maximum age constraint: 145.5 Ma. We apply the beginning of Cretaceous as the soft maximum age for the eupolypod groups (see above).

Coding of fern life-form

Taxa	non-epiphytic (0) / epiphytic (1)
Abrodictyum caudatum	1
Abrodictyum elongatum	0
Abrodictyum rigidum	0
Acrostichum aureum	0
Acrostichum danaeifolium	0
Actiniopteris dimorpha	0
Actinostachys pennula	0
Acystopteris japonica	0
Acystopteris taiwaniana	0

Adenophorus abietinus	1
Adenophorus hymenophylloides	1
Adenophorus montanus	1
Adenophorus oahuensis	1
Adenophorus periens	1
Adenophorus pinnatifidus	1
Adenophorus tamariscinus	1
Adenophorus tenellus	1
Adenophorus tripinnatifidus	1
Adiantopsis radiata	0
Adiantum adiantoides	0
Adiantum aethiopicum	0
Adiantum argutum	0
Adiantum capillus-veneris	0
Adiantum diaphanum	0
Adiantum formosum	0
Adiantum hispidulum	0
Adiantum malesianum	0
Adiantum pedatum	0
Adiantum peruvianum	0
Adiantum raddianum	0
Adiantum tenerum	0
Adiantum tetraphyllum	0

Adiantum viridescens	0
Alansmia cultrata	1
Alansmia glandulifera	1
Alansmia lanigera	1
Alansmia senilis	1
Allosorus argenteus	0
Allosorus farinosus	0
Allosorus nitidulus	0
Alsophila bryophila	0
Alsophila cuspidata	0
Alsophila dregei	0
Alsophila foersteri	0
Alsophila hooglandii	0
Alsophila stelligera	0
Anemia adiantifolia	0
Anemia mexicana	0
Anemia phyllitidis	0
Anemia rotundifolia	0
Anemia tomentosa	0
Angiopteris evecta	0
Angiopteris lygodiifolia	0
Anogramma guatemalensis	0
Anogramma leptophylla	0

Antrophym latifolium	1
Arachniodes amoena	0
Arachniodes aristata	0
Arachniodes blinii	0
Arachniodes caudata	0
Arachniodes cavaleri	0
Arachniodes centrochinensis	0
Arachniodes chinensis	0
Arachniodes denticulata	0
Arachniodes festina	0
Arachniodes miqueliana	0
Arachniodes quadripinnata	0
Arachniodes rhomboides	0
Arachniodes simplicior	0
Arachniodes speciosa	0
Arachniodes standishii	0
Arachniodes superba	0
Argyrochosma fendleri	0
Argyrochosma incana	0
Argyrochosma jonesii	0
Argyrochosma limitanea	0
Arthromeris lehmannii	1
Arthromeris wallichiana	1

Arthropteris palisotii	0
Arthropteris parallela	0
Arthropteris paucivenia	1
Ascogrammitis dilatata	1
Ascogrammitis anfractuosa	1
Ascogrammitis david-smithii	1
Ascogrammitis nana	1
Ascogrammitis pichinchae	1
Ascogrammitis pichinchensis	1
Ascogrammitis sp.1	1
Ascogrammitis sp.2	1
Aspidotis californica	0
Aspidotis densa	0
Asplenium abscissum	0
Asplenium adiantum-nigrum	0
Asplenium affine	1
Asplenium alatum	0
Asplenium angustum	1
Asplenium anisophyllum	1
Asplenium aureum	0
Asplenium auritum	1
Asplenium australasicum	1
Asplenium bulbiferum	1

Asplenium bullatum	0
Asplenium contiguum	1
Asplenium cordatum	0
Asplenium cristatum	1
Asplenium cuneatiforme	1
Asplenium cuneifolium	0
Asplenium cuspidatum	1
Asplenium dielmannii	0
Asplenium difforme	0
Asplenium elliottii	1
Asplenium erectum	0
Asplenium feei	1
Asplenium fissum	0
Asplenium flabellifolium	1
Asplenium fontanum	0
Asplenium forisiense	0
Asplenium formosae	0
Asplenium formosum	0
Asplenium fragile	0
Asplenium gemmiferum	0
Asplenium hallbergii	0
Asplenium harpeodes	1
Asplenium hemionitis	0

Asplenium heterochroum	0
Asplenium hispanicum	0
Asplenium hobdyi	0
Asplenium hookerianum	0
Asplenium incisum	0
Asplenium jahandiezii	0
Asplenium juglandifolium	1
Asplenium laciniatum	0
Asplenium lamprophyllum	0
Asplenium lucidum	0
Asplenium lunulatum	0
Asplenium marinum	0
Asplenium monanthes	1
Asplenium myriophyllum	0
Asplenium nidus	1
Asplenium normale	1
Asplenium obtusatum	0
Asplenium planicaule	0
Asplenium platyneuron	0
Asplenium polyodon	1
Asplenium praemorsum	1
Asplenium prolongatum	0
Asplenium protensum	0

Asplenium pteropus	1
Asplenium resiliens	0
Asplenium rhizophyllum	0
Asplenium rigidum	0
Asplenium ritoense	0
Asplenium ruta-muraria	0
Asplenium sandersonii	1
Asplenium scolopendrium	0
Asplenium shuttleworthianum	0
Asplenium simplicifrons	1
Asplenium surrogatum	1
Asplenium tenerum	1
Asplenium theciferum	1
Asplenium tricholepis	1
Asplenium trichomanes	0
Asplenium varians	0
Asplenium vieillardii	0
Asplenium viride	0
Asplenium wrightii	0
Asplenium yunnanense	0
Athyrium decurrentialatum	0
Athyrium filix-femina	0
Athyrium nipponicum	0

Athyrium otophorum	0
Athyrium yokoscense	0
Azolla filiculoides	0
Azolla nilotica	0
Azolla pinnata	0
Azolla rubra	0
Blechnum brasiliense	0
Blechnum cyatheoides	0
Blechnum insigne	0
Blechnum medium	0
Blechnum neglectum	0
Blechnum nom.nov.	0
Blechnum occidentale	0
Blechnum orientale	0
Blechnum polypodioides	0
Blechnum schomburgkii	0
Blechnum spicant	0
Blotiella pubescens	0
Bolbitis appendiculata	0
Bolbitis auriculata	0
Bolbitis heteroclita	0
Bommeria ehrenbergiana	0
Bommeria hispida	0

Botrychium dissectum	0
Botrychium japonicum	0
Botrychium lanceolatum	0
Botrychium lunaria	0
Botrychium lunarioides	0
Botrychium simplex	0
Botrychium strictum	0
Botrychium virginianum	0
Calciphilopteris ludens	0
Callistopteris apiifolia	0
Calochlaena straminea	0
Calochlaena villosa	0
Calymmodon gracilis	1
Calymmodon luerssenianus	1
Campyloneurum angustifolium	1
Campyloneurum aphanophlebium	1
Campyloneurum asplundii	1
Campyloneurum brevifolium	1
Campyloneurum chlorolepis	1
Campyloneurum latum	1
Campyloneurum xalapense	1
Cephalomanes javanicum	0
Ceradenia fucoides	1

Ceradenia jungermannioides	1
Ceradenia kalbreyeri	1
Ceradenia pilipes	1
Ceradenia spixiana	1
Ceratopteris richardii	0
Ceratopteris thalictroides	0
Cheilanthes distans	0
Cheilanthes lasiophylla	0
Cheilanthes sieberi	0
Cheiloplecton rigidum	0
Cheiropleuria integrifolia	0
Christensenia aesculifola	0
Chrysogrammitis musgraviana	1
Cibotium barometz	0
Cibotium glaucum	0
Cibotium schiedei	0
Cochlidium punctatum	1
Cochlidium rostratum	1
Cochlidium seminudum	1
Cochlidium serrulatum	1
Coniogramme fraxinea	0
Coniogramme japonica	0
Crepidomanes bipunctatum	1

Crepidomanes minutum	1
Crepidomanes thysanostomum	0
Cryptogramma crispa	0
Ctenitis decurrentipinnata	0
Ctenitis eatonii	0
Ctenitis refulgens	0
Ctenitis rhodolepis	0
Ctenitis sloanei	0
Ctenitis submarginalis	0
Ctenopteris lasiostipes	1
Ctenopteris nutans	1
Ctenopteris repandula	1
Culcita coniifolia	0
Culcita macrocarpa	0
Cyathea alata	0
Cyathea arborea	0
Cyathea dejecta	0
Cyathea horrida	0
Cyathea howeana	0
Cyathea multiflora	0
Cyathea parvula	0
Cyathea poeppigii	0
Cyathea senilis	0

Cyclopeltis crenata	0
Cyclopeltis semicordata	0
Cyrtomium falcatum	0
Cyrtomium fortunei	0
Cyrtomium macrophyllum	0
Cyrtomium nephrolepioides	0
Cyrtomium urophyllum	0
Cystodium sorbifolium	0
Cystopteris fragilis	0
Danaea alata	0
Danaea falcata	0
Danaea nodosa	0
Danaea simplicifolia	0
Danaea sp.	0
Danaea urbanii	0
Danaea wendlandii	0
Davallia burbidgei	1
Davallia clarkei	1
Davallia corniculata	1
Davallia divaricata	1
Davallia epiphylla	1
Davallia fejeensis	1
Davallia griffithiana	1

Davallia hirsuta	1
Davallia hymenophylloides	1
Davallia mariesii	1
Davallia melanophlebia	1
Davallia parvula	1
Davallia perdurans	1
Davallia plumosa	1
Davallia polypodioides	1
Davallia pulchra	1
Davallia pyxidata	1
Davallia repens	1
Davallia solida	1
Davallia tasmanii	0
Davallia trichomanoides	1
Davallia triphylla	1
Davallia yunnanensis	1
Dennstaedtia punctilobula	0
Dennstaedtia scabra	0
Deparia bonincola	0
Deparia lancea	0
Deparia petersenii	0
Deparia unifurcata	0
Dicksonia antarctica	0

Dicksonia thyrsopteroides	0
Dicranopteris linearis	0
Dictymia brownii	1
Dictymia mckeei	1
Didymochlaena truncatula	0
Didymoglossum cuspidatum	1
Didymoglossum ekmanii	1
Didymoglossum hildebrandtii	1
Didymoglossum krausii	1
Didymoglossum membranaceum	1
Diplaziopsis javanica	0
Diplazium bombonasae	0
Diplazium centripetale	0
Diplazium cristatum	0
Diplazium dilatatum	0
Diplazium hachijoense	0
Diplazium legalloi	0
Diplazium okudairai	0
Diplazium plantaginifolium	0
Diplazium proliferum	0
Diplazium virescens	0
Diplazium wichurae	0
Diplopterygium bancroftii	0

Diplopterygium glaucum	0
Diplopterygium laevissimum	0
Dipteris conjugata	0
Doryopteris gleichenioides	0
Doryopteris nobilis	0
Doryopteris rediviva	0
Doryopteris sagittifolia	0
Doryopteris viridis	0
Dracoglossum plantagineum	0
Drynaria acuminata	1
Drynaria bonii	1
Drynaria coronans	1
Drynaria drynarioides	1
Drynaria laurentii	1
Drynaria meyeniana	1
Drynaria mollis	1
Drynaria nom.nov.	1
Drynaria novoguineensis	1
Drynaria pilosa	1
Drynaria rigidula	1
Drynaria sagitta	1
Drynaria sinica	1
Drynaria sp.	1

Drynaria sparsisora	1
Drynaria splendens	1
Drynaria volkensii	1
Dryopteris aemula	0
Dryopteris annamensis	0
Dryopteris bissetiana	0
Dryopteris crassirhizoma	0
Dryopteris diffracta	0
Dryopteris erythrosora	0
Dryopteris expansa	0
Dryopteris filix-mas	0
Dryopteris goldiana	0
Dryopteris hasseltii	0
Dryopteris hendersonii	0
Dryopteris heterolaena	0
Dryopteris hookeriana	0
Dryopteris labordei	0
Dryopteris marginalis	0
Dryopteris mariformis	0
Dryopteris maximowiczii	0
Dryopteris neolacera	0
Dryopteris nodosa	0
Dryopteris pacifica	0

Dryopteris sacrosancta	0
Dryopteris sieboldii	0
Dryopteris sparsa	0
Dryopteris squamiseta	0
Dryopteris uniformis	0
Dryopteris wallichiana	0
Elaphoglossum amygdalifolium	0
Elaphoglossum andicola	1
Elaphoglossum aubertii	0
Elaphoglossum burchellii	0
Elaphoglossum callifolium	0
Elaphoglossum crassifolium	1
Elaphoglossum crinitum	1
Elaphoglossum deltoides	0
Elaphoglossum erinaceum	1
Elaphoglossum flaccidum	1
Elaphoglossum glabellum	1
Elaphoglossum heterolepis	1
Elaphoglossum hybridum	0
Elaphoglossum lechlerianum	1
Elaphoglossum lingua	1
Elaphoglossum lonchophyllum	1
Elaphoglossum minutum	0

Elaphoglossum paleaceum	1
Elaphoglossum papillosum	1
Elaphoglossum peltatum	1
Elaphoglossum piloselloides	1
Elaphoglossum samoense	1
Elaphoglossum tripartitum	0
Enterosora parietina	1
Enterosora percrassa	1
Enterosora trifurcata	1
Equisetum arvense	0
Equisetum bogotense	0
Equisetum diffusum	0
Equisetum ferrissii	0
Equisetum giganteum	0
Equisetum myriochaetum	0
Equisetum pratense	0
Equisetum ramosissimum	0
Equisetum scirpoides	0
Equisetum sylvaticum	0
Equisetum telmateia	0
Equisetum variegatum	0
Eupodium laeve	0
Gaga arizonica	0

Galactodenia subscabra	1
Gleichenella pectinata	0
Gleichenia dicarpa	0
Goniophlebium amoenum	1
Goniophlebium argutum	1
Goniophlebium chinense	1
Goniophlebium formosanum	1
Goniophlebium manmeiense	1
Goniophlebium mehibitense	1
Goniophlebium microrhizoma	1
Goniophlebium percussum	1
Goniophlebium persicifolium	1
Goniophlebium pseudoconnatum	1
Goniophlebium subauriculatum	1
Grammitis bryophila	1
Grammitis deplanchei	0
Grammitis kyimbilensis	1
Grammitis melanoloma	1
Gymnocarpium dryopteris	0
Gymnocarpium oyamense	0
Gymnocarpium remotepinnatum	0
Gymnosphaera capensis	0
Gymnosphaera klossii	0

Gymnosphaera podophylla	0
Gymnosphaera ramispina	0
Gymnosphaera salvinii	0
Haplopteris elongata	1
Haplopteris graminea	1
Haplopteris taeniophylla	1
Haplopteris volkensii	1
Hecistopteris pumila	1
Helminthostachys zeylanica	0
Hemidictyum marginatum	0
Hemionitis arifolia	0
Hemionitis palmata	0
Histiopteris incisa	0
Homalosorus pycnocarpos	0
Hymenasplenium cheilosorum	0
Hymenasplenium excisum	1
Hymenasplenium laetum	0
Hymenasplenium unilaterale	1
Hymenophyllum acanthoides	1
Hymenophyllum apiculatum	1
Hymenophyllum armstrongii	1
Hymenophyllum atrovirens	0
Hymenophyllum badium	1

Hymenophyllum baileyanum	1
Hymenophyllum barbatum	1
Hymenophyllum bivalve	1
Hymenophyllum caespitosum	1
Hymenophyllum caudiculatum	1
Hymenophyllum cruentum	1
Hymenophyllum cupressiforme	1
Hymenophyllum demissum	1
Hymenophyllum dentatum	1
Hymenophyllum deplanchei	1
Hymenophyllum dicranotrichum	1
Hymenophyllum digitatum	1
Hymenophyllum dilatatum	1
Hymenophyllum exsertum	1
Hymenophyllum flexuosum	1
Hymenophyllum frankliniae	1
Hymenophyllum fuciforme	1
Hymenophyllum fucoides	1
Hymenophyllum heimii	1
Hymenophyllum hirsutum	1
Hymenophyllum hygrometricum	1
Hymenophyllum imbricatum	1
Hymenophyllum inaequale	1

Hymenophyllum javanicum	1
Hymenophyllum kuhnii	1
Hymenophyllum lanceolatum	1
Hymenophyllum leratii	0
Hymenophyllum lyallii	1
Hymenophyllum malingii	1
Hymenophyllum marginatum	1
Hymenophyllum minimum	1
Hymenophyllum mnioides	1
Hymenophyllum multifidum	0
Hymenophyllum pallidum	0
Hymenophyllum paniense	1
Hymenophyllum pectinatum	1
Hymenophyllum peltatum	1
Hymenophyllum perrieri	1
Hymenophyllum plicatum	1
Hymenophyllum polyanthos	1
Hymenophyllum poolii	1
Hymenophyllum pulcherrimum	1
Hymenophyllum rolandi-principis	1
Hymenophyllum rufescens	1
Hymenophyllum rugosum	1
Hymenophyllum scabrum	1

Hymenophyllum secundum	1
Hymenophyllum serrulatum	1
Hymenophyllum sibthorpioides	1
Hymenophyllum sp.	1
Hymenophyllum subdimidiatum	1
Hymenophyllum tenellum	1
Hymenophyllum tunbrigense	1
Hymenophyllum undulatum	0
Hymenophyllum villosum	1
Hymenophyllum wrightii	1
Hypodematium crenatum	0
Hypolepis tenuifolia	0
Jamesonia canescens	0
Jamesonia cheilanthoides	0
Jamesonia flexuosa	0
Jamesonia verticalis	0
Lastreopsis hispida	0
Lastreopsis subrecedens	0
Lellingeria apiculata	1
Lellingeria brevistipes	1
Lellingeria depressa	1
Lellingeria dissimulans	1
Lellingeria flagellipinnata	1

Lellingeria hirsuta	1
Lellingeria isidrensis	1
Lellingeria itatimensis	0
Lellingeria kaieteura	1
Lellingeria melanotrichia	1
Lellingeria phlegmaria	1
Lellingeria pseudocapillaris	1
Lellingeria randallii	1
Lellingeria simacensis	1
Lellingeria sp.1	1
Lellingeria sp.2	1
Lellingeria subsessilis	1
Lellingeria suspensa	1
Lellingeria tenuicula	1
Lemmaphyllum carnosum	1
Lemmaphyllum microphyllum	1
Lemmaphyllum rostratum	1
Lemmaphyllum squamatum	0
Lepidomicrosorium buergerianum	0
Lepidomicrosorium subhemionitideum	1
Lepidomicrosorium superficiale	1
Lepisorus accedens	1
Lepisorus annamensis	1

Lepisorus annuifrons	1
Lepisorus boninensis	1
Lepisorus excavatus	1
Lepisorus henryi	1
Lepisorus kawakamii	1
Lepisorus lewisii	0
Lepisorus marginatus	1
Lepisorus miyoshianus	1
Lepisorus monilisorus	1
Lepisorus mucronatus	1
Lepisorus nudus	1
Lepisorus platyrhynchos	1
Lepisorus pseudonudus	1
Lepisorus pseudoussuriensis	1
Lepisorus scolopendrius	1
Lepisorus sinensis	1
Lepisorus spicatus	1
Lepisorus thaipaiensis	1
Lepisorus thunbergianus	1
Lepisorus uchiyamae	0
Leptochilus cantoniensis	0
Leptochilus decurrens	1
Leptochilus digitatus	0

Leptochilus ellipticus	0
Leptochilus hemionitideus	0
Leptochilus henryi	0
Leptochilus simplicifrons	0
Leptochilus triphyllus	0
Leptochilus wrightii	1
Leptochilus xhemitomus	0
Leptolepia nom.nov.	0
Leptolepia novae-zelandiae	0
Leptopteris superba	0
Leptopteris wilkesiana	0
Leucostegia immersa	1
Leucostegia pallida	1
Leucotrichium mitchelliae	1
Leucotrichium mortonii	1
Leucotrichium organense	1
Leucotrichium pseudomitchelliae	1
Leucotrichium schenckii	1
Lindsaea blotiana	0
Lindsaea botrychioides	0
Lindsaea ensifolia	0
Lindsaea imrayana	0
Lindsaea multisora	0

Lindsaea parasitica	1
Lindsaea plicata	0
Lindsaea quadrangularis	0
Lindsaea rufa	0
Lindsaea stricta	0
Llavea cordifolia	0
Lomagramma matthewii	0
Lomariopsis japurensis	0
Lomariopsis pollicina	0
Lomariopsis sorbifolia	0
Lomariopsis sp.	0
Lomariopsis spectabilis	0
Lonchitis hirsuta	0
Lophosoria quadripinnata	0
Loxogramme abyssinica	1
Loxogramme avenia	1
Loxogramme dictyopteris	1
Loxogramme grammitoides	1
Loxogramme salicifolia	1
Loxsoma cunninghamii	0
Loxsomopsis pearcei	0
Lygodium japonicum	0
Lygodium lanceolatum	0

Lygodium radiatum	0
Lygodium reticulatum	0
Macrothelypteris torresiana	0
Marattia alata	0
Marsilea crenata	0
Marsilea crotophora	0
Marsilea drummondii	0
Marsilea gibba	0
Marsilea mutica	0
Marsilea nubica	0
Marsilea vera	0
Matonia pectinata	0
Matteuccia struthiopteris	0
Maxonia apiifolia	0
Megalastrum subincisum	0
Melpomene allosuroides	1
Melpomene gracilis	1
Melpomene melanosticta	1
Melpomene moniliformis	0
Metaxya rostrata	0
Mickelia guianensis	0
Mickelia nicotianifolia	0
Microgramma baldwinii	1

Microgramma bifrons	1
Microgramma fuscopunctata	1
Microgramma lycopodioides	1
Microgramma mauritiana	1
Microgramma megalophylla	1
Microgramma nitida	1
Microgramma percussa	1
Microgramma sp.	1
Microgramma squamulosa	1
Microgramma tecta	1
Microgramma vacciniifolia	1
Microlepia platyphylla	0
Microlepia speluncae	0
Microlepia szechuanica	0
Microsorum carnosum	1
Microsorum commutatum	0
Microsorum crustaceum	1
Microsorum cuspidatum	1
Microsorum grossum	0
Microsorum hainanense	1
Microsorum insigne	0
Microsorum lastii	1
Microsorum linguiforme	1

Microsorum luzonense	1
Microsorum membranaceum	1
Microsorum mirabile	1
Microsorum musifolium	1
Microsorum novae-zealandiae	1
Microsorum papuanum	0
Microsorum pteropus	0
Microsorum punctatum	1
Microsorum pustulatum	1
Microsorum sarcopus	1
Microsorum scandens	1
Microsorum sinuosum	1
Microsorum spectrum	1
Microsorum thailandicum	0
Microsorum varians	1
Microsorum vieillardii	1
Microsorum whiteheadii	0
Mildella intramarginalis	0
Monachosorum henryi	0
Moranopteris achilleifolia	1
Moranopteris hyalina	1
Moranopteris longisetosa	1
Moranopteris taenifolia	1

Moranopteris zurquina	1
Mycopteris alsopteris	1
Mycopteris semihirsuta	1
Mycopteris sp.1	1
Mycopteris sp.2	1
Mycopteris subtilis	1
Mycopteris taxifolia	1
Myriopteris alabamensis	0
Myriopteris aurea	0
Myriopteris covillei	0
Myriopteris lanosa	0
Myriopteris lendigera	0
Myriopteris myriophylla	0
Myriopteris newberryi	0
Myriopteris pringlei	0
Myriopteris rufa	0
Myriopteris wrightii	0
Neocheiropteris palmatopedata	1
Neolepisorus ensatus	1
Neolepisorus fortunei	1
Neolepisorus phyllomanes	0
Neolepisorus zippelii	1
Nephrolepis abrupta	0

Nephrolepis cordifolia	0
Nephrolepis davalliae	1
Nephrolepis davallioides	1
Nephrolepis exaltata	1
Nephrolepis falcata	1
Nephrolepis hirsutula	0
Nephrolepis lauterbachii	1
Nephrolepis rivularis	1
Nephrolepis undulata	0
Nesolindsaea kirkii	0
Niphidium crassifolium	1
Notholaena aschenborniana	0
Notholaena aurantiaca	0
Notholaena aurea	0
Notholaena brachypus	0
Notholaena bryopoda	0
Notholaena californica	0
Notholaena candida	0
Notholaena grayi	0
Notholaena greggii	0
Notholaena lemmonii	0
Notholaena leucopoda	0
Notholaena neglecta	0

Notholaena rigida	0
Notholaena rosei	0
Notholaena schaffneri	0
Notholaena standleyi	0
Notholaena sulphurea	0
Notholaena trichomanoides	0
Notogrammitis billardierei	1
Notogrammitis ciliata	0
Notogrammitis crassior	0
Notogrammitis heterophylla	1
Notogrammitis pseudociliata	1
Odontosoria aculeata	0
Odontosoria biflora	0
Odontosoria chinensis	0
Oleandra articulata	1
Oleandra pistillaris	1
Onoclea sensibilis	0
Onoclea sensibilis var. *interrupta*	0
Onocleopsis hintonii	0
Onychium japonicum	0
Ophioglossum pendulum	1
Ophioglossum reticulatum	0
Oreogrammitis congener	1

Oreogrammitis dolichosora	0
Oreogrammitis forbesiana	1
Oreogrammitis hookeri	1
Oreogrammitis knutsfordiana	1
Oreogrammitis padangensis	1
Oreogrammitis reinwardtioides	1
Osmolindsaea japonica	0
Osmolindsaea odorata	0
Osmunda banksiifolia	0
Osmunda claytoniana	0
Osmunda javanica	0
Osmunda regalis	0
Osmundastrum cinnamomeum	0
Osmundastrum cinnamomeum var. *fokiense*	0
Paesia scaberula	0
Paragramma longifolia	1
Parapolystichum glabellum	0
Parapolystichum glabellumeffusum	0
Pecluma alfredii	1
Pecluma chnoophora	1
Pecluma dulcis	1
Pecluma eurybasis	1
Pecluma hartwegiana	1

Pecluma longepinnulata	1
Pecluma longepinnulata	1
Pecluma ptilodon	1
Pecluma rhachipterygia	0
Pellaea andromedifolia	0
Pellaea atropurpurea	0
Pellaea bipinnata	0
Pellaea breweri	0
Pellaea cochisensis	0
Pellaea intermedia	0
Pellaea marantae	0
Pellaea rotundifolia	0
Pellaea sargentii	0
Pellaea sinuata	0
Pellaea truncata	0
Pellaea vestita	0
Pentagramma triangularis	0
Phanerophlebia nobilis	0
Phanerosorus sarmentosus	0
Phegopteris connectilis	0
Phegopteris decursivepinnata	0
Phegopteris hexagonoptera	0
Phlebodium areolatum	1

Phlebodium decumanum	1
Phlebodium pseudoaureum	1
Pilularia globulifera	0
Pilularia novae-hollandiae	0
Pityrogramma austroamericana	0
Pityrogramma calomelanos	0
Pityrogramma chaerophylla	0
Plagiogyria euphlebia	0
Plagiogyria japonica	0
Plagiogyria matsumureana	0
Plagiogyria semicordata	0
Platycerium alcicorne	1
Platycerium andinum	1
Platycerium bifurcatum	1
Platycerium coronarium	1
Platycerium elephantotis	1
Platycerium ellisii	1
Platycerium grande	1
Platycerium hillii	1
Platycerium holttumii	1
Platycerium madagascariense	1
Platycerium quadridichotomum	1
Platycerium ridleyi	1

Platycerium stemaria	1
Platycerium veitchii	0
Platycerium wallichii	1
Platycerium wandae	1
Platycerium willinckii	1
Pleocnemia rufinervis	0
Pleopeltis angusta	1
Pleopeltis astrolepis	1
Pleopeltis bradeorum	1
Pleopeltis buchtienii	1
Pleopeltis christensenii	1
Pleopeltis crassinervata	1
Pleopeltis desvauxii	1
Pleopeltis disjuncta	1
Pleopeltis ensiformis	1
Pleopeltis fraseri	1
Pleopeltis friedrichsthalianum	1
Pleopeltis fructuosa	1
Pleopeltis furfuraceum	1
Pleopeltis intermedia	1
Pleopeltis macrocarpa	1
Pleopeltis marginata	1
Pleopeltis monosora	1

Pleopeltis muenchii	1
Pleopeltis myriolepis	1
Pleopeltis platylepis	1
Pleopeltis pleopeltifolia	1
Pleopeltis polypodioides	1
Pleopeltis pycnocarpa	1
Pleopeltis rosei	1
Pleopeltis rzedowskianum	1
Pleopeltis sanctae-rosae	1
Pleopeltis thyssanolepis	0
Pleopeltis wiesbaurii	1
Pleurosoriopsis makinoi	1
Polybotrya alfredii	0
Polybotrya caudata	0
Polybotrya cervina	0
Polybotrya guianensis	0
Polybotrya sessilisora	0
Polybotrya trianae	0
Polyphlebium angustatum	1
Polyphlebium borbonicum	1
Polyphlebium endlicherianum	1
Polypodium adelphum	1
Polypodium arcanum	1

Polypodium chrysolepis	1
Polypodium glycyrrhiza	0
Polypodium hesperium	0
Polypodium macaronesicum	0
Polypodium martensii	1
Polypodium pellucidum	0
Polypodium plesiosorum	1
Polypodium rhodopleuron	1
Polypodium scouleri	0
Polypodium subpetiolatum	1
Polypodium vulgare	1
Polystichopsis chaerophylloides	0
Polystichum acutidens	0
Polystichum caducum	0
Polystichum capillipes	0
Polystichum conjunctum	0
Polystichum craspedosorum	0
Polystichum eximium	0
Polystichum fraxinellum	0
Polystichum glaciale	0
Polystichum hillebrandii	0
Polystichum hookerianum	0
Polystichum lemmonii	0

Polystichum luctuosum	0
Polystichum munitum	0
Polystichum neolobatum	0
Polystichum omeiense	0
Polystichum setiferum	0
Polystichum tonkinense	0
Polystichum transkeiense	0
Polystichum tripteron	0
Polystichum yunnanense	0
Polytaenium cajenense	1
Prosaptia alata	1
Prosaptia contigua	1
Prosaptia obliquata	1
Prosaptia rhodocarpa	1
Pseudophegopteris aurita	0
Pseudophegopteris cruciata	0
Psilotum nudum	1
Pteridium aquilinum	0
Pteridium esculentum	0
Pteridrys lofouensis	0
Pteris arborea	0
Pteris comans	0
Pteris cretica	0

Pteris ensiformis	0
Pteris fauriei	0
Pteris multifida	0
Pteris pallens	0
Pteris platyzomopsis	0
Pteris praestantissima	0
Pteris propinqua	0
Pteris quadriaurita	0
Pteris tremula	0
Pteris vittata	0
Pterozonium brevifrons	0
Ptisana attenuata	0
Ptisana fraxinea	0
Ptisana oreades	0
Ptisana purpurascens	0
Ptisana salicina	0
Pyrrosia angustata	1
Pyrrosia assimilis	1
Pyrrosia christii	1
Pyrrosia foveolata	1
Pyrrosia linearifolia	1
Pyrrosia lingua	1
Pyrrosia longifolia	1

Pyrrosia niphoboloides	1
Pyrrosia nuda	1
Pyrrosia piloselloides	1
Pyrrosia polydactyla	1
Pyrrosia rasamalae	1
Pyrrosia rupestris	1
Pyrrosia serpens	1
Pyrrosia subfurfuracea	1
Radiogrammitis havilandii	1
Radiogrammitis hirtelloides	1
Radiogrammitis holttumii	1
Radiogrammitis jagoriana	1
Radiogrammitis parva	1
Radiovittaria gardneriana	1
Regnellidium diphyllum	0
Rhachidosorus pulcher	0
Rheopteris cheesmaniae	1
Rumohra adiantiformis	1
Saccoloma brasiliense	0
Saccoloma elegans	0
Saccoloma inaequale	0
Salpichlaena hookeriana	0
Salpichlaena thalassica	0

Salpichlaena volubilis	0
Salvinia cucullata	0
Salvinia minima	0
Salvinia molesta	0
Salvinia natans	0
Salvinia oblongifolia	0
Schizaea dichotoma	0
Schizaea sp.	0
Scleroglossum sulcata	0
Selliguea brachypodia	1
Selliguea dareiformis	1
Selliguea enervis	1
Selliguea hellwigii	1
Selliguea heterocarpa	1
Selliguea laciniata	1
Selliguea lanceolata	1
Selliguea plantaginea	0
Selliguea triloba	1
Serpocaulon adnatum	1
Serpocaulon attenuatum	1
Serpocaulon catharinae	1
Serpocaulon dissimile	1
Serpocaulon falcaria	1

Serpocaulon fraxinifolium	1
Serpocaulon funckii	1
Serpocaulon lasiopus	1
Serpocaulon loriciforme	1
Serpocaulon meniscifolium	1
Serpocaulon ptilorhizon	1
Serpocaulon sessilifolium	1
Serpocaulon silvulae	1
Serpocaulon subandinum	1
Serpocaulon triseriale	1
Serpocaulon wagneri	1
Serpocaulon wiesbaueri	1
Sphaeropteris capitata	0
Sphaeropteris celebica	0
Sphaeropteris horrida	0
Sphaeropteris medullaris	0
Sphaeropteris robusta	0
Sphenomeris clavata	0
Stenochlaena milnei	0
Stenochlaena tenuifolia	0
Stenogrammitis hartii	1
Stenogrammitis hildebrandtii	1
Stenogrammitis limula	1

Stenogrammitis myosuroides	1
Stenogrammitis oosora	1
Stenogrammitis prionodes	1
Stenogrammitis pumila	1
Stenogrammitis saffordii	1
Stenogrammitis subcoriacea	1
Stenogrammitis wittigiana	1
Sticherus laevigatus	0
Sticherus palmatus	0
Stigmatopteris lechleri	0
Stigmatopteris longicaudata	0
Stromatopteris moniliformis	0
Synammia feuillei	1
Synammia intermedia	1
Tapeinidium luzonicum	0
Tapeinidium moorei	0
Tapeinidium pinnatum	0
Tectaria apiifolia	0
Tectaria decurrens	0
Tectaria devexa	0
Tectaria fimbriata	0
Tectaria fuscipes	0
Tectaria harlandii	0

Tectaria incisa	0
Tectaria nayarii	0
Tectaria prolifera	0
Tectaria subtriphylla	0
Tectaria trifoliata	0
Tectaria variolosa	0
Tectaria zeilanica	0
Telmatoblechnum serrulatum	0
Teratophyllum wilkesianum	0
Terpsichore eggersii	1
Terpsichore hanekeana	1
Terpsichore lehmanniana	1
Thelypteris abrupta	0
Thelypteris affinis	0
Thelypteris ciliata	0
Thelypteris clypeolutata	0
Thelypteris consanguinea	0
Thelypteris dayi	0
Thelypteris dentata	0
Thelypteris ecallosa	0
Thelypteris erubescens	0
Thelypteris esquirolii	0
Thelypteris gemmulifera	0

Thelypteris glandulosa	0
Thelypteris griffithii	0
Thelypteris interrupta	0
Thelypteris limbosperma	0
Thelypteris longissima	0
Thelypteris noveboracensis	0
Thelypteris oligocarpa	0
Thelypteris opulenta	0
Thelypteris ovata	0
Thelypteris palustris	0
Thelypteris poiteana	0
Thelypteris pozoi	0
Thelypteris reticulata	0
Thelypteris seemannii	0
Thelypteris simplex	0
Thelypteris taiwanensis	0
Thelypteris uraiensis	0
Thelypteris xylodes	0
Themelium conjunctisorum	1
Thylacopteris papillosa	1
Thyrsopteris elegans	0
Tmesipteris tannensis	1
Todea barbara	0

Tricholepidium maculosum	1
Trichomanes alatum	0
Trichomanes ankersii	0
Trichomanes arbuscula	0
Trichomanes crispum	0
Trichomanes elegans	0
Trichomanes hostmannianum	0
Trichomanes lucens	1
Trichomanes osmundoides	0
Trichomanes pinnatum	0
Trichomanes scandens	0
Triplophyllum funestum	0
Tryonia myriophylla	0
Vandenboschia davallioides	0
Vandenboschia radicans	0
Vittaria graminifolia	1
Vittaria lineata	1
Woodsia ilvensis	0
Woodsia manchuriensis	0
Woodsia obtusa	0
Woodsia polystichoides	0
Woodwardia areolata	0
Woodwardia fimbriata	0

Woodwardia japonica	0
Woodwardia kempii	0
Woodwardia prolifera	0
Woodwardia unigemmata	0
Woodwardia virginica	0

References

1. Scotese, C. R. 3D paleogeographic and plate tectonic reconstructions: The PALEOMAP Project is back in town. *Houst. Geol. Soc. Bull.* **44,** 13–15 (2002).
2. Smith, W. H. F. & Sandwell, D. T. Global sea floor topography from satellite altimetry and ship depth soundings. *Science* **277,** 1956–1962 (1997).
3. Lythe, M. B. & Vaughan, D. G. BEDMAP–bed topography of the Antarctic. (British Antarctic Survey, Natural Environment Research Council, 2000).
4. Jakobsson, M., Macnab, R., Cherkis, N. & Schenke, H.-W. The international bathymetric chart of the Arctic ocean (IBCAO). (Research Publication RP-2, National Geophysical Data Center, 2004).
5. Scotese, C. R. PALEOMAP PaleoAtlas for GPlates and the PaleoData Plotter Program, PALEOMAP Project, http://www.earthbyte.org/paleomap-paleoatlas-for-gplates/. (2016).
6. Ziegler, A. M. A proposal to produce an atlas of paleogeographic maps. (Department of Geophysical Sciences, University of Chicago, 1975).
7. Ziegler, A. M. & Scotese, C. R. Thoughts on format for the forthcoming 'Atlas of Paleogeographic Maps'. (Department of Geophysical Sciences, University of Chicago, 1977).
8. Ziegler, A. M. *et al.* Paleogeographic interpretation: with an example from the Mid-Cretaceous.

Annu. Rev. Earth Planet. Sci. **13,** 385–425 (1985).

9. Rees, P. M. *et al.* Permian phytogeographic patterns and climate data/model comparisons. *J. Geol.* **110,** 1–31 (2002).

10. Rees, P. M., Ziegler, A. M. & Valdes, P. J. in *Warm Climates in Earth History* (eds. Huber, B. T., Macleod, K. G. & Wing, S. L.) 297–318 (Cambridge University Press, 2000).

11. Scotese, C. R. *Atlas of Plate Tectonic Reconstructions (Mollweide Projection), Volumes 1-6. https://www.academia.edu/9712803/.* (PALEOMAP Project, Evanston, IL., 2014).

12. Peltier, W. R. Global glacial isostasy and the surface of the ice-age Earth: The ICE-5G (VM2) Model and GRACE. *Annu. Rev. Earth Planet. Sci.* **32,** 111–149 (2004).

13. Stewart, W. N. & Rothwell, G. W. *Paleobotany and the evolution of plants*. (Cambridge University Press, 1993).

14. Taylor, E. L., Taylor, T. N. & Krings, M. *Paleobotany: the biology and evolution of fossil plants*. (Academic Press, 2009).

15. Rothwell, G. W. & Nixon, K. C. How does the inclusion of fossil data change our conclusions about the phylogenetic history of euphyllophytes? *Int. J. Plant Sci.* **167,** 737–749 (2006).

16. Pryer, K. M. *et al.* Phylogeny and evolution of ferns (monilophytes) with a focus on the early leptosporangiate divergences. *Am. J. Bot.* **91,** 1582–1598 (2004).

17. Schneider, H. *et al.* Ferns diversified in the shadow of angiosperms. *Nature* **428,** 553–557 (2004).

18. Fiz-Palacios, O., Schneider, H., Heinrichs, J. & Savolainen, V. Diversification of land plants: insights from a family-level phylogenetic analysis. *BMC Evol. Biol.* **11,** 1–10 (2011).

19. Kenrick, P. & Crane, P. R. The origin and early evolution of plants on land. *Nature* **389,** 33–39 (1997).

20. Carpenter, R. Early tertiary *Tmesipteris* (Psilotaceae) macrofossil from Tasmania. *Aust. Syst. Bot.* **1,** 171–176 (1988).

21. Lehtonen, S. Towards resolving the complete fern tree of life. *PLoS ONE* **6,** e24851 (2011).

22. Rothwell, G. W. & Stockey, R. A. Fossil Ophioglossales in the Paleocene of western North America. *Am. J. Bot.* **76,** 637–644 (1989).

23. Liu, Z., Hilton, J. & Li, C. Review on the origin, evolution and phylogeny of Marattiales. *Chin. Bull. Bot.* **17,** 39–52 (2000).

24. Murdock, A. G. A taxonomic revision of the eusporangiate fern family Marattiaceae, with description of a new genus *Ptisana*. *Taxon* **57,** 737–755 (2008).

25. Wang, Y. Fertile organs and in situ spores of *Marattia asiatica* (Kawasaki) Harris (Marattiales) from the Lower Jurassic Hsiangchi Formation in Hubei, China. *Rev. Palaeobot. Palynol.* **107,** 125–144 (1999).

26. Murdock, A. G. Phylogeny of marattioid ferns (Marattiaceae): inferring a root in the absence of a closely related outgroup. *Am. J. Bot.* **95,** 626–641 (2008).

27. Hill, C. Jurassic *Angiopteris* (Marattiales) from North Yorkshire. *Rev. Palaeobot. Palynol.* **51,** 65–93 (1987).

28. Bek, J. & Pšenička, J. *Senftenbergia plumosa* (Artis) emend. and its spores from the Carboniferous of the Kladno and Pilsen basins, Bohemian Massif, and some related and synonymous taxa. *Rev. Palaeobot. Palynol.* **116,** 213–232 (2001).

29. Wang, S.-J., Hilton, J., He, X.-Y., Seyfullah, L. J. & Shao, L. The anatomically preserved stem *Zhongmingella* gen. nov. from the Upper Permian of China: evaluating the early evolution and phylogeny of the Osmundales. *J. Syst. Palaeontol.* **12,** 1–22 (2014).

30. Rößler, R. & Galtier, J. First *Grammatopteris* tree ferns from the Southern Hemisphere–new insights in the evolution of the Osmundaceae from the Permian of Brazil. *Rev. Palaeobot. Palynol.* **121,** 205–230 (2002).

31. Schuettpelz, E. & Pryer, K. M. Evidence for a Cenozoic radiation of ferns in an angiosperm-

dominated canopy. *Proc. Natl. Acad. Sci.* **106,** 11200–11205 (2009).

32. Phipps, C. *et al. Osmunda* (Osmundaceae) from the Triassic of Antarctica: an example of evolutionary stasis. *Am. J. Bot.* **85,** 888–895 (1998).

33. Metzgar, J. S., Skog, J. E., Zimmer, E. A. & Pryer, K. M. The paraphyly of *Osmunda* is confirmed by phylogenetic analyses of seven plastid loci. *Syst. Bot.* **33,** 31–36 (2008).

34. Miller, C. N. *Evolution of the fern family Osmundaceae based on anatomical studies*. (Museum of Paleontology, U. of Michigan, 1971).

35. Jud, N. A., Rothwell, G. W. & Stockey, R. A. *Todea* from the Lower Cretaceous of western North America: implications for the phylogeny, systematics, and evolution of modern Osmundaceae. *Am. J. Bot.* **95,** 330–339 (2008).

36. Serbet, R. & Rothwell, G. W. *Osmunda cinnamomea* (Osmundaceae) in the Upper Cretaceous of western North America: additional evidence for exceptional species longevity among filicalean ferns. *Int. J. Plant Sci.* **160,** 425–433 (1999).

37. Miller, C. N. *Evolution of the fern genus Osmunda*. (Museum of Paleontology, University of Michigan, 1967).

38. Axsmith, B. J., Krings, M. & Taylor, T. N. A filmy fern from the Upper Triassic of North Carolina (USA). *Am. J. Bot.* **88,** 1558–1567 (2001).

39. Klavins, S. D., Taylor, T. N. & Taylor, E. L. Matoniaceous ferns (Gleicheniales) from the middle Triassic of Antarctica. *J. Paleontol.* **78,** 211–217 (2004).

40. Herendeen, P. S. & Skog, J. E. *Gleichenia chaloneri*-a new fossil fern from the lower Cretaceous (Albian) of England. *Int. J. Plant Sci.* 870–879 (1998).

41. Gandolfo, M., Nixon, K., Crepet, W. & Ratcliffe, G. A new fossil fern assignable to Gleicheniaceae from Late Cretaceous sediments of New Jersey. *Am. J. Bot.* **84,** 483–483 (1997).

42. Wikström, N., Kenrick, P. & Vogel, J. C. Schizaeaceae: a phylogenetic approach. *Rev.*

Palaeobot. Palynol. **119,** 35–50 (2002).

43. Rothwell, G. W. Complex Paleozoic Filicales in the evolutionary radiation of ferns. *Am. J. Bot.* **74,** 458–461 (1987).

44. Kvaček, J., Dašková, J. & Renáta, P. A new schizaeaceous fern, *Schizaeopsis ekrtii* sp. nov., and its in situ spores from the Upper Cretaceous (Cenomanian) of the Czech Republic. *Rev. Palaeobot. Palynol.* **140,** 51–60 (2006).

45. Skog, J. E. The relationship of the fossil fern *Schizaeaopsis* Berry to modern genera in the Schizaeaceae. *Am. Fern J.* **83,** 20–29 (1993).

46. Hernandez-Castillo, G. R., Stockey, R. A. & Rothwell, G. W. *Anemia quatsinoensis* sp. nov. (Schizaeaceae), a permineralized fern from the Lower Cretaceous of Vancouver Island. *Int. J. Plant Sci.* **167,** 665–674 (2006).

47. Dettmann, M. E. & Clifford, H. T. Phylogeny and biogeography of *Ruffordia*, *Mohria* and *Anemia* (Schizaeaceae) and *Ceratopteris* (Pteridaceae): evidence from in situ and dispersed spores. *Alcheringa* **16,** 269–314 (1992).

48. Skog, J. E. The lower cretaceous ferns in the genus *Anemia* (Schizaeceae), Potomac group of Virginia, and relationships within the genus. *Rev. Palaeobot. Palynol.* **70,** 279–295 (1992).

49. Yamada, T. & Kato, M. *Regnellites nagashimae* gen. et sp. nov., the oldest macrofossil of Marsileaceae, from the Upper Jurassic to Lower Cretaceous of western Japan. *Int. J. Plant Sci.* **163,** 715–723 (2002).

50. Lupia, R., Schneider, H., Moeser, G., Pryer, K. & Crane, P. Marsileaceae sporocarps and spores from the Late Cretaceous of Georgia, USA. *Int. J. Plant Sci.* **161,** 975–988 (2000).

51. Hu, S., Taylor, D. W., Brenner, G. J. & others. A new marsilealean fern species from the Early Cretaceous of Jordan. *Palaeoworld* **17,** 235–245 (2008).

52. Nagalingum, N. S., Nowak, M. D. & Pryer, K. M. Assessing phylogenetic relationships in extant

heterosporous ferns (Salviniales), with a focus on *Pilularia* and *Salvinia*. *Bot. J. Linn. Soc.* **157,** 673–685 (2008).

53. Collinson, M. E. in *Pollen and spores* (eds. Blackmore, S. & Barnes, S. H.) 119–150 (Clarendon, Oxford, 1991).

54. Metzgar, J. S., Schneider, H. & Pryer, K. M. Phylogeny and divergence time estimates for the fern genus *Azolla* (Salviniaceae). *Int. J. Plant Sci.* **168,** 1045–1053 (2007).

55. Skog, J. E. *Loxsomopteris anasilla*, a new fossil fern rhizome from the Cretaceous of Maryland. *Am. Fern J.* **66,** 8–14 (1976).

56. Collinson, M. E. in *Pteridology in Perspective* (eds. Camus, J., Gibby, M. & Johns, R. J.) 349–394 (Royal Botanic Gardens, Kew, 1996).

57. Cantrill, D. J. Early Cretaceous fern foliage from President Head, Snow Island, Antarctica. *Alcheringa* **22,** 241–258 (1998).

58. Smith, S. Y., Rothwell, G. W. & Stockey, R. A. *Cyathea cranhamii* sp. nov. (Cyatheaceae), anatomically preserved tree fern sori from the Lower Cretaceous of Vancouver Island, British Columbia. *Am. J. Bot.* **90,** 755–760 (2003).

59. Nishida, H. Structure and affinities of the petrified plants from the Cretaceous of Japan and Saghalien, V. Tree fern stems from Hokkaido, *Paracyathocaulis ogurae* gen. et comb. nov. and *Cyathocaulis yezopteroides* sp. nov. *Bot. Mag.* **102,** 255–282 (1989).

60. Lantz, T. C., Rothwell, G. W. & Stockey, R. A. *Conantiopteris schuchmanii*, gen. et sp. nov., and the role of fossils in resolving the phylogeny of Cyatheaceae sl. *J. Plant Res.* **112,** 361–381 (1999).

61. Schneider, H. & Kenrick, P. An Early Cretaceous root-climbing epiphyte (Lindsaeaceae) and its significance for calibrating the diversification of polypodiaceous ferns. *Rev. Palaeobot. Palynol.* **115,** 33–41 (2001).

62. Tidwell, W. D. & Ash, S. R. A review of selected triassic to Early Cretaceous ferns. *J. Plant Res.* **107,** 417–442 (1994).

63. Krassilov, V. & Bacchia, F. Cenomanian florule of Nammoura, Lebanon. *Cretac. Res.* **21,** 785–799 (2000).

64. Bonde, S. D. & Kumaran, K. The oldest macrofossil record of the mangrove fern *Acrostichum* L. from the Late Cretaceous Deccan Intertrappean beds of India. *Cretac. Res.* **23,** 149–152 (2002).

65. Serbet, R. & Rothwell, G. W. Anatomically preserved ferns from the Late Cretaceous of western North America: Dennstaedtiaceae. *Int. J. Plant Sci.* **164,** 1041–1051 (2003).

66. Page, C. The taxonomy and phytogeography of bracken—a review. *Bot. J. Linn. Soc.* **73,** 1–34 (1976).

67. Der, J. P., Thomson, J. A., Stratford, J. K. & Wolf, P. G. Global chloroplast phylogeny and biogeography of bracken (*Pteridium*; Dennstaedtiaceae). *Am. J. Bot.* **96,** 1041–1049 (2009).

68. Su, T. *et al.* A new *Drynaria* (Polypodiaceae) from the upper Pliocene of Southwest China. *Rev. Palaeobot. Palynol.* **164,** 132–142 (2011).

69. Skog, J. E. Biogeography of Mesozoic leptosporangiate ferns related to extant ferns. *Brittonia* **53,** 236–269 (2001).

70. Deng, S. Ecology of the Early Cretaceous ferns of northeast China. *Rev. Palaeobot. Palynol.* **119,** 93–112 (2002).

71. Rothwell, G. W. & Stockey, R. A. Combining characters of Pteridaceae and tree ferns: *Pterisorus radiata* gen. et sp. nov., a permineralized Lower Cretaceous filicalean with radial sori. *Int. J. Plant Sci.* **167,** 695–701 (2006).

72. Collinson, M. E. Cainozoic ferns and their distribution. *Brittonia* **53,** 173–235 (2001).

73. Smith, A. R. & Cranfill, R. B. Intrafamilial relationships of the thelypteroid ferns (Thelypteridaceae). *Am. Fern J.* **92,** 131–149 (2002).

74. Rothwell, G. W. & Stockey, R. A. *Onoclea sensibilis* in the Paleocene of North America, a dramatic example of structural and ecological stasis. *Rev. Palaeobot. Palynol.* **70,** 113–124 (1991).

75. Wang, Q., Ablaev, A. G., Wang, Y. & Li, C. Paleocene Wuyun flora in northeast China: *Woodwardia bureiensis*, *Dryopteris* sp. and *Osmunda sachalinensis*. *Acta Phytotaxon. Sin.* **44,** 712–720 (2005).

76. Pigg, K. B. & Rothwell, G. W. Anatomically preserved *Woodwardia virginica* (Blechnaceae) and a new filicalean fern from the middle Miocene Yakima Canyon flora of central Washington, USA. *Am. J. Bot.* **88,** 777–787 (2001).

77. Borgardt, S. J. & Pigg, K. B. Anatomical and developmental study of petrified *Quercus* (Fagaceae) fruits from the Middle Miocene, Yakima Canyon, Washington, USA. *Am. J. Bot.* **86,** 307–325 (1999).

78. Stockey, R. A., Nishida, H. & Rothwell, G. W. Permineralized ferns from the middle Eocene Princeton chert. I. *Makotopteris princetonensis* gen. et sp. nov. (Athyriaceae). *Int. J. Plant Sci.* **160,** 1047–1055 (1999).

79. Scotese, C. R. *PointTracker, PALEOMAP Project, University of Texas at Arlington*. (2004).

80. Silvestro, D., Cascales-Miñana, B., Bacon, C. D. & Antonelli, A. Revisiting the origin and diversification of vascular plants through a comprehensive Bayesian analysis of the fossil record. *New Phytol.* **207,** 425–436 (2015).

81. Scotese, C. R. *Some thoughts on global climate change: the transition from icehouse to hothouse, in the Earth history: the evolution of the Earth system. https://www.academia.edu/12082909/.* (PALEOMAP Project, Evanston, IL., 2016).

82. Berner, R. A. & Kothavala, Z. Geocarb III: a revised model of atmospheric CO2 over Phanerozoic time. *Am. J. Sci.* **301,** 182–204 (2001).

83. Berner, R. A. Phanerozoic atmospheric oxygen: new results using the GEOCARBSULF model. *Am. J. Sci.* **309,** 603–606 (2009).

84. Snedden, J. W. & Liu, C. A compilation of Phanerozoic sea-level change, coastal onlaps and recommended sequence designations. *Search and Discovery Article* 40594 (2010).

85. Gastil, R. G. The distribution of mineral dates in time and space. *Am. J. Sci.* **258,** 1–35 (1960).

86. Brink, H.-J. Periodic signals of the Milky Way concealed in terrestrial sedimentary basin fills and in planetary magmatism? *Int. J. Geosci.* **6,** 831–845 (2015).

87. Cogné, J.-P. & Humler, E. Global scale patterns of continental fragmentation: Wilson's cycles as a constraint for long-term sea-level changes. *Earth Planet. Sci. Lett.* **273,** 251–259 (2008).

88. Large, R. R. *et al.* Cycles of nutrient trace elements in the Phanerozoic ocean. *Gondwana Res.* **28,** 1282–1293 (2015).

89. Long, J. A. *et al.* Severe selenium depletion in the Phanerozoic oceans as a factor in three global mass extinction events. *Gondwana Res.* **36,** 209–218 (2015).

90. Rabosky, D. L. *et al.* Rates of speciation and morphological evolution are correlated across the largest vertebrate radiation. *Nat Commun* **4,** (2013).

91. Rabosky, D. L. Automatic detection of key innovations, rate shifts, and diversity-dependence on phylogenetic trees. *PLoS ONE* **9,** e89543 (2014).

92. Team, R. C. *R: a language and environment for statistical computing. Vienna, Austria; 2015*. (2015).

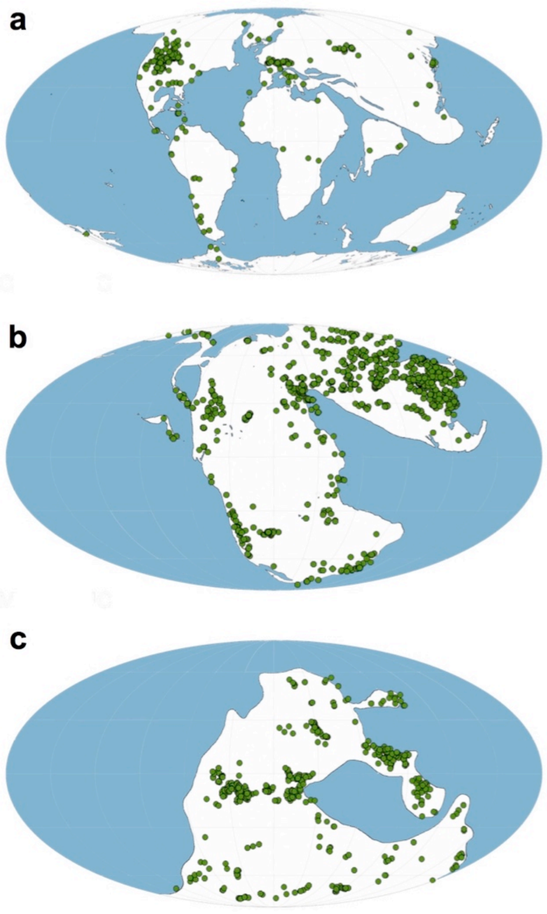

Supplementary Figure 1. Paleodistribution of the fossil data used in this study. Maps show continental positions at **a**, 50 Ma, **b**, 180 Ma and **c**, 320 Ma[5], and green dots show locations of fern fossils with mean age estimate falling between **a**, 99.6 and 0 Ma, **b**, 251 and 99.6 Ma and **c**, 388 and 251 Ma. Maps are based on PALEOMAP Global Plate Tectonic Model[5] and were created using Adobe Photoshop CC 2015, PointTracker[79] and QGIS v. 2.0.1 (http://www.qgis.org).

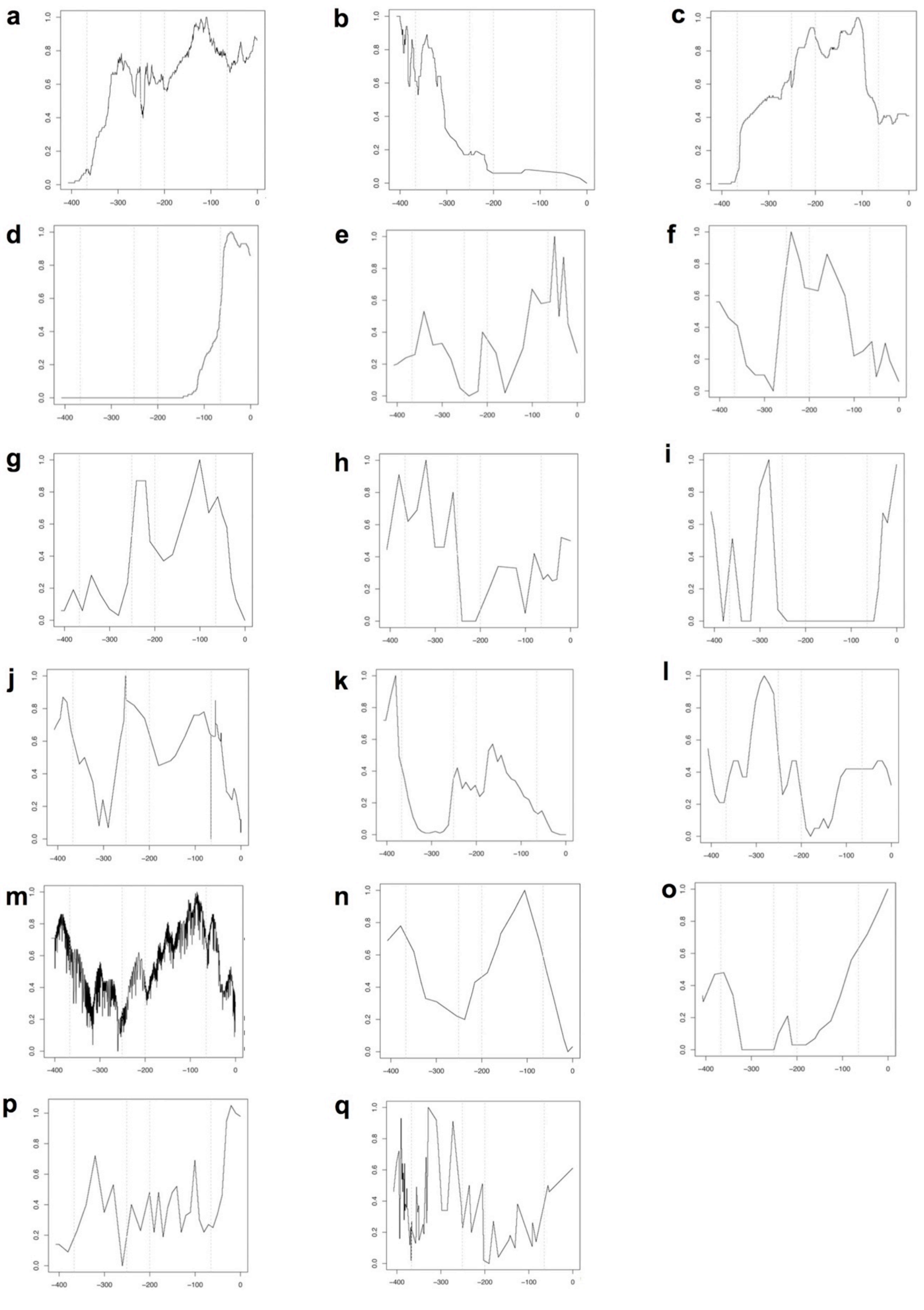

Supplementary Figure 2. Paleoenvironmental trajectories used in this study. Diversity of **a**, ferns, **b**, lycophytes etc. (free-sporing vascular plants excluding ferns), **c**, gymnosperms[80], **d**, angiosperms[80]. Extent of the biomes: **e**, wet tropical, **f**, arid, **g**, warm temperate, **h**, cool temperate, **i**, polar. **j**, Global

mean temperature[81]. **k**, Atmospheric CO_2[82]. **l**, Atmospheric O_2[83]. **m**, Eustatic sealevel[84]. **n**, Magmatic activity[85,86]. **o**, Continental fragmentation[87]. **p**, Mountain area. **q**, Selenium concentration in marine sediments[88,89].

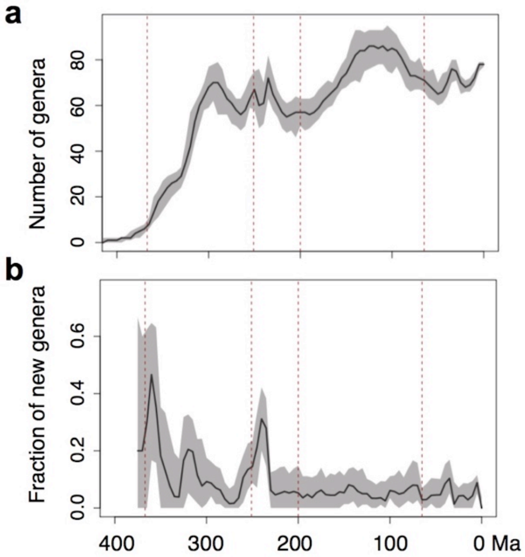

Supplementary Figure 3. Dynamics of fern generic diversity through time. a, The range-through diversity trajectory of sampled genera based on the estimated times of origination and extinction. **b**, The amount of turnover calculated as the fraction of new genera originating in a 5 Ma time bin out of the total number of genera recorded within the time bin. Global mass extinction events are indicated by dotted red lines.

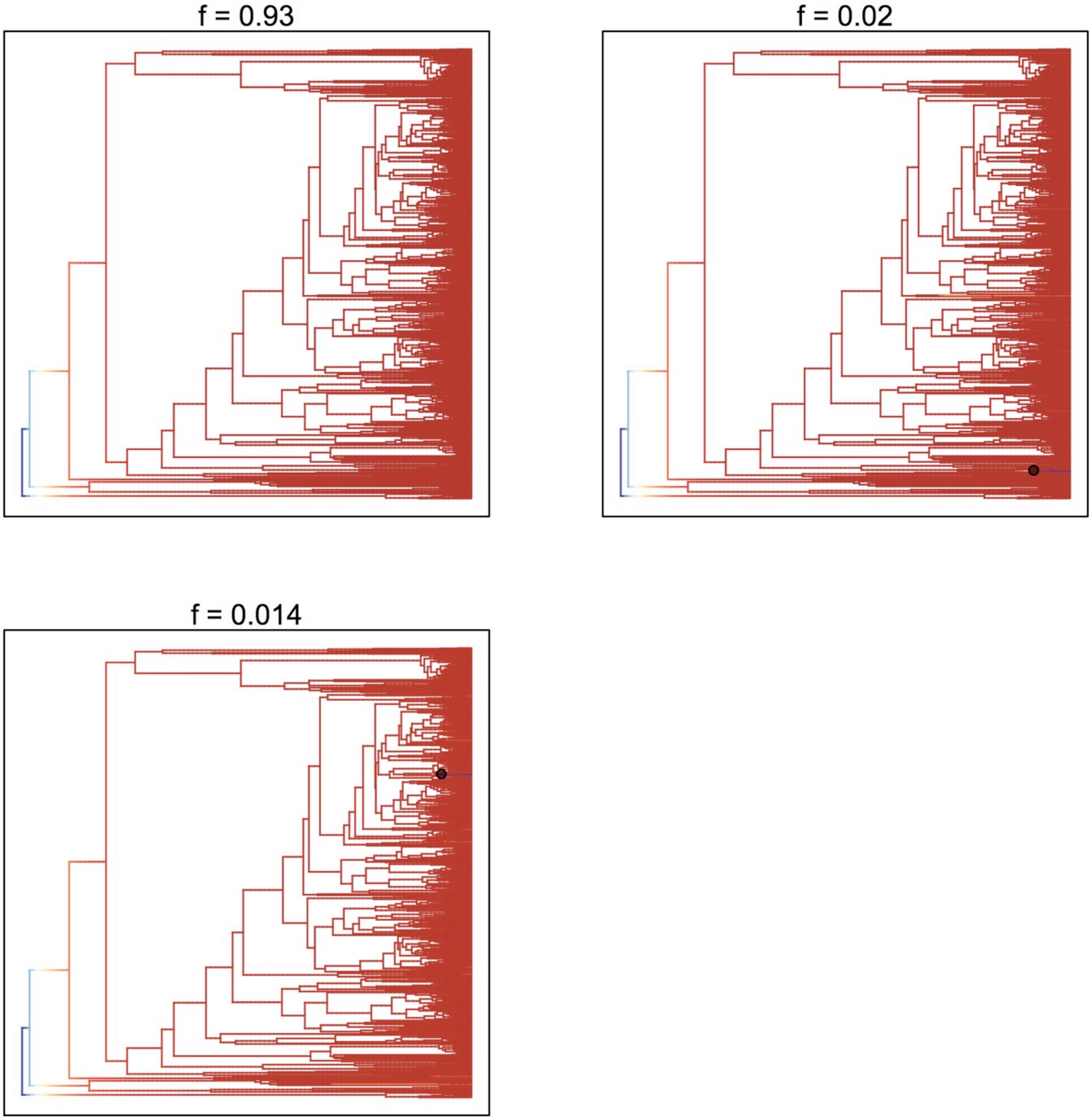

Supplementary Figure 4. Rates of climatic niche evolution in ferns. The three rate shift configurations in the 95% credible set of shift configurations with their overall frequencies for the evolution of climatic niche across the fern phylogeny as estimated with BAMM[90,91]. No rate changes are supported by the most frequent configuration.

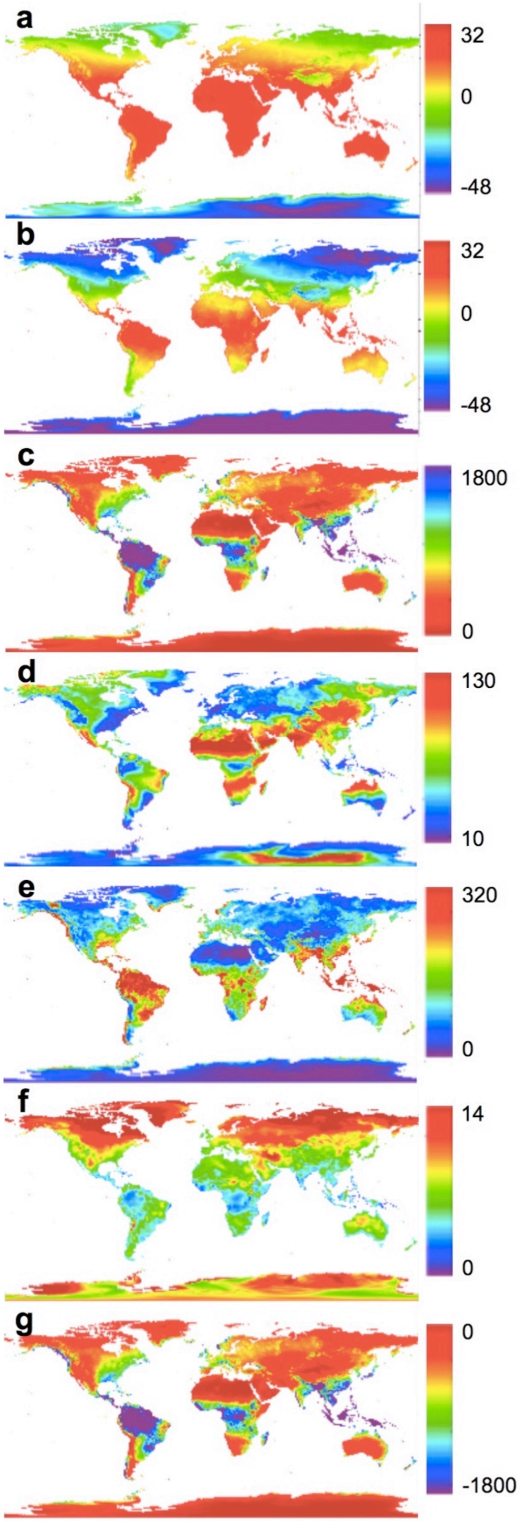

Supplementary Figure 5. Climatic niche components. a, Bio1 (annual mean temperature). **b**, Bio6 (min temperature of coldest month). **c**, Bio12 (annual precipitation). **d**, Bio15 (precipitation

seasonality). **e**, interannual precipitation variation. **f**, interannual temperature variation. **g**, The first principal component of the four climatic layers. Maps were created using the statistical programming language R[92].

Supplementary Table 1. Origination and extinction times of fern genera as estimated from the fossil record.

Taxon	Origination (Ma)			Extinction (Ma)		
	Mean	95% CI		Mean	95% CI	
**Pseudoborniales**						
Pseudobornia	380.00	362.36	398.57	363.64	354.79	381.90
**Sphenophyllales**						
Bowmanites	355.29	337.24	370.08	270.04	255.15	279.11
Cheirostrobus	346.63	336.67	366.44	321.23	286.22	339.06
Eviostachya	369.36	364.78	374.94	360.55	358.99	363.61
Gondwanophyton	299.73	272.76	345.26	267.47	245.05	288.86
Hamatophyton	382.99	372.41	393.02	363.52	359.01	374.80
Lilpopia	316.00	299.22	350.83	269.95	245.56	296.91
Parasphenophyllum	317.15	304.02	358.17	292.42	252.87	305.93
Peltastrobus	319.78	300.04	359.79	294.69	252.76	317.22
Rotafolia	369.52	359.07	379.25	362.70	353.10	373.93
Sentistrobus	314.56	299.03	355.03	289.63	251.82	305.85
Sphenophyllostachys	346.86	315.60	370.82	291.06	257.32	303.08
Sphenophyllum	367.70	364.81	370.39	251.55	250.65	252.63
Xihuphyllum	374.49	359.15	388.91	368.15	358.98	384.61
**Equisetales**						
Annularia	349.23	328.28	365.23	247.83	243.79	252.15
Archaeocalamites	359.91	352.90	366.27	281.60	274.52	289.31
Arthropitys	334.59	317.83	357.60	246.31	241.83	250.73
Asterophyllites	358.83	356.59	360.29	257.64	251.75	264.38
Austroannularia	288.81	272.65	319.47	250.69	240.64	259.59
Bengaliaraniganjensis	301.34	271.34	345.38	270.10	245.32	297.51
Benlightfootia	296.36	273.21	327.53	251.38	242.08	259.08
Calamites	359.00	357.16	360.46	173.09	171.04	175.31
Calamostachys	357.77	349.00	365.23	231.18	225.50	236.80
Cruciatheca	300.25	285.72	318.53	279.83	253.83	292.78
Equisetinostachys	286.16	274.79	308.03	252.17	243.88	258.73
Equisetites	325.86	299.92	354.71	59.55	58.70	60.84

Equisetocaulis	229.06	204.62	245.73	202.34	145.52	227.34
Equisetum	342.03	324.72	359.39	extant		
Giridia	296.87	284.59	318.41	273.34	250.20	286.63
Gondwanites	320.88	299.09	359.93	296.00	254.36	317.70
Gondwanostachys	298.97	272.53	343.53	266.91	245.26	289.62
Lelstotheca	262.15	251.04	293.50	252.37	243.76	259.99
Neocalamites	297.50	295.68	299.21	130.56	102.80	153.92
Notocalamites	303.19	284.04	347.16	271.15	246.20	287.94
Paracalamites	300.91	295.24	309.70	252.37	245.24	259.39
Paracalamitina	287.57	260.21	336.05	253.87	239.68	268.68
Peltotheca	311.41	297.03	351.53	283.35	249.70	298.97
Phyllotheca	302.35	294.47	312.28	226.36	215.63	234.65
Raniganjia	351.98	337.13	370.49	247.32	235.66	255.82
Sakoarota	302.49	280.66	333.87	269.33	245.19	293.15
Schizoneura	295.13	291.03	299.6	153.73	150.67	157.05
Spaciinodum	245.29	241.07	253.36	239.52	229.87	244.91
Tchernovia	300.61	251.15	349.32	274.60	245.14	316.36
Umbellaphyllites	292.75	287.88	300.16	248.67	245.92	251.57
**Ophioglossales**						
Botrychium	78.10	57.87	110.55	extant		
Ophioglossum	24.34	16.41	36.39	extant		
**Psilotales**						
Psilotum	41.78	16.09	87.40	extant		
Tmesipteris	37.84	28.57	51.13	extant		
**Marattiales**						
Acitheca	322.31	311.79	332.95	252.93	246.29	259.87
Angiopteris	231.95	208.94	247.17	extant		
Asterotheca	340.20	310.45	361.07	207.11	201.62	214.98
Boreopteris	252.64	248.47	257.13	246.82	242.6	250.82
Caulopteris	407.87	391.63	423.28	245.74	239.32	251.05
Danaea	69.67	35.45	128.24	extant		
Danaeites	355.11	322.02	375.08	79.51	44.59	105.27
Danaeopsis	260.31	254.33	265.51	86.58	84.30	88.55
Dizeugotheca	290.48	274.75	307.23	250.77	244.16	256.76
Drepanozamites	239.28	230.97	250.70	187.45	144.05	203.98
Eoangiopteris	324.64	307.06	360.02	249.16	237.78	259.29
Gemellitheca	287.12	251.09	336.57	261.10	243.47	298.00
Goolandia	115.95	93.60	165.89	87.91	58.77	104.90
Grandeuryella	317.16	304.81	356.31	292.88	254.47	305.87
Herbstopteris	240.50	235.07	246.94	234.13	218.90	240.89

Lobatopteris	321.19	314.69	328.21	302.77	298.82	305.43
Marantoidea	240.70	235.05	247.81	233.75	218.43	240.95
Marattia	228.47	214.15	242.05	extant		
Marattiopsis	245.88	241.07	253.56	82.82	72.85	88.45
Megaphyton	353.05	314.32	377.35	253.39	240.49	262.19
Mesozoisynangia	110.88	94.09	138.99	90.41	69.52	103.65
Myriotheca	348.48	313.21	371.27	294.61	258.85	305.77
Ogmos	242.83	235.03	252.32	236.29	221.17	244.98
Pecopteris	378.55	368.32	385.03	41.54	32.49	51.52
Pectinangium	302.34	274.38	335.26	269.14	245.00	292.47
Protoblechnum	261.64	251.01	294.38	251.76	243.04	259.90
Psaronius	393.40	363.64	419.69	248.94	241.91	254.60
Ptychocarpus	326.72	302.56	364.38	248.67	236.41	259.35
Qasimia	293.26	253.67	335.02	249.14	238.59	259.31
Radstockia	320.63	300.39	360.43	296.26	254.99	317.41
Rajahia	260.48	252.99	268.69	250.87	246.00	256.26
Rhinipteris	229.41	202.90	247.22	205.41	151.62	234.75
Rienitsia	233.46	213.42	246.76	201.73	169.62	225.36
Scolecopteris	321.76	315.02	332.04	239.94	234.40	244.78
Sydneia	321.49	307.09	359.96	296.79	254.39	311.34
Symopteris	241.75	236.77	247.12	199.59	190.53	203.94
Taiyuanitheca	261.77	251.06	294.50	251.83	243.12	259.72
Tietea	297.71	256.59	341.00	257.45	241.64	292.34
Tranquillia	228.03	203.22	245.61	202.70	148.73	227.51
Tuvichapteris	293.22	251.83	335.95	246.70	236.17	253.72
Zhutheca	261.15	251.09	290.75	251.91	243.16	259.88
**Paleozoic leptosporangiate ferns**						
Anachoropteris	348.10	322.39	369.16	251.80	245.24	259.02
Ankyropteris	351.10	325.32	367.07	274.69	253.76	292.26
Apotropteris	314.78	299.20	356.91	291.00	253.87	305.94
Botryopteris	357.63	346.36	367.34	268.08	246.85	292.83
Catenopteris	317.41	299.03	357.87	292.51	253.05	311.56
Doneggia	314.81	299.23	356.16	289.62	252.51	305.87
Kaplanopteris	314.77	299.11	354.98	289.25	251.67	305.92
Norwoodia	324.94	311.55	361.26	300.54	256.93	314.56
Psalixochlaena	354.30	331.07	373.65	298.84	270.71	312.77
Rhabdoxylon	322.99	312.58	344.14	298.27	273.70	311.73
Senftenbergia	351.26	329.16	369.00	260.79	245.92	280.79
Sermaya	319.97	306.24	350.05	292.30	257.92	305.52
Skaaripteris	261.21	251.06	291.41	251.90	243.46	259.81

Tubicaulis	320.77	312.56	332.04	251.57	244.21	259.64
**Osmundales**						
Anomopteris	253.70	251.70	257.11	219.22	201.61	233.76
Ashicaulis	249.93	241.04	264.82	70.99	41.36	98.30
Aurealcaulis	69.80	57.91	85.64	30.80	23.01	38.03
Bathypteris	261.15	251.14	292.83	251.61	243.2	259.54
Birmoltia	236.91	228.37	243.99	224.84	209.36	234.20
Birtodites	238.41	228.36	247.76	220.56	196.21	234.99
Cacumen	161.54	136.96	213.62	88.47	58.16	111.07
Chasmatopteris	261.43	251.01	289.26	252.32	243.94	259.86
Cladophlebis	291.58	286.99	298.91	40.06	33.95	50.55
Donwelliacaulis	242.93	235.34	251.45	236.68	222.62	244.77
Elantodites	236.02	231.34	240.10	227.2	220.63	231.33
Grammatopteris	347.99	326.93	368.65	252.84	238.96	274.67
Guairea	300.34	251.94	352.63	194.38	92.30	292.80
Iegosigopteris	260.99	251.02	287.52	251.64	243.11	259.60
Itopsidema	231.12	204.47	247.95	206.26	149.68	234.94
Leptopteris	36.76	28.78	47.36	extant		
Lunea	192.32	148.49	241.07	149.44	86.73	199.26
Millerocaulis	234.38	214.3	245.72	106.11	85.44	122.41
Nymbopteron	241.88	235.31	251.24	223.61	206.49	234.9
Osmunda	220.75	203.94	236.93	extant		
Osmundacaulis	249.66	241.03	262.25	70.54	36.56	96.96
Osmundastrum	73.97	40.67	133.15	extant		
Osmundopsis	243.60	236.03	254.55	126.55	91.31	144.93
Paleosmunda	260.97	251.07	290.48	251.40	242.90	259.54
Parsorophyllum	248.19	241.22	258.28	222.55	204.50	234.72
Phyllopteroides	148.01	138.68	158.76	91.72	81.27	99.21
Rastropteris	307.33	284.19	350.20	276.43	247.74	298.55
Rooitodites	236.32	231.58	241.81	226.91	219.24	232.51
Shuichengella	261.70	252.05	281.37	251.18	243.09	258.49
Sphenopteris	326.42	321.78	331.68	33.65	28.29	39.43
Stormbergia	240.57	235.07	247.08	225.42	213.05	233.95
Thamnopteris	287.00	273.48	315.65	265.83	247.72	278.23
Todea	192.13	172.31	235.79	extant		
Todites	257.66	252.88	260.78	119.43	99.77	142.16
Zalesskya	262.02	251.09	294.81	251.94	242.93	259.65
Zhongmingella	257.58	252.21	275.22	249.82	242.32	254.19
**Hymenophyllales**						
Eogonocormus	146.99	105.53	195.28	101.73	68.98	144.47

Hopetedia	238.42	228.23	248.23	221.20	198.08	234.95
Hymenophyllites	362.55	345.11	377.92	97.41	61.54	134.10
Hymenophyllum	94.55	85.95	106.37	extant		
Trichomanes	86.13	66.77	116.20	extant		
**Gleicheniales**						
Aninopteris	204.05	192.26	218.82	176.19	156.41	192.15
Antarctipteris	245.78	241.05	254.05	239.77	230.70	244.91
Boodlepteris	101.36	89.33	128.17	76.31	57.19	85.73
Camptopteris	247.52	235.22	256.42	195.14	185.86	201.71
Chansitheca	301.81	280.55	331.85	248.06	238.47	253.93
Clathropteris	248.34	240.03	255.08	149.32	140.42	161.02
Delosorus	139.37	113.30	194.89	104.41	67.64	128.76
Dicranopteris	87.57	81.37	97.97	extant		
Dictyophyllum	249.47	244.37	253.75	145.58	142.89	149.15
Gleichenia	178.50	174.42	182.67	extant		
Gleicheniopsis	133.83	100.89	177.89	48.29	25.13	63.97
Gleichenipteris	243.15	235.54	252.17	237.00	223.75	244.98
Gleichenites	245.21	242.50	247.53	49.82	34.90	58.51
Goeppertella	227.44	203.85	245.00	149.84	120.73	167.02
Hausmannia	241.30	238.51	245.11	60.16	58.98	61.49
Kenderlykia	209.05	179.17	244.91	165.28	95.06	203.87
Konijnenburgia	101.53	94.62	111.44	91.80	82.10	97.12
Laccopteris	243.31	226.00	255.84	50.40	33.03	59.52
Matonia	207.65	179.88	242.22	extant		
Matonidium	183.66	171.83	207.34	76.78	60.48	85.24
Microphyllopteris	258.69	247.22	289.83	85.43	58.55	106.02
Nathorstia	191.14	150.08	240.50	64.48	31.32	91.29
Oligocarpia	352.79	329.76	369.55	210.01	196.69	227.39
Phlebopteris	239.86	235.58	244.26	102.71	92.64	115.24
Piazopteris	207.38	179.55	242.18	97.80	61.92	133.49
Selenocarpus	211.78	191.51	241.76	154.49	116.56	175.35
Soloropteris	245.90	241.08	254.34	239.96	231.13	244.99
Sticherus	87.17	66.10	117.16	extant		
Szea	295.05	271.18	340.55	262.34	243.81	283.46
Tasmanopteris	194.31	151.39	241.77	151.19	86.89	198.73
Thaumatopteris	244.82	236.24	255.95	178.59	159.46	195.16
Tomaniopteris	242.34	235.16	251.02	236.05	222.22	244.95
Weichselia	187.45	175.05	200.19	79.12	69.67	84.40
Wingatea	231.16	209.17	245.87	202.8	170.56	220.49
**Schizaeales**						

Anemia	140.01	131.13	146.54	extant		
Baieropsis	141.90	111.66	170.41	76.76	52.93	104.44
Cynepteris	249.18	237.73	258.57	202.79	191.24	215.84
Eleganopteris	252.98	247.78	258.91	245.17	238.68	250.74
Klukia	189.73	178.76	201.77	108.71	99.32	115.14
Klukiopsis	189.65	161.88	239.50	143.11	83.54	175.66
Lygodium	111.02	81.89	144.86	extant		
Mohriopsis	166.87	146.9	202.18	138.88	100.00	158.86
Norimbergia	229.92	201.67	245.48	205.88	149.7	234.21
Paralygodium	51.80	34.51	77.00	32.56	22.90	46.53
Pekinopteris	228.94	202.24	246.63	203.43	148.02	234.20
Pelletixia	144.79	136.16	159.41	108.20	89.51	124.08
Ruffordia	161.49	140.68	201.24	30.80	8.78	47.79
Schizaea	64.01	56.05	75.90	extant		
Schizaeangium	151.53	104.21	210.47	115.43	70.12	158.99
Schizaeopsis	140.01	114.60	180.89	71.35	39.46	98.86
Schizaeopteris	95.03	65.69	139.40	73.00	42.85	99.24
Stachypteris	200.59	177.29	238.65	152.28	117.96	171.69
**Salviniales**						
Azinia	95.39	66.22	137.16	74.01	45.31	99.52
Azolla	126.74	99.47	145.32	extant		
Hydropteris	71.04	68.59	73.55	65.60	62.82	67.84
Marsilea	194.75	178.21	210.06	extant		
Marsileaceaephyllum	112.47	108.98	116.65	99.15	95.04	102.81
Mendozaphyllum	87.15	65.53	130.59	65.49	34.65	83.49
Nammouria	111.84	93.52	160.61	86.06	58.36	99.47
Parazolla	80.41	65.57	119.45	60.04	31.46	70.51
Pilularia	89.70	66.22	123.21	extant		
Regnellidium	102.2	83.83	142.19	extant		
Regnellites	169.00	140.18	233.49	131.84	81.24	159.96
Rodeites	78.11	61.05	116.81	56.59	29.08	68.91
Salvinia	83.34	69.34	98.97	extant		
**Cyatheales**						
Acanthopteris	164.27	156.67	177.18	105.93	87.17	129.92
Alienopteris	137.65	112.86	194.44	101.83	65.19	124.72
Alsophila	16.13	2.64	53.15	extant		
Alsophilites	192.54	149.33	240.23	121.24	84.69	139.78
Alsophilocaulis	40.00	23.08	75.20	23.39	2.37	33.32
Cibotiocaulis	124.05	65.69	190.28	94.08	54.82	143.67
Cibotium	101.11	66.95	143.19	extant		

Conantiopteris	137.12	112.1	195.24	101.36	63.33	124.53
Coniopteris	329.43	303.57	358.08	37.15	28.36	46.31
Culcitites	172.75	146.01	235.63	134.36	82.61	160.57
Cyathea	222.57	203.47	239.62	extant		
Cyathocarpus	314.82	298.99	355.06	290.13	252.63	306.7
Cyathocaulis	171.81	145.92	210.31	64.03	30.42	89.69
Cyathodendron	55.26	53.27	56.87	33.52	32.38	34.28
Dendropteridium	27.26	6.09	53.60	5.44	0.00	17.17
Dicksonia	203.66	192.1	215.19	extant		
Eboracia	206.92	197.96	221.27	116.64	90.29	142.2
Eocyathea	136.60	112.27	190.14	102.27	66.99	124.82
Gonatosorus	214.70	193.5	244.62	102.64	67.59	142.75
Haydenia	177.66	156.13	235.69	139.52	87.14	160.94
Kwazulupteris	115.76	91.69	148.52	87.62	63.30	107.81
Kylikipteris	207.44	181.45	242.14	138.71	98.49	157.19
Lophosoria	148.45	112.7	222.62	extant		
Loxsomopteris	137.07	112.35	192.68	102.59	64.99	124.91
Natalipteris	115.72	93.81	149.88	87.60	61.18	106.55
Nishidacaulis	142.65	101.55	203.43	107.72	67.69	143.9
Oguracaulis	152.13	102.68	214.99	116.22	71.30	160.39
Onychiopsis	182.03	168.74	203.01	83.03	80.02	85.25
Paracyathocaulis	112.87	65.78	168.63	85.44	50.00	124.32
Protopteris	147.38	127.13	180.55	81.23	59.89	93.28
Rickwoodopteris	88.89	70.61	127.25	68.02	39.70	83.42
Sergioa	138.32	112.31	187.66	74.22	46.73	88.66
Solenostelopteris	154.11	130.21	214.40	117.06	71.92	139.79
Thyrsopteris	340.63	304.41	372.07	extant		
**Polypodiales**						
Acrostichum	71.42	67.84	75.78	extant		
Adiantopteris	239.34	228.11	253.20	94.54	63.54	125.83
Adiantum	126.72	114.15	139.20	extant		
Allantodiopsis	89.92	79.48	100.83	33.22	30.87	35.00
Anogramma	6.10	2.65	15.90	extant		
Antrophyum	25.69	19.82	35.64	extant		
Arthropteris	10.27	1.83	42.68	extant		
Aspidistes	185.45	173.02	205.28	127.26	113.45	139.18
Asplenium	236.24	228.85	242.56	extant		
Astralopteris	132.17	100.12	174.07	87.06	66.15	97.26
Athyrium	155.79	127.85	199.84	extant		
Blechnum	151.01	141.00	163.04	extant		

Ceratopteris	58.71	34.59	94.73	extant		
Coniogramme	36.10	5.56	83.58	extant		
Cretacifilix	138.47	99.82	197.11	104.69	65.11	142.01
Cryptogrammites	42.12	28.47	74.43	26.28	6.44	33.82
Ctenitis	7.68	2.07	23.17	extant		
Cuyenopteris	39.08	23.02	73.30	23.13	2.40	32.97
Davallia	56.83	37.12	72.57	extant		
Dennastra	77.66	61.72	118.94	54.53	27.64	66.38
Dennstaedtia	175.48	161.69	190.77	extant		
Dennsteadtiopsis	50.71	40.74	70.04	30.38	23.03	36.42
Deparia	19.37	5.36	47.31	extant		
Diplazium	71.37	34.81	134.93	extant		
Drynaria	13.73	6.26	25.78	extant		
Dryopteris	173.58	164.55	180.63	extant		
Elaphoglossum	40.49	15.51	88.12	extant		
Eogymnocarpium	139.66	99.85	199.93	104.38	63.47	145.14
Grammitis	24.20	15.32	39.13	extant		
Hemionitis	81.67	57.77	123.27	extant		
Histiopteris	36.40	28.79	46.81	extant		
Hypolepis	23.14	16.15	33.99	extant		
Krameropteris	121.81	98.03	177.85	91.22	60.57	111.25
Lastreopsis	16.47	2.64	54.64	extant		
Lepisorus	38.04	28.70	52.09	extant		
Leptolepia	8.11	0.06	36.88	extant		
Lindsaea	40.64	34.07	50.81	extant		
Makotopteris	59.12	40.52	101.36	35.54	18.91	48.40
Microlepiopsis	86.43	71.78	109.24	68.57	50.79	82.72
Microsorum	47.09	37.70	57.46	extant		
Midlandia	88.43	70.68	128.09	67.59	38.66	83.38
Nephrolepis	10.64	5.39	17.68	extant		
Oleandra	134.53	119.57	152.44	extant		
Onoclea	112.70	94.05	141.68	extant		
Paesia	40.39	34.17	50.31	extant		
Pellaea	10.04	1.81	40.94	extant		
Pleopeltis	41.29	16.11	91.51	extant		
Polypodium	52.24	34.18	72.69	extant		
Polystichum	79.86	65.63	108.17	extant		
Protodrynaria	43.78	28.56	78.04	27.19	8.37	37.15
Pteridium	37.05	33.98	40.91	extant		
Pteris	99.83	95.61	104.76	extant		

Pyrrosia	5.60	3.64	9.20	extant		
Rumohra	49.74	34.46	66.21	extant		
Saccoloma	102.9	94.76	114.88	extant		
Salpichlaena	74.39	68.98	84.41	extant		
Stramineopteris	153.12	130.22	209.92	116.95	71.89	139.9
Tecaropteris	55.63	23.09	97.41	35.14	9.63	64.25
Tectaria	135.30	123.91	151.61	extant		
Thelypteridaceophyllum	7.82	1.83	31.06	1.71	0.08	3.17
Thelypteris	76.00	66.42	85.21	extant		
Trawetsia	55.73	37.34	92.61	34.09	16.55	48.46
Wessiea	97.48	70.75	149.79	5.63	0.01	14.40
Woodwardia	121.92	97.58	145.22	extant		
**Incertae cedis**						
Acrostichopteris	228.12	209.77	242.32	101.06	86.50	114.80
Adiantitophyllum	124.06	100.12	176.83	92.15	60.37	111.88
Asplenites	239.70	229.49	251.35	61.83	29.46	82.22
Boweria	355.30	329.70	376.35	311.2	271.78	349.97
Crenulopteris	318.51	314.97	323.44	302.42	297.86	305.14
Daeniopteris	254.78	245.32	274.99	203.07	163.95	233.80
Dictyonymba	242.78	235.11	251.93	235.73	220.34	244.91
Diplazites	316.65	304.08	354.62	292.00	252.83	305.95
Discopteris	320.49	300.39	360.80	296.08	254.98	317.60
Displinites	237.19	229.57	244.98	224.82	209.63	234.00
Dryopterites	147.38	121.65	177.66	97.73	68.36	117.11
Fremouwa	245.82	241.07	253.97	240.12	231.80	244.89
Gordonopteris	247.85	242.24	255.60	234.45	223.93	240.96
Gouldiopteris	242.82	235.22	251.83	236.40	221.21	244.98
Holmesopteris	317.07	304.36	340.51	299.23	273.46	312.57
Katasiopteris	253.92	251.34	258.17	245.67	239.89	250.73
Korallipteris	100.63	86.41	144.57	78.04	50.75	88.62
Leconama	242.81	235.02	251.65	236.63	222.96	244.87
Micronymbopteris	242.44	235.13	251.26	235.99	220.91	244.93
Molteniella	238.09	228.22	247.67	220.67	195.86	234.77
Neuropteridium	244.16	242.33	245.50	234.96	234.46	235.42
Nunatakia	124.27	99.96	177.83	92.33	60.22	111.95
Nymbofelicia	242.54	235.06	251.27	236.25	221.52	245.00
Nymboidiantum	239.78	235.60	244.62	226.89	218.88	232.09
Nymbophlebis	243.07	235.06	252.54	236.91	222.34	244.95
Nymborhipteris	243.10	235.14	252.04	236.40	221.32	244.83
Paratrizygia	297.04	273.34	329.02	267.49	245.56	287.35

Pterisorus	154.11	130.05	216.68	117.31	72.19	139.79
Ptilotonymba	242.97	235.05	251.58	236.50	222.40	244.96
Raphaelia	225.15	210.45	237.50	127.38	101.08	144.06
Renaultia	336.03	322.20	360.50	305.38	298.30	309.38
Schleporia	246.14	241.17	255.00	240.07	231.23	244.92
Schopfiopteris	245.83	241.22	254.44	239.79	231.03	244.96
Scolopendrites	253.71	251.71	257.12	238.26	233.28	243.61
Selleyopteris	172.97	151.03	235.61	134.13	83.26	155.67
Sendersonia	262.39	251.14	297.38	252.13	243.40	259.96
Speirsopteris	74.64	56.86	117.84	48.27	25.00	60.17
Tempskya	143.78	136.94	149.54	69.65	61.94	83.69
Trizygia	294.32	272.95	311.63	249.17	241.68	255.64
Yavanna	136.98	112.11	192.36	101.83	65.81	124.91
Yulebacaulis	142.60	100.23	205.12	107.04	65.22	143.72

Supplementary Table 2. Molecular age estimates.

Clade	stem age (Ma)	95% HPD	crown age (Ma)	95% HPD
MONILOPHYTES	n.a.	n.a.	421.3	379.16-465.97
EQUISETIDAE	421.3	379.16-465.97	80.1	50.87-113.95
EQUISETALES	421.3	379.16-465.97	80.1	50.87-113.95
**Equisetaceae**	421.3	379.16-465.97	80.1	50.87-113.95
Equisetum	421.3	379.16-465.97	80.1	50.87-113.95
OPHIOGLOSSIDAE	358.29	318.00-401.48	244.2	186.64-306.29
OPHIOGLOSSALES	244.2	186.64-306.29	148.94	108.17-187.78
**Ophioglossaceae**	244.2	186.64-306.29	148.94	108.17-187.78
Botrychium	29.63	14.51-48.37	16.1	6.00-28.52
Botrypus	54.61	27.25-86.01	22.44	14.89-45.08
Cheiroglossa	not included			
Helminthostachys	119.79	84.78-159.10	n.a.	n.a.
Japanobotrychium	not included			
Mankyua	not included			
Ophioderma	69.8	41.38-101.05	n.a.	n.a.
Ophioglossum	69.8	41.38-101.05	n.a.	n.a.
Rhizoglossum	not included			
PSILOTALES	244.2	186.64-306.29	55.82	123.40-91.76
**Psilotaceae**	244.2	186.64-306.29	55.82	123.40-91.76
Psilotum	55.82	23.40-91.76	n.a.	n.a.

Tmesipteris	55.82	23.40-91.76	n.a.	n.a.
MARATTIIDAE	358.29	318.00-401.48	202.71	185.29-224.20
MARATTIALES	358.29	318.00-401.48	202.71	185.29-224.20
**Marattiaceae**	358.29	318.00-401.48	202.71	185.29-224.20
Angiopteris	182.69	172.00-198.04	1.78	0.06-4.69
Christensenia	202.71	185.29-224.20	n.a.	n.a.
Danaea	188.8	177.71-203.20	30.43	17.29-45.96
Eupodium	181.39	176.19-191.92	n.a.	n.a.
Marattia	182.69	172.00-198.04	n.a.	n.a.
Ptisana	181.39	176.00-191.92	17.91	8.06-29.84
POLYPODIIDAE	414.34	374.15-456.27	377.19	339.23-414.21
OSMUNDALES	377.19	339.23-414.21	241.26	217.19-270.38
**Osmundaceae**	377.19	339.23-414.21	241.26	217.19-270.38
Claytosmunda	embedded in *Osmunda*			
Leptopteris	155.27	136.00-191.72	12.1	2.44-26.16
Osmunda	223.92	208.04-243.87	206.75	201.60-217.65
Osmundastrum	241.26	217.19-270.38	12	1.12-30.70
Plenasium	embedded in *Osmunda*			
Todea	155.27	136.00-191.72	n.a.	n.a.
HYMENOPHYLLALES	315.27	282.11-348.57	216.09	175.76-255.40
**Hymenophyllaceae**	315.27	282.11-348.57	216.09	175.76-255.40
TRICHOMANOIDEAE	216.09	175.76-255.40	175.15	142.88-206.42
Abrodictyum	97.82	58.14-139.30	56.71	27.70-93.68
Callistopteris	128.45	84.72-169.36	n.a.	n.a.
Cephalomanes	97.82	58.14-139.30	n.a.	n.a.
Crepidomanes	130.04	103.42-157.17	88.5	63.90-114.91
Didymoglossum	140.78	114.95-167.68	103.56	79.33-127.81
Polyphlebium	151.65	123.66-178.90	62.46	30.86-98.15
Trichomanes	152.15	118.79-185.83	99.47	73.34-128.01
Vandenboschia	130.04	103.42-157.17	27.11	5.77-55.76
HYMENOPHYLLOIDEAE	216.09	175.76-255.40	47.37	35.92-60.01
Hymenophyllum	216.09	175.76-255.40	47.37	35.92-60.01
GLEICHENIALES	280.44	261.59-320.01	134.09	110.23-161.65
**Gleicheniaceae**	280.44	261.59-320.01	134.09	110.23-161.65
Dicranopteris	32.42	14.24-54.56	n.a.	n.a.
Diplopterygium	91.74	55.83-127.57	28.83	12.00-50.02
Gleichenella	32.42	14.24-54.56	n.a.	n.a.
Gleichenia	95.45	89.30-107.47	n.a.	n.a.
Sticherus	110.35	95.11-129.08	76.99	37.80-110.62
Stromatopteris	95.45	89.30-107.47	n.a.	n.a.

**Dipteridaceae**	242.48	235.00-256.62	50.38	22.57-87.25
Cheiropleuria	50.38	22.57-87.25	n.a.	n.a.
Dipteris	50.38	22.57-87.25	n.a.	n.a.
**Matoniaceae**	242.48	235.00-256.62	96.6	49.83-149.11
Matonia	96.6	49.83-149.11	n.a.	n.a.
Phanerosorus	96.6	49.83-149.11	n.a.	n.a.
SCHIZAEALES	322.79	290.55-354.72	250.76	213.60-287.39
**Lygodiaceae**	250.76	213.60-287.39	45.58	23.31-72.61
Lygodium	250.76	213.60-287.39	45.58	23.31-72.61
**Schizaeaceae**	190.36	161.39-222.54	91.32	51.37-133.42
Actinostachys	91.32	51.37-133.42	n.a.	n.a.
Schizaea	91.32	51.37-133.42	24.01	15.59-48.87
**Anemiaceae**	190.36	161.39-222.54	145.26	136.00-162.15
Anemia	190.36	161.39-222.54	145.26	136.00-162.15
SALVINIALES	290.3	260.52-320.25	195.55	155.00-235.65
**Marsileaceae**	195.55	155.00-235.65	109.29	99.60-125.22
Marsilea	109.29	99.60-125.22	31.5	18.17-46.60
Pilularia	90.26	83.50-101.25	9.44	2.39-20.26
Regnellidium	90.26	83.50-101.25	n.a.	n.a.
**Salviniaceae**	195.55	155.00-235.65	105.13	83.50-130.34
Azolla	105.13	83.50-130.34	52.39	34.91-70.97
Salvinia	105.13	83.50-130.34	49.73	29.85-71.40
CYATHEALES	279	249.60-307.72	150.76	133.22-171.67
**Thyrsopteridaceae**	140.81	123.85-160.72	n.a.	n.a.
Thyrsopteris	140.81	123.85-160.72	n.a.	n.a.
**Loxsomataceae**	121.24	112.00-137.26	23.5	8.66-42.22
Loxsoma	23.5	8.66-42.22	n.a.	n.a.
Loxsomopsis	23.5	8.66-42.22	n.a.	n.a.
**Culcitaceae**	62.89	34.77-95.55	14.54	4.21-28.85
Culcita	62.89	34.77-95.55	14.54	4.21-28.85
**Plagiogyriaceae**	62.89	34.77-95.55	31.89	15.99-50.50
Plagiogyria	62.89	34.77-95.55	31.89	15.99-50.50
**Cibotiaceae**	128.9	120.34-140.29	17.48	8.67-28.21
Cibotium	128.9	120.34-140.29	17.48	8.67-28.21
**Cyatheaceae**	132.06	125.00-142.80	30.28	21.65-41.12
Alsophila	24.18	17.38-31.72	16.29	10.91-22.26
Cyathea	24.18	17.38-31.72	14.43	9.63-19.49
Gymnosphaera	26.47	19.07-34.80	16.61	9.30-24.25
Sphaeropteris	30.28	21.65-41.12	27.15	17.69-37.01
**Dicksoniaceae**	127.43	118.31-138.30	118.77	112.72-126.46

Calochlaena	118.77	112.72-126.46	26.66	9.83-50.65
Dicksonia	115.94	112.00-122.86	10.1	2.95-20.13
Lophosoria	115.94	112.00-122.86	n.a.	n.a.
**Metaxyaceae**	127.43	118.31-138.30	n.a.	n.a.
Metaxya	127.43	118.31-138.30	n.a.	n.a.
POLYPODIALES	279	249.60-307.72	248.81	221.72-273.80
**Lonchitidaceae**	182.43	155.69-211.48	n.a.	n.a.
Lonchitis	182.43	155.69-211.48	n.a.	n.a.
**Saccolomataceae**	235.21	208.09-262.00	15.16	5.77-27.39
Saccoloma	235.21	208.09-262.00	15.16	5.77-27.39
**Cystodiaceae**	221.07	194.15-248.43	n.a.	n.a.
Cystodium	221.07	194.15-248.43	n.a.	n.a.
**Lindsaeaceae**	182.43	155.69-211.48	127.49	106.54-150.87
Lindsaea	65.51	52.99-79.27	44.22	32.28-56.23
Nesolindsaea	40.98	29.24-52.95	n.a.	n.a.
Odontosoria Old World	127.49	106.54-150.87	15.76	3.55-35.78
Odontosoria New World	79.91	64.93-95.64	n.a.	n.a.
Osmolindsaea	48.57	36.61-61.18	10.44	2.73-20.53
Sphenomeris	71.25	57.63-86.04	n.a.	n.a.
Tapeinidium	40.98	29.24-52.95	20.32	11.50-29.69
Xyropteris	not included			
**Pteridaceae**	223.92	199.06-247.93	177.61	155.14-200.82
CRYPTOGRAMMOIDEAE	161.03	136.24-186.88	79.99	49.15-115.07
Coniogramme	58.7	33.00-88.65	21.72	7.73-38.82
Cryptogramma	58.7	33.00-88.65	n.a.	n.a.
Llavea	79.99	49.15-115.07	n.a.	n.a.
PARKERIOIDEAE	161.03	136.24-186.88	144.2	119.48-169.89
Acrostichum	80.44	56.56-106.23	38.85	18.04-61.85
Ceratopteris	80.44	56.56-106.23	2.07	0.01-6.10
PTERIDOIDEAE	144.2	119.48-169.89	103.34	82.40-123.89
Actiniopteris	41.39	17.48-70.26	n.a.	n.a.
Anogramma	17.8	3.28-37.25	n.a.	n.a.
Austrogramme	not included			
Cerosora	not included			
Cosentinia	not included			
Jamesonia	57.67	42.02-73.47	25.86	14.69-38.39
Onychium	41.39	17.48-70.26	n.a.	n.a.
Pityrogramma	40.46	23.01-58.89	8.83	1.83-18.71
Pteris	103.34	82.40-123.89	89.01	61.35-114.47
Pterozonium	29.94	11.97-50.65	n.a.	n.a.

Syngramma	not included			
Taenitis	not included			
Tryonia	57.37	11.97-50.65	n.a.	n.a.
CHEILANTHOIDEAE	156.11	135.56-178.76	135.21	102.05-166.43
Adiantopsis	31.99	23.36-40.92	n.a.	n.a
Aleuritopteris	35	21.52-48.05	25.71	12.22-39.18
Allosorus	35	21.52-48.05	n.a.	n.a.
Argyrochosma	48.06	34.35-62.37	29.35	15.31-44.23
Aspidotis	32.37	19.99-44.96	14.71	4.86-26.22
Astrolepis	not included			
Bommeria	93.74	71.05-119.26	26.98	7.24-53.85
Calciphilopteris	135.21	102.05-166.43	n.a.	n.a.
Cheilanthes	not monophyletic			
Cheiloplecton	33.88	23.37-45.22	n.a.	n.a.
Doryopteris	24.88	16.36-33.81	12.33	5.58-19.92
Gaga	32.37	19.99-44.96	n.a.	n.a.
Hemionitis	23.07	13.35-32.59	n.a.	n.a.
Lytoneuron	not included			
Mildella	52.09	36.00-68.56	n.a.	n.a.
Myriopteris	52.09	36.00-68.56	37.9	25.15-51.68
Notholaena	not monophyletic			
Ormopteris	24.88	16.36-33.81	n.a.	n.a.
Paragymnopteris	embedded in *Pellaea*			
Parahemionitis	23.07	13.35-32.59	n.a.	n.a.
Pellaea	48.06	34.35-62.37	36.43	25.24-49.03
Pentagramma	48.79	38.11-60.28	n.a.	n.a.
Trachypteris	not included			
VITTARIOIDEAE	156.11	135.56-178.76	141.16	121.06-161.49
Adiantum	141.16	121.06-161.49	115.94	87.40-143.20
Ananthacorus	not included			
Antrophyum	78.53	63.03-94.54	n.a.	n.a.
Haplopteris	67.61	51.84-85.58	43.23	28.36-58.41
Hecistopteris	44.58	28.92-60.78	n.a.	n.a.
Polytaenium	56.25	41.73-70.48	34.31	21.63-47.95
Radiovittaria	44.58	28.92-60.78	n.a.	n.a.
Rheopteris	103.51	85.63-122.69	n.a.	n.a.
Scoliosorus	not included			
Vaginularia	not included			
Vittaria	56.25	41.73-70.48	23.71	10.38-37.92
**Dennstaedtiaceae**	213.89	189.42-237.99	125.06	93.62-160.85

Blotiella	37.57	20.76-55.51	n.a.	n.a.
Dennstaedtia	56.08	37.47-74.75	28.86	11.85-47.75
Histiopteris	37.57	20.76-55.51	n.a.	n.a.
Hypolepis	56	36.87-77.27	n.a.	n.a.
Leptolepia	83.55	70.60-104.22	30.25	11.24-53.26
Microlepia	56.08	37.47-74.75	16.36	8.30-26.14
Monachosorum	102.19	70.61-136.78	n.a.	n.a.
Oenotrichia	not included			
Paesia	50.38	31.78-71.75	n.a.	n.a.
Pteridium	61.69	40.42-84.38	11.33	3.91-20.91
**Cystopteridaceae**	91.53	53.17-133.49	62.29	35.35-93.75
Acystopteris	34.72	16.16-57.57	12.89	1.26-30.67
Cystopteris	34.72	16.16-57.57	n.a.	n.a.
Gymnocarpium	62.29	35.35-93.75	18.36	4.49-36.92
**Hemidictyaceae**	137.04	114.28-161.35	n.a.	n.a.
Hemidictyum	137.04	114.28-161.35	n.a.	n.a.
**Aspleniaceae**	137.04	114.28-161.35	104.15	85.96-123.03
Asplenium	104.15	85.96-123.03	82.59	68.93-97.47
Hymenasplenium	104.15	85.96-123.03	56.09	34.04-80.00
**Diplaziopsidaceae**	91.53	53.17-133.49	40.34	17.19-70.02
Diplaziopsis	40.34	17.19-70.02	n.a.	n.a.
Homalosorus	40.34	17.19-70.02	n.a.	n.a.
**Desmophlebiaceae**	not included			
Desmophlebium	not included			
**Thelypteridaceae**	119.5	96.77-143.87	89.91	70.73-110.68
PHEGOPTERIDOIDEAE	89.91	70.43-110.68	59.96	34.57-85.89
Macrothelypteris	59.96	34.57-85.89	n.a.	n.a.
Phegopteris	29.7	16.50-45.38	16.51	6.62-28.10
Pseudophegopteris	29.7	16.50-45.38	15.41	3.64-29.79
THELYPTERIDOIDEAE	89.91	70.43-110.68	77.4	60.94-94.55
Amauropelta	embedded in *Thelypteris*			
Amblovenatum	embedded in *Thelypteris*			
Ampelopteris	not included			
Chingia	embedded in *Thelypteris*			
Christella	embedded in *Thelypteris*			
Coryphopteris	embedded in *Thelypteris*			
Cyclogramma	not included			
Cyclosorus	embedded in *Thelypteris*			
Glaphyropteridopsis	embedded in *Thelypteris*			
Goniopteris	embedded in *Thelypteris*			

Meniscium	embedded in *Thelypteris*			
Menisorus	not included			
Mesophlebion	not included			
Mesopteris	not included			
Metathelypteris	embedded in *Thelypteris*			
Nannothelypteris	not included			
Oreopteris	embedded in *Thelypteris*			
Parathelypteris	not included			
Plesioneuron	not included			
Pneumatopteris	embedded in *Thelypteris*			
Pronephrium	embedded in *Thelypteris*			
Pseudocyclosorus	embedded in *Thelypteris*			
Sphaerostephanos	embedded in *Thelypteris*			
Stenogramma	embedded in *Thelypteris*			
Steiropteris	embedded in *Thelypteris*			
Thelypteris	89.91	70.43-110.68	77.4	60.94-94.55
Trigonospora	embedded in *Thelypteris*			
**Woodsiaceae**	110.9	90.92-132.19	62.55	38.33-89.01
Woodsia	110.9	90.92-132.19	62.55	38.33-89.01
**Rhachidosoraceae**	129.76	102.96-158.67	n.a.	n.a.
Rhachidosorus	129.76	102.96-158.67	n.a.	n.a.
**Onocleaceae**	91.75	76.40-107.94	66.46	55.80-79.43
Onoclea	91.75	76.40-107.94	66.46	55.80-79.43
Onocleopsis	42.24	16.84-63.74	n.a.	n.a.
Matteuccia	42.24	16.84-63.74	n.a.	n.a.
**Blechnaceae**	91.75	76.40-107.94	71.62	58.81-84.97
STENOCHLAENOIDEAE	67.84	54.58-80.61	53.34	38.72-68.22
Salpichlaena	53.34	38.72-68.22	13.1	4.09-21.51
Stenochlaena	41.85	25.66-58.02	2.45	0.00-9.30
Telmatoblechnum	41.85	25.66-58.02	n.a.	n.a.
WOODWARDIOIDEAE	71.62	58.81-84.97	46.72	29.81-63.02
Anchistea	embedded in *Woodwardia*			
Lorinseria	embedded in *Woodwardia*			
Woodwardia	71.62	58.81-84.97	46.72	29.81-63.02
BLECHNOIDEAE	67.84	54.58-80.61	57.93	44.85-71.66
Austroblechnum	embedded in *Blechnum*			
Blechnidium	embedded in *Blechnum*			
Blechnopsis	embedded in *Blechnum*			
Blechnum	67.84	54.58-80.61	57.93	44.85-71.66
Brainea	embedded in *Blechnum*			

Cleistoblechnum	embedded in *Blechnum*			
Cranfilia	embedded in *Blechnum*			
Diploblechnum	embedded in *Blechnum*			
Doodia	embedded in *Blechnum*			
Icarus	embedded in *Blechnum*			
Lomaria	embedded in *Blechnum*			
Lomaridium	embedded in *Blechnum*			
Lomariocycas	embedded in *Blechnum*			
Neoblechnum	embedded in *Blechnum*			
Oceaniopteris	embedded in *Blechnum*			
Parablechnum	embedded in *Blechnum*			
Sadleria	embedded in *Blechnum*			
Struthiopteris	embedded in *Blechnum*			
**Athyriaceae**	105.23	87.24-124.38	88.85	67.04-111.17
Athyrium	74.29	54.43-95.29	43.13	24.77-64.09
Deparia	88.85	67.04-111.17	27.5	11.64-46.84
Diplazium	74.29	54.43-95.29	48.17	33.27-65.02
**Didymochlaenaceae**	144.01	99.99-175.90	n.a.	n.a.
Didymochlaena	144.01	99.99-175.90	n.a.	n.a.
**Hypodematiaceae**	144.01	99.99-175.90	72.68	30.37-123.07
Hypodematium	72.68	30.37-123.07	n.a.	n.a.
Leucostegia	72.68	30.37-123.07	15.67	3.87-33.46
**Dryopteridaceae**	152.29	131.84-172.99	122.02	99.29-144.76
Aenigmopteris	not included			
Dryopolystichum	not included			
DRYOPTERIDOIDEAE	111.58	86.61-135.14	98.81	75.55-122.53
Arachniodes	77.2	56.74-97.75	50.99	35.80-67.38
Ctenitis	76.9	56.42-99.00	37.49	22.18-54.19
Cyrtomium	39.56	29.09-50.57	15.66	6.95-25.23
Dryopteris	77.2	56.74-97.75	45.82	33.49-59.02
Phanerophlebia	48.63	35.48-63.12	n.a.	n.a.
Polystichum	39.56	29.09-50.57	34.95	25.49-44.86
POLYBOTRYOIDEA	111.58	86.61-135.14	95.57	62.98-127.00
Cyclodium	embedded in *Polybotrya*			
Maxonia	31.83	19.62-46.00	n.a.	n.a.
Olfersia	embedded in *Polybotrya*			
Polybotrya	31.83	19.62-46.00	27.73	17.26-39.57
Polystichopsis	73.34	43.70-105.09	n.a.	n.a.
Stigmatopteris	95.57	62.98-127.00	14.58	4.38-29.09
Trichoneuron	not included			

ELAPHOGLOSSOIDEAE	122.02	99.29-144.76	98.53	78.53-119.71
Arthrobotrya	not included			
Bolbitis	70	54.80-85.44	42.82	24.93-61.84
Elaphoglossum	58.83	45.95-72.27	46.67	35.72-58.67
Lastreopsis	49.08	25.96-73.53	32.27	11.60-56.05
Lomagramma	40.74	19.56-61.27	n.a.	n.a.
Megalastrum	36.81	17.28-59.41	n.a.	n.a.
Mickelia	58.83	45.95-72.27	45.34	29.16-61.80
Parapolystichum	80.18	48.75-108.96	23.38	7.50-45.52
Pleocnemia	91.02	72.02-111.37	n.a.	n.a.
Rumohra	36.81	17.28-59.41	n.a.	n.a.
Teratophyllum	40.74	19.56-61.27	n.a.	n.a.
**Lomariopsidaceae**	134.71	114.80-155.38	99.13	73.79-124.14
Cyclopeltis	99.13	73.79-124.14	26.39	8.92-50.50
Dracoglossum	78.4	55.19-102.75	n.a.	n.a.
Lomariopsis	78.4	55.19-102.75	29.03	17.45-41.59
Thysanosoria	not included			
**Nephrolepidaceae**	134.71	114.80-155.38	61.62	32.40-95.12
Nephrolepis	134.71	114.80-155.38	61.62	32.40-95.12
**Tectariaceae**	119.39	103.22-135.80	97.99	69.33-122.48
Arthropteris	97.99	69.33-122.48	39.17	15.68-67.16
Draconopteris	not included			
Hypoderris	not included			
Malaifilix	not included			
Pteridrys	83.43	58.37-108.82	n.a.	n.a.
Tectaria	66.78	46.21-89.61	44.48	31.31-60.06
Triplophyllum	66.78	46.21-89.61	n.a.	n.a.
**Oleandraceae**	114.36	98.64-129.78	32.05	5.63-80.22
Oleandra	114.36	98.64-129.78	32.05	5.63-80.22
**Davalliaceae**	108.91	94.53-123.99	63.11	41.19-88.53
Davallia	108.91	94.53-123.99	63.11	41.19-88.53
**Polypodiaceae**	108.91	94.53-123.99	101.47	87.48-114.93
LOXOGRAMMOIDEAE	101.47	87.48-114.93	73.77	55.16-91.40
Dictymia	73.77	55.16-91.40	27.43	8.73-50.81
Loxogramme	73.77	55.16-91.40	45.55	29.41-63.48
DRYNARIOIDEAE	90.1	77.99-102.13	67.17	39.70-93.39
Aglaomorpha	59.65	37.55-86.72	40.17	25.30-57.70
Arthromeris	28.11	17.42-39.52	12.07	2.97-23.37
Gymnogrammitis	embedded in *Selliguea*			
Paraselliguea	not included			

Phymatopteris	not included			
Polypodiopteris	embedded in *Selliguea*			
Selliguea	28.11	17.42-39.52	25.96	16.27-36.49
PLATYCERIOIDEAE	84.17	72.04-96.46	71.5	58.82-84.72
Platycerium	71.5	58.82-84.72	46.68	35.46-61.96
Pyrrosia	71.5	58.82-84.72	49.04	35.86-62.84
MICROSOROIDEAE	84.17	72.04-96.46	72.8	60.90-85.12
Dendroconche	not included			
Goniophlebium	66.26	54.85-77.38	43.18	26.75-59.79
Kaulinia	not included			
Kontumia	not included			
Lecanopteris	embedded in *Microsorum*			
Lemmaphyllum	32.23	22.83-41.64	17.55	8.56-27.34
Lepidomicrosorium	17.76	9.59-26.80	10.85	4.76-17.69
Lepisorus	42.48	33.41-51.66	37.92	28.61-47.18
Leptochilus	37.47	27.51-48.14	30.43	21.33-39.76
Microsorum	66.26	54.85-77.38	64.25	53.52-75.21
Neocheiropteris	17.76	9.59-26.80	n.a.	n.a.
Neolepisorus	27.64	18.86-36.80	19.71	11.23-29.08
Paragramma	46.4	5.69-21.29	n.a.	n.a.
Podosorus	not included			
Thylacopteris	72.8	60.90-85.12	n.a.	n.a.
Tricholepidium	22.67	13.51-32.20	n.a.	n.a.
POLYPODIOIDEAE	89.49	77.57-101.49	87.08	75.59-98.92
Acrosorus	not included			
Adenophorus	45.35	34.19-57.09	31.38	20.92-43.10
Alansmia	49.18	36.23-61.96	22.39	9.46-36.99
Archigrammitis	not included			
Ascogrammitis	52.05	43.68-60.77	35.52	24.10-47.22
Calymmodon	39.4	29.36-49.51	23.36	12.35-35.16
Campyloneurum	68.11	47.82-86.77	37.55	20.87-57.80
Ceradenia	42.27	29.73-54.62	32.35	20.86-44.57
Chrysogrammitis	47.29	37.25-57.04	n.a.	n.a.
Cochlidium	36.37	25.25-48.10	25.4	15.34-36.66
Ctenopterella	not included			
Ctenopteris	not monophyletic			
Dasygrammitis	not included			
Enterosora	not monophyletic			
Galactodenia	42.77	34.52-50.98	n.a.	n.a.
Grammitis	36.37	25.25-48.10	28.03	15.89-40.49

Lellingeria	38.02	30.26-45.64	32.57	24.91-40.58
Leucostrichum	49.18	36.23-61.96	31.11	18.85-44.47
Lomaphlebia	not included			
Luisma	not included			
Melpomene	36.15	28.14-43.80	26.95	15.53-37.78
Microgramma	77.37	60.78-92.21	45.68	30.42-62.98
Micropolypodium	not included			
Moranopteris	47.29	37.25-57.04	25.7	15.93-36.35
Mycopteris	45.03	36.66-53.27	30.06	18.11-42.49
Niphidium	68.11	47.82-86.77	n.a.	n.a.
Notogrammitis	32.39	18.76-40.54	21.53	11.84-32.06
Oreogrammitis	not monophyletic			
Pecluma	62.91	44.95-80.11	43.26	29.29-58.73
Phlebodium	62.91	44.95-80.11	18.69	3.77-41.45
Pleopeltis	72.88	53.68-88.90	35.65	24.37-49.00
Pleurosoriopsis	49.08	27.17-75.18	n.a.	n.a.
Polypodium	49.08	27.17-75.18	36.47	20.41-54.17
Prosaptia	20.86	12.92-29.40	9.99	4.81-15.90
Radiogrammitis	not monophyletic			
Scleroglossum	31.78	19.91-43.99	n.a.	n.a.
Serpocaulon	69.32	47.88-87.53	44.5	29.26-60.95
Stenogrammitis	36.15	28.14-43.80	24	16.59-31.69
Synammia	67.17	39.70-93.39	10.55	1.28-24.98
Terpsichore	67.77	58.32-77.15	37	19.86-54.17
Themelium	16.07	5.33-28.40	n.a.	n.a.
Tomophyllum	embedded in *Grammitis*			
Unnamed genus[1]	69.32	47.88-87.53	n.a.	n.a.
Xiphopterella	not included			
Zygophlebia	not included			

Ma=millions of years
HPD=highest porterior density
[1]*Polypodium chrysolepis* Hook. is not resolved as a member of any named genus, but as a sister lineage to *Serpocaulon.*

Supplementary Table 3. Minimum divergence incongruence metrics.

Taxon	Fossil estimate 95% CI		Molecular min-max estimate		MDI
Acrostichum	67.84	75.78	18.04	106.23	0
Adiantum	114.15	139.20	87.40	161.49	0
Alsophila	2.64	53.15	10.91	31.72	0

Anemia	131.13	146.54	136.00	222.54	0
Angiopteris	208.94	247.17	0.06	198.04	-10.9
Anogramma	2.65	15.90	<3.28	37.25	0
Antrophyum	19.82	35.64	<63.03	94.54	n.a.
Arthropteris	1.83	42.68	15.68	122.48	0
Asplenium	228.85	242.56	68.93	123.03	-105.82
Athyrium	127.85	199.84	24.77	95.29	-32.56
Azolla	99.47	145.32	34.91	130.34	0
Blechnum	141.00	163.04	44.85	80.61	-60.39
Botrychium	57.87	110.55	27.25	187.78	0
Ceratopteris	34.59	94.73	0.01	106.23	0
Cibotium	66.95	143.19	8.67	140.29	0
Coniogramme	5.56	83.58	7.73	88.65	0
Ctenitis	2.70	23.17	22.18	99.00	0
Cyathea	203.47	239.62	9.63	31.72	-171.75
Danaea	35.45	128.24	17.29	203.20	0
Davallia	37.12	72.57	26.26	53.00	0
Dennstaedtia	161.69	190.77	11.85	74.75	-86.94
Deparia	5.36	47.31	11.64	111.17	0
Dicksonia	192.10	215.19	2.95	122.86	-69.24
Dicranopteris	81.37	97.97	<14.24	54.56	-26.81
Diplazium	34.81	134.93	33.27	95.29	0
Drynaria	6.26	25.78	25.3	86.72	0
Dryopteris	164.55	180.63	33.49	97.75	-66.8
Elaphoglossum	15.51	88.12	35.72	72.27	0
Equisetum	324.72	359.39	50.87	465.97	0
Gleichenia	174.42	182.67	<89.30	107.47	-66.95
Grammitis	15.32	39.13	15.89	48.10	0
Hemionitis	57.77	123.27	13.35	45.17	-12.6
Histiopteris	28.79	46.81	<20.76	55.51	0
Hymenophyllum	85.95	106.37	35.92	255.40	0
Hypolepis	16.15	33.99	<36.87	77.27	0
Lastreopsis	2.64	54.64	11.6	73.53	0
Lepisorus	28.70	52.09	28.61	51.66	0
Leptolepia	0.06	36.88	11.24	104.22	0
Leptopteris	28.78	47.36	2.44	191.72	0
Lindsaea	34.07	50.81	32.28	79.27	0
Lophosoria	112.7	222.62	<112.00	122.86	0
Lygodium	81.89	144.86	23.31	287.39	0
Marattia	214.15	242.05	<172.00	198.04	-16.11

Marsilea	178.21	210.06	18.17	125.22	-52.99
Matonia	179.88	242.22	<49.83	149.11	-30.77
Microsorum	37.70	57.46	53.52	77.38	0
Nephrolepis	5.39	17.68	32.40	155.38	14.72
Oleandra	119.57	152.44	5.63	129.78	0
Onoclea	94.05	141.68	55.8	107.94	0
Ophioglossum	16.41	36.39	41.38	159.10	4.99
Osmunda	203.94	236.93	201.60	243.87	0
Osmundastrum	40.67	133.15	1.12	270.38	0
Paesia	34.17	50.31	<31.78	71.75	0
Pellaea	1.81	40.94	25.24	62.37	0
Pilularia	66.22	123.21	2.39	101.25	0
Pleopeltis	16.11	91.51	24.37	88.90	0
Polypodium	34.18	72.69	20.41	75.18	0
Polystichum	65.63	108.17	35.48	99.00	0
Psilotum	16.09	87.40	<23.40	91.76	0
Pteridium	33.98	40.91	3.91	84.38	0
Pteris	95.61	104.76	61.35	123.89	0
Pyrrosia	3.64	9.20	35.86	84.72	26.66
Regnellidium	83.83	142.19	<83.50	101.25	0
Rumohra	34.46	66.21	<17.28	59.41	0
Saccoloma	94.76	114.88	5.77	262.00	0
Salpichlaena	74.39	84.41	4.09	68.22	-6.17
Salvinia	69.34	98.97	29.85	130.34	0
Schizaea	56.05	75.90	15.59	133.42	0
Sticherus	66.10	117.16	37.80	129.08	0
Tectaria	123.91	151.61	31.31	89.61	-34.3
Thelypteris	66.42	85.21	60.94	110.68	0
Thyrsopteris	304.41	372.07	<123.85	160.72	-143.69
Tmesipteris	28.57	51.13	<23.40	91.76	0
Todea	172.31	235.79	<136.00	191.72	0
Trichomanes	66.77	116.20	73.34	185.83	0
Woodwardia	97.58	145.22	29.81	84.97	-12.61

Origination and extinction times as estimated from fossil record with PyRate compared with molecular estimates (min=upper 95% CI estimate of the crown group, max=lower 95% CI estimate of the stem group) and their MDI incogruence metric.

Supplementary Table 4. Baseline origination and extinction rates (λ_0 and μ_0) and correlation parameters.

Parameter		Linear model			Exponential model		
		Mean	95% HPD		Mean	95% HPD	
**Baseline rates**	λ_0	0.019	0.001	0.043	0.103	0.005	0.276
	μ_0	0.015	0.002	0.032	0.014	0	0.053
**Correlation**	G_0	-1.759	-5.264	0.714	-2.414	-4.35	0.041
**parameters**	G_1	-0.057	-1.62	1.299	0.012	-1.008	1.002
**(origination)**	G_2	-0.609	-2.856	1.05	0.143	-0.467	0.891
	G_3	0.919	-0.789	3.816	-0.084	-1.102	0.825
	G_4	-0.986	-4.603	0.982	-0.41	-1.648	0.47
	G_5	2.005	-1.1	8.799	1.822	-0.053	3.635
	G_6	-0.653	-3.428	0.931	0.549	-0.575	2.237
	G_7	0.299	-1.001	2.055	-0.646	-1.969	0.364
	G_8	1.468	-0.919	5.006	0.248	-0.548	1.384
	G_9	0.656	-0.992	3.029	0.068	-0.896	1.149
	G_10	-0.048	-1.829	1.804	-0.568	-1.843	0.39
	G_11	0.257	-1.59	2.54	0.007	-1.334	1.359
	G_12	-0.201	-1.803	1.102	-0.814	-2.663	0.458
	G_13	-0.202	-1.969	1.146	0.28	-0.771	1.722
	G_14	3.855	-1.061	15.837	-0.398	-1.605	0.523
	G_15	1.583	-1.038	7.377	-0.141	-1.69	1.032
	G_16	0.419	-0.708	2.203	0.809	-0.383	2.392
**Correlation**	H_0	1.29	-0.91	5.709	1.174	-0.531	3.661
**parameters**	H_1	-0.536	-2.307	0.6	-0.379	-2.111	0.779
**(extinction)**	H_2	-0.038	-1.22	1.025	-1.088	-2.391	0.133
	H_3	-0.399	-2.443	1.062	-0.025	-1.145	1.055
	H_4	-0.221	-1.769	1.145	-1.365	-3.253	0.265
	H_5	0.5	-1.363	2.936	1.535	-0.268	3.474
	H_6	-4.193	-10.495	-0.592	1.586	-0.228	3.283
	H_7	-1.02	-3.506	0.628	0.282	-0.775	1.738
	H_8	2.365	-0.535	6.292	0.216	-0.984	1.779
	H_9	2.15	-0.226	6.422	0.605	-0.624	2.305
	H_10	-1.234	-4.776	0.588	-1.004	-2.572	0.187
	H_11	0.458	-0.609	2.179	2.739	0.007	4.859
	H_12	0.316	-1.4	2.468	-3.307	-5.895	-0.814
	H_13	-1.407	-3.834	0.255	0.194	-1.286	2.005
	H_14	-0.682	-2.533	0.775	0.822	-0.528	2.95

H_15	3.123	-0.302	9.244	-0.702	-2.818	0.617
H_16	0.472	-0.937	2.29	-0.304	-1.898	0.917

The curves were numbered as follows: 0 fern diversity; 1 selenium concentration in marine sediments; 2 wet tropical biome extent; 3 cool temperate biome extent; 4 magmatic activity; 5 athmospheric CO2; 6 gymnosperm diversity; 7 angiosperm diversity; 8 warm temperate biome extent; 9 polar biome extent; 10 continental fragmentation; 11 arid biome extent; 12 athmospheric O2; 13 eustatic sea level; 14 diversity of lycophytes etc. (free sporing vascular plants excluding ferns); 15 global mean temperature; 16 mountain area.

Supplementary Table 3. Shrinkage weights, based on local and global shrinkage parameters, and global shrinkage.

		**Linear model**			**Exponential model**		
**Parameter**		**Mean**	**95% HPD**		**Mean**	**95% HPD**	
**Shrinkage weights**	w(G_0)	**0.618**	0.035	1	**0.762**	0.299	1
**(origination)**	w(G_1)	0.289	0	0.923	0.269	0	0.864
	w(G_2)	0.378	0	0.957	0.252	0	0.842
	w(G_3)	0.452	0	0.971	0.28	0	0.881
	w(G_4)	0.412	0	0.976	0.332	0	0.899
	w(G_5)	**0.515**	0	0.992	**0.68**	0.175	1
	w(G_6)	0.382	0	0.962	0.375	0	0.938
	w(G_7)	0.295	0	0.93	0.416	0	0.931
	w(G_8)	**0.57**	0.026	1	0.297	0	0.877
	w(G_9)	0.4	0	0.959	0.286	0	0.879
	w(G_10)	0.306	0	0.934	0.392	0	0.927
	w(G_11)	0.33	0	0.95	0.31	0	0.898
	w(G_12)	0.302	0	0.927	0.454	0	0.952
	w(G_13)	0.309	0	0.931	0.319	0	0.897
	w(G_14)	**0.607**	0.008	1	0.326	0	0.91
	w(G_15)	0.456	0	0.988	0.313	0	0.905
	w(G_16)	0.326	0	0.942	0.446	0	0.95
**Shrinkage weights**	w(H_0)	0.456	0	0.983	**0.518**	0	0.972
**(extinction)**	w(H_1)	0.353	0	0.946	0.351	0	0.923
	w(H_2)	0.282	0	0.91	**0.537**	0	0.961
	w(H_3)	0.348	0	0.946	0.284	0	0.875
	w(H_4)	0.319	0	0.927	0.577	0	0.973
	w(H_5)	0.375	0	0.958	0.611	0.033	1
	w(H_6)	**0.834**	0.427	1	**0.633**	0.039	1

	w(H_7)	0.477	0	0.97	0.315	0	0.903
	w(H_8)	0.69	0.102	1	0.322	0	0.905
	w(H_9)	0.636	0.064	1	0.395	0	0.944
	w(H_10)	0.488	0	0.981	**0.511**	0	0.961
	w(H_11)	0.336	0	0.938	**0.782**	0.25	1
	w(H_12)	0.364	0	0.943	0.84	0.487	1
	w(H_13)	**0.563**	0.044	1	0.337	0	0.921
	w(H_14)	0.426	0	0.954	0.448	0	0.959
	w(H_15)	**0.694**	0.053	1	0.426	0	0.947
	w(H_16)	0.352	0	0.947	0.346	0	0.92
**Global shrinkage**	τ	0.999	0.138	2.48	0.839	0.306	1.491

Shrinkage weights greater than 0.5 (highlighted in bold) indicate significant evidence for correlation (positive or negative depending on the respective G or H value). The curves were numbered as follows: 0 fern diversity; 1 selenium concentration in marine sediments; 2 extent of wet tropical biome; 3 extent of cool temperate biome; 4 magmatic activity; 5 atmospheric CO_2; 6 gymnosperm diversity; 7 angiosperm diversity; 8 extent of warm temperate biome; 9 extent of polar biome; 10 continental fragmentation; 11 extent of arid biome; 12 atmospheric O_2; 13 eustatic sea level; 14 diversity of lycophytes etc. (free sporing vascular plants excluding ferns); 15 global mean temperature; 16 mountain area.
