# Supplementary material for: Environmentally driven extinction and opportunistic origination explain fern diversification patterns
Source: Sci Rep. 2017 Jul 6;7:4831. doi: 10.1038/s41598-017-05263-7 (PMC5500532; doi:10.1038/s41598-017-05263-7)
Supplement: Supplementary file 4 — Dataset 3 [file 41598_2017_5263_MOESM4_ESM.doc]

Abbott, M.L., 1968. Lycopsid Stems and Roots and Sphenopsid Fructification and Stems from the Upper Freeport Coal of Southeastern Ohio. Palaeontographica Americana 6, 5–49.

Abdel-Kireem, M.R., Schrank, E., Samir, A.M., Ibrahim, M.I.A., 1996. Cretaceous palaeoecology, palaeogeography and palaeoclimatology of the northern Western Desert, Egypt. Journal of African Earth Sciences 22, 93–111. doi:10.1016/0899-5362(95)00125-5

Accarie, H., Beaudoin, B., Dejax, J., Friès, G., -G. Michard, J., Taquet, P., 1995. Découverte d’un Dinosaure théropode nouveau (Genusaurus sisteronis n. g., n. sp.) dans l’Albien marin de Sisteron (Alpes de Haute-Provence, France) et extension au Crétacé inférieur de la lignée cératosaurienne [Discovery of a new theropod dinosaur (Genusaurus sisteronis n. g., n. sp.) in the marine Albian of Sisteron (Alpes de Haute-Provence, France) and extension of the ceratosaur lineage into the Lower Cretaceous]. Comptes Rendus de l’Académie des Sciences à Paris, série IIa 320, 327–344.

Adamenko, O.M., 1974. Mesozoic and Cenozoic of the Steppe Altai.

Ahmad, F., Abu Hamad, A., Obeidat, M., 2012. Palynological study of the Early Cretaceous Kurnub Sandstone Formation, Mahis area, Central Jordan. Acta Palaeobotanica 52, 303–315.

Aliev, M.M., Genkina, R.Z., 1970. New data on paleobotanical characteristics of continental lower Mesozoic deposits, Kavak-Tau Ridge, Tyan’-Shan’, Iurskie, melovyei paleogenovye otlozheniya. Zapada Srednei Azii.

Alifanov, V.R., Sennikov, A.G., 2001. Discovery of dinosaur remains in a Moscow suburb. Doklady Earth Sciences 376, 1–3.

Allen, A.T., Lester, J.G., 1954. Contributions to the Paleontology of Northwest Georgia. Georgia Geological Survey Bulletin 62, 1–166.

Allenbach, R., van Konijnenburg-van Cittert, J.H.A., 1997. On a small flora with Araucariaceous conifers from the Röschenz Beds of Court, Jura Mountains, Switzerland. Eclogae geol. Helv. 90, 571–579.

Álvarez-Ramis, C., Prámparo, M., Papú, O.H., 2004. Estudio preliminar de paleofloras cretácicas procedentes de la base de la Formación Loncoche (Mendoza, Argentina). Coloquios de Paleontología 54, 7–14.

Amantov, V.A., Boguslavskii, I.S., Verbitzkaya, N.G., 1980. Pervaya nakhodka vulkanitov nizhnei permi y zapadnom zabaikalie [First finding of volcanites of Lower Permian age in West Transbaikal]. Sovetskaya Geologiya 5, 56–58.

Anashkina, K.K., 1969. On stratigraphical position of the Jurassic continental deposits of the Lower part of Argin’, Shilka, Amazar Rivers. Geologiia i Geofizika 63–69.

Anashkina, K.K., Rutshtein, I.G., Shmarlin, V.S., 1973. Lower Jurassic in the Shilka zone of the Mongol-Okhotsk suture. Sovetskaya Geologiya 137–141.

Anastas’eva, O.M., Migacheva, E.E., 1956. About variegated Mesozoic deposits of the south-western margin of the Russian platform, Geologicheskii sbornik Lvovskogo geologicheskogo obshchestva.

Anderson, H.M., Anderson, J.M., 2008. Molteno ferns : late Triassic biodiversity in southern Africa. Pretoria : South African National Biodiversity Institute.

Anderson, J.M., Anderson, H.M., 2000. Table 35 Molteno vegetative genera, phytosociological table.

Anderson, J.M., Anderson, H.M., 1985a. , Palaeoflora of Southern Africa. Prodromus of South African Megafloras Devonian to Lower Cretaceous.

Anderson, J.M., Anderson, H.M., 1985b. , Palaeoflora of Southern Africa. Prodromus of South African Megafloras Devonian to Lower Cretaceous.

Anderson, J.M., Anderson, H.M., 1985c. , Palaeoflora of Southern Africa. Prodromus of South African Megafloras Devonian to Lower Cretaceous.

Anderson, J.M., Anderson, H.M., 1985d. , Palaeoflora of Southern Africa. Prodromus of South African Megafloras Devonian to Lower Cretaceous.

Anderson, J.M., Anderson, H.M., 1985e. , Palaeoflora of Southern Africa. Prodromus of South African Megafloras Devonian to Lower Cretaceous.

Anderson, J.M., Anderson, H.M., 1985f. , Palaeoflora of Southern Africa. Prodromus of South African Megafloras Devonian to Lower Cretaceous.

Anderson, J.M., Anderson, H.M., 1983. Palaeoflora of Southern Africa Molteno Formation (Triassic), Palaeoflora of Southern Africa Molteno Formation (Triassic).

Andrews, H., Kasper, A., 1970. Plant fossils of the Trout Valley Formation. Maine Geological Survey Bulletin 23, 3–16.

Andrews, H., Kasper, A., Forbes, W., Gensel, P., Chaloner, W., 1977. Early Devonian flora of the Trout Valley Formation of northern Maine. Review of Palaeobotany and Palynology 23, 255–285.

Andrews, H., Kasper, A., Mencher, E., 1968. Psilophyton forbesii, a new Devonian plant from northern Maine. Bulletin of the Torrey Botanical Club 95, 1–11.

Andrews, H.N., Gensel, P.G., Forbes, W.H., 1974. An apparently heterosporous plant from the Middle Devonian of New Brunswick. Palaeontology 17, 387–408.

Andrews, H.N., Gensel, P.G., Kasper, A.E., 1975. A new fossil plant of probable intermediate affinities (Trimerophyte-Progymnosperm). Canadian Journal of Botany 53, 1719–1728.

Andrews, H.N., Kern, E.M., 1947. The Idaho Tempskyas and associated fossil plants. Annals of the Missouri Botanical Garden 34, 119–186.

Andrews, H.N., Phillips, T.L., 1968. Rhacophyton from the Upper Devonian of West Virginia. Journal of the Linnaen Society 61, 37–64.

Anhui Stratigraphic Group, 1978. The Stratigraphic Tables of Anhui, Geology Press.

Antoine, P.O., Marivaux, L., Croft, D.A., Billet, G., Ganerod, M., Jaramillo, C., Martin, T., Orliac, M., Tejada, J., Altamirano, A.J., Duranthon, F., Fanjat, G., Rousse, S., Salas-Gismondi, R., 2011. Middle Eocene rodents from Peruvian Amazonia reveal the pattern and timing of caviomorph origins and biogeography. Proceedings of The Royal Society B 279, 1319–1326.

Antunes, M.T., Mein, P., Nascimento, A., Pais, J., 1986. Le gisement pleistocene de Morgadinho, en Algarve. Ciencias da Terra (UNL) 8, 9–22.

Anzótegui, L.M., 2004. Megaflora de la Formación Chiquimil, (Mioceno Superior), en los valles de Santa María y Villavil, provincias de Catamarca y Tocumán, Argentina. Ameghiniana 41, 303–314.

Appert, O., 1977. Die Glossopteris flora der Sakoa in Sudwest-Madagaskar. Palaeontographica Abteilung B 162, 1–50.

Appert, O., 1973. DiePteridophyten aus dem Oberen Jura des Manamana in Sudwest-Madagaskar. Schweizerische Palaontologische Abhandlungen Memoires suisses de Paleontologie 94, 1–62.

Araujo Carvalho, M., 2001. Paleoenvironmental reconstruction based on palynological and palynofacies analyses of the Aptian-Albian succession in the Sergipe Basin, northeastern Brazil.

Archangelsky, A., Archangelsky, S., Poire, D.G., Canessa, N.D., 2008. Aptian Angiosperm Pollen from the Ticó Flora Patagonia, Argentina. Revista del Museo Argentino de Ciencias Naturales 10, 185–198.

Archangelsky, A., Llorens, M., 2005. Palinología de la Formación Kachaike, Cretácico Inferior de la Cuenca Austral, provincia de Santa Cruz. II. Esporas. Ameghiniana 42, 311–328.

Archangelsky, A., Llorens, M., 2003. Palinología de la Formación kachaike, Cretácico Inferior de la Cuenca Austral, provincia de Santa Cruz. I-Esporas lisas y cinguladas. Ameghiniana 40, 71–80.

Archangelsky, S., 2009. Biogeographic implications of Albian Mohria-like spores (Family Anemiaceae) in SW Gondwana (Patagonia). Review of Palaeobotany and Palynology 157, 301–308. doi:10.1016/j.revpalbo.2009.06.002

Archangelsky, S., 1973. Palinología del Paleoceno de Chubut. I. Descripciones Sistemáticas. Ameghiniana 10, 339–399.

Archangelsky, S., 1972. Esporas de la Formación Río Turbio (Eoceno), Provincia de Santa Cruz. Revista del Museo de la Plata (nueva serie), Sección Paleontología 6, 65–100.

Archangelsky, S., Archangelsky, A., 2013. Aptian angiosperm pollen from the Ticó Flora Patagonia, Argentina 174, 559–571. doi:10.1086/668693

Archangelsky, S., Archangelsky, A., Cladera, G., 2012. Palinología y paleoambientes en el perfil de Bajo Comisión (Cretácico), provincia de Santa Cruz, Argentina. Revista del Museo Argentino de Ciencias Naturales 14, 23–39.

Archangelsky, S., Cuneo, R., 1984. Zonacion del Permico continental de Argentina sobre la base de sus plantas fosiles. Memoria III Congreso Latinoamericano Paleontologia 143–158.

Archangelsky, S., Gamerro, J.C., 1966a. Estudio palinológico de la Formación Baqueró (Cretácico), provincia de Santa Cruz. II. Ameghiniana 4, 201–209.

Archangelsky, S., Gamerro, J.C., 1966b. Estudio palinológico de la Formación Baqueró (Cretácico), Provincia de Santa Cruz. III. Ameghiniana 4, 229–236.

Archangelsky, S., Gamerro, J.C., 1966c. Estudio palinológico de la Formación Baqueró (Cretácico), Provincia de Santa Cruz. IV. Ameghiniana 4, 363–372.

Archangelsky, S., Gamerro, J.C., 1965. Estudio Palinológico de la Formación Baqueró (Cretácico), Provincia de Santa Cruz I. Ameghiniana 4, 159–170.

Archangelsky, S., Villar de Seoane, L., 1998. Estudios palinológicos de la Formación Baqueró (Cretácico), Provincia de Santa Cruz, Argentina. VIII. Ameghiniana 35, 7–19.

Archangelsky, S., Villar de Seoane, L., 1994. Estudio palinológico de la Formación Baqueró (Cretácico), Provincia de Santa Cruz, Argentina. VI. Ameghiniana 31, 41–53.

Archangelsky, S., Villar de Seoane, L., 1992. Estudio palinológico de la Formación Baqueró (Cretácico), Provincia de Santa Cruz, Argentina. V. Publicación Especial de la Asociación Paleontológica Argentina 2, 23–27.

Archangelsky, S., Wagner, R.H., 1983. Glossopteris anatolica sp. nov. from uppermost Permian strata in south-east Turkey. British Museum of Natural History Bulletin 37, 81–91.

Arnold, C.A., 1955. A Tertiary Azolla from British Columbia. Contributions from the Museum of Paleontology, University of Michigan 12, 37–45.

Arnold, C.A., 1949. Fossil Flora of the Michigan Coal Basin. Contributions from the Musuem of Paleontology: University of Michigan 7, 131–269.

Arnold, C.A., 1941a. Observations on Fossil Plants from the Devonian of Eastern North America, V. Hyenia banksii, sp. Nov. Contributions from the Museum of Paleontology, University of Michigan 6, 53–57.

Arnold, C.A., 1941b. Some Paleozoic plants from Central Colorado and their stratigraphic significance. Contrib. Mus. Paleontol. University of Michigan 6, 59–70.

Arnold, C.A., 1939. Observations On Fossil Plants From the Devonian of Eastern North America IV. Plant Remains from the Catskill Delta Deposits of Northern Pennsylvania and Southern New York. Contributions from the Museum of Paleontology, University of Michigan 5, 271–314.

Arnold, C.A., 1932. Fossil Plants from the Pocono (Oswayo) Sandstone of Pennsylvania. Michigan Academy of Science Papers 17, 51–57.

Arnold, C.A., Sadlick, W., 1962. A Mississippian flora from northeastern Utah and its faunal and stratigraphic relation. Contributions from the Museum of Paleontology, University of Michigan XVII, 243–263.

Arrondo, O.G., 1972. Estudio geologico y paleontologico en la zona de la Estancia La Juanita y Aldredores, Provincia de Santa Cruz, Argentina. Revista del Museo de La Plata, n.s. (Paleontologia) 7, 1–194.

Arrondo, O.G., Morel, E.M., Ganuza, D.G., Herve, F., Parada, M.A., Muehlebach, W., 1988. La Formacion Panguipulli y su contenido paleofloristico Chile. Actas, Quinto Congreso Geologico Chileno y primeras jornadas de geofisica 137–145.

Arrondo, O.G., Petriella, B., 1980. Alicura, nueva localidad plantifera liasica de la provincia de Neuquen, Argentina. Ameghiniana 17, 200–215.

Ash, S., 1999. An Upper Triassic upland flora from north-central New Mexico, U.S.A. Review of Palaeobotany and Palynology 105, 183–199.

Ash, S., 1991. A new Jurassic Phlebopteris (Plantae, Filicales) from the Wallowa terrane in the Snake River Canyon, Oregon and Idaho. Journal of Paleontology 65, 322–329.

Ash, S., 1989. The Upper Triassic Chinle Flora of the Zuni Mountains, New Mexico, New Mexico Geological Society Guidebook, 40th Field Conference, Southeastern Colorado Plateau.

Ash, S.R., 1975. The Chinle (Upper Triassic) flora of southeastern Utah, Four Corners Geological Society Guidebook, 8th Field Conference, Canyonlands.

Ash, S.R., 1972. Late Triassic plants from the Chinle Formation in north-eastern Arizona. Palaeontology 15, 598–618.

Ash, S.R., Basinger, J.F., 1991. A high latitude Upper Triassic flora from the Heiberg Formation, Sverdrup Basin, Arctic Archipelago. Contributions to Canadian Paleontology, Geological Survey of Canada, Bulletin 412, 101–131.

Ash, S., Tidwell, W.D., 1982. Notes on Upper Paleozoic plants of central New Mexico, New Mexico Geological Society Guidebook, 33rd Field Conference, Albuquerque Country II.

Askin, R.A., 1990. Cryptogam spores from the upper Campanian and Maastrichtian of Seymour Island, Antarctica. Micropaleontology 141–156.

Askin, R.A., 1989. Endemism and heterochrony in the Late Cretaceous (Campanian) to Paleocene palynofloras of Seymour Island, Antarctica: implications for origins, dispersal and palaeoclimates of southern floras. Geological Society of America Special Paper 107–119.

Atta-Peters, D., Salami, M.B., 2006. Aptian-Maastrichtian palynomorphs from the offshore Tano Basin, western Ghana. Journal of African Earth Sciences 43, 379–394.

Aulenback, K.R., LePage, B.A., 1998. Taxodium wallisii sp. nov.: First occurence of Taxodium from the Upper Cretaceous. International Journal of Plant Sciences 159, 367–390.

Austen, P., 2010. Smokejacks fieldwork 2007-2009. Wealden News 28–31.

Axelrod, D., 1998. The Oligocene Haynes Creek Flora of Eastern Idaho. University of California Publications in Geological Sciences 143, 1–99.

Axelrod, D.I., 1995. The Miocene Purple Mountain Flora of Western Nevada 139, 1–62.

Axelrod, D.I., 1985. Miocene Floras From the Middlegate Basin, West-Central Nevada 129, 1–209.

Axelrod, D.I., 1964. The Miocene Trapper Creek Flora of Southern Idaho. University of California Publications in Geological Sciences 51, 1–148.

Axelrod, D.I., 1956. Mio-Pliocene floras from west-central Nevada. University of California Publications in Geological Sciences 33, 1–321.

Azcarate, V., Fasola, A., 1970. Sobre formas nuevas para la flora triasica de Los Molles. Boletin Museo Nacional de Historia Natural 29, 249–269.

Baez, A.M., Zamaloa, M.C., Romero, E.J., 1990. Nuevos hallazgos de microfloras y anuros Paleógenos en el Noroeste de Patagonia: implicancias Paleoambientales y Paleobiogeográficas. Ameghiniana 27, 83–94.

Baldoni, A., Dibbern, M., Genine, A., 1985. Nota sobre una especie de Blechnum (Blechnaceae - Pteridophyta) e impronta de Fagus subferruginea Dusen en el Terciario de la provincia de Chubut, Argentina. Ameghiniana 22, 229–232.

Baldoni, A.M., 1992. Palynology of the Lower Lefipan Formation (Upper Cretaceous) of Barranca de Los Perros,Chubut Province, Argentina. Part I. Cryptogam Spores and Gymnosperm Pollen. American Association of Stratigraphic Palynologists 16, 117–136.

Baldoni, A.M., 1987. Dos nuevas especies de megasporas de la Formacion Kachaike, Cretacico Inferior de Santa Cruz, Argentina, Anais do X Congreso Brasileiro de Paleontologia, Sociedade Brasileira de Paleontologia.

Baldoni, A.M., 1981a. Tafoflora del Jurasico superior de la zona de Lago Argentino provincia de Santa Cruz, Republica Argentina. Ameghiniana 18, 97–102.

Baldoni, A.M., 1981b. Tafofloras jurasicas y eocretacicas de America del Sur, Cuencas sedimentarias del Jurasico y Cretácico de America del Sur (Comite Sudamericano del Jurasico y Cretacico).

Baldoni, A.M., 1980. Analisis de algunas tafofloras jurasicas y eocretacicas de Argentina y Chile. Actas II Congreso Argentino de Paleontologia y Bioestratigrafia y I Congreso Latinoamericano de Paleontologia. Buenos Aires 1978 41–65.

Baldoni, A.M., 1979. Nuevos elementos paleofloristicos de la tafoflora de la Formacion Spring Hill, limite Jurasico-Cretacico, subsuelo de Argentina y Chile austral. Ameghiniana 16, 103–119.

Baldoni, A.M., 1978. Plantas fosiles Jurasicas del subsuelo de Plaza Huincul, Provincia del Neuquen. Boletin de la Asociacion Latinoamericana de Paleobotanica y Palinologia 5, 1–12.

Baldoni, A.M., Batten, D.J., 1997. Cretaceous megaspores from two boreholes in the Austral Basin, Santa Cruz Province, Argentina, and their stratigraphic and paleoenvironmental significance. Neues Jahrbuch für Geologie und Paläontologie, Abhandlungen 205, 97–110.

Baldoni, A.M., Olivero, E., 1983. Plantas fosiles de la Formacion Lago la Plata procedentes de Arroyo Canogas, Provincia de Chubut, Argentina. Ameghiniana 20, 34–40.

Baldoni, M.A., Barreda, V.D., 1986. Estudio palinológico de las formaciones López de Bertodano y Sobral, Isla Vicecomodoro Marambio, Antártida. Boletín del IG-USP, Serie Científica 89–98.

Balme, B.E., 1988. Miospores from Late Devonian (Early Frasnian) strata, Carnarvon Basin, Western Australia. Palaeontographica Abteilung B 209, 109–166.

Bando, Y., 1970. Lower Triassic ammonoids from the Kitakami Massif. Transactions and Proceedings of the Paleontological Society of Japan, N.S. 79, 337–354.

Banerji, J., 1992. Osmundaceous fronds in Lower Cretaceous beds at Chunakhal, Rajmahal Hills, Bihar, India. Alcheringa 16, 1–13.

Banks, H.P., 1981. Peridermal Activity (Wound Repair) in an Early Devonian (Emsian) Trimerophyte from the Gaspe Peninsula, Canada. The Palaeobotanist 28-29, 20–25.

Barabas-Stuhl, A., 1981. Microflora of the Permian and Lower Triassic sediments of the Mecsek Mountains (south Hungary). Acta Geologica Academiae Scientarum Hungaricae 24, 49–97.

Barale, G., 1981. La paleoflore Jurassique du Jura Francais: Etude systematique, aspects stratigraphiques et paleoecologiques, Documents des Laboratoires de Geologie Lyon.

Baranova, Z.E., Kirichkova, A.I., 1990. Structure-facial zonation and stratigraphy of the Lower-Middle Jurassic deposits of the PreCaspian basin. Sovetskaya Geologiya 56–67.

Baranova, Z.E., Kirichkova, A.I., 1972. New data on stratigraphy and flora of the Middle Jurassic deposits of the Emba region. Doklady Akademii Nauk SSSR 203, 1139–1142.

Baranova, Z.E., Kirichkova, A.I., Zauer, V.V., 1975. Stratigraphy and flora of the Jurassic deposits of the east Precaspian Basin. Trudy Vsesoyuznyi Neftyanoi 332.

Barreda, V., 1997a. Palinoestratigrafía de la Formación San Julián en el área de Playa La Mina (provincia de Santa Cruz), Oligoceno de la Cuenca Austral. Ameghiniana 34, 283–294.

Barreda, V., 1997b. Palinomorph assemblage of the Chenque Formation, Late Oligocene?- Miocene from Golfo San Jorge Basin, Patagonia, Argentina, Part 1: terrestrial Algae, trilete and monolete spores. Ameghiniana 34, 69–80.

Barreda, V.D., 1996. Bioestratigrafía de polen y esporas de la Formación Chenque, Oligoceno tardío?-Mioceno de las provincias de Chubut y Santa Cruz, Patagonia, Argentina. Ameghiniana 35–56.

Barreda, V.D., Cúneo, N.R., Wilf, P., Currano, E.D., Scasso, R.A., Brinkhuis, H., 2012. Cretaceous/Paleogene Floral Turnover in Patagonia: Drop in Diversity, Low Extinction, and a Classopollis Spike. PLoS ONE 7, e52455. doi:10.1371/journal.pone.0052455

Barreda, V.D., Gutiérrez, P.R., Limarino, C.O., 1998. Edad y paleoambiente de la “Serie del Yeso”, Valle del Cura, provincia de San Juan: evidencias palinológicas. Ameghiniana 35, 321–335.

Barreda, V.D., Ottone, E.G., Dávila, F.M., Astini, R.A., 2006. Edad y paleoambiente de la Formación del Buey (Mioceno), sierra de Famatina, La Rioja, Argentina: evidencias sedimentológicas y palinológicas. Ameghiniana 42, 215–226.

Barreda, V.D., Palamarczuk, S., Medina, F., 1999. Palinología de la Formación Hidden Lake (Coniaciano-Santoniano), Isla James Ross, Antártida. Revista Española de Micropaleontología 53–72.

Barreda, V., Palamarczuk, S., 2000a. Palinoestratigrafía de depósitos del Oligoceno tardío-Mioceno en el área sur del Golfo San Jorge, provincia de Santa Cruz, Argentina. Ameghiniana 37, 103–117.

Barreda, V., Palamarczuk, S., 2000b. Palinomorfos continentales y marinos de la Formación Monte León en su área tipo, provincia de Santa Cruz, Argentina. Ameghiniana 37, 3–12.

Bartholomai, A., 1966. The discovery of plesiosaurian remains in freshwater sediments in Queensland. The Australian Journal of Science 28, 437.

Bartlett, C.S., Jr., Webb, H.W., 1971. Geology of the Bristol and Wallace quadrangles, Virginia. Virginia Division of Mineral Resources Report of Investigations 25, 1–93.

Bashforth, A.R., Falcon-Lang, H.J., Gibling, M.R., 2010. Vegetation heterogeneity on a Late Pennsylvanian braided-river plain draining the Variscan Mountains, La Magdalena Coalfield, northwestern Spain. Palaeogeography, Palaeoclimatology, Palaeoecology 292, 367–390.

Bashirov, O.M., 1987. About the Rhaetian and Early Jurassic floras of the TransCaucasus. Izvestiya Akademii Nauk Azerbaidzhanskoi SSR. Earth Sciences 72–78.

Basson, P.W., 1968. The fossil flora of the Drywood Formation of southwestern Missouri. University of Missouri Studies, Columbia, MO USA 44.

Bateman, R.M., Morton, N., Dower, B.L., 2000. Early Middle Jurassic plant communities in Northwest Scotland: Paleoecological and paleoclimatic significance. GeoResearch Forum 6, 501–512.

Bateman, R.M., Rothwell, G.W., 1990. A reappraisal of the Dinantian floras at Oxroad Bay, East Lothian, Scotland. 1. Floristics and the development of whole-plant concepts. Transactions of the Royal Society of Edinburgh 81, 127–159.

Bateman, R.M., Scott, A.C., 1990. A reappraisal of the Dinantian floras at Oxroad Bay, East Lothian, Scotland. 2. Volcanicity, palaeoenvironments and palaeoecology. Transactions of the Royal Society of Edinburgh 81, 161–194.

Batten, D.J., 1988. Revision of S.J. Dijkstra’s Late Cretaceous megaspore and other plant microfossils from Limburg, the Netherlands. Mededelingen rijks geologische dienst 41, 1–55.

Batten, D.J., 1982. Palynofacies and salinity in the Purbeck and Wealden of southern England, Aspects of Micropalaeontology: Papers Presented to Professor Tom Barnard.

Batten, D.J., 1969. Some British Wealden megaspores and their facies distribution. Palaeontology 12, 333–350.

Batten, D.J., Collinson, M.E., 2001. Revision of species of Minerisporites, Azolla and associated plant microfossils from deposits of the Upper Palaeocene and Palaeocene/Eocene transition in the Netherlands, Belgium and the USA. Review of Palaeobotany and Palynology 115, 1–32.

Batten, D.J., Uwins, P.J., 1985. Early-Late Cretaceous (Aptian-Cenomanian) Palynomorphs. Journal of Micropalaeontology 4, 151–167.

Batyaeva, S.K., Vasil’eva, N.A., 1985. Locality Chernyi Etap-1, 2, 3, Trudy Paleontologicheskogo Instituta. Jurassic continental biocoenosis of Southern Siberia and surrounding areas.

Beck, C.B., 1957. Tetraxylopteris schmidtii Gen. et. sp. Nov., A Probable Pteridosperm Precursor from the Devonian of New York. American Journal of Botany 44, 350–367.

Becker, H.F., 1973. The York Ranch Flora of the Upper Ruby River Basin, Southwestern Montana. Palaeontographica Abteilung B 143, 18–93.

Becker, H.F., 1969. Fossil Plants of the Tertiary Beaverhead Basins in Southwestern Montana. Palaeontographica Abteilung B 127, 1–142.

Beheshev, I.I., Dzhalilov, Y.M., Portnyagina, L.A., Yudin, G.T., Mualla, A., Zaza, T., 1988. Triassic stratigraphy of Syria. International Geology Review 30, 1292–1301.

Beialy, S. El, Atfy, H.S. El, Zavada, M.S., Khoriby, E.M. El, 2010. Palynological, palynofacies, paleoenvironmental and organic geochemical studies on the Upper Cretaceous succession of the GPTSW-7 well, North Western Desert, Egypt. Marine and Petroleum Geology 27, 370–385.

Bell, J., Holden, J., Pettigrew, T.H., Sedman, K.W., 1979. The Marl Slate and basal Permian breccia at Middridge, Co. Durham. Proceedings of the Yorkshire Geological Society 42(3), 439–460.

Bell, W.A., 1962a. Flora of Pennsylvanian Pictou Group of New Brunswick, Bulletin of the Geological Survey of Canada.

Bell, W.A., 1962b. Upper Cretaceous floras of the Dunvegan, Bad Heart, and Milk River Formations of Western Canada. Geological Survey of Canada 94, 1–76.

Bell, W.A., 1960. Mississippian Horton Group of Type Windsor-Horton District, Nova Scotia. Geological Survey of Canada Memoir 314, 1–58.

Bell, W.A., 1957. Flora of the Upper Cretaceous Nanaimo Group of Vancouver Island, British Columbia. Geological Survey of Canada Memoir 293, 1–84.

Bell, W.A., 1956. Lower Cretaceous Floras of Western Canada. Geological Survey of Canada Memoir 285 1–153.

Bell, W.A., 1948. Early Carboniferous strata of St. Georges Bay area, Newfoundland. Canada Geological Survey Bulletin 10, 1–45.

Bell, W.A., 1940. The Pictou Coalfield, Nova Scotia. Canada Geologic Survey.

Belozerova, V.N., 1970. About the Jurassic flora of Kok-Yangak (Kirgizia). Vestnik Leningradskogo Universiteta 51–54.

Bel’tenev, E.B., Lebedev, E.L., 1968. New data on the age of volcanics adjacent to the Okhotsk sea. Doklady Akademii Nauk SSSR 182, 407–410.

Benecke, A.K., 1976. Several new forms of Glossopteris fructifications from the Beaufort Daptocephalus-Zone (Upper Permian) of Natal, South Africa. Palaeontologia Africana 19, 97–125.

Bera, S., Gupta, S., Khan, M.A., De, A., Mukhopadhyay, R., 2014. First megafossil evidence of Cyatheaceous tree fern from the Indian Cenozoic. Journal of Earth System Science 123, 1433–1438.

Bergad, R.D., Hall, J.W., 1971. A Cretaceous Azolla Massula with large Glochidia. The Botanical Gazette 132, 237–239.

Berry, C.M., 1994. First record of the Devonian lycophyte Leclercquia from South America. Geology Magazine 131, 269–272.

Berry, C.M., Edwards, D., 1996. The herbaceous lycophyte Haskinsia Grierson and Banks from the Devonian of western Venezuela, with observations on leaf morphology and fertile specimens. Botanical Journal of the Linnean Society 122, 103–122.

Berry, E.W., 1939. A Miocene flora from the gorge of the Yumuri River, Matanzas, Cuba. The Johns Hopkins University Studies in Geology 13, 95–135.

Berry, E.W., 1938. Tertiary flora from the Rio Pichileufu, Argentina. Geological Society of America Special Paper 12, 1–149.

Berry, E.W., 1937. Upper Cretaceous Plants from Patagonia. Science 86, 221–222.

Berry, E.W., 1936. Tertiary plants from Venezuela. Proceedings of the United States National Museum 83, 335–360.

Berry, E.W., 1933. A new Lygodium from the late Tertiary of Ecuador. Journal of the Washington Academy of Sciences 23, 208–210.

Berry, E.W., 1929. The flora of the Frontier Formation. United States Geological Survey Professional Paper 158, 129–135.

Berry, E.W., 1928. Tertiary fossil plants from the Argentine Republic. Proceedings of the United States National Museum 73, 1–27.

Berry, E.W., 1922a. Late Tertiary plants from Jancocata, Bolivia. The Johns Hopkins University Studies in Geology 4, 205–221.

Berry, E.W., 1922b. Pliocene fossil plants from Eastern Bolivia. The Johns Hopkins University Studies in Geology 4, 145–203.

Berry, E.W., 1922c. The flora of the Cheyenne Sandstone of Kansas. United States Geological Survey Professional Paper 129, 199–231.

Berry, E.W., 1922d. The Mesozoic flora of Peru, George Hungtington Williams Memorial Publication. Contributions to the Paleobotany of Peru, Bolivia, and Chile (Johns Hopkins University Studies in Geology, number 4).

Berry, E.W., 1921. Tertiary fossil plants from Venezuela. Proceedings of the United States National Museum 59, 553–579.

Berthelin, M., Broutin, J., Kerp, H., Crasquin-Soleau, S., Platel, J.P., Roger, J., 2003. The Oman Gharif mixed paleoflora: A useful tool for testing Permian Pangea reconstructions. Palaeogeography, Palaeoclimatology, Palaeoecology 196, 85–98.

Besairie, H., Collignon, M., 1972. Geologie de Madagascar I. Les Terrains Sedimentaires, Annales Geologiques de Madagascar.

Betekhtina, O.A., Sukhov, S.V., 1968. Fauna and flora of the Late Paleozoic of Gorlovka coal-bearing basin (South of the Novosibirsk region), Trudy. Institut Geologii i Geofiziki. Academy of Science of the USSR. Siberian division. Biostratigraphy of the Boreal Mesozoic.

Bettar, I., Meón, H., 2001. Palynological study of the Middle/Upper Albian transition inthe Tarfaya Basin (Southwest of Morocco) and some new data about the African-South-Amrican Province. Review of Micropaleontology 44, 107–123.

Blackburn, D.T., 1981. Tertiary megafossil flora of Maslin Bay, South Australia: numerical taxonomic study of selected leaves. Alcheringa 5, 9–28. doi:10.1080/03115518108565430

Blackburn, D.T., Sluiter, I.R.K., 1994. The Oligo-Miocene coal floras of southeastern Australia, in: Hill, R.S. (Ed.), History of the Australian Vegetation: Cretaceous to Recent. pp. 328–367.

Blazey, E.B., 1974. Fossil flora of the Mogollon Rim, central Arizona. Palaeontographica Abteilung B 146, 1–20.

Boardman, D.R., Iannuzzi, R., 2010. PRESENCE OF THE GENUS GIRIDIA, SPHENOPHYTE, IN THE PARANÁ BASIN (LOWER PERMIAN, RIO BONITO FORMATION). Revista Brasileira de Paleontologia 13, 5–12.

Bodylevskii, V.I., et al., 1972. Stratigraphy of the USSR. Jurassic System.

Bogner, J., Hoffman, G.L., Aulenback, K.R., 2005. A fossilized aroid infructescence, Albertarum pueri geno. nov. et sp. nov., of Late Cretaceous (late Campanian) age from the Horseshoe Canyon Formation of southern Alberta, Canada. Canadian Journal of Earth Sciences 83, 591–598.

Böhme, M., Aiglstorfer, M., -O. Antoine, P., Appel, E., Havlik, P., Métais, G., Laq The Phuc, Schneider, S., Setzer, F., Tappert, R., Dang Ngoc Tran, Uhl. J. Prieto, D., 2013. Na Duong (northern Vietnam) – an exceptional window into Eocene ecosystems from Southeast Asia. Zitteliana A 53, 120–167.

Boinet, T., Babin, C., Broutin, J., Lardeux, H., Pons, D., Racheboeuf, P., 1986. Les grandes etapes de l’evolution paleozoique du Massif de Santander (Andes de Colombie): signification de la discordance du Devonian moyen. Comptes Rendus de l’Academie des Sciences de Paris, Serie II 303, 707–712.

Bollinger, T., 1992. Kleinsäugerstratigraphie in der miozänen Hörnlischüttung (Ostschweiz); (Biostratigraphy of small mammals in the Hörnlischüttung of Miocene age (East Switzerland)). Documenta naturae 75, 1–296.

Bonamo, P.M., Banks, H.P., 1966. Calamophyton in the Middle Devonian of New York State. American Journal of Botany 53, 778–791.

Bond, G., 1973. The palaeontology of Rhodesia. Geological Survey of Rhodesia Bulletin 70, 1–121.

Boneham, R.F., 1974. Chieftain No.20 Flora (Middle Pennsylvanian) of Vigo County Indiana. Proceedings of the Indiana Academy of Science 84, 89–113.

Bose, M.N., 1961. Leaf-cuticle and other plant microfossils from the Mesozoic rocks of Andoya, Norway. The Palaeobotanist 8, 1–7.

Bose, M.N., Banerji, J., 1984. The fossil floras of Kachchh. I - Mesozoic megafossils. The Palaeobotanist 33, 1–189.

Bose, M.N., Taylor, E.L., Taylor, T.N., 1990. Gondwana Floras of India and Antarctica - a Survey and Reappraisal, Antarctic Paleobiology, Its Role in the Reconstruction of Gondwana.

Brack, S.D., 1970. On a New Structurally Preserved Arborescent Lycopsid Fructification from the Lower Pennsylvanian of North America. American Journal of Botany 57, 317–330.

Bradshaw, M.A., McCartan, L., 1991. Palaeoecology and systematics of Early Devonian bivalves from the Horlick Formation, Ohio Range, Antarctica. Alcheringa 15, 1–42.

Bradshaw, M.J., Penney, S.R., 1982. A cored Jurassic sequence from north Lincolnshire, England: stratigraphy, facies analysis and regional context. Geological Magazine 119, 113–134.

Bragin, Y.N., Golubev, S.A., Polyanskii, B.V., 1981. Stratigrfiya nizhnemezozoiskoi Uglenosnoi Formatsii Irana [Stratigraphy of the Lower Mesozoic coal-bearing formation in Iran]. Izvestiya Akademii Nauk SSSR. Seria geologicheskaya 8, 64–77.

Branagan, D.F., 1969. Northwestern Coalfield (In: The geology of New South Wales), Journal of the Geological Society of Australia.

Brauckmann, C., 1978. Beitrag zur Flora der Grube Guimarota (Ober-Jura; Mittel-Portugal). Geologica et Palaeontologica 12, 213–222.

Brauckmann, C., Herd, K.J., 2000. Eine weitere neue Breyeriiden-Art (Insecta: Palaeodictyoptera) aus dem Ober-Karbon von Osnabrück (Deutschland). Neues Jahrbuch für Geologie und Paläontologie, Monatshefte 2000, 333–344.

Brauer, D.F., 1981. Heterosporous, Barinophytacean Plants from the Upper Devonian of North America and A Discussion of the Possible Affinities of the Barinophytaceae. Review of Palaeobotany and Palynology 33, 347–362.

Brauer, D.F., 1980. Barinophyton citrulliforme (Barinophytales Incertae Sedis Barinophytaceae) from the Upper Devonian of Pennsylvania. American Journal of Botany 67, 1186–1206.

Brayard, A., Bucher, H., 2008. Smithian (Early Triassic) ammonoid faunas from northwestern Guangxi (South China): taxonomy and biochronology. Fossils and Strata 55, 1–179.

Brayard, A., Bylund, K.G., Jenks, J.F., Stephen, D.A., Olivier, N., Escarguel, G., Fara, E., Vennin, E., 2013. Smithian ammonoid faunas from Utah: implications for Early Triassic biostratigraphy, correlation and basinal paleogeography. Swiss Journal of Palaeontology 132, 141–219.

Brenner, G.J., 1974. Palynostratigraphy of the Lower Cretaceus Gevar’am and Talme Yafe Formations in the Gevar’am well (Southern Coastal Plain, Israel). Bulletin of the Geological Survey of Israel 59, 1–27.

Brenner, G.J., 1968. Middle Cretaceous spores and pollen from Northeastern Peru. Pollen et Spores 10, 341–383.

Brenner, G.J., Bickoff, I.S., 1992. Palynology and age of the Lower Cretaceous basal Kurnub Group from the coastal plain to the Northern Negev of Israel. Palynology 16, 137–185.

Bristow, C.R., 1991. Geology of the Petersfield district, Hampshire.

Brook, G.A., Burney, D.A., Coward, J., 1990. Desert paleoenvironmental data from cave speleothems with examples from the Chihuahuan. Palaeogeography, Palaeoclimatology, Palaeoecology 76, 311–330.

Broutin, J., 1986. Etude paleobotanique et palynologique du passage Carbonifere Permien dans le sud-ouest de la Peninsule Iberique, Cahiers de Paleontologie. Editions du Centre National de la Recherche Scientifique, Paris.

Broutin, J., Gisbert, J., 1985. Entorno paleoclimatico y ambiental de la flora Stephano-Autuniente del Pirineo Catalan. Compte Rendu, Dixieme Congres International de Stratigraphie et de Geologie du Carbonifere (Madrid, 1983) 3, 53–66.

Brown, B., 1913. A new crested dinosaur. The American Museum Journal 13, 138–144.

Brown, R., 1933. Fossil plants from the Aspen Shale of southwestern Wyoming. Proceedings of the United States National Museum 82, 1–10.

Brown, R.W., 1962. Paleocene flora of the Rocky Mountains and Great Plains. United States Geological Survey Professional Paper 375, 1–119.

Brühwiler, T., Bucher, H., 2012. Systematic palaeontology, in Smithian (Early Triassic) ammonoids from the Salt Range, Pakistan. Special Papers in Palaeontology 88, 22–114.

Brühwiler, T., Bucher, H., Goudemand, N., Galfetti, T., 2012a. Smithian (Early Triassic) ammonoid faunas from exotic blocks from Oman: taxonomy and biochronology. Palaeontographica Abteilung A 296, 3–107.

Brühwiler, T., Bucher, H., Krystyn, L., 2012b. Middle and Late Smithian (Early Triassic) ammonoids from Spiti, India. Special Papers in Palaeontology 88, 115–174.

Brühwiler, T., Bucher, H., Roohi, G., Yaseen, A., Rehman, K., 2011. A new early Smithian ammonoid fauna from the Salt Range (Pakistan). Swiss Journal of Palaeontology 130, 187–201.

Brühwiler, T., Bucher, H., Ware, D., Schneebeli-Hermann, E., Hochuli, P.A., Roohi, G., Reahman, K., Yaseen, A., 2012c. Smithian (Early Triassic) ammonoids from the Salt Range, Pakistan. Special papers in Palaeontology 88, 5–114.

Brühwiler, T., Ware, D., Bucher, H., Krystyn, L., Goudemand, N., 2010. New Early Triassic ammonoid faunas from the Dienerian/Smithian boundary beds at the Induan/Olenekian GSSP candidate at Mud (Spiti, Northern India). Journal of Asian Earth Sciences 39, 724–739.

Buddington, A.F., Chapin, T., 1929. Geology and mineral deposits of southeastern Alaska. United States Geological Survey Bulletin 800, 1–398.

Buffetaut, E., Cuny, G., Lachkar, G., Contini, D., Pharisat, A., Vienet, B., 1995. Le fémur de dinosaure théropode du Musée d’Histoire naturelle de Gray (Haute-Sâone): identification, datation et essai de localisation de l’origine d’un fossile inhabituel [The theropod dinosaur femur from the Natural History Museum of Gray Haute-Sâone, France): identification, dating and determination of the geographic origin of an unusual fossil]. Bulletin de la Société Géologique de France 166, 69–75.

Burakova, A.T., 1971. Jurassic flora of Greater Balkhan Ridge. Vestnik Leningradskogo Universiteta 28–35.

Burakova, A.T., 1965. On age of the lower part of the Jurassic unit in the south-western mountain chains of Gissar Ridge. Vestnik Leningradskogo Universiteta 41–46.

Burakova, A.T., Fedorov, P.V., 1989. On age of the lower part of the red unit, upper part of river Kugart (North-eastern Fergana). Vestnik Leningradskogo Universiteta 1, 67–70.

Burakova, A.T., Mikulin, M.V., Kurbatov, V.V., 1973. Age subdivision of the Jurassic continental unit of Zeravshan-Gissar mountain area based on floral data (Tadzhikistan). Vestnik Leningradskogo Universiteta 45–51.

Burakova, A.T., Verzilin, N.N., Mironenko, O.A., 1978. Jurassic flora of Kok-Yangak brown coal district and its paleoclimatical significance. Vestnik Leningradskogo Universiteta 41–48.

Burnham, R.J., 1994. Paleoecological and Floristic Heterogeneity in the Plant-Fossil Record - An Analysis Based on the Eocene of Washington. United States Geological Survey Bulletin 2085-B, 1–36.

Bur. of Geol. and Min. Resources of Anhui, 1987. Regional Geology of Anhui Province, People’s Republic of China Ministry of Geology and Mineral Resources, Geological Memoirs.

Bur. of Geol. and Min. Resources of Fujian, 1985. Regional Geology of Fujian Province, People’s Republic of China Ministry of Geology and Mineral Resources, Geological Memoirs.

Bur. of Geol. and Min. Resources of Gansu, 1989. Regional Geology of Gansu Province, People’s Republic of China Ministry of Geology and Mineral Resources, Geological Memoirs.

Bur. of Geol. and Min. Resources of Guangdong, 1988. Regional Geology of Guangdong Province, People’s Republic of China Ministry of Geology and Mineral Resources, Geological Memoirs.

Bur. of Geol. and Min. Resources of Guangxi Zhuang, 1985. Regional geology of Guangxi Zhuang Autonomous Region, People’s Republic of China Ministry of Geology and Mineral Resources, Geological Memoirs.

Bur. of Geol. and Min. Resources of Guizhou, 1987. Regional Geology of Guizhou Province, People’s Republic of China Ministry of Geology and Mineral Resources, Geological Memoirs.

Bur. of Geol. and Min. Resources of Hebei, 1989. Regional Geology of Hebei Province, Beijing Municipality and Tianjing Municipality, People’s Republic of China Ministry of Geology and Mineral Resources, Geological Memoirs.

Bur. of Geol. and Min. Resources of Henan, 1989. Regional Geology of Henan province, People’s Republic of China Ministry of Geology and Mineral Resources, Geological Memoirs.

Bur. of Geol. and Min. Resources of Hubei, 1990. Regional geology of Hubei Provence, People’s Republic of China Ministry of Geology and Mineral Resources, Geological Memoirs.

Bur. of Geol. and Min. Resources of Hunan, 1988. Regional Geology of Hunan Province, People’s Republic of China Ministry of Geology and Mineral Resources, Geological Memoirs.

Bur. of Geol. and Min. Resources of Jiangsu, 1984. Regional geology of Jiangsu Province and Shanghai Municipality, People’s Republic of China Ministry of Geology and Mineral Resources, Geological Memoirs.

Bur. of Geol. and Min. Resources of Jiangxi, 1984. Regional Geology of Jiangxi Province, People’s Republic of China Ministry of Geology and Mineral Resources, Geological Memoirs.

Bur. of Geol. and Min. Resources of Jilin, 1989. Regional Geology of Jilin Province, People’s Republic of China Ministry of Geology and Mineral Resources, Geological Memoirs.

Bur. of Geol. and Min. Resources of Liaoning, 1989. Regional Geology of Liaoning Province, People’s Republic of China Ministry of Geology and Mineral Resources, Geological Memoirs.

Bur. of Geol. and Min. Resources of Shaanxi, 1989. Regional Geology of Shaanxi Province, People’s Republic of China Ministry of Geology and Mineral Resources, Geological Memoirs.

Bur. of Geol. and Min. Resources of Shanxi, 1989. Regional Geology of Shanxi Province, People’s Republic of China Ministry of Geology and Mineral Resources, Geological Memoirs.

Bur. of Geol. and Min. Resources of Sichuan, 1991. Regional Geology of Sichuan Province, People’s Republic of China Ministry of Geology and Mineral Resources, Geological Memoirs.

Bur. of Geol. and Min. Resources of Yunnan, 1990. Regional Geology of Yunnan Province, People’s Republic of China Ministry of Geology and Mineral Resources, Geological Memoirs.

Bur. of Geol. and Min. Resources of Zhejiang, 1989. Regional Geology of Zhejiang Province, People’s Republic of China Ministry of Geology and Mineral Resources, Geological Memoirs.

Burton, C.K., 1973. Mesozoic, Geology of the Malay Peninsula (West Malaysia and Singapore).

Bystritskaya, L.I., 1974. Plant complexes in Jurassic deposits of the Kuzbass. Trudy Tomskogo Gosudarstvennogo Universiteta 227, 32–49.

Cabrera, L., Jung, W., Kirchner, M., Sáez, A., Schleich, H.H., 1994. Crocodilian and palaeobotanical findings from the Tertiary Lignites of the As Pontes Basin (Galicia, NW-Spain) (Crocodylia, Plantae). Courier Forschungsinstitut Senckenberg 173, 153–165.

Cahoon, E.J., 1960. Sphenobaiera ikorfatensis F. papillata from the Lakota Formation of the Black Hills. Bulletin of the Torrey Botanical Club 87, 247–257.

Cai, C.Y., Dou, Y.W., Edwards, D., 1993. New observations on a Pridoli plant assemblage from north Xinjiang, northwest China, with comments on its evolutionary and palaeogeographica significance. Geological Magazine 130, 155–170.

Campbell, J.D., Untergasser, B., 1972. Two new megaspore species from the continental Upper Cretaceous (Campanian-Maastrichtian) of the Alberta Plains. Canadian Journal of Botany 50, 2553–2557.

Canright, J.E., 1978. A Late Paleozoic Flora from Promontory Butte in Central Arizona. Paleontology, Stratigraphy, and Vegetation in East-Central Arizona: American Association of Stratigraphic Palynologists 11th Annual Meeting 25–30.

Canright, J.E., 1968. Spores and Associated Macrofossils from the Devonian of Arizona. Geoscience and Man, American Association of Stratigraphic Palynologists, Proceedings of the First Annual Meeting 1, 83–89.

Canright, J.E., Blazey, E.B., 1974. A Lower Permian flora from Promontory Butte, central Arizona, In S.R. Ash(ed.), Guidebook to Devonian, Permian and Triassic Plant Localities, East-central Arizona. Paleobotanical Section, Botanical Society of America, 25th Annual AIBS Meeting.

Cantrill, D.J., Nagalingum, N.S., 2005. Ferns from the Cretaceous of Alexander Island, Antarctica: Implications for Cretaceous phytogeography of the Southern Hemisphere. Review of Palaeobotany and Palynology. doi:10.1016/j.revpalbo.2005.08.004

Cantrill, D.J., Nichols, G.J., 1996. Taxonomy and palaeoecology of Early Cretaceous (Late Albian) angiosperm leaves from Alexander Island, Antarctica. Review of Palaeobotany and Palynology 92, 1–28.

Cao, L., Wang, X., 1999. Heterospores of the Genus Ariadnaesporites (Salviniaceae) from the Late Cretaceous, Heilongjiang. Acta Micropalaeontologica Sinica 16, 50–53.

Carpenter, R.J., 1991. Palaeovegetation and Environment at Cethana, Tasmania.

Carpenter, R.J., Jordan, G.J., Hill, R.S., 2007. A Toothed Lauraceae Leaf from the Early Eocene of Tasmania, Australia. International Journal of Plant Sciences 168, 1191–1198.

Carpenter, R.J., Jordan, G.J., Macphail, M.K., Hill, R.S., 2012. Near-tropical Early Eocene terrestrial temperatures at the Australo-Antarctic margin, western Tasmania. Geology 40, 267–270. doi:10.1130/G32584.1

Carrillo Bravo, J., 1965. Estudio geologico de una parte del anticlinorio de Huayacocotla. Bulletin of the Mexican Association of Petroleum Geologists 17, 73–96.

Cartwright, A.M., Cleland, T.P., Derby, S.F., Ergas, E.E., Fay, H.I., Loughney, K.M., Riedel, J.A., Rocheford, M.K., Scott, C.L., Skaggs, K.L., 2005. Aspects of the paleontology and stratigraphy of the lower Triassic-Lower Cretaceous strata of the eastern Bighorn Basin, WY. Geological Society of America Abstracts with Programs 37, 302.

Carvalho, M.R., Wilf, P., J Hermsen, E., Gandolfo, M.A., Cúneo, N.R., Johnson, K.R., 2013. First record of Todea (Osmundaceae) in South America, from the early Eocene paleorainforests of Laguna del Hunco (Patagonia, Argentina). American Journal of Botany 100, 1831–1848.

Cecil, C.B., 1990. Paleoclimate controls on stratigraphic repetition of chemical and siliciclastic rocks. Geology 18, 533–536.

Central South China Stratigraphic Group, 1974. The Stratigraphic Tables of Central South China, Geology Press.

Chamberlain, A.K., 1981. Biostratigraphy of the Great Blue Formation, Brigham Young University Geology Studies.

Chandler, M.E.J., 1964. The Lower Tertiary Floras of Southern England. IV. A summary and survey of findings in the light of recent botanical observations.

Chandler, M.E.J., 1963a. , The Lower Tertiary Floras of Southern England. III. Flora of the Bournemouth Beds, The Boscombe, and the Highcliff Sands. British Museum (Natural History), London.

Chandler, M.E.J., 1963b. , The Lower Tertiary Floras of Southern England. III. Flora of the Bournemouth Beds, The Boscombe, and the Highcliff Sands. British Museum (Natural History), London.

Chandler, M.E.J., 1963c. , The Lower Tertiary Floras of Southern England. III. Flora of the Bournemouth Beds, The Boscombe, and the Highcliff Sands. British Museum (Natural History), London.

Chandler, M.E.J., 1963d. , The Lower Tertiary Floras of Southern England. III. Flora of the Bournemouth Beds, The Boscombe, and the Highcliff Sands. British Museum (Natural History), London.

Chandler, M.E.J., 1963e. , The Lower Tertiary Floras of Southern England. III. Flora of the Bournemouth Beds, The Boscombe, and the Highcliff Sands. British Museum (Natural History), London.

Chandler, M.E.J., 1962. The Lower Tertiary Floras of Southern England. II. Flora of the Pipe-Clay Series of Dorset (Lower Bagshot), British Museum (Natural History) London. .

Chandler, M.E.J., 1961a. , The Lower Tertiary Floras of Southern England. I. Palaeocene Floras. London Clay Flora (Supplement). British Museum (Natural History) London. 1-354 + 34 Plates.

Chandler, M.E.J., 1961b. , The Lower Tertiary Floras of Southern England. I. Palaeocene Floras. London Clay Flora (Supplement). British Museum (Natural History) London. 1-354 + 34 Plates.

Chandler, M.E.J., 1961c. , The Lower Tertiary Floras of Southern England. I. Palaeocene Floras. London Clay Flora (Supplement). British Museum (Natural History) London. 1-354 + 34 Plates.

Chandler, M.E.J., 1961d. Flora of the Lower Headon Beds of Hampshire and the Isle of Wight. Bulletin of the British Museum (Natural History) Geology 5, 93–157.

Chandler, M.E.J., 1957. The Oligocene Flora of the Bovey Tracey Lake Basin, Devonshire. Bulletin of the British Museum (Natural History) Geology 3, 71–123.

Chandrasekharam, A., 1974. Megafossil flora from the Genesee Locality, Alberta, Canada. Palaeontographica Abteilung B 147, 1–41.

Chao, K.K., 1959. Lower Triassic Ammonoids from Western Kwangsi, China. Palaeontologica Sinica. New Series 9, 1–33.

Chitaley, S., McGregor, D.C., 1988. Bisporangiostrobus harrisii gen. et sp. nov., An eligulate lycopsid cone with duosporites megaspores and geminospora microspores from the Upper Devonian of Pennsylvania, U.S.A. Palaeontographica Abteilung B 210, 127–149.

Christie, R.L., 1964. Geological Reconnaissance of northeastern Ellesmere Island, District of Franklin. Geological Survey of Canada Memoir 331, 1–79.

Christophel, D.C., 1994. The early Tertiary macrofloras of continental Australia, in: History of the Australian Vegetation. Cretaceous to Recent.

Christophel, D.C., 1976. Fossil floras of the Smoky Tower locality, Alberta, Canada. Palaeontographica Abteilung B 157, 1–43.

Christophel, D.C., Greenwood, D.R., 1987. A megafossil flora from the Eocene of Golden Grove South Australia. Transactions of the Royal Society of South Australia 111, 155–162.

Christophel, D.C., Harris, W.K., Syber, A.K., 1987. The Eocene flora of the Anglesea Locality, Victoria. Alcheringa 11, 303–323. doi:10.1080/03115518708619139

Ciarapica, S.G., Cirilli, S., Martini R., Rettori R., Zaninetti L., Salvini-Bonnard G., 1990. Carbonate buildups and associated facies in the Monte Facito Formation (southern Apennines). Boll. Soc. Geol. Italiana 109, 151–164.

Cladera, G., del Fueyo, G.M., de Seoane, L.V., Archangelsky, S., 2007. Early Cretaceous riparian vegetation in Patagonia, Argentina. Revista del Museo Argentino de Ciencias Naturales 9, 49–58.

Clapham, W.B., 1970. Permian miospores from the Flowerpot Formation of western Oklahoma. Micropaleontology 16, 15–36.

Cleal, C.J., Rees, P.M., 2003. The Middle Jurassic flora from Stonesfield, Oxfordshire, UK. Palaeontology 46, 739–801.

Cleal, C.J., Thomas, B.A., 1995. Palaeozoic Palaeobotany of Great Britain, Geological Conservation Review Series, Chapman & Hall: London.

Clyde, W.C., Wilf, P., iglesias, A., Slingerland, R.L., Barnum, T., Bijl, P.K., Bralower, T.J., Brinkhuis, H., Comer, E.E., Huber, B.T., Ibañez-Mejia, M., 2014. New age constraints for the Salamanca Formation and lower Río Chico Group in the western San Jorge Basin, Patagonia, Argentina: Implications for Cretaceous-Paleogene extinction recovery and land mammal age correlations. Geological Society America Bulletin 126, 289–306. doi:10.1130/B30915.1

Collignon, M., 1973. Ammonites du Trias inferieur et moyen d’Afghanistan. Annales de Paleontologie (Invertebres) 29, 127–183.

Collinson, M., Kvacek, Z., Zastawniak, E., 2001. The aquatic plants Salvinia (Salviniales) and Limnobiophyllum (Arales) from the Late Miocene flora of Sosnica (Poland). Acta Palaeobot. 41, 253–282.

Conran, J.G., Kaulfuss, U., Bannister, J.M., Mildenhall, D.C., Lee, D.E., 2010. Davallia (Polypodiales: Davalliaceae) macrofossils from Early Miocene Otago (New Zealand) with in situ spores. Review of Palaeobotany and Palynology 84–94.

Cook, E., Ross, A.J., 1996. The stratigraphy, sedimentology and palaeontology of the Lower Weald Clay (Hauterivian) at Keymer Tileworks, West Sussex, southern England. Proceedings of the Geologists’ Association 107, 231–239.

Coope, G.R., Shotton, F.W., Strachan, I., Dance, S.P., 1961. A Late Pleistocene fauna and flora from Upton Warren, Worcestershire. Philosophical Transactions of the Royal Society of London. Series B, Biological Sciences 244, 379–421.

Cooray, P.G., 1967. An Introduction to the Geology of Ceylon.

Cornet, B., Olsen, P.E., 1990. Early to Middle Carnian (Triassic) Flora and Fauna of the Richmond and Taylorsville Basins, Virginia and Maryland, U.S.A., Virginia Museum of Natural History Guidebook.

Cornet, B., Phillips, T.L., Andrews, H.N., 1976. The Morphology and Variation in Rhacophyton ceratangium from the Upper Devonian and its Bearing on Frond Evolution. Palaeontographica Abteilung B 158, 105–129.

Corsin, P., Stampfli, G., 1977. La Formation de Shemshak dans L’Elburz Oriental (Iran) Flore-Stratigraphie-Paleogeographie. Géobios 10, 509–571.

Cortes, J.M., Baldoni, A.M., 1984. Plantas fosiles Jurasicas al sur del rio Chubut medio. Actas, Noveno Congreso Geologico Argentino 4, 432–443.

Cridland, A.A., Morris, J.E., 1963. Taeniopteris, Walchia and Dichophyllum in the Pennsylvanian System of Kansas. University of Kansas Science Bulletin 44, 71–85.

Croft, W.N., Lang, W.H., 1942. The Lower Devonian flora of the Senni Beds of Monmouthsire and Breconshire. Philosophical Transactions of the Royal Society of Britain 221, 131–163.

Csiki, Z., Ionescu, A., Grigorescu, D., 2008. The Budurone microvertebrate site from the Maastrichtian of the Hateg Basin – flora, fauna, taphonomy and paleoenvironment. Acta Palaeontologica Romaniae 6, 49–66.

Ctyroky, P., 1973. Permian flora from the Ga’ara region (western Iraq). Neues Jahrbuch für Geologie und Paläontologie Monatshefte 383–388.

Curry, R.P., 1975. Miospores from the Upper Devonian (Frasnian) Greenland Gap Group, Allegheny Front, Maryland, West Virginia and Virginia, U.S.A. Review of Palaeobotany and Palynology 20, 119–131.

Dagis, A.S., Kazakov, A.M., 1984. Stratigraphy, lithology and cyclicity of Triassic deposits of north-central Siberia. Trudy Instituta Geologii i Geofiziki (Novosibirsk) 586, 1–178.

Damborenea, S.E., 1987. Early Jurassic Bivalvia of Argentina: Part 1, Stratigraphical introduction and superfamilies Nuculanacea, Arcacea, Mytilacea and Pinnacea. Palaeontographica A199, 23–111.

Da-Ning, W., Ying-Niang, Z., 1990. Late Cretaceous to Tertiary palynofloras in Xinjiang and Qinghai, China. Review of Palaeobotany and Palynology 65, 9–104. doi:10.1016/0034-6667(90)90060-V

Danze-Corsin, P., 1956. Contribution a l’etude des flores devoniennes du Nord de la France. II. - Flore eodevonienne de Rebreuve. [Contribution to the Study of the Devonian Flora of Northern France. Part II - Eodevonian Flora of Rebreuve]. Extrait des Annales de la Societe Geologique du Nord 76, 24–50.

Danze-Corsin, P., 1955. Contribution a l’etude des flores devoniennes du Nord de la France. I. - Flore eodevonienne de Matringhem. [Contribution to the Study of the Devonian Flora of Northern France. Part I - Eodevonian Flora of Matringhem.]. Extrait des Annales de la Societe Geologique du Nord 75, 143–159.

Darrah, W.C., 1969. A Critical Review of the Upper Pennsylvanian Floras of Eastern United States with Notes on the Mazon Creek Flora of Illinois.

Dawson, J.W., 1882. The Fossil Plants of the Erian (Devonian) and Upper Silurian Formations of Canada. Geological Survey of Canada 95–141.

Dawson, J.W., 1873. Report on the Fossil Plants of the Lower Carboniferous and Millstone Grit Formations of Canada, Geological Survey of Canada.

Dawson, J.W., 1871. On New Tree Ferns and Other Fossils from the Devonian. Quarterly Journal of the Geological Sciences 27, 269–278.

Dawson, J.W., 1867. On some remains of Palaeozoic insects recently discovered in Nova Scotia and New Brunswick. The Canadian Naturalist 3, 202–206.

De, A., De, B., Bera, S., 2003. First Record of Raniganjia Bengalensis (Rigby) Pant & Nautiyal from Lower Permian Beds (Barakar Formation) of South Karanpura Coalfield, Jharkhand, India. Geological Society of India 61, 487–490.

de Brun, P., Vedel, L., 1919. Etude géologique et paléontologique des environs de St-Ambroix (Gard). Première partie (Houiller, Trias, Infralias). Imprimerie Générale, Nimes 1–220.

De Jong, T.A.H., 1974. The fossil flora of Bous-Volkingen (Upper Westphalian, Saar Basin).

Delair, J.B., Sarjeant, W.A.S., 1985. History and bibliography of the study of fossil vertebrate footprints in the British Isles: supplement 1973–1983. Palaeogeography, Palaeoclimatology, Palaeoecology 49, 123–160.

Delevoryas, T., Srivastava, S.C., 1981. Jurassic plants from the Department of Francisco Morazan, central Honduras. Review of Palaeobotany and Palynology 34, 345–357.

de Lima, M.R., 1982. Palinologia da Formaçao Codo Na Regiao de Codo, Maranhao. Instituto de Geociências , Universidade de São Paulo 13, 43–134.

Delle, G.V., 1967. Middle Jurassic flora of Tkvarcheli coal basin (TransCaucasus), Academy of Sciences of the USSR. Botanical Institute. Trudy. Series 8. Paleobotanika. Problems in studies of fossil flora of coal deposits of the USSR.

Delle, G.V., 1962. Materials about the Jurassic flora of the Azerbaijan. Botanicheskii Zhurnal 47, 690–693.

Delle, G.V., 1960. New data on Jurassic flora of Tkvarcheli. Doklady Akademii Nauk SSSR 133, 1150–1153.

de Oliveira, M.E.C.B., Pons, D., 1975. Taphoflora of Karoo in the Zambesi Basin (Tete Region, Mozambique). Boletim IG, Instituto de Geociencias, Universidade de Sao Paulo 6, 33–53.

de Sousa, M.J., Wagner, R.H., 1983. New data on the fossil flora of the Pejão Coalfield and the adjoining Paraduça No 1 Mine (Dura Basin, North Portugal), Contributions to the Carboniferous Geology and Palaeontology of the Iberian Peninsula.

Dettmann, M.E., 1989. Antarctica: Cretaceous cradle of austral temperate rainforests?, Origins and Evolution of the Antarctic Biota.

Dettmann, M.E., 1973. Angiosperm pollen from Albian to Turonian sediments of Eastern Australia. Special publications of the geological Society of Australia 3–34.

Dettmann, M.E., 1963. Upper Mesozoic microfloras from South-Eastern. Procidings of Royal Society of Victoria 1–176.

Dettmann, M.E., Thomson, M.R.A., 1987. Cretaceous palynomorphs from the James Ross Island area, Antarctica - A pilot Study. British Antarctic Survey Bulletin 13–59.

Dietl, G., Schweigert, G., 2011. Im Reich der Meerengel. Der Nusplinger Plattenkalk und seine Fossilien.

Diez, J.B., Grauvogel-Stamm, L., Broutin, J., Ferrer, J., Gisbert, J., Linan, E., 1996. Première découverte d’un paléoflora anisienne dans la faciès Buntsandstein de la branche aragonaise de la Cordillère Ibérique (Espagne). C.R. Acad. Sci. Paris, t. 323, série II a 341–347.

Dijkstra, S.J., 1961a. On megaspores, Charophyta fruits and some other small fossils from the Cretaceous. The Palaeobotanist 8, 8–18.

Dijkstra, S.J., 1961b. Some Paleocene megaspores and other small fossils. Mededelingen van de Geologische Stichting, Nieuwe Serie 5–11.

Dijkstra, S.J., 1951. Wealden Megaspores and their stratigraphical value. Mededelingen Van de Geologische Stichting, Nieuwe Serie 7–22.

Dijkstra, S.J., 1949. Megaspores and some other fossils from the Aachenian (Senonian) in South Limburg, Netherlands. Mededelingen Van de Geologische Stichting, Niewe Serie 19–32.

Dilcher, D.L., Lott, T.A., Axsmith, B.J., 2005. Fossil plants from the Union Chapel Mine, Alabama, Pennsylvanian Footprints in the Black Warrior Basin of Alabama.

DiMichele, W.A., Aronson, R.B., 1992. The Pennsylvanian-Permian vegetational transition: a terrestrial analogue to the onshore-offshore hypothesis. Evolution 46, 807–824.

DiMichele, W.A., Dolph, G., 1981. Compression floras of the Upper Mansfield/Lower Brazil and Upper Staunton Formation in Parke and Clay Counties, Indiana, Guidebook to Pennsylvanian Plant Localities, AIBS Field Trip No. 2. Indiana University Press, Bloomington, IN USA.

DiMichele, W.A., Eble, C.F., Chaney, D.S., 1996. A drowned lycopsid forest above the Mahoning Coal (Conemaugh Group, Upper Pennsylvanian) in eastern Ohio, U.S.A. International Journal of Coal Geology 31, 249–276.

DiMichele, W.A., Mamay, S.H., Chaney, D.S., Hook, R.W., Nelson, W.J., 2001. An Early Permian flora with Late Permian and Mesozoic affinities from north-central Texas. Journal of Paleontology 75, 449–460.

Dimichele, W.A., Nelson, W.J., 1989. Small-Scale Spatial Heterogeneity in Pennsylvanian-Age Vegetation from the Roof Shale of the Springfield Coal (Illinois Basin). Palaios 4, 276–280.

DiMichele, W.A., Pfefferkorn, H.W., Phillips, T.L., 1996. Persistence of Late Carboniferous tropical vegetation during glacially driven climatic and sea-level fluctuations. Palaeogeography, Palaeoclimatology, Palaeoecology 125, 105–128.

Dimichele, W.A., Phillips, T.L., McBrinn, G.E., 1991. Quantitative Analysis and Paleoecology of the Secor Coal and Roof-Shale Floras (Middle Pennsylvanian, Oklahoma). Palaios 6, 390–409.

Dobruskina, I.A., 1994. Triassic Floras of Eurasia, Osterreichische Akademie der Wissenschaften Schriftenreihe der Erdwissenschaftlichen Kommissionen. H. Zapfe (Ed.), Springer-Verlag, New York.

Dobruskina, I.A., 1982. Triassic Floras of Eurasia, Akademia Nauk SSSR, Transactions.

Dobruskina, I.A., 1961. About the Mesozoic flora of the upper part of Amur river. Vestnik Moskovskogo Universiteta 29–35.

Dolding, P.J.D., 1992. Palynology of the Marambio Group (Upper Cretaceous) of northern Humps Island. Antartic Science 4, 311–326.

Doludenko, M.P., Orlovskaya, E.R., 1976. Jurassic floras of the Karatau Range, southern Kazakhstan. Palaeontology 19, 627–640.

Doludenko, M.P., Svanidze, T.I., 1969. The Late Jurassic flora of Georgia.

Doludenko, M.P., Svanidze, T.I., 1968. Callovian flora of Georgia and boundary between the Middle and Upper Jurassic. Izvestiya Akademii Nauk SSSR. Seria geologicheskaya 119–131.

Doran, J.B., 1980. A new species of Psilophyton from the Lower Devonian of northern New Brunswick, Canada. Canadian Journal of Botany 58, 2241–2262.

Dorf, E., 1942. Upper Cretaceous floras of the Rocky Mountain region; Flora of the Lance Formation at its type locality, Niobrara County, Wyoming. Contributions to Palaeontology.

Dorf, E., 1934. Lower Devonian flora from Beartooth Butte, Wyoming. Bulletin of the Geological Survey of America 45, 425–440.

Dorf, E., Cooper, J.R., 1943. Early Devonian plants from Newfoundland. Journal of Paleontology 17, 264–270.

Dorf, E., Rankin, D., 1962. Early Devonian plants from the Traveler Mountain area, Maine. Journal of Paleontology 36, 999–1004.

Dorofeev, P.I., 1963a. , Tretichnye Flory Zapadnoi Sibiri (Tertiary Floras of Western Siberia). Izdat. Akad. Nauk, Moscow/Leningrad. 343 pp.

Dorofeev, P.I., 1963b. , Tretichnye Flory Zapadnoi Sibiri (Tertiary Floras of Western Siberia). Izdat. Akad. Nauk, Moscow/Leningrad. 343 pp.

Dorofeev, P.I., 1963c. , Tretichnye Flory Zapadnoi Sibiri (Tertiary Floras of Western Siberia). Izdat. Akad. Nauk, Moscow/Leningrad. 343 pp.

Dorofeev, P.I., 1963d. , Tretichnye Flory Zapadnoi Sibiri (Tertiary Floras of Western Siberia). Izdat. Akad. Nauk, Moscow/Leningrad. 343 pp.

Doubinger, J., 1956. Contribution a l’etude des flores Autuno-Stephaniennes. Memoires de la Societe Geologique de France (Nouvelle Serie) 35, 1–180.

Doubinger, J., Marocco, R., 1981. Contenu palynologique de Groupe Copacabana (Permien Interieur et Moyen) sur la bordure sud de la Cordillere de Vilcabamba, Region de Cuzco (Perou). Geologische Rundschau 70, 1086–1099.

Doubinger, J., Roy-Dias, C., 1986. La Paleoflore Autuno-Stephanienne de la région de Demnate (est de Marrakech-Maroc). Géobios 19, 549–571.

Dou, Y.W., Sun, Z.H., 1983. Devonian Plants, Palaeontological Atlas of Xinjiang, Vol. II. Late Palaeozoic Section. Geological Publishing House, Beijing.

Dronov, V.I., Andreeva, T.F., 1962. Stratigraphy of the Jurassic deposits of Central and South-Eastern Pamir. Papers of Soviet geologists for the First International Colloquium on Jurassic system. Stratigraphy of the Jurassic System. 343–358.

Durante, M.V., 1976. The Carboniferous and Permian Stratigraphy of Mongolia on the Basis of Palaeobotanical Data. The Joint Soviet Mongolian Scientific-Research Geological Expedition Transactions 19, 34–92.

Dutra, T.L., Batten, D.J., 2000. Upper Cretaceous floras of King George Island, West Antarctica, and their palaeoenvironmental and phytogeographic implications. Cretaceous Research 181–209. doi:10.1006/cres.2000.0221

Dybova-Jachowicz, S., 1974. Analyse palynologique des sediments rouge saliferes du Zechstein Superieur (“Zouber” Rouge) a Klodawa, Pologne. Review of Palaeobotany and Palynology 17, 57–61.

Dzhinaridze, N.M., 1966. On the age of the Dzhaskoi suite of the Zhigansk region. Trudy VNIGRI. Geology and oil/gas resources of the Western Yakutiya. 249, 129–136.

Eberth, D.A., Russell, D.A., Braman, D.R., Deino, A.L., 1994. The age of the dinosaur-bearing sediments at Tebch, Inner Mongolia, People’s Republic of China. Canadian Journal of Earth Sciences 30, 2101–2106.

Ecke, H.H., 1986. Palynologie des Zechsteins und Unteren Buntsandsteins im Germanischen Becken.

Edelman, D.W., 1975. The Eocene Germer Basin Flora of South-Central Idaho.

Edwards, D., 1972. A Zosterophyllum fructification from the Lower Old Red Sandstone of Scotland. Review of Palaeobotany and Palynology 14, 77–83.

Edwards, D., 1970. Fertile Rhyniophytina from the Lower Devonian of Britain. Palaeontology 13, 451–461.

Edwards, D., 1969. Zosterophyllum from the lower Old Red Sandstone of South Wales. New Phytologist 68, 923–931.

Edwards, D., Richardson, J.B., 1974. Lower Devonian (Dittonian) plants from the Welsh Borderland. Palaeontology 17, 311–324.

Edwards, W.N., 1934. Jurassic plants from New Zealand. Annals and Magazine of Natural History 10, 81–109.

Eggert, D.A., Kanemoto, N.Y., 1977. Stem Phloem of a Middle Pennsylvanian Lepidodendron. Botanical Gazette 138, 102–111.

Ehiro, M., Zakharov, Y.D., Minjin, C., 2006. Early Triassic (Olenekian) ammonoids from Khentey Province, Mongolia, and their paleobiogeographic significance. Bulletin of the Tohoku University Museum 5, 83–97.

Eisawi, A.A.M., Ibrahim, A.B., Rahim, O.B.A., Schrank, E., 2012. Palynozonation of the Cretaceous to Lower Paleogene Strata of the Muglad Basin, Sudan. Palynology 36, 191–207.

El-Khayal, A.A., Chaloner, W.G., Hill, C.R., 1980. Paleozoic plants from Saudi Arabia. Nature 285, 33–34.

El-Khayal, A.A., Wagner, R.H., 1985. Upper Permian stratigraphy and megafloras of Saudi Arabia: palaeogeographic and climate implications. Compte Rendu Congres International de Stratigraphie et de Geologie du Carbonifere 3, 17–26.

Ellis, C.H., Tschudy, R.H., 1964. The Cretaceous megaspore genus Arcellites Miner. Micropaleontology 10, 73–79.

Emilian Popa, M., J Cleal, C., 2012. Aspects of Romanian Palaeozoic Palaeobotany and Palynology. Part III. The Late Carboniferous flora of Baia Nouă, Sirinia Basin. Geologia Croatica 65, 329–343.

Endo, R., 1940. A new genus of Thecodontia from the Lycoptera Beds in Manchoukuo. Bulletin of the Central National Museum of Manchoukuo 2, 1–14.

Erickson, B.R., 1991. Flora and Fauna of the Wannagan Creek Quarry: Late Paleocene of North America. Scientific Publications of the Science Museum of Minnesota 7, 1–19.

Erickson, B.R., 1982. Wannaganosuchus, a new alligator from the Paleocene of North America. Journal of Paleontology 56, 492–506.

Ermolaev, D.I., Teslenko, Y.V., 1964. Paleobotanical materials on the stratigraphy of the Jurassic deposits of the Irkutsk coal basin. Doklady Akademii Nauk SSSR 155, 562–564.

Eshet, Y., 1990. Paleozoic-Mesozoic Palynology of Israel: I: Palynological Aspects of the Permo-Triassic Succession in the Subsurface of Israel. Geological Survey of Israel 81, 1–57.

Evans, S.E., Manabe, M., Cook, E., Hirayama, R., Isaji, S., Nicholas, C.J., Unwin, D., Yabumoto, Y., 1998. An Early Cretaceous assemblage from Gifu Prefecture, Japan. Lower and Middle Cretaceous Terrestrial Ecosystems, New Mexico Museum of Natural History and Science Bulletin 14, 183–186.

Evans, S.E., Milner, A.R., 1994. Middle Jurassic microvertebrate assemblages from the British Isles, In the Shadow of the Dinosaurs: Early Mesozoic Tetrapods, N. C. Fraser and H.-D. Sues (eds.), Cambridge University Press.

Fairon-Demaret, M., 1996. Dorinnotheca streelii Fairon-Demaret, gen. et sp. nov., a new early seed plant from the upper Famennian of Belgium. Review of Palaeobotany and Palynology 93, 217–233.

Farabee, M.J., Taylor, E.L., Taylor, T.N., 1990. Correlation of Permian and Triassic palynomorph assemblages from the central Transantartic Mountains, Antartica. Review of Palaeobotany and Palynology 65, 257–265.

FARAHIMANESH, M., GERRIENNE, P., PRESTIANNI, C., GALTIER, J., 2011. Study of the flora preserved in coal balls of Bouxharmont from the Lower Pennsylvanian of Belgium. Miscellanea palaeontologica 2011 6.

Farke, A.A., Ryan, M.J., Barrett, P.M., Tanke, D.H., Braman, D.R., Loewen, M.A., Graham, M.R., 2011. A new centrosaurine from the Late Cretaceous of Alberta, Canada, and the evolution of parietal ornamentation in horned dinosaurs. Acta Palaeontologica Polonica 56, 691–702. doi:10.4202/app.2010.0121

Fasola, A., 1969. Estudio palinológico de la Formación Loreto (Terciario Medio), Provincia de Magallanes, Chile. Ameghiniana VI, 3–49.

Fefilova, L.A., 1968. Euramerican ferns from the Permian sediments of the Pechora Preurals. Doklady Akademii Nauk SSSR 183, 680–683.

Ferguson, D.K., Lee, D.E., Bannister, J.M., Zetter, R., Jordan, G.J., Vavra, N., Mildenhall, D.C., 2010. The taphonomy of a remarkable leaf bed assemblage from the Late Oligocene–Early Miocene Gore Lignite Measures, southern New Zealand. International Journal of Coal Geology 83, 173–181. doi:10.1016/j.coal.2009.07.009

Field, M.H., 1999. Variations in Azolla section Azolla megaspore apparatus and their implications for palaeotaxonomy and European Pleistocene biostratigraphy. Review of Palaeobotany and Palynology 105, 85–92.

Fijalkowska, A., 1994. Palynological aspects of the Permo-Triassic succession in the Holy Cross Mountains, Poland. Documenta Naturae 87, 1–76.

Flint, J.C.E., Gould, R.E., 1975. A note on the fossil megafloras of the Nymboida and Red Cliff Coal Measures, Southern Clarence-Moreton Basin, NSW. Journal and Proceedings of the Royal Society of New South Wales 108, 70–74.

Fontaine, W.M., 1889. The Potomac or Younger Mesozoic Flora. Monographs of the United States Geological Survey 15, 1–377.

Foster, C.B., Harris, W.K., 1981. Azolla capricornica sp. nov. first Tertiary record of Azolla Lamarck (Salviniaceae) in Australia. Transactions of the Royal Society of South Australia 105, 195–204.

Fowler, K., 1975. Megaspores and massulae of Azolla prisca from the Oligocene of the Isle of Wright. Palaeontology 18, 483–507.

Fraser, N.C., Grimaldi, D.A., Olsen, P.E., Axsmith, B., 1996. A Triassic Lagerstatte from eastern North America. Nature 380, 615–619.

Fraser, N.C., Olsen, P.E., Dooley, A.C., Ryan, T.R., 2007. A New Gliding Tetrapod (Diapsida: Archosauromorpha) from the Upper Triassic (Carman) of Virginia. Journal of Vertebrate Paleontology 27, 261–265.

Freeman, E.P., 1976. Mammal teeth from the Forest Marble (Middle Jurassic) of Oxfordshire, England. Science 194, 1053–1055.

Freile, C., 1972. Estudio Palinológico de la Formación Cerro Dorotea (Maestrichtiano-Paleoceno) de la Provincia de Santa Cruz. I. Revista del Museo de la Plata (nueva serie), Sección Paleontología 6, 39–63.

Frenguelli, J., 1953. La Flora Fósil de la región del Alto Río Chalia en Santa Cruz (Patagonia). Paleontología. Notas del museo XVI, 239–257.

Frenguelli, J., 1944. Contribuciones al conocimiento de la flora del Gondwana superior en la Argentina, XV. Notas del Museo de La Plata, Palaeontologia 9, 271–310.

Frenguelli, J., 1941. Nuevos elementos florísticos del Magellaniano de Patagonia Austral. Notas del Museo de La Plata. Paleontología VI, 173–210.

Fry, W.L., 1954. A study of the Carboniferous lycopod, Paurodendron, gen. nov. American Journal of Botany 41, 415–428.

Fujian Stratigraphic Group, 1979. The Stratigraphic Tables of Fujian, Geology Press.

Galtier, J., 1997. Coal-ball floras of the Namurian-Westphalian of Europe. Review of Palaeobotany and Palynology 95, 51–72.

Galtier, J., WANG, S.-J., LI, C.-S., Hilton, J., 2001. A new genus of filicalean fern from the Lower Permian of China. Botanical Journal of the Linnean Society 137, 429–442.

Gandolfo, M.A., Nixon, K.C., Crepet, W.L., Ratcliffe, G.E., 1997. A new fossil fern assignable to Gleicheniaceae from Late Cretaceous sediments of New Jersey. American Journal of Botany 84, 483–193.

Gansu Stratigraphic Group, 1980. The Stratigraphic Tables of Gansu, Geology Press.

Gao, K.-Q., Fox, R.C., -F. Zhou, C., -Q. Li, D., 2010. A new nonmammalian eucynodont (Synapsida: Therapsida) from the Triassic of northern Gansu Province, China, and its biostratigraphic and biogeographic implications. American Museum Novitates 3685, 1–25.

Gao, L., 1981. Devonian Spore Assemblages of China. Review of Palaeobotany and Palynology 34, 11–23.

Gee, C.T., 1989. Permian Glossopteris and Elatocladus megafossil floras from the English Coast, eastern Ellsworth Land, Antarctica. Antarctic Science 1, 35–44.

Gee, C.T., 1985. A Late Jurassic flora from the Orville Coast-eastern Ellsworth Land region of the Antarctic Peninsula. American Journal of Botany 72, 894–894.

Geng, B.-Y., 1992. Studies on Early Devonian flora of Sichuan. Acta Phytotaxonomica Sinica 30, 197–211.

Geng, B.Y., 1985. Huia recurvata -- A new plant from the Lower Devonian of southeastern Yunnan, China. Acta Botanica Sinica 27, 419–426.

Genkina, R.Z., 1966. Fossil flora and stratigraphy of the Lower Mesozoic deposits of Issyk-Kul’ basin (North Kirgizia).

Genkina, R.Z., 1960. Fossil flora and stratigraphy of the coal deposits of the North Sos’va basin. Izvestiya Akademii Nauk SSSR. Seria geologicheskaya 10, 70–76.

Gensel, P.G., 2002. Inventory of Devonian Plants from New Brunswick.

Gensel, P.G., 1982. Oricilla, A New Genus Referable to the Zosterophyllophytes from the Late Early Devonian of Northern New Brunswick. Review of Palaeobotany and Palynology 37, 345–359.

Gensel, P.G., 1979. Two Psilophyton species from the Lower Devonian of Eastern Canada with a discussion of morphological variation within the genus. Palaeontographica Abteilung B 168, 81–99.

Gensel, P.G., Andrews, H.N., Forbes, W.H., 1975. A New Species of Sawdonia with Notes on the Origin of Microphylls and Lateral Sporangia. Botanical Gazette 136, 50–62.

Gensel, P.G., Barnett-Lawrence, M., 1996. Plant megafossils from the Escuminac Formation, Devonian Fishes and Plants of Miguasha, Quebec, Canada.

Gerasimov, P.A., 1972. Stratigraphy of the USSR. Jurassic System.

Gerrienne, P., 1997. The fossil plants from the Lower Devonian of Marchin (northern margin of Dinant Synclinorium, Belgium). V. Psilophyton genseliae sp. nov., with hypotheses on the origin of Trimerophytina. Review of Palaeobotany and Palynology 303–324.

Gerrienne, P., 1995. Les Fossiles Vegetaux Du Devonien inferieur de Marchin (Bord Nord du synclinorium de Dinant, Belgique). III. Psilophyton Parvulum nov. sp. Géobios 28, 131–144.

Gerrienne, P., 1993. Inventaire Des Vegetaux Eodevoniens de Belgique. Annales dela Societe geologique de Belgique 116, 105–117.

Gerrienne, P., 1988. Early Devonian plant remains from Marchin (North of Dinant Synclinorium, Belgium), I. Zosterophyllum deciduum sp. nov. Review of Palaeobotany and Palynology 317–335.

Gerrienne, P., 1983. Les Plantes Emsiennes de Marchin (Vallee Du Hoyoux, Belgique). Annales de la Societe de Belgique 106, 19–35.

Geyer, G., Kleber, K.P., 1987. Flügelreste und Lebensspuren von Insekten aus dem Unteren Keuper Mainfrankens. Neues Jahrbuch für Geologie und Paläontologie, Abhandlungen 174, 331–355.

Gillespie, W.H., 1983. Plant Megafossils from the Carboniferous of Georgia, U.S.A. Dixième Congrès International de Stratigraphie et de Gèologie du Carbonifère 2, 247–256.

Gillespie, W.H., Clendening, J.A., 1962. A Lower Kittanning Flora from Northern West Virginia. Proceedings of West Virginia Academy of Science 34, 125–132.

Gillespie, W.H., Crawford, T.J., Waters, J.A., 1989. Plant Fossils of the Pennsylvanian System of Georgia, Guidebook Addendum: 38th Annual Meeting, Southeastern Section, The Geological Society of America.

Gillespie, W.H., Hennen, G.J., Balasco, C., 1975. Plant Megafossils from Dunkard Strata in Northwestern West Virginia and Southwestern Pennsylvania. Proceedings of the First I.C. White Memorial Symposium “The Age of the Dunkard.”

Gillespie, W.H., Pfefferkorn, H.W., 1986. Taeniopterid lamina on Phasmatocycas megasporophylls (Cycadales) from the Lower Permian of Kansas, U. S. A. Review of Palaeobotany and Palynology 49, 99–116.

Gillespie, W.H., Pfefferkorn, H.W., 1984. Plant Fossils of the New River Gorge, West Virginia. Proceedings of the West Virginia Academy of Science 56, 124–135.

Gillespie, W.H., Pfefferkorn, H.W., 1976. Plant Fossils in Early & Middle Parts of the Proposed Pennsylvanian System Stratotype in West Virginia, G.S.A. Field Trip Guidebook No. 3.

Girotti, O., Capasso Barbato, L., Esu, D., Gliozzi, E., Kotsakis, T., Martinetto, E., Petronio, C., Sardella, R., Squazzini, E., 2003. The section of Torre Picchio (Terni, Umbria, Central Italy): A Villafranchian site rich in vertebrates, molluscs, ostracods and plants. Rivista Italiana di Paleontologia e Stratigrafia 109, 77–98.

Gladenkov, Y.B., Bratseva, G.M., Mitrofanova, L.I., Sinel’nikova, V.N., 1988. Raschleneniye oligotsen-nizhnemiotsenovykh tolshch vostochnoy Kamchatki (zaliv Korfa). Subdivision of Oligocene-lower Miocene layers of eastern Kamchatka, Gulf of Korfa. Izvestiya Akademii Nauk SSSR. Seriya Geologicheskaya 1988, 3–16.

Golubeva, I.I., 1988. Permskii otlozheniia Vilyviskoi sineklizi (Permian deposits of the Vilvi Syncline). Sovetskaya Geologiya 4, 106–117.

Gomez, A., Jaramillo, C., Parra, M., Mora, A., 2009. Huesser Horizon: A lake and marine incursion in Northwestern South America during the Early Miocene. Palaios 24, 199–210. doi:10.2110/palo.2007.p07-074r

Gomolitskii, N.P., 1990. Flora and stratigraphy of the Aktash suite of Sogutty. Uzbekskii Geologicheskii Zhurnal (Uzbek Geological Journal). 3–7.

Gomolitskii, N.P., 1972. Subdivision of the Jurassic continental deposits of Middle Asia based on paleobotanic data. Sovetskaya Geologiya 124–130.

Gomolitskii, N.P., 1968. On stratigraphy of Jurassic continental deposits of the Yakkabag Mountain (Middle Asia). Izvestiya Akademii Nauk SSSR. Seria geologicheskaya 110–115.

Gomolitskii, N.P., Khudaiberdyev, R.K., 1978. About the Middle Asia Jurassic flora. The Palaeobotanist 25, 104–108.

Gomolitskii, N.P., Lobanova, A.V., 1969. On stratigraphy of the Jurassic deposits of Angren. Sovetskaya Geologiya 110–115.

Gonzalez, C.C., Gandolfo, M.A., Zamaloa, M.C., Cúneo, N.R., Wilf, P., Johnson, K.R., 2007. Revision of the Proteaceae macrofossil record from Patagonia, Argentina. The Botanical Review 73, 235–266.

González-Guzmán, A.E., 1967. A palynological study on the upper Los Cuervos and Mirador formations (Lower and middle Eocene; Tibu area, Colombia).

Good, C.W., 1975. Pennsylvanian-Age Calamitean Cones, Elater-Bearing Spores, and Associated Vegetative Organs. Palaeontographica 153, 28–99.

Good, C.W., 1971. The Ontogeny of Carboniferous Articulates: Calamite Leaves and Twigs. Palaeontographica 133, 137–158.

Gorelova, S.G., 1982. Fitostratigrafiya verkhnepaleozoiskikh otlozhenii Gorlovskogo Basseina [Phytostratigraphy of the Upper Paleozoic deposits of Gorlovskii Basin]. Sovetskaya Geologiya 7, 57–62.

Goswami, S., Singh, K.J., Chandra, S., 2006. Palaeobotany of Gondwana basins of Orissa State, India: A bird’s eye view. Journal of Asian Earth Sciences 28, 218–233.

Gould, R.E., 1974. The fossil flora of the Walloon Coal Measures: a survey. Proceedings of the Royal Society of Queensland 85, 33–41.

Graham, A., 1993. Contribution toward a Tertiary palynostratigraphy for Jamaica: The status of Tertiary paleobotanical studies in northern Latin America and preliminary analysis of the Guys Hill Member (Chapelton Formation, middle Eocene) of Jamaica, in Wright, R. M., and Rbinson, E. eds., Biostratigraphy of Jamaica: Boulder, Colorado, Geological Society of America Memoir 182.

Graham, A., 1991. Studies in Neotropical Paleobotany. X. The Pliocene communities of Panama - Composistion, numerical representations, and paleocommunity paleoenvironmental reconstructions. Annals of the Missouri Botanical Garden 78, 465–475.

Graham, A., 1989. Studies in neotropical paleobotany. VII. The Lower Miocene communities of Panama - the La Boca Formation. Annals of the Missouri Botanical Garden 76, 50–66.

Graham, A., 1988a. Studies in Neotropical Paleobotany. VI. The Lower Miocene Communities of Panama - The Cucaracha Formation. Annals of the Missouri Botanical Garden 75, 1467–1479.

Graham, A., 1988b. Studies in Neotropical Paleobotany. V. The Lower Miocene Communities of Panama - The Culebra Formation. Annals of the Missouri Botanical Garden 75, 1440–1466.

Graham, A., 1987. Miocene communities and paleoenvironments of Southern Costa Rica. American Journal of Botany 74, 1501–1518.

Graham, A., 1963. Systematic revision of the Sucker Creek and Trout Creek Miocene floras of southeastern Oregon. American Journal of Botany 921–936.

Graham, A., Cozadd, D., Areces-Mallea, A., Frederiksen, N.O., 2000. Studies in neotropical paleobotany. XIV. A palynoflora from the Middle Eocene Saramaguacan Formation of Cuba. American Journal of Botany 87, 1526–1539.

Graham, A., Gregory-Wodzicki, K.M., Wright, K.L., 2001. Studies in neotropical paleobotany. XV. A Mio-Pliocene palynoflora from the eastern cordillera, Bolivia: Implications for the uplift history of the Central Andes. American Journal of Botany 88, 1545–1557.

Graham, A., Jarzen, D.L., 1969. Studies in neotropical paleobotany. I. The Oligocene communities of Puerto Rico. Annals of the Missouri Botanical Garden 56, 308–357.

Graham, A., Stewart, R.H., Stewart, J.L., 1985. Studies in Neotropical Paleobotany. III. The Tertiary communities of Panama - Geology of the pollen-bearing deposits. Annals of the Missouri Botanical Garden 72, 485–503.

Grebe, H., Schweitzer, H.J., 1964. Die Sporae dispersae des niederrheinischen Zechsteins. Fortschritte in der Geologie von Rheinland und Westfalen 12, 201–224.

Greggs, R.G., McGregor, D.C., Rouse, G.E., 1962. Devonian plants from the Type section of the Ghost River formation fo Western Alberta. Science 135, 930–931.

Gregory, W.A., Hart, G.F., 1995. Distribution of pollen and spores in a subsurface marine Wilcox (Paleocene-Eocene) section in southwest Louisiana. Palynology 19, 1–43.

Grierson, J.D., Banks, H.P., 1963. Lycopods of the Devonian of New York State. Palaeontographica Americana 4, 221–295.

Grierson, J.D., Hueber, F.M., 1968. Devonian Lycopods from northern New Brunswick, International Symposium on the Devonian System, Oswald, D. H., (Ed.) Alberta Soc. Petroleum Geol., Calgary.

Griffing, D.H., Bridge, J.S., Hotton, C.L., 2001. Coastal-fluvial palaeoenvironments and plant palaeoecology of the Lower Devonian (Emsian), Gaspe Bay, Quebec, Canada, P. F. Friend, B. P. J. Williams, (Editors), New Perspectives on the Old Red Sandstone, Geological Society, London, Special Publications.

Grigorescu, D., Venczel, M., Csiki, Z., Limberea, R., 1999. New latest Cretaceous microvertebrate fossil assemblages from the Hateg Basin (Romania). Geologie en Mijnbouw 78, 301–314.

Gromov, V.V., Lebedev, E.L., Stavtsev, A.L., 1980. Geological structure of Ul’insk depression (Okhotsk belt). Sovetskaya Geologiya 74–85.

Guex, J., 1978. Le Trias inférieur des Salt Ranges (Pakistan): problèmes biochronologiques. Eclogae Geologicae Helvetiae 71, 105–141.

Gunther, P., Hills, L.V., 1970. Heterospory in Ariadnaesporites. Pollen et Spores 12, 123–130.

Gunther, P.R., Hills, L.V., 1972. Megaspores and other palynomorphs of the Brazeau Formation (Upper Cretaceous), Nordegg Area, Alberta. Geoscience and Man 4, 29–48.

Guppy, D.J., Lindner, A.W., Rattigan, J.H., Casey, J.N., 1958. The Geology of the Fitzroy Basin, Western Australia. Bureau of Mineral Resources, Geology and Geophysics Bulletin 36, 1–116.

Guskov, V.A., Pukhonto, S.K., Yatzuk, N.E., 1980. Verkhnepermskie otlozheniya Severo-Vostochnogo Pay-Khoya (Upper Permian deposits of northeast Pay-Khoy). Sovetskaya Geologiya 2, 68–75.

Habib, D., 1970. Middle Cretaceous palynomorph assemblages from clays near the Horizon Beta deep-sea outcrop. Micropaleontology 16, 345–379.

Habib, D., 1969. Middle Cretaceous palynomorphs in a deep-sea core from the Seismic Reflector Horizon A outcrop area. Micropaleontology 15, 1–4.

Haddoumi, H., Allain, R., Meslouh, S., Metais, G., Monbaron, M., Pons, D., -C. Rage, J., Vullo, R., Zouhri, S., Gheerbrant, E., 2016. Guelb el Ahmar (Bathonian, Anoual Syncline, eastern Morocco): first continental flora and fauna including mammals from the Middle Jurassic of Africa. Gondwana Research 29, 290–319. doi:10.1016/j.gr.2014.12.004

Halle, T.G., 1916. Lower Devonian Plants from Roragen in Norway. Kungl. Svenska Vetenskapsakademiens Handlingar 57.

Halle, T.G., 1913. Some Mesozoic plant-bearing deposits in Patagonia and Tierra del Fuego and their floras. Kungliga Svenska Vetenskapsakademiens Handlingar 51, 1–58.

Hall, J.W., 1975. Ariadnaesporites and Glomerisporites in the Late Cretaceous: ancestral Salviniaceae. American Journal of Botany 62, 359–369.

Hall, J.W., 1969. Studies on fossil Azolla: Primitive types of megaspores and massulae from the Cretaceous. American Journal of Botany 56, 1173–1180.

Hall, J.W., 1968. A new genus of Salviniaceae and a new species of Azolla from the late Cretaceous. American Fern Journal 58, 77–88.

Hall, J.W., 1967. Two new species of Ariadnaesporites. Pollen et Spores 9, 563–568.

Hall, J.W., 1963. Megaspores and other fossils in the Dakota Formation (Cenomanian) of Iowa, (U.S.A.). Pollen et Spores 5, 425–443.

Hall, J.W., Bergad, R.D., 1971. A critical study of three Cretaceous salviniaceous megaspores. micropaleontology 17, 345–356.

Hall, J.W., Peake, N.M., 1968. Megaspore assemblages in the Cretaceous of Minnesota. Micropaleontology 14, 456–464.

Hall, S.A., 1977. Cretaceous and Tertiary dinoflagellates from Seymour Island, Antarctica. Nature 267, 239–241.

Hankel, O., 1993. Early Triassic plant microfossils from Sakamena sediments of the Majunga Basin, Madagascar. Review of Palaeobotany and Palynology 77, 213–233.

Hankel, O., 1987. Lithostratigraphic subdivision of the Karoo rocks of the Luwegu Basin (Tanzania) and their biostratigraphic classification based on microfloras, macrofloras, fossil woods and vertebrates. Geologische Rundschau 76, 539–656.

Hao, S.G., 1992. Some observations on Zosterophyllum australianum Lang & Cookson from the Lower Devonian of Yunnan, China. Botanical Journal of the Linnean Society 109, 189–202.

Hao, S.-G., Gensel, P.G., 2001. The Posongchong floral assemblages of southeastern Yunnan, China -- Diversity and disparity in Early Devonian plant assemblages, Plants Invade the Land: evolutionary and environmental perspectives. Ed. P. G. Gensel and D. Edwards.

Hao, S.-G., Gensel, P.G., 1998. Some new plant finds from the Posongchong Formation of Yunnan, and consideration of a possible phytogeographic similarity between South China and Australia during the Early Devonian. Science in China, Series D 41, 1–13.

Hao, S.-G., Wang, D.-M., 2000. Two species of Zosterophyllum Penhallow (Z. australianum Lang and Cookson, Z. ramosum sp. nov.) from the Lower Devonian (Pragian) of southeastern Yunnan, China. Acta Palaeontologica Sinica 39, 26–41.

Harington, C.R., Ross, R.L.M., Matthewes, R.W., Stewart, K.M., Beattie, O., 2004. A late Pleistocene Stellar sea lion (Eumetopias jubatus) from Courtenay, British Columbia: its death, associated biota, and paleoenvironment. Canadian Journal of Earth Sciences 41, 1285–1297.

Harris, A.C., Raine, J.I., 2002. A sclerite from a late Cretaceous moth (Insecta: Lepidoptera) from Rakaia Gorge, Canterbury, New Zealand. Journal of the Royal Society of New Zealand 32, 457–462.

Harris, T.M., 1979. The Yorkshire Jurassic Flora V. Coniferales. British Museum (Natural History) 5, 1–150.

Harris, T.M., 1937. Stratigraphic relations of the plant beds, The Fossil Flora of Scoresby Sound, East Greenland.

Hasegawa, Y., Manabe, M., Isaji, S., Ohkura, M., Shibata, I., Yamaguchi, I., 1995. Terminally resorbed iguanodontid teeth from the Neocomian Tetori Group, Ishikawa and Gifu Prefecture, Japan. Bulletin of the National Science Museum, Tokyo, Series C 21, 35–49.

Hastings, M., 1853. On the Tertiary Beds of Hordwell, Hampshire. Philosophical Magazine and Journal of Science, Fourth Series 6, 1–11.

Haubold, H., 1985. Stratigraphische Grundlagen des Stefan C und Rotliegenden im Thuringer Wald, Schriftenreihe fur Geologische Wissenschaften.

Haubold, H., Allen, A., Atkinson, T.P., Buta, R.J., Lacefield, J.A., Minkin, S.C., Relihan, B.A., 2005. Interpretation of the tetrapod footprints from the early Pennsylvanian of Alabama, Pennsylvanian Footprints in the Black Warrior Basin of Alabama.

Heads, S.W., Wang, Y., 2013. First fossil record of Melanoplus differentialis (Orthoptera: Acrididae: Melanoplinae). Entomological News 123, 33–37.

Heard, A., 1927. On Old Red Sandstone plants showing structure from Brecon (South Wales). Quarterly Journal of the Geological Society of London 85, 195–209.

Heilongjiang Stratigraphic Group, 1979. The Stratigraphic Tables of Heilongjiang, Geology Press.

Henderson, S.M.K., 1932. Notes on Lower Old Red Sandstone plants from Callander, Perthsire. Transactions of the Royal Society of Edinburgh 57, 277–285.

Herbst, R., 2015. The Osmundaceae (Filices) from the Cretaceous of South Africa: new species and revision.

Herbst, R., 1968. Las floras liasicas argentinas con consideraciones estratigraficas. Jdas. Geologicas Argentinas (Actas) 3, 145–162.

Herbst, R., 1966. Revision de la flora liasica de Piedra Pintada, Provincia de Neuquen, Argentina. Revista del Museo de La Plata, n.s. (Paleontologia) 30, 27–53.

Herbst, R., 1965. La flora fosil de la Formacion Roca Blanca, Provincia Santa Cruz, Patagonia, con consideraciones geologicas y estratigraficas. Opera Lilloana 12, 1–101.

Herbst, R., 1964. La flora liasica de la zona del Rio Atuel, Mendoza, Argentina. Revista del Asociacion Geologica Argentina 19, 108–131.

Herbst, R., Anzotegui, L.M., Jalfin, G., 1987. Estratigrafía, paleoambiente y dos especies de Salvinia Adanson (Filicopsida), del Mioceno superior de Salta, Argentina. Facena 7, 15–42.

Herbst, R., Melchor, R., Troncoso, A., 1998. Las Pteridophyta y el paleoambiente de la parte media de la formación La Ternera (triásico superior), en Quebrada La Cachivarita, III Región, Chile. Revista geológica de Chile 25, 85–107.

Herendeen, P.S., Magallon-Puebla, S., Lupia, R., Crane, P.R., Kobylinska, J., 1999. A prelimimary conspectus of the Allon flora from the Late Cretaceous (Late Santonian) of central Georgia, U.S.A. Annals of the Missouri Botanical Garden 89, 407–471.

Hernandez-Castillo, G.R., Stockey, R.A., Rothwell, G.W., 2006. Anemia quatsinoensis sp. nov. (Schizaeaceae), a permineralized fern from the Lower Cretaceous of Vancouver Island. International Journal of Plant Sciences 167, 665–674.

Hernandez, P.J., Azcarate, V., 1971. Estudio paleobotanico preliminar sobre restos de una tafoflora de la Peninsula Byers (Cerro Negro); Islas Shetland del Sur, Antartica. Serie Cientifica Instituto Antartico Chileno 2, 15–50.

Herngreen, G.F.W., 1975. Palynology of the Middle and Upper Cretaceous strata in Brazil. Medelingen Rijks Geologische Dienst, Nieuwe Serie 26, 39–91.

Herngreen, G.F.W., 1973. Palynology of the Albian-Cenomanian strata of Borehole 1-QS-1-MA, State of Maranhao, Brazil. Pollen et Spores 15, 515–555.

Hess, D., et al., 1990. Floral community variation in basal Pennsylvanian strata overlying paleokarst on mid-Mississippian age St. Louis Limestone, McClure Quarry, Tennessee, McDonough County, Illinois. North-central Section, Geological Society of America, Abstracts with Programs 22, 5.

Hickey, L.J., 1977. Stratigraphy and paleobotany of the Golden Valley Formation (Early Tertiary) of western North Dakota. The Geological Society of America Memoir 150, 1–183.

Hickey, L.J., Johnson, K.R., Dawson M.R., 1988. The Stratigraphy, sedimentology and fossils of the Haughton Formation: a post-impact crater-fill, Devon Island, N.W.T., Canada. Meteoritics 23, 221–231.

Hill, C.R., Moore, D.T., Greensmith, J.T., Williams, R., 1985. Palaeobotany and petrology of a Middle Jurassic ironstone bed at Wrack Hills, North Yorkshire. Proceedings of the Yorkshire Geological Society 45, 277–292.

Hill, R.S., 1991. Leaves of Eucryphia (Eucryphiaceae) from tertiary sediments in south-eastern Australia. Australian Systematic Botany 4, 481–497. doi:10.1071/SB9910481

Hill, R.S., 1987. Tertiary Isoetes from Tasmania. Alcheringa 12, 157–162. doi:10.1080/03115518808619003

Hill, R.S., 1984. Tertiary Nothofagus macrofossils from Cethana, Tasmania. Alcheringa 8, 81–86. doi:10.1080/03115518408619610

Hill, R.S., 1982. The Eocene Megafossil Flora of Nerriga. New South Wales, Australia. Palaeontographica Abteilung B 181, 44–77.

Hill, R.S., Carpenter, R.J., 1991. Evolution of Acmopyle and Dacrycarpus (Podocarpaceae) Foliage as Inferred from Macrofossils in South-eastern Australia. Australian Systematic Botany 4, 449–479. doi:10.1071/SB9910449

Hill, R.S., Macphail, M.K., 1985. A fossil flora from rafted Plio Pleistocene mudstones at Regatta Point, Tasmania. Australian Journal of Botany 33, 497–517.

Hill, R.S., Macphail, M.K., 1983. Reconstruction of the Oligocene vegetation at Pioneer, northeast Tasmania. Alcheringa 7, 281–299. doi:10.1080/03115518308619613

Hills, L.V., Gopal, B., 1967. Azolla primaeva and its phylogenetic significance. Canadian Journal of Botany 45, 1179–1191.

Hills, L., Weiner, N., 1965. Azolla geneseana, n. sp., and revision of Azolla primaeva. micropaleontology 11, 255–261.

HILTON, J., Shi-Jun, W., GALTIER, J., GLASSPOOL, I., STEVENS, L., 2004. An Upper Permian permineralized plant assemblage in volcaniclastic tuff from the Xuanwei Formation, Guizhou Province, southern China, and its palaeofloristic significance. Geological Magazine 141, 661–674.

Hilton, J., Wang, S.-J., Galtier, J., -S. Li, C., 2001. An Early Permian plant assemblage from the Taiyuan Formation of northern China with compression/impression and permineralized preservation. Review of Palaeobotany and Palynology 114, 175–189.

Hocknull, S.A., Cook, A.G., 2008. Hypsilophodontid (Dinosauria: Ornithischia) from latest Albian, Winton Formation, central Queensland. Memoirs of the Queensland Museum 52, 212.

Hoeg, O.A., 1942. The Downtonian and Devonian Flora of Spitsbergen, Skrifter.

Hoffman, G.L., Stockey, R.A., 1994. Sporophytes, megaspores, and massulae of Azolla stanleyi from the Paleocene Joffre Bridge locality, Alberta. Canadian Journal of Botany 72, 301–308.

Hollick, A., Berry, E.W., 1924. A Late Tertiary flora from Bahia, Brazil. The Johns Hopkins University Studies in Geology 5, 11–137.

Holman, J.A., Fisher, D.C., Kapp, R.O., 1986. Recent discoveries of fossil vertebrates in the lower peninsula of Michigan. Michigan Academician 18, 431–463.

Holmes, W., 2001. The Middle Triassic megafossil flora of the Basin Creek Formation, Nymboida coal measures, New South Wales, Australia. Part 2. Filicophyta, in: Proceedings of the Linnean Society of New South Wales. LINNEAN SOC NEW SOUTH WALES PO BOX 137, MATRAVILLE, NEW SOUTH WALES, 2036, AUSTRALIA, pp. 39–87.

Holmes, W.B.K., Ash, S.R., 1979. An Early Triassic megafossil flora from the Lorne Basin, New South Wales. Proceedings of the Linnean Society of New South Wales 103, 47–70.

Holmes, Wbk., others, 2003. The Middle Triassic Megafossil Flora of the Basin Creek Formation, Nymboida Coal Measures, New South Wales, Australia. Part 3. Fern-like Foliage, in: Proceedings of the Linnean Society of New South Wales. Linnean Society of New South Wales, p. 53.

Hope, R.C., Patterson, O.F., III, 1970. Pekinopteris auriculata: a new plant from the North Carolina Triassic. Journal of Paleontology 44, 1137–1139.

Hope, R.C., Patterson, O.F., III, 1969. Triassic flora from the Deep River Basin, North Carolina. North Carolina Department of Conservation and Development, Division of Mineral Resources Special Publication 2, 1–12.

Hradecka, L., Lobitzer, H., Ottner, F., Schlagintweit, F., Svobodova, M., Szente, I., Svabenicka, L., Zorn, I., 2005. Biostratigraphy and paleoenvironment of the lower Gosau subgroup of Eisenbach brook in Salzkammergut (Upper Austria). Beiträge zur Geologie des Gmundner Bezirks/Gmundner Geo-Studien 3, 25–42.

Hsu, J., 1966. On plant remains from the Devonian of Yunnan and their significance in the identification of the stratigraphical sequence of this region. Acta Botanica Sinica 14, 50–69.

Huang, B.H., 1986. The fossil plants of Elitu Formation at Xianghuang Qi (Banner) District, Nei Mongol and its significance, Contributions to the Project of Plate Tectonics in Northern China.

Huang, Q., Dilcher, D., 1994. Evolutionary and Paleoecological Implications of Fossil Plants from the Lower Cretaceous Cheyenne Sandstone of the Western Interior. Geological Society of America Special Paper 287, 129–144.

Huckriede, R., 1982. Die unterkretazische Karsthöhlen-Füllung von Nehden im Sauerland. 1. Geologische, paläozoologische und paläobotanische Befunde und Datierung [The Lower Cretaceous karst cave fill of Nehden in Sauerland. 1 Geological, paleozoological, and paleobotanical finds and dating]. Geologica et Palaeontologica 16, 183–242.

Hueber, F.M., 1983. A new species of Baragwanathia from the Sextant Formation (Emsian) Northern Ontario, Canada. Botanical Journal of the Linnean Society 86, 57–79.

Hueber, F.M., 1982. Megaspores and a Palynomorph from the Lower Potomac Group in Virginia. Smithsonian Contributions to Paleobiology 1–69.

Hueber, F.M., Grierson, J.D., 1961. On the Occurrence of Psilophyton princeps in the Early Upper Devonian of New York. American Journal of Botany 48, 473–479.

Huene, F. v., 1931. Die fossilen Fährten im Rhät von Ischugualasto in Nordwest-Argentinien [The fossil footprints in the Rhaetian of Ischigualasto in northwest Argentina]. Palaeobiologica 4, 99–112.

Hughes, N.F., 1955. Wealden Plant Microfossils. Geological Magazine 92, 201–217.

Hu, S., Dilcher, D.L., Schneider, H., Jarzen, D.M., 2006. Eusporangiate ferns from the Dakota Formation, Minnnesota, USA. International Journal of Plant Sciences 167, 579–589.

Hyatt, A., Smith, J.P., 1905. The Triassic cephalopod genera of America. United States Geological Survey Professional Paper 40, 1–394.

Iannuzzi, R., Pfefferkorn, H.W., 2002. A Pre-Glacial, Warm-Temperate Floral Belt in Gondwana (Late Visean, Early Carboniferous). Palaios 17, 571–590.

Ibrahim, M.I.A., 1996. Aptian-Turonian palynology of the Ghazalat-1 well (GTX-1), Qattara Depression, Egypt. Review of Palaeobotany and Palynology 94, 137–168.

Il’ina, V.I., Teslenko, Y.V., 1971. On the question of the boundary between the Lower and Middle Jurassic in continental units of Siberia. Akademia Nauk SSSR, Geologiya i Geofizika 3–10.

Istchenko, T.A., 1975. Pozdnesiluriiskaia Flora Podolii, Pozdnesiluriiskaia Flora Podolii.

Iwai, J., Hongnusonthi, A., Asama, K., Kobayashi, T., Konno, E., Nakornsri, N., Veeraburas, M., Yuyen, W., 1975. Non-marine Mesozoic Formations and Fossils in Thailand and Malaysia, Geology and Palaeontology of Southeast Asia.

Jaekel, O., 1910. Die Fussstellung und Lebensweise der grossen Dinosaurier [The foot posture and way of life of the large dinosaurs]. Zeitschrift der Deutschen Geologischen Gesellschaft 62, 270–277.

Jain, R.K., Hall, J.W., 1969. A contribution to the Early Tertiary fossil record of the Salviniaceae. American Journal of Botany 56, 527–539.

Jansonius, J., 1962. Palynology of Permian and Triassic sediments, Paece River Area, Western Canada. Palaeontographica Abteilung B 110, 35–98.

Jaramillo, C.A., Dilcher, D.L., 2001. Middle Paleogene palynology of Central Colombia, South America: A study of pollen and spores from tropical latitudes. Palaeontographica Abteilung B 258, 87–213.

Jardiné, S., Magloire, H., 1965. Palynologuie et stratigraphie du Crétacé des Bassin du Sénégal et Cote d’Ivoire. Mémoires du Bureau de Recherches Géologiques et Miniéres 32, 187–245.

J Cleal, C., M van Waveren, I., 2012. A reappraisal of the Carboniferous macrofloras of the Zonguldak–Amasra Coal Basin, north-western Turkey. Geologia Croatica 65, 283–298.

Jennings, J.R., 1984. Distribution of Fossil Plant Taxa in the Upper Mississippian and Lower Pennsylvanian of the Illinois Basin 2, 301–312.

Jennings, J.R., 1980. Fossil plants from the Fountain Formation (Pennsylvanian) of Colorado. Journal of Paleo. 54, 149–158.

Jiangsu Stratigraphic Group, 1978. The Stratigraphic Tables of Jiangsu, Geology Press.

Jiangxi Stratigraphic Group, 1980. The Stratigraphic Tables of Jiangxi, Geology Press.

Jilin Stratigraphic Group, 1978. The Stratigraphic Tables of Jilin, Geology Press.

Johnson, K.R., 2003. Denver Museum of Nature & Science archival records of Kirk Johnson fieldwork in Cretaceous rocks of the Rocky Mountain region.

Johnson, K.R., 2002. Megaflora of the Hell Creek and lower Fort Union Formations in the western Dakotas: Vegetational response to climate change, the Cretaceous-Tertiary boundary event, and rapid marine transgression, The Hell Creek Formation and the Cretaceous-Tertiary boundary in the northern Great Plains: An integrated continental record of the end of the Cretaceous.

Johnson, N.G., Gensel, P.G., 1992. A reinterpretation of the Early Devonian land plant, Bitelaria Istchenko and Istchenko, 1979, based on new material from New Brunswick, Canada. Review of Palaeobotany and Palynology 74, 109–138.

Jordan, G.J., Macphail, M.K., Barnes, R., Hill, R.S., 1995. An Early to Middle Pleistocene Flora of Subalpine Affinities in Lowland Western Tasmania. Australian Journal of Botany 43, 231–242. doi:10.1071/BT9950231

Josht, A., Tewari, R., Mehrotra, R., Chakraborty, P., De, A., 2003. Plant remains from the Upper Siwalik sediments of West Kameng District, Arunachal Pradesh. Geological Society of India 61, 319–324.

Jud, N.A., Rothwell, G.W., Stockey, R.A., 2008. Todea from the Lower Cretaceous of Western North America: implications for the phylogeny, systematics, and evolution of modern Osmundaceae. American Journal of Botany 95, 330–339.

Kalantar, I.Z., 1980. News on Stratigraphy of the Triassic deposits of the Polar PreUrals, News on Stratigraphy of the Triassic of the PaleoUrals.

Kalugin, A.K., Kirichkova, A.I., 1968. Stratigraphy of the Jurassic continental units of Mangyshlak. Byulleten’ nauchno-tekhnicheskoi informatsii. Seriya: Geologiya mestorozhdenii poleznykh iskopaemykh. Problem of oil and gas resourses of the Mangyshlak and Ustyurt. 15–23.

Karaszewski, W., 1965. O srodkowoliasowym wieku flory z Chmielowa pod Ostrowcem i jej znaczeniu dla stratygrafii kontynentalnej jury [On the Middle Liassic age of the flora from Chmielow, near Ostrowiec (central Poland) and its significance for stratigraphy of the continental Jurassic]. Kwartalnik Geologiczny 9, 261–270.

Karrow, P.F., Morris, T.F., McAndrews, J.H., Morgan, A.V., Smith, A.J., Walker, I.R., 2007. A diverse late-glacial (Mackinaw Phase) biota from Leamington, Ontario. Canadian Journal of Earth Sciences 44, 287–296.

Karrow, P.F., Seymour, K.L., Miller, B.B., Mirecki, J.E., 1997. Pre-Late Wisconsinan Pleistocene biota from southeastern Michigan, U.S.A. Palaeogeography, Palaeoclimatology, Palaeoecology 133, 81–101.

Kasper, A., Andrews, H., Forbes, W., 1974. New fertile species of Psilophyton from the Devonian of Maine. American Journal of Botany 61, 339–359.

Kazakov, A.M., Dagis, A.S., 1984. Stratigrafiya Triasa yuzhnogo Kharaulakha i severnogo Orulgana (Severnoe Verkhovanye) [Stratigraphy of theTriassic of southern Kharaulakh and northern Orulgan (northern Verkhovanye)]. Akademia Nauk SSSR, Sibirskoe Otdelenie, Instituta Geologii i Geophyziki, Trudy 689, 81–95.

Keating, J.M., 1992. Palynology of the Lachman Crags Member, Santa Marta Formation (Upper Cretaceous) of north-west James Ross Island. Antartic Science 4, 293–304.

Kelber, K.-P., van Konijnenburg-van Cittert, J.H.A., 1997. A new Rhaetian flora from the neighbourhood of Coburg (Germany) - preliminary results. Proceedings 4th EPPC 58, 105–114.

Kerey, I.E., Kelling, G., Wagner, R.H., 1985. An outline stratigraphy and palaeobotanical records from the middle Carboniferous rocks of northwestern Turkey. Extrait des Annales de la Societe Geologique du Nord 203–216.

Kermack, K.A., Mussett, F., Rigney, H.W., 1973. The lower jaw of Morganucodon. Zoological Journal of the Linnean Society of London 53, 87–175.

Kerp, J.H.F., Poort, R.J., Swinkels, H.A.J.M., Verwer, R., 1990. Aspects of Permian palaeobotany and palynology. IX. Conifer-dominated Rotliegend floras from the Saar-Nahe Basin (?Late Carboniferous-Early Permian; SW-Germany) with special reference to the reproductive biology of early conifers. Review of Palaeobotany and Palynology 62, 205–248.

Kershaw, A.P., 1976. A Late Pleistocene and Holocene pollen diagram from Lynch’s Crater, northeastern Queensland, Australia. New Phytologist 77, 469–498.

Kershaw, A.P., Bretherton, S.C., van der Kaars, S., 2007. A complete pollen record of the last 230 ka from Lynch’s Crater, north-eastern Australia. Palaeogeography, Palaeoclimatology, Palaeoecology 251, 23–45. doi:10.1016/j.palaeo.2007.02.015

Khoo, T.T., Yaw, B.S., Kimura, T., Kim, J.H., 1988. Geology and palaeontology of the Redang islands, Trengganu, Peninsular Malaysia. Journal of Southeast Asian Earth Sciences 2, 123–130.

Khuc, V., 2000. The Triassic of Indochina Peninsula and its interregional correlation, Permian-Triassic Evolution of Tethys and Western Circum-Pacific.

Khudolei, K.M., 1972. Stratigraphy of the USSR. Jurassic System.

Kimura, T., 1987. Recent knowledge of Jurassic and Early Cretaceous Floras in Japan and Phytogeography of this Time in East Asia. Bulletin of Tokyo Gakugei University Section IV 39, 87–115.

Kimura, T., Ohana, T., 1989. Late Jurassic Plants from the Oginohama Formation, Oshika Group in the Outer Zone of Northeast Japan (I). Bulletin of the National Science Museum, Tokyo, Series C 15, 1–24.

Kimura, T., Ohana, T., Tsujii, M., 1988. Early Jurassic plants in Japan. Part 8. Supplementary description and concluding remarks. Transactions and Proceedings of the Palaeontological Society of Japan 501–522.

Kimyai, A., 1966. New plant microfossils from the Raritan Formation (Cretaceous) in New Jersey. micropaleontology 12, 461–476.

Kirichkova, A.I., 1990. Triassic-Early Jurassic flora of the Eastern Urals. Paleontologicheskii Zhurnal (Paleontological Journal) 110–119.

Kirichkova, A.I., 1976. Paleobotanic characteristics and correlation of the Upper Jurassic continental deposits of Western Yakutiya. Geologiia i Geofizika 44–54.

Kirichkova, A.I., 1966. On the occurence of an Early Jurassic flora in the Eastern Siberia. Trudy VNIGRI. Geology and oil potential in the Western Yakutiya 249, 120–124.

Kirichkova, A.I., Baranova, Z.E., 1978. Development of the Jurassic floras of Southern Emba and Mangyshlak, Topics in Paleobiogeography.

Kirichkova, A.I., Kalugin, A.K., 1973. About the boundary between the Lower and Upper Jurassic on Mangyshlak. Doklady Akademii Nauk SSSR 213, 410–412.

Kirichkova, A.I., Samylina, V.A., 1984. Pecularities of paleofloristic characteristics of continental deposits of Upper Jurassic and Neocomian of Northern Siberia, Trudy Instituta Geologii i Geofiziki. Boundary stages of the Jurassic and Cretaceous systems.

Kirina, T.I., Kolesnikov, C.M., 1967. Biostratigraphy of interlayered continental and marine Mesozoic deposits of Eastern Siberia, Stratigrafiya i paleontologiya mezozoiskikh i paleogen-neogenovikh kontinental. Nikh otlozhenii Aziatskoi chasti.

Kirkland, J.I., Armstrong, H.J., 1992. Taphonomy of the Mygatt-Moore (M&M) Quarry, middle Brushy Basin Member, Morrison Formation (Upper Jurassic) western Colorado. Journal of Vertebrate Paleontology 12, 55A.

Klinger, H.C., Kennedy, W.J., 1979. Cretaceous faunas from Southern Africa. Lower Cretaceous ammonites, including a new bochianitid genus, from Umgazana, Transkei. Annals of the South African Museum 78, 11–19.

Klitzsch, E., Lejal-Nicol, A., 1984. Flora and fauna from strata in southern Egypt and northern Sudan (Nubia and surrounding areas). Berliner geowissenschaftliche Abhandlungen Reihe A 50, 47–79.

Klompmaker, A.A., Herngreen, G.F.W., Oosterink, H.W., 2010. Biostratigraphic correlation, paleoenvironment stress, and subrosion pipe collapse: Dutch Rhaetian shales uncover their secrets. Facies 56, 597–613. doi:10.1007/s10347-010-0227-z

Knowlton, F.H., 1916. A Lower Jurassic flora from the upper Matanuska Valley, Alaska. Proceedings of the U.S. National Museum 51, 451–460.

Knowlton, F.H., 1899. Fossil Flora of the Yellowstone National Park. USGS Monograph Series 32.

Kolosov, P.N., Ivensen, G.V., Mikhailova, T.E., Kurzanov, S.M., Efimov, M.B., Gubin, Y.M., 2009. Taphonomy of the Upper Mesozoic Tetrapod Teete Locality (Yakutia). Paleontological Journal 43, 201–207.

Komatsu, T., Watanabe, M., Ishida, N., 2009. Lower Creatceous bivalves form Gumizaki area, Kagoshima prefecture, Japan. Memoir of the Fukui Prefectural Dinosaur Museum 8, 11–29.

Kora, M., Beialy, S. El, 1989. Early Cretaceous palynomorphs from Gabal Musaba Salama area, Southwestern Sinai, Egypt. Review of Palaeobotany and Palynology 58, 129–138.

Kotyk, M.E.A., 1998. Late Silurian and Early Devonian fossil plants of Bathurst Island, Arctic Canada.

Kovach, W.L., Dilcher, D.L., 1988. Megaspores and other dispersed plant remains from the Dakota Formation (Cenomanian) of Kansas, U.S.A. Palynology 12, 89–119.

Koval’chuk, G.M., 1961a. Paleobotanical characteristics of the Lower Mesozoic deposits of the Alakul’ lake. Transactions of the Laboratory of Coal Geology. History of the Lower Mesozoic coal deposition in Kazakhstan. Part 1. 13, 216–224.

Koval’chuk, G.M., 1961b. Paleobotanical characteristics of the Lower Mesozoic deposits of the Karaganda basin. Academy of Sciences of the USSR. Laborotary of Coal Geology. Transactions. History of the Lower Mesozoic coal deposition in Kazakhstan. Part 2. Chapter 4 13, 67–85.

Kowalski, E.A., 2001. Middle to late Miocene environments of southern Ecuador: temperature, elevation and fossil plants of the Nabon Basin.

Krasilov, V.A., 1973. Materials on stratigraphy and paleoflora of coal unit in the Bureya basin, Fossil floras and phytostratigraphy of the Far East.

Krasilov, V.A., Shorokhova, S.A., 1973. Early Jurassic flora of Petrovka River, Fossil floras and phytostratigraphy of the Far East.

Krausel, R., 1959. Die Juraflora von Sassendorf bei Bamberg. Senckenbergiana lethaea 40, 97–136.

Krausel, R., Weyland, H., 1938. Neue Pflanzenfunde im Mitteldevon von Elberfeld. Palaeontographica Abteilung B 83, 172–195.

Krausel, R., Weyland, H., 1926. Beitrage zur Kenntnis der Devonflora V. Uber Material und Fundorte. Senckenbergische Naturforschende Gesellschaft Abhandlungen 40, 114–155.

Krausel, R., Weyland, H., 1923. Beitrage zur Kenntnis der Devonflora. I. Die Fundorte der beschriebenen Pflazen. Senckenbergiana 5, 154–184.

Krausel, V.R., Weyland, H., 1932. Pflanzenreste aus dem Devon III. Uber Hyenia Nath. Senckenbergiana 14, 274–280.

Krumenacker, L.J., 2010. Chronostratigraphy and Paleoecology of the mid-Cretaceous Wayan Formation of Eastern Idaho, with a description of the first Oryctodromeus specimens from Idaho.

Krystyn, L., Bhargava, O.N., Richoz, S., 2007. A candidate GSSP for the base of the Olenekian Stage: Mud at Pin Valley; district Lahul & Spiti, Himachal Pradesh (Western Himalaya), India. Albertiana 35, 5–20.

Kuhn, O., 1939. Beiträge zur Keuperfauna von Halberstadt [Contributions to the Keuper fauna of Halberstadt]. Palaeontologische Zeitschrift 21, 258–286.

Kurzanov, S.M., Efimov, M.B., Gubin, Y.M., 2003. New archosaurs from the Jurassic of Siberia and Mongolia. Paleontological Journal 37, 53–57.

Kustatscher, E., Bauer, K., Reich, M., 2012. A new Middle Triassic (Pelsonian) plant locality in the Non Valley (Trentino, Northern Italy). Geo.Alp 9, 60–73.

Kustatscher, E., Bizzarini, F., Roghi, G., 2011a. Plant fossils in the Cassian Beds and Other Carnian Formations of the Southern Alps (Italy). Geo.Alp 8, 146–155.

Kustatscher, E., Dellantonio, E., Van Konijnenburg-Van Cittert, J.H., 2014. The ferns of the late Ladinian, Middle Triassic flora from Monte Agnello, Dolomites, Italy. Acta Palaeontologica Polonica 59, 741–755.

Kustatscher, E., Giordano, D., Riva, A., 2011b. La flora anisica della Valle di San Lucano, in: Aldighieri, B., Testa, B. (Eds.), L’armonia fra uomo e natura nelle valli dolomitiche - Atti delle giornate di studio di Agordo, 12-13 novembre 2010.

Kustatscher, E., Kelber, K.-P., van Konijnenburg-van Cittert, J.H., 2012. Danaeopsis HEER ex SCHIMPER 1869 and its European Triassic species. Review of Palaeobotany and Palynology 183, 32–49.

Kustatscher, E., Van Konijnenburg-van Cittert, J.H.A., 2011. The ferns of the Middle Triassic flora from Thale (Germany). Neues Jahrbuch für Geologie und Paläontologie, Abhandlungen 261, 209–248.

Kustatscher, E., van Konijnenburg-van Cittert, J.H.A., 2008. Lycophytes and horsetails from the Triassic flora of Thale (Germany). Neues Jahrbuch für Geologie und Paläontologie, Abhandlungen 250, 65–77.

Kustatscher, E., Wachtler, M., KONIJNENBURG-VAN CITTERT, V., JOHANNA, H., 2007. Horsetails and seed ferns from the Middle Triassic (Anisian) locality Kühwiesenkopf (Monte Prá della Vacca), dolomites, northern Italy. Palaeontology 50, 1277–1298.

Kustatscher, E., Wachtler, M., van Konijnenburg-van Cittert, J.H.A., 2010. Lycophytes from the Middle Triassic (Anisian) Locality Kühwiesenkopf (Monte Prà Della Vacca) in the Dolomites. Palaeontology 53, 595–626.

Kustatscher, E., Wachtler, M., van Konijnenburg-van Cittert, J.H.A., 2004. A number of additional and revised taxa from the Ladinian flora of the Dolomites, Northern Italy. Geo.Alp 1, 57–69.

Kvaček, J., Vodrážka, R., 2016. Late Cretaceous flora of the Hidden Lake Formation, James Ross Island (Antarctica), its biostratigraphy and palaeoecological implications. Cretaceous Research 58, 183–201.

Kvacek, Z., Walther, H., 1998. The Oligocene Volcanic Flora of Kundratice near Litomerice, Ceske Stredohori Volcanic Complex (Czech Republic) - A review. Sbornik Narodniho Muzea v Praze, Rada B - Prirodni Vedy (Acta Musei Nationalis Pragae, Series B, Historia Naturalis) 54, 1–43.

Lacey, W.S., Eggert, D.A., 1964. A Flora from the Chester Series (Upper Mississippian) of Southern Illinois. American Journal of Botany 51, 976–985.

Lakhanpal, R.N., 1970. Tertiary floras of India and their bearing on the historical geology of the region. Taxon 675–694.

Lakhanpal, R.N., 1958. The Rujada Flora of west central Oregon. University of California Publications in Geological Sciences 35, 1–66.

Langston, W., Jr., 1976. A Late Cretaceous vertebrate fauna from the St. Mary River Formation in western Canada, Athlon: Essays on Palaeontology in Honour of Loris Shano Russell.

Langston, W., Jr., 1975. The ceratopsian dinosaurs and associated lower vertebrates from the St. Mary River Formation (Maestrichtian) at Scabby Butte, southern Alberta. Canadian Journal of Earth Sciences 12, 1576–1608.

Lang, W.H., 1937. On the plant-remains from the Downtonian of England and Wales. Philosophical Transactions of the Royal Society 227B, 245–291.

Lang, W.H., 1932. Contributions to the study of the Old Red Sandstone flora of Scotland. VIII. On Arthrostigma, Psilophyton, and some associated plant-remains from the Strathmore Beds of the Caledonian Lower Old Red Sandstone. Transactions of the Royal Society of Edinburgh 57, 491–521.

Lang, W.H., 1927. Contributions to the Study of the Old Red Sandstone Flora of Scotland. VI. On Zosterophyllum myretonianum, Penh., and some other plant-remains from the Carmyllie Beds of the Lower Old Red Sandstone. VII. On a specimen of Pseudosporochnus from the Stromness Beds. Transactions of the Royal Society of Edinburgh 55, 443–455.

Lapasha, C.A., Miller, C.N., 1985. Flora of the Early Cretaceous Kootenai Formation in Montana, Bryophytes and Tracheophytes Excluding Conifers. Palaeontographica Abteilung B 196, 111–145.

Lapparent, A.F. d., Claracq, P., Nougarède, F., 1958. Nouvelles découvertes de Vertébrés dans les séries continentales au Nord d’Edjeleh (Sahara central) [New discoveries of vertebrates in the continental series north of Edjeleh (central Sahara)]. Comptes Rendus de l’Académie des Sciences à Paris 247, 2399–2402.

Lawal, O., 1991. Palynological age and correlation of a black shale section in the Eze-Aku Formation, Lower benue Trough, Nigeria. Journal African Earth Sciences 12, 473–482.

Lawal, O., Moullade, M., 1986. Palynological biostratigraphy of Cretaceous sediments in the Upper Benue basin, NE. Nigeria. Revue de Micropaleontologie 29, 61–83.

Leary, R., 1974. Two Early Pennsylvanian Floras of Western Illinois. Transactions, Ill. State Acad. Sci. 67, 430–440.

Leary, R.L., 1981. Early Pennsylvanian geology and paleobotany of the Rock Island County, Illinois area. Part I. Geology. Illinois States Museum Repts. Invest.

Leary, R., Pfefferkorn, H.W., 1977. An Early Pennsylvanian Flora with Megalopteris and Noeggerathiales from West-Central Illinois, Illinois State Geological Survey.

Lebanidze, Z.A., 1988. New locality of the Bathonian flora in Georgia. Bulletin of the Academy of Sciences of the Georgian SSR 130, 361–364.

Lebedev, E.L., 1973. Jurassic plants of the Western Okhotsk Region. Paleontologicheskii Zhurnal (Paleontological Journal) 84–94.

Lebedev, E.L., 1965. Late Jurassic flora of Zeya River and the Jurassic-Cretaceous boundary, Transactions. Geological Institute.

Lebedev, E.L., 1963. Upper Jurassic flora of Zeya river and its significance for separation of Upper Jurassic and Lower Cretaceous continental deposits in Amur river basin. Doklady Akademii Nauk SSSR 150, 149–151.

Leclercq, S., 1969. Calamophyton primaevum: The complex morphology of its fertile appendage. American Journal of Botany 56, 773–781.

Leclercq, S., 1942. Quelques plantes fossiles recueillies dans le Devonien inferieur des environs de Nonceveux (Bordure orientale du bassin de Dinant). Annales de la Societe Geologique de Belgique 65, 193–211.

Leclercq, S., Andrews, H.N., 1960. Calamophyton bicephalum, A New Species from the Middle Devonian of Belgium. Annals of the Missouri Botanical Garden 47, 1–25.

Leclercq, S., Banks, H.P., 1962. Pseudosporochnus nodosus sp. nov., A Middle Devonian Plant with Cladoxylalean Affinities. Palaeontographica Abteilung B 110, 1–34.

Lee, D.E., Bannister, J.M., Lindqvist, J.K., 2007. Late Oligocene — Early Miocene leaf macrofossils confirm a long history of Agathis in New Zealand. New Zealand Journal of Botany 45, 565–578. doi:10.1080/00288250709509739

Lee, D.S., 1988a. Geology of Korea, Kyohak-Sa Publishing Company, Seoul, South Korea.

Lee, D.S., 1988b. Geology of Korea (Chapter 6: Mesozoic Erathem).

Lee, Y.-N., Lee, H.-J., Lü, J., Kobayashi, Y., 2008. New pterosaur tracks from the Hasandong Formation (Lower Cretaceous) of Hadong County, South Korea. Cretaceous Research 29, 345–353. doi:10.1016/j.cretres.2007.05.004

Leffingwell, H.A., 1970. Palynology of the Lance (Late Cretaceous) and Fort Union (Paleocene) Formations of the type Lance area, Wyoming. Geological Society of America Special Paper 127, 1–64.

Leisman, G.A., Gillespie, W.H., Mapes, G., 1988. Plant megafossils from the Hartford Limestone (Virgilian-Upper Pennsylvanian) near Hamilton, Kansas., Regional geology and paleontology of upper Palezoic Hamilton quarry area in southeastern Kansas. (eds. G. Mapes and R. H. Mapes) Guidebook 6, Kansas Geological Survey, Lawrence, KS.

Lejal-Nicol, A., 1986. Decouverte d’une flore a Callipteris dans la region de Suez (Egypte). Actes du 111th Congres National des Societes Savantes, Poitiers, Section des Sciences 2, 9–22.

Lejal-Nicol, A., 1975. Sur la paleoflore post-carbonifere de la bordure est du Bassin de Mourzouk (Libye). Geologische Rundschau 64, 159–174.

Lele, K.M., Streel, M., 1969. Middle Devonian (Givetian) plant microfossils from Goe (Belgium). Extruit des Annales de la Societe Geologique de Belgique 92, 89–121.

Lemoigne, Y., 1984. Donnees nouvelles sur la paleoflore de Colombie. Géobios 17, 667–690.

Leonardi, G., 1994. , Annotated Atlas of South America Tetrapod Footprints (Devonian to Holocene) with an Appendix on Mexico and Central America. República Federativa do Brasil, Ministério de Minas e Energia, Secretaria de Minas e Metalurgia, Companhia de Pesquisa de Recursos Minerais, Brasília.

Leonardi, P., 1967. Strati di La Valle (Ladinico superiore), In: Le Dolomiti Geologia dei Monti Tra Isarco e Piave. P. Leonardi (ed.).

LePage, B.A., Beauchamp, B., Pfefferkorn, H.W., Utting, J., 2003. Late Early Permian plant fossils from the Canadian High Arctic: a rare paleoenvironmental/climatic window in northwest Pangea. Palaeogeography, Palaeoclimatology, Palaeoecology 191, 345–372.

le Roux, S.F., Anderson, H.M., 1977. A review of the localities and flora of the Lower Permian Karroo strata at Vereeniging, South Africa. Palaeontologia Africana 20, 27–42.

Lessuisse, A., Fairon-Demaret, M., 1980. Le gisement a plantes de Niaster (Aywaille, Belgique): repere biostratigraphique noveau aux abords de la limite Covinien-Givetien. Annales de la Societe Geologique de Belgique 103, 157–181.

Liaoning Stratigraphic Group, 1978. The Stratigraphic Tables of Liaoning, Geology Press.

Li, C.S., Hueber, F.M., Hotton, C.L., 2000. A neotype for Drepanophycus spinaeformis Goppert 1852. Canadian Journal of Botany 78, 889–902.

Li, J.H., Ding, B.L., 1981. Two new lamellibranch genera from Lower Triassic of Anhui. Acta Palaeontologica Sinica 20, 325–330.

Li, J., Lockley, M.G., Zhang, Y., Hu, S., Matsukawa, M., Bai, Z., 2012. An important ornithischian tracksite in the Early Jurassic of the Shenmu region, Shaanxi, China. Acta Geologica Sinica 86, 1–10.

Linck, O., 1968. Die marinen Muschelfauna des Schilfsandsteins von Eberstadt, Wuerttemberg, (Trias, Karn, mittl. Keuper 2) und deren Bedeutung. Jahreshefte des Vereins fuer Vaterlaendsiche Naturkunde in Wuerttemberg 123, 69–133.

Lindgren, J., Currie, P.J., Rees, J., Siverson, M., Lindström, S., Alwmark, C., 2008. Theropod dinosaur teeth from the lowermost Cretaceous Rabekke Formation on Bornholm, Denmark. Geobios 41, 253–262. doi:10.1016/j.geobios.2007.05.001

Lindgren, J., Rees, J., Siverson, M., Cuny, G., 2004. The first Mesozoic mammal from Scandinavia. GFF 126, 325–330.

Little, S.A., Stockey, R.A., Rothwell, G.W., 2006a. Solenostelopteris skogiae sp. nov. from the Lower Cretaceous of Vancouver Island [Canada]. Journal of Plant Research 119, 525–532.

Little, S.A., Stockey, R.A., Rothwell, G.W., 2006b. Stramineopteris aureopilosus gen. et sp. nov.: reevaluating the role of vegetative anatomy in the resolution of leptosporangiate fern phylogeny. International Journal of Plant Sciences 167, 683–694. doi:10.1086/501156

Li, W., Batten, D.J., Zhang, D., Zhang, L., 1987. Early Cretaceous megaspores from the Jalainor Group of Northeast Inner Mongolia, P. R. China. Palaeontographica Abteilung B 203, 117–135.

Li, X.-X., Cai, C.-Y., 1977. Early Devonian Zosterophyllum remains from southwest China. Acta Palaeontologica Sinica 16, 12–34.

Li, X.X., Rigby, J.F., 1995. Further contributions to the study of the Qubu Flora from southern Xizang (Tibet). The Palaeobotanist 44, 38–47.

Li, X.X., Shen, G.L., 1996. A brief review of the Permian macrofloras in southeast Asia and their phytogeographical delimitation. Journal of Southeast Asian Earth Sciences 13, 161–170.

Li, X.X., Yao, Z.Q., Deng, L.H., 1982a. An Early Late Permian flora from Toba, Qamdo District, eastern Xizang, Paleontology of Tibet.

Li, X.X., Yao, Z.Q., Zhu, J.N., Duan, S.Y., Hu, Y.F., 1982b. Late Permian plants from northern Xizang, Paleontology of Tibet.

Llorens, M., 2008a. Estudio palinológico de la Formación Punta del Barco, Cretácico Inferior (Aptiano) de la provincia de Santa Cruz, Argentina. Descripciones sistemáticas I: esporas monoletes y triletes lisas y cinguladas. Ameghiniana 45, 153–166.

Llorens, M., 2008b. Estudio palinológico de la Formación Punta del Barco, Cretácico Inferior (Aptiano) de la provincia de Santa Cruz, Argentina: Descripciones sistemáticas II: esporas triletes apiculadas, murornadas, tricrasadas y perinadas. Implicancias paleoecológicas. Ameghiniana 45, 273–288.

Loladze, E.M., 1979. New data on the Late Jurassic and Early Cretaceous floras of Abkhazia and Dzirul’ massif. Bulletin of the Academy of Sciences of the Georgian SSR 94, 385–388.

Long, A.G., 1989. On the occurrence of Clepsydropsis parvula Galtier in tournaisian rocks (Cementstone Group) of Berwickshire, in: Transactions of the Botanical Society of Edinburgh. Taylor & Francis, pp. 397–399.

Long, A.G., 1968. Some specimens of Cladoxylon from the Calciferous Sandstone Series of Berwickshire. Transactions of the Royal Society Edinburgh B 68, 45–60.

Long, A.G., 1964a. A petrified Lower Carboniferous Lepidodendron showing rooting organs identified with Calamopsis (Solms-Laubach). Transactions of the Royal Society Edinburgh B 66, 35–48.

Long, A.G., 1964b. On the structure of some petioles associated with Rhetinangium (Gordon). Transactions of the Royal Society of Edinburgh 66, 1–7.

Lorch, J., 1967. A Jurassic flora of Makhtesh Ramon, Israel. Israel Journal of Botany 16, 131–156.

Lucas, S.G., Heckert, A.B., Huber, P., 1998. Aetosaurus (Archosauromorpha) from the Upper Triassic of the Newark Supergroup, eastern United States, and its biochronological significance. Palaeontology 41, 1215–1230.

Luchnikov, V.S., 1987. About subdivisions of the Lower Jurassic of Tadzhikistan based on paleobotanic data. Sovetskaya Geologiya 66–75.

Luchnikov, V.S., 1982. Stratigraphy of the Jurassic coal deposits of Central Tadzhikistan. Sovetskaya Geologiya 75–85.

Luchnikov, V.S., 1973. Stratigraphy of the Jurassic deposits of the Darvaz Ridge. Sovetskaya Geologiya 38–49.

Lundblad, A.B., 1950. Studies in the Rhaeto-Liassic Floras of Sweden I (Pteridophyta, Pteridospermae, and Cycadophyta from the mining district of NW Scania). Kungl. Svenska Vetenskapsakademiens Handlingar 1, 1–82.

Lyell, C., Dawson, J.W., 1853. On the Remains of a Reptile (Dendrerpeton acadianum, Wyman and Owen) and of a Land Shell discovered in the Interior of an Erect Fossil Tree in the Coal Measures of Nova Scotia. Quarterly Journal of the Geological Society of London 9, 58–63. doi:10.1144/GSL.JGS.1853.009.01-02.19

MacClary, J.S., 1938. Dinosaur trails of Purgatory. Scientific American 158, 72.

MacGinitie, H.D., 1969. The Eocene Green River Flora of northwestern Colorado and northeastern Utah. University of California Publications in Geological Sciences 83, 1–140.

MacGinitie, H.D., 1962. The Kilgore Flora: A Late Miocene Flora from Northern Nebraska. University of California Publications in Geological Sciences 35, 67–158.

MacGinitie, H.D., 1953. Fossil Plants of the Florissant Beds of Colorado. Carnegie Inst. of Washington, Contributions to Paleontology Publ. 599, 1–198.

MacGinitie, H.D., 1941. A Middle Eocene Flora from the Central Sierra Nevada. Carnegie Institution of Washington Publication 534, 1–178.

MacLeod, S.E., Hills, L.V., 1990. Conformable Late Jurassic (Oxfordian) to Early Cretaceous Strata, northern Bowser Basin, British Columbia: a sedimentological and paleontological model. Canadian Journal of Earth Sciences 27, 989–998.

Macphail, M., Cantrill, D.J., 2006. Age and implications of the Forest Bed, Falkland Islands, southwest Atlantic Ocean: Evidence from fossil pollen and spores. Palaeogeography, Palaeoclimatology, Palaeoecology 602–629. doi:10.1016/j.palaeo.2006.03.010

Macphail, M.K., Hill, R.S., Forsyth, S.M., Wells, P.M., 1991. A Late Oligocene‒ Early Miocene cool climate flora in Tasmania. Alcheringa 15, 87–106. doi:10.1080/03115519108619011

Maheshwari, H.K., 1991. Provincialism in Gondwana floras. The Palaeobotanist. Four decades of Indian Palaeobotany. 40, 101–127.

Maheshwari, H.K., Singh, N.P., 1974. On some plant fossils from the Pariwar Formation, Jaisalmer Basin, Rajasthan. The Palaeobotanist 23, 116–123.

Mahmoud, M.S., Moawad, A.-R.M., 2000. Jurassic-Cretaceous (Bathonian to Cenomanian) palynology and stratigraphy of the West Tiba-1 borehole, northern Western Desert, Egypt. Journal of African Earth Sciences 30, 401–416. doi:10.1016/S0899-5362(00)00026-9

Mai, D.H., 2001. Die mittelmiozaenen und obermiozaenen Floren aus der Meuroer und Raunoer Folge in der Lausitz. III. Fundstellen und Palaeobiologie. Palaeontographica Abteilung B 258, 1–85.

Mai, D.H., 1998. Contribution to the flora of the middle Oligocene Calau Beds in Brandenberg, Germany. Review of Palaeobotany and Palynology 101, 43–70.

Mai, D.H., 1997. Die oberoligozanen Floren am Nordrand der Sachsischen Lausitz. Palaeontographica Abteilung B 244, 1–124.

Mai, D.H., Walther, H., 1988. Die pliozaenen Floren von Thueringen, Deutsche Demokratische Republik. Quartaerpalaeontologie 7, 55–297.

Mai, D.H., Walther, H., 1978. Die Floren des Haselbacher Serie im Weisselster-Becken (Bezirk Leipzig, DDR). Abhandlungen des Staatlichen Museums fuer Mineralogie und Geologie zu Dresden 28, 1–200.

Mamay, S.H., 1976. Paleozoic origin of the cycads. U.S. Geological Survey Professional Paper 934.

Mamay, S.H., 1962. Occurrence of Pseudobornia Nathorst in Alaska. The Palaeobotanist 11, 19–22.

Mamay, S.H., 1960. Padgettia, a new genus based on fertile neuropteroid foliage from the Permian of Texas. Palaeobotanist 9, 53–57.

Mamay, S.H., 1954. A Permian Discinites cone. Journal of Washington Academy of Science 44, 7–11.

Mamay, S.H., Mapes, G., 1992. Early Virgilian plant megafossils from the Kinney Brick Company Quarry, Manzanita Mountains, New Mexico. New Mexico Bureau of Mines & Mineral Resources Bulletin 138, 61–85.

Manchester, S.R., Akhmetiev, M.A., Kodrul, T.M., 2002. Leaves and fruits of Celtis aspera (newberry) comb. nov. (Celtidaceae) from the Paleocene of North America and eastern Asia. International Journal of Plant Sciences 163, 725–736.

Mangerud, G., 1994. Palynostratigraphy of the Permian and lowermost Triassic succession, Finnmark Platform, Barents Sea. Review of Palaeobotany and Palynology 82, 317–349.

Manum, S.B., Bose, M.N., Os Vigran, J., 1991. The Jurassic Flora of Andoya, northern Norway. Review of Palaeobotany and Palynology 68, 233–256.

Mapes, G., Rothwell, G.W., 1988. Diversity among Hamilton conifers (In G. Mapes and R. H. Mapes (eds.), Regional Geology and Paleontology o fUpper Paleozoic Hamilton Quarry Area in Southeastern Kansas. Guidebook, 22nd Annual Meeting, South-Central Section, Geological Society of America.

Marinov, N.A., Zonenshain, L.P., Blagonravov, V.A., 1973. Geology of the People’s Republic of Mongolia: Stratigraphy.

Markovich, E.M., 1971a. Jurassic flora and vegetation of the Or’-Ilek province.

Markovich, E.M., 1971b. The Middle Jurassic flora of Southern Belorussia. Doklady Akademii Nauk SSSR 200, 419–421.

Markovich, E.M., 1970. Correlation of sections of the Nazarovo and Rybinsk regions, Methods of correlation of continental coal unit sections using as an example some Mesozoic basins of Siberia.

Markovich, E.M., 1968. Comparison of sections of the Middle Jurassic deposits of Borodinskii and Pereyaslavskoye coal deposits. Sovetskaya Geologiya 108–111.

Markovich, E.M., Ignat’ev, G.A., Pereyaslavskii, I.V., 1984. On the age of coal-bearing unit of Kadara (Northern TransBaykal). Sovetskaya Geologiya 52–59.

Markovich, E.M., Okuneva, T.M., Trussova, E.K., 1988. Ammonites, bivalves, conchostracans, and plants of Transbaikalia and Yakutiya. Newsletters in Stratigraphy. 1.Soviet Union 19, 79–94.

Markovich, E.M., Prosviryakova, Z.P., 1972. About the role of paleobotanic analysis for studies of tectonically disturbed coal-bearing areas (Chervonoozerskaya area of the Pripyat’ basin as an example). Sovetskaya Geologiya 127–130.

Marshall, J.E.A., Allen, K.C., 1982. Devonian miospore assemblages from Fair Isle, Shetland. Palaeontology 25, 277–312.

Martinez Garcia, E., Wagner, R.H., Garcia-Ramos, J.C., 1991. La cobertera paleozoica posthercinica en el Oriente de Asturias. Geogaceta 10, 160–162.

Martínez, L.C., Olivo, M.S., 2015. Tempskya in the Valanginian of South America (Mulichinco Formation, Neuquén Basin, Argentina)—Systematics, palaeoclimatology and palaeoecology. Review of Palaeobotany and Palynology 219, 116–131.

Massari, F., Neri, C., Pittau, P., Fontana, D., Stefani, C., 1994. Sedimentology, palynostratigraphy and sequence stratigraphy of a continental to shallow-marine rift-related succession: Upper Permian of the eastern Southern Alps (Italy). Mem. Sci. Geol. 46, 119–234.

Matskevich, M.M., 1972. Stratigraphy of the USSR. Jurassic System.

Matsumoto, M., Nishida, H., 2003. Osmunda shimokawaensis sp. nov. and Osmunda cinnamomea L. based on permineralized rhizomes from the Middle Miocene of Shimokawa, Hokkaido, Japan. Paleontological research 7, 153–165.

Matsuoka, H., Kusuhashi, N., Takada, T., Setogushi, T., 2002. A clue to the Neocomian vertebrate fauna: initial results from the Kuwajima “Kaseki-kabe” (Tetori Group) in Shiramine, Ishikawa, central Japan. Memoirs of the Faculty of Science, Kyoto University, Series of Geology and Mineralogy 59, 33–45.

Matten, L.C., 1974. The Givetian flora from Cairo, New York: Rhacophyton, Triloboxylon, and Cladoxylon. Botanical Journal of the Linnean Society 68, 303–318.

Mautino, L.R., Anzótegui, L.M., 2000. Esporas del Mioceno y retrabajadas del Mesozoico en la Formación Chiquimil, Vallecito, provincia de Catamarca. Ameghiniana 37, 13–22.

Mautino, L.R., Anzótegui, L.M., 1998. Palinología de la Formación Chiquimil (Mioceno superior) en Vallecito, provincia de Catamarca, Argentina. Parte I. Esporas: Especies nuevas. Ameghiniana 35, 227–233.

McComas, M.A., 1988. Upper Pennsylvanian compression floras of the 7-11 Mine, Columbiana County, northeastern Ohio. Ohio Journal of Science 88, 48–52.

McGregor, D.C., 1960. Devonian spores from Melville Island Canadian Arctic archipelago. Palaeontology 3, 26–44.

McIver, E.E., 2002. The paleoenvironment of Tyrannosaurus rex from southwestern Saskatchewan, Canada. Canadian Journal of Earth Sciences 39, 207–221.

McIver, E.E., Basinger, J.F., 1993. Flora of the Ravenscrag Formation (Paleocene), Southwestern Saskatchewan, Canada. Palaeontographica Canadiana 10, 1–85.

McLoughlin, S., Drinnan, A.N., 1995. A Middle Jurassic Flora from the Walloon Coal Measures, Mutdapilly, Queensland, Australia. Memoirs of the Queensland Museam 38, 257–272.

McLoughlin, S., Drinnan, A.N., Rozefelds, A.C., 1995. A Cenomanian Flora from the Winton Formation, Eromanga Basin, Queensland, Australia. Memoirs of the Queensland Museam 38, 273–313.

McLoughlin, S., Tosolini, A.-M.P., Nagalingum, N.S., Drinnan, A.N., 2002. Early Cretaceous (Neocomian) flora and fauna of the Lower Strzelecki Group, Gippsland Basin, Victoria. Memoirs of the Association of Australasian Palaeontologists 26, 1–144.

Medeiros, M.A., Schultz, C.L., 2002. A fauna dinossauriana da “Laje do Coringa”, Cretáceo Médio do nordeste do Brasil [The dinosaurian fauna of “Laje do Coringa”, middle Cretaceous of northeastern Brazil]. Arquivos do Museu Nacional, Rio de Janeiro 60, 155–162.

Mejia-Velazquez, P.J., Dilcher, D.L., Jaramillo, C.A., Fortini, L.B., Manchester, S.R., 2012. Palynological composition of a Lower Cretaceous South American tropical sequence: Climatic implications and diversity comparisons with other latitudes. American Journal of Botany 99, 1819–1827. doi:10.3732/ajb.1200135

Mejia-Velazquez, P., Manchester, S.R., Jaramillo, C., 2012. Floristic reconstruction of two Lower Cretaceous sections from Peru during the period of early angiosperm diversification and their climatic implications. 13th International Palynological Congress and 9th International Organization of Palaeobotany Conference GS02–GS006.

Melendi, D.L., Scafati, L.H., Volkheime, W., 2003. Palynostratigraphy of the Paleogene Huitrera Formation in N-W Patagonia, Argeyesina. Neues Jahrbuch für Geologie und. Paläoyeslogie, Abhandlungen 228, 205–273.

Mellon, G.B., 1967. Stratigraphy and Petrology of the Lower Cretaceous Blairmore and Mannville Groups, Alberta Foothills and Plains. Research Council of Alberta 21, 1–270.

Mellon, G.B., Hall, J.H., Stelck, C.R., 1963. Lower Cretaceous Section, Belcourt Ridge, Northeastern British Columbia. Bulletin of Canadian Petroleum Geology 11, 64–72.

Menendez, C.A., 1961. Estípite petrificado de una nueva Cyatheaceae del Terciario de Neuquén. Boletín de la Sociedad Argentina de Botánica IX, 331–358.

Menéndez, C.A., 1959. Flora cretacica de la serie del Castillo al S del Cerro Cachetaman, Chubut. Revista de la Asociación Geológica Argentina XIV, 219–238.

Menendez, C.A., Azcuy, C.L., 1971. Microflora carbónica de la localidad de Paganzo, Provincia de La Rioja. Parte II. Ameghiniana VIII, 25–36.

Menendez, C.A., Azcuy, C.L., 1969. Microflora carbónica de la ciudad de Paganzo, Provincia de La Rioja. Parte I. Ameghiniana VI, 77–97.

Meyen, S.V., 1997. Permian conifers of Western Angaraland. Review of Palaeobotany and Palynology 96, 351–447.

Meyer-Berthaud, B., Rowe, N.P., 1997. A Lower Carboniferous plant assemblage from Thuringia (Germany): compressions. Review of Palaeobotany and Palynology 97, 361–379.

Meyer, H.W., Manchester, S.R., 1997. The Oligocene Bridge Creek flora of the John Day Formation, Oregon. University of California Publications in Geological Sciences 141, 1–195.

Michels, D., 1986. Oekologie und Fazies des juengsten Ober-Devon von Velbert (Rheinisches Schiefergebirge). Goettinger Arbeiten zur Geologie und Palaeontologie 29, 1–86.

Mildenhall, D.C., Kennedy, E.M., Lee, D.E., Kaulfuss, U., Bannister, J.M., 2014. Palynology of the early Miocene Foulden Maar, Otago, New Zealand: Diversity following destruction. Review of Palaeobotany and Palynology 27–42. doi:10.1016/j.revpalbo.2014.02.003

Miner, E.L., 1935a. Paleobotanical Examinations of Cretaceous and Tertiary Coals: I. Cretaceous Coals from Greenland. The American Midland Naturalist 16, 585–615.

Miner, E.L., 1935b. Paleobotanical Examinations of Cretaceous and Tertiary Coals: II. Cretaceous and Tertiary Coals from Montana. The American Midland Naturalist 16, 616–625.

Miner, E.L., 1932. Megaspores ascribed to Selaginellites, from the Upper Cretaceous Coals of Western Greenland. Journal of the Washington Academy of Sciences 22, 497–506.

Mogucheva, N.K., 1984. Stratigrafiya fauna i flora Triasa Sibiri.

Mogucheva, N.K., Batyaeva, S.K., 1987. New data on the Triassic deposits of Gorny Altai, Trudy Instituta Geologii i Geofiziki (Novosibirsk). Boreal Triassic.

Molnar, R.E., Wiffen, J., Hayes, B., 1998. A probable theropod bone from the latest Jurassic of New Zealand. New Zealand Journal of Geology and Geophysics 41, 145–148.

Morel, E.M., 1994. El Triasico del Cerro Cacheuta, Mendoza (Argentina). Parte 1: Geologia, contenido paleofloristico y cronoestratigrafia. Ameghiniana 31, 161–176.

Mørk, A., Elvebakk, G., Forsberg, A.W., Hounslow, M.W., Nakrem, H.A., Vigran, J.O., Weitschat, W., 1999. The type section of the Vikinghøgda Formation: a new Lower Triassic unit in central and eastern Svalbard. Polar Research 18, 51–82.

Mu, E. -z., Boucot, A.J., Chu, X., -y. Rong, J., 1986. Correlation of the Silurian rocks of China. Geological Society of America Special Paper 202, 1–80.

Murphy, J.C., Clites, E.C., Demko, T.M., Bodenbender, B.E., 2005. Fluvial sedimentology and plant taphonomy of a dinosaur site and associated strata, upper Jurassic Morrison and lower Cretaceous Cloverly Formations, Bighorn Basin, WY. Geological Society of America Abstracts with Programs 37, 32.

Murphy, N.L., Trexler, D., Thompson, M., 2007. “Leonardo,” a mummified Brachylophosaurus (Ornithischia: Hadrosauridae) from the Judith River Formation of Montana, Horns and Beaks: Ceratopsian and Ornithopod Dinosaurs.

Murray, N., 1939. The Microflora of the Upper and Lower Estuarine Series of the East Midlands. Geological Magazine 905, 478–489.

Mustoe, G.E., 2002. Eocene Bird, Reptile, and Mammal Tracks from the Chuckanut Formation, Northwest Washington. Palaios 17, 403–413.

Nakazawa, K., 1973. On the Permian fossils from Jengka Pass, Pahang, Malay Peninsula. Science Reports of the Tohoku University. Second Series, Geology. Special Volume (Hatai Memorial Volume) 6, 277–296.

Narváez, P.L., Mego, N., Prámparo, M.B., 2012. Cretaceous cicatricose spores from north and central-western Argentina. Taxonomic and biostratigraphic discussion. Palynology 37, 1–15. doi:10.1080/01916122.2012.762062

Narváez, P.L., Sabino, I.F., 2008. Palynology of the Las Curtiembres Formation (Late Cretaceous, Salta Group Basin), Las Conchas Creek area, northwestern Argentina. Ameghiniana 45, 473–482.

Nasu, T., Seto, K., 1976. Fossil macrospores and massulae of Salvinia natans from the Pliocene and the Quaternary sediments in the Kinki and Tokai districts, Japan. Bulletin of the Osaka Museum of Natural History 30, 37–48.

Naugolnykh, S.V., 1998. Kungurian flora of the Middle Cis-Urals. GEOS.

Naugolnykh, S.V., 1996. Permian floral assemblages of Kozhim section (Pechora Fore-Ural), their age and taxonomic composition. GEOS. Abstracts and proceedings, Vakhrameev memorial conference 49–54.

Nei Monggol Stratigraphic Group, 1978. Regional stratigraphic table of northern China, Nei Monggol part, Geological Publishing House, Beijing, China.

Nel, A., Neraudeau, D., Perrichot, V., Girard, V., Gomez, B., 2008. A new dragonfly family from the Upper Cretaceous of France. Acta Palaeontologica Polonica 53, 165–168.

Neraudeau, D., Perrichot, V., -P. Colin, J., Girard, V., Gomez, B., Guillocheau, F., Masure, E., Peyrot, D., Tostain, F., Videt, B., Vullo, R., 2008. A new amber deposit from the Cretaceous (uppermost Albian-lowermost Cenomanian) of southwestern France. Cretaceous Research 29, 925–929. doi:10.1016/j.cretres.2008.05.009

Nevo, A., 1964. Fossil urodeles in Early Lower Cretaceous deposits of Makhtesh Ramon, Israel. Nature 201, 415–416.

Nevo, A., 1956. Fossil frogs from a Lower Cretaceous bed in southern Israel (Central Negev). Nature 178, 1191–1192.

Nikiforova, O.I., 1977. Podolia, in: Martinsson, A. (Ed.), The Silurian-Devonian Boundary (IUGS Publication, Series A, Number 5). pp. 52–64.

Niklas, K.J., Phillips, T.L., Carozzi, A.V., 1976. Morphology and paleoecology of Protosalvinia from the Upper Devonian (Famennian) of the Middle Amazon Basin of Brazil. Palaeontographica Abteilung B 155, 1–30.

Nikol’skii, V.M., Prosviryakova, Z.P., 1980. Age of the coal deposits of the lower part of the Ili River brown-coal locality. Sovetskaya Geologiya 66–70.

Ningxia Stratigraphic Group, 1980. The Stratigraphic Tables of Ningxia, Geology Press.

Norvick, M.S., Burger, D., 1975. Palynology of the Cenomanian of Bathurst Island, Northern Territory, Australia. Department of Minerals and Energy, Bureau of Mineral Resources, Geology and Geophysics 1–169.

Novojilov, N.I., 1958. Mérostomates du dévonien inférieur et moyen de Sibérie. Annales de la Société Géologique du Nord 78, 243–257.

Nwojiji, C.N., Osterloff, P., Okoro, A., Ukeri, P.O., 2013. Palynostratigraphy and Age of the Sequence Penetrated by the Kolmani River 1 Well in the Gongola Basin, Northern Benue Trough, Nigeria. Journal of Geosciences and Geomatics 1, 15–21. doi:10.12691/jgg-1-1-3

Nye, E., Feist-Burkhardt, S., Horne, D.J., Ross, A.J., Whittaker, J.E., 2008. The palaeoenvironment associated with a partial Iguanodon skeleton from the Upper Weald Clay (Barremian, Early Cretaceous) at Smokejacks Brickworks (Ockley, Surrey, UK), based on palynomorphs and ostracods. Cretaceous Research 29, 417–444. doi:10.1016/j.cretres.2008.01.004

Obhrel, J., 1961. Die Flora der Srbsko-Schichten (Givet) des mittelbohmischen Devons. Sbornik U. U. G. 26, 7–46.

Obrhel, J., 1962. The flora of the Pridoli-beds (Budnany-Stufe) of the Mid-Bohemian Silurian. [Die Flora der Pridoli-Schichten (Budnany-Stufe) des mittelboehmischen Silurs]. Geologie 11, 83–97.

Obrhel, J., 1961. Die Flora der Srbsko-Schichten (Givet) des mittelboehmischen Devons. [The Flora of the Srbsko Strata (Givetian) of the Central Bohemian Devonian]. Sbornik Ustredniho Ustavu Geologickeho 26, 7–46.

Odreman-Rivas, O., Wagner, R.H., 1979. Precisiones sobre algunas floras Carboniferas Permicas de los Andes Venezolanos. Boletin de Geologia 13, 77–79.

Oliver, C.G., Menendez, C.A., 1968. Geologia de la Quebrada Juan de Morales, Tarapaca, Chile y su flora Jurasica. Terceras Jornadas Geologicas Argentinas 163–171.

Olsen, P.E., Huber, P., 1997. Stop 3: Triangle Brick Quarry, TRIBI: Triassic Basin Initiative, Abstracts with Programs and Field Trip Guidebook, Duke University, Durham.

Olsen, P.E., Remington, C.L., Cornet, B., Thomson, K.S., 1978. Cyclic change in Late Triassic lacustrine communities. Science 201, 729–733.

Olson, E.C., Barghusen, H., 1962. Permian vertebrates from Oklahoma and Texas. Part I.—Vertebrates from the Flowerpot Formation, Permian of Oklahoma. Oklahoma Geological Survey, Circular 59, 5–48.

Omran, A.M., Soliman, H.A., Mahmoud, M.S., 1990. Early Cretaceous palynology of three boreholes from northern Western Desert (Egypt). Review of Palaeobotany and Palynology 66, 293–312.

Oosthuizen, R.D.F., 1984. Preliminary catalogue and report on the biostratigraphy and palaeogeographic distribution of the Bokkeveld fauna. Transactions of the Geological Society of South Africa 87, 125–140.

OPLUŠTIL, S., Cleal, C.J., 2007. A comparative analysis of some Late Carboniferous basins of Variscan Europe. Geological Magazine 144, 417–448.

Orlovskaya, E.P., 1977. On the Early Mesozoic flora of the Ilek River basin. Materials on the History of Fauna and Flora of Kazakhstan. Mesocenozoic Fauna and Flora of North-Western Kazakhstan. 7, 105–125.

Orlovskaya, E.P., 1958. Materials on Jurassic flora of the Maykuben’ coalfield. Materials on the history of fauna and flora of Kazakhstan 2, 117–142.

Orlovskaya, E.R., 1974. Jurassic flora of the Ili coal basin. Akademia Nauk SSSR, Kazakhstan 6, 93–104.

Osborn, H.F., 1912. Forty-Third Annual Report of the American Museum of Natural History for the Year 1911. Department of Vertebrate Palaeontology 102–104.

Ozaki, K., 1991. Late Miocene and Pliocene Floras in Central Honshu, Japan, Bulletin of Kanagawa Prefectural Museam Natural Science Special Issue.

Palamarczuk, S., Barreda, V., 2000. Palinología del Paleogeno tardío-Neógeno temprano, pozo Aries x-1, plataforma Continental Argentina, Tierra del Fuego. Ameghiniana 37, 221–234.

Palazzesi, L., Barreda, V., 2004. Primer registro palinológico de la Formación Puerto Madryn, Mioceno de la provincia de Chubut, Argentina. Ameghiniana 41, 355–362.

Palma-Heldt, S.C., Fernandoy, F.J., Quezada, I., Leppe, M.A., 2004. Registro palinológico de Cabo Shirreff, Isla Livingstone, nueva localidad para el Mesozoico de las Shetland del Sur. V Simposio Argentino y I Latinoamericano sobre Investigaciones Antárticas.

Pantic, N.K., 1981. Macroflora and palynomorphs from Lower Jurassic of Budos Mountain, Montenegro. Annales Geologiques de la Peninsule Balkanique 45, 157–171.

Papú, O.H., 2002. Nueva microflora de edad maastichtiana en la localidad de Calmu-Co, sur de Mendoza, Argentina. Ameghiniana 39, 415–426.

Papú, O.H., 1993. Nuevos Hallasgos del Género Grapnelispora (Stover & Partridge) en la Formación Loncoche de Ranquil-Co, Cretácico Superior en la Provincia de Mendoza, Argentina. Ameghiniana 30, 143–147.

Papú, O.H., 1989. Estudio Palinológico de la Formación Paso del Sapo (Cretácico Superior), Valle Medio del Río Chubut. Granos de Polen, Consideraciones Estadísticas, Paleoecológicas y Paleoambientales. Ameghiniana 25, 193–202.

Parker, J.M., 1966. Triassic reptilian fossil from Wake Count, North Carolina. The Journal of the Elisha Mitchell Scientific Society 82, 92.

Passalia, M.G., 2007a. A mid-Cretaceous flora from the Kachaike Formation, Patagonia, Argentina. Cretaceous Research 28, 830–840. doi:10.1016/j.cretres.2006.12.006

Passalia, M.G., 2007b. Nuevos registros para la flora cretácica descripta por Halle (1913) en lago San Martín, Santa Cruz, Argentina 44, 565–595.

Passalia, M.G., Del Fueyo, G., Archangelsky, S., 2010. An Early Cretaceous zamiaceous cycad of South West Gondwana: Restrepophyllum nov. gen. from Patagonia, Argentina. Review of Palaeobotany and Palynology 161, 137–150. doi:10.1016/j.revpalbo.2010.04.001

Passoni, L., van Konijnenburg-van Cittert, J.H.A., 2003. New taxa of fossil Carnian plants from Mount Pora (Bergamasc Alps, Northern Italy). Review of Palaeobotany and Palynology 123, 321–346.

Patra, B.P., 1971. Notes on some Upper Gondwana plants from the Athgarh Sandstones, Cuttack District, Orissa. The Palaeobotanist 20, 325–333.

Patton, W.J.H., 1958. Mississippian succession in South Nahanni River area, Northwest Territories, Jurassic and Carboniferous of Western Canada.

Pendleton, J.L., Cleal, C.J., Falcon-Lang, H.J., Wagner, R.H., Wellman, C.H., 2012. Palaeobotany of the Pennsylvanian (mid-Bolsovian–Cantabrian; Moscovian) Warwickshire Group of the Bristol Coalfield, UK: Biostratigraphy and palaeoecology. Review of Palaeobotany and Palynology 179, 17–43.

Peng, N., Liu, Y., Kuang, H., Jiang, X., Xu, H., 2012. Stratigraphy and geochronology of vertebrate fossil-bearing Jurassic strata from Linglongta, Jianchang county, western Liaoning, northeastern China. Acta Geologica Sinica 86, 1326–1339.

Peppe, D.J., Erickson, J.M., Hickey, L.J., 2007. Fossil Leaf Species from the Fox Hills Formation (Upper Creataceous: North Dakota, USA) and their Paleographic Significance. Journal of Paleontology 81, 550–567.

Peppe, D.J., Hickey, L.J., Miller, I.M., Green, W.A., 2008. A morphotype catalogue, floristic analysis and stratigraphic description of the Aspen Shale flora Cretaceous–Albian) of Southwestern Wyoming. Bulletin of the Peabody Museum of Natural History 49, 181–208.

Peppers, R.A., 1997. Palynology of the Lost Branch Formation of Kansas--new insights on the major floral transition at the Middle-Upper Pennsylvanian boundary. Review of Palaeobotany and Palynology 98, 223–246.

Peppers, R.A., 1996. Palynological Correlation of major Pennsylvanian (Middle and Upper Carboniferous) chronostratigraphic boundaries in the Illinois and other coal basins. Geological Society of America Memoir 188, 1–111.

Peppers, R.A., 1970. Correlation and Palynology of Coals in the Carbondale and Spoon Formations (Pennsylvanian) of the Northeastern Part of the Illinois Basin. Illinois State Geological Survey Bulletin 93, 1–174.

Perez Loinaze, V., Archangelsky, S., Cladera, G., 2011. Palynostratigraphic study of the Early Cretaceous Río Mayer and Kachaike formations at the Quebrada El Moro Section, Austral Basin, southwestern Argentina. Cretaceous Research 161–171. doi:10.1016/j.cretres.2011.10.015

Person, C.P., Delevoryas, T., 1982. The Middle Jurassic flora of Oaxaca, Mexico. Palaeontographica Abteilung B 180, 82–119.

Petersen, K.L., Mehringer, P.J., Jr., Gustafson, C.E., 1983. Late-glacial vegetation and climate at the Manis mastodon site, Olympic Peninsula, Washington. Quaternary Research 20, 215–231.

Pettitt, J.M., 1965. Two Heterosporous Plants from the Upper Devonian of North America. Bulletin of the British Museum (Natural History) 10, 83–95.

Peyrot, D., Jolly, D., Barron, E., 2005. Apport de données palynologiques à la reconstruction paléoenvironnementale de l’Albo-Cénomanien des Charentes (Sud-Ouest de la France). Comptes Rendus Palevol 4, 151–165. doi:10.1016/j.crpv.2004.11.016

Pfefferkorn, H.W., 1979. High diversity and stratigraphic age of the Mazon Creek flora (In M. Nitcki (ed.)). Mazon Creek Fossils. University of Chicago Press, Chicago, IL USA 129–142.

Philippe, M., Thevenard, F., Barale, G., Ferry, S., Guignard, G., 1998. Middle Bathonian floras and phytocoenoses of France. Palaeogeography, Palaeoclimatology, Palaeoecology 143, 135–158.

Phillips, T.L., Andrews, H.N., Gensel, P.G., 1972. Two Heterosporous Species of Archaeopteris from the Upper Devonian of West Virginia. Palaeontographica Abteilung B 139, 47–71.

Phillips, T.L., DiMichele, W.A., 1998. A Transect through a Clastic-swamp to Peat-swamp Ecotene in the Springfield Coal, Middle Pennsylvanian Age of Indiana, USA. Palaios 13, 113–128.

Picard, L., Hirsch, F., 1987. The Jurassic stratigraphy in Israel and the adjacent countries, Publications of the Israel Academy of Sciences and Humanities.

Pickett, J.W., Macphail, M.K., Partridgec, A.D., Poled, M.S., 1997. Middle Miocene palaeotopography at Little Bay, near Maroubra, New South Wales. Australian Journal of Earth Sciences: An International Geoscience Journal of the Geological Society of Australia 44, 509–518. doi:10.1080/08120099708728330

Pigg, K.B., Stockey, R.A., 1991. Platanaceous plants from the Paleocene of Alberta, Canada. Review of Palaeobotany and Palynology 70, 125–146.

Pinna, G., 1985. Exceptional preservation in the Jurassic of Osteno. Philosophical Transactions of the Royal Society of London Series B, Biological Sciences 311, 171–180.

Pinto, I.D., 1956. Artopods da Formacao Santa Maria (Triassico Superior) Do Rio Grande do Sul, com noticias sobre alguns restos vegetais. Boletim da Sociedade Brasileira de Geologia 5, 75–87.

Pirrie, D., Riding, J.B., 1988. Sedimentology, Palynology and structure of Humps Island, northern Antartic Peninsula. British Antarctic Survey Bulletin 80, 1–19.

Playford, G., 1976. Plant microfossils from the Upper Devonian and Lower Carboniferous of the Canning Basin, Western Australia. Palaeontographica Abteilung B 158, 1–71.

Plumstead, E.P., 1977. A new phytostratigraphical Devonian zone in southern Africa which includes the first record of Zosterophyllum. Transactions of the Geological Society of South Africa 80, 267–277.

Pole, M., 1997. Paleocene plant macrofossils from Kakahu, south Canterbury, New Zealand. Journal of the Royal Society of New Zealand 27, 371–400.

Pole, M.S., Hill, R.S., Green, N., Macphail, M.K., 1993. The Oligocene Berwick Quarry Flora - Rainforest in a Drying Environment. Australian Systematic Botany 6, 399–427. doi:10.1071/SB9930399

Pole, M., Vajda, V., 2009. A new terrestrial Cretaceous-Paleogene site in New Zealand turnover in macroflora confirmed by palynology. Cretaceous Research 30, 917–938. doi:10.1016/j.cretres.2009.02.007

Pomerantseva, A.A., 1970. Correlation of sections of the Abansk Region, Methods of correlation of continental coal unit sections using as an example some Mesozoic basins of Siberia.

Popa, M.E., 2000. Early Jurassic land flora of the Getic Nappe.

Popov, S.V., Sychevskaya, E.K., Akhmet’ev, M.A., Zaporohets, N.I., Golovina, L.A., 2008. Stratigraphy of the Maikop Group and Pteropoda Beds in Northern Azerbaijan. Stratigraphy and Geological Correlation 16, 664–677.

Popov, Y.N., 1962. Nekotorya rannetriasovye ammonoidei Severnogo Kavkaza. Paleontologicheskii Zhurnal 1962, 40–46.

Potonie, R., Klaus, W., 1954. Einige Sporengattungen des alpinen Salzgebirges. Geologisches Jahrbuch, Hannover 68, 517–546.

Potter, D.R., 1963. An emendation of the sporomorph Arcellites Miner 1935. Oklahoma Geology Notes 23, 227–230.

Povilauskas, L., 2012. Palinología de la Formación Monte Chico (Cretácico Tardío) de la Provincia de Santa Cruz, Argentina: Gimnospermas. Revista Brasileira de Paleontologia 15, 85–94. doi:10.4072/rbp.2012.1.07

Povilauskas, L., 2011. Palinología de la Formación Monte Chico (Cretácico Tardío) de la Provincia de Santa Cruz, Argentina: Esporas. Revista Brasileira de Paleontologia 14, 255–268. doi:10.4072/rbp.2011.3.05

Povilauskas, L., Barreda, V., Marenssi, S., 2008. Polen y esporas de la Formación La Irene (Maastrichtiano), sudoeste de la provincia de Santa Cruz, Argentina: primeros resultados. Geobios 41, 819–831. doi:10.1016/j.geobios.2008.07.002

Povilauskas, L.K., 2010. Palinología del entorno cretácico/paleógeno del SO de la provincia de Santa Cruz: significado estratigráfico y paleoambiental.

Prámparo, M.B., 1994. Lower Cretaceous palynoflora of the La Cantera Formation, San Luis Basin: correlation with other Cretaceous palynofloras of Argentina. Cretaceous Research 15, 193–203.

Prasad, G.V.R., Godinot, M., 1994. Eutherian tarsal bones from the Late Cretaceous of India. Journal of Paleontology 68, 892–902.

Prasad, G.V.R., Sahni, A., 1988. First Cretaceous mammal from India. Nature 332, 638–640.

Preda, I., Culda, V., Badaluta, A., Streanga, V., 1985. La flore liasique de Preghada (Banat). Analele Universitatii Bucuresti, Geologie 34, 71–75.

Prinada, V.D., 1969. Iskopaemaia flora Korvunchianskoi sviti (Flora of the Korvunchanskoi Suite). Mezhdunarodnyi Stratigraficheskii Komitet SSSR, Trudy 3.

Prince, N.K., Lockley, M.G., 1989. The sedimentology of Purgatoire tracksite region, Morrison Formation of southeastern Colorado, Dinosaur Tracks and Traces.

Pross, J., Contreras, L., Bijl, P.K., Greenwood, D.R., Bohaty, S.M., Schouten, S., Bendle, J.A., Röhl, U., Tauxe, L., Raine, J.I., Huck, C.E., van de Flierdt, T., Jamieson, S.S.R., Stickley, C.E., van de Schootbrugge, B., PARSE ERROR, 2012. Persistent near-tropical warmth on the Antarctic continent during the early Eocene epoch. Nature 73–77. doi:10.1038/nature11300

Prosviryakova, Z.P., 1966. Jurassic flora of the Mangyshlak and its significance for stratigraphy.

Prosviryakova, Z.P., 1965. Paleobotanic characteristics of Mesozoic coal deposits of the Crimea-Caucasus-PreCaspian province, Zakonomernosti razvitiya i razmeshcheniya rannemezozoiskogo uglenakopleniya na territorii Kryma, Kavkaza i Prikaspiya. (Regularities in evolution and distribution of Early Mesozoic coal production in the territories of Crimea, Caucasus and PreCaspian).

Pruvost, P., 1919. Introduction À l’etude du terrain houiller du nord et du Pas-de-Calais: la faune continentale du terrain houiller du nord de la France.

PŠENIČKA, J., BEK, J., Zodrow, E.L., Cleal, C.J., Hemsley, A.R., 2003. A new late Westphalian fossil marattialean fern from Nova Scotia. Botanical Journal of the Linnean Society 142, 199–212.

Qinghai Stratigraphic Group, 1980. The Stratigraphic Tables of Qinghai, Geology Press.

Quattrocchio, M., 1978. Contribución al conocimiento de la Palinología Estratigráfica de la Formación Lumbrera (Terciario Inferior, grupo Salta). Ameghiniana XV, 285–300.

Quattrocchio, M.E., Martínez, M.A., Carpinelli Pavisich, A., Volkheimer, W., 2006. Early Cretaceous palynostratigraphy, palynofacies and palaeoenvironments of well sections in northeastern Tierra del Fuego, Argentina. Cretaceous Research 27, 584–602. doi:10.1016/j.cretres.2005.11.012

Quinet, G.-E., 1969a. , Bernissart...il y a 125.000.000 d’Années. Le Règne des Iguanodons [Bernissart...125,000,000 Years Ago. The Reign of the Iguanodon].

Quinet, G.-E., 1969b. , Bernissart...il y a 125.000.000 d’Années. Le Règne des Iguanodons [Bernissart...125,000,000 Years Ago. The Reign of the Iguanodon].

Quinet, G.-E., 1969c. , Bernissart...il y a 125.000.000 d’Années. Le Règne des Iguanodons [Bernissart...125,000,000 Years Ago. The Reign of the Iguanodon].

Rajanikanth, A., Sukh-Dev, 1989. The Kota Formation: fossil flora and stratigraphy. Geophytology 19, 52–64.

Rao, R., Xu, J., Chen, Y., Zou, D., 1987. The Triassic System of Qinghai-Xizang Plateau, Geological Memoirs, People’s Republic of China Ministry of Geology and Mineral Resources.

Rayner, R.J., Coventry, M.K., 1985. A Glossopteris flora from the Permian of South Africa. South African Journal of Science 81, 21–32.

Read, C.B., 1947. Pennsylvanian floral zones and floral provinces. Journal of Geology 55, 271–279.

Read, C.B., 1936a. A Devonian Flora from Kentucky. Journal of Paleontology 10, 215–227.

Read, C.B., 1936b. The Flora of the New Albany Shale. Part 1. Diichnia kentuckiensis, A New Representative of the Calamopityeae. United States Geological Survey Professional Paper 185-H, 149–155.

Read, C.B., 1935. An occurence of the genus Cladoxylon Unger, in North America. Journal of the Washington Academy of Sciences 25, 493–497.

Read, C.B., 1934. A flora of Pottsville age from the Mosquito Range, Colorado. U.S. Geological Survey Prof. Paper 185-D, 79–96.

Read, C.B., Campbell, G., 1939. Preliminary Account of the New Albany Shale Flora. The American Midland Naturalist 21, 435–453.

Read, C.B., Mamay, S.H., 1964. Upper Paleozoic floral zones and floral provinces of the United States. Geological Survey Professional Paper 454, K1–K32.

Rees, P.M., Cleal, C.J., 2004. Lower Jurassic floras from Hope Bay and Botany Bay, Antarctica. Special Papers in Palaeontology 72, 5–90.

Ren, H., Rigby, J., Shuying, D., 1990. REVISION OF GLOSSOPTERIS FLORA FROM SOUTHERN XIZANG. Chinese Journal of Geology 3, 003.

Rex, G.M., Scott, A.C., 1987. The Sedimentology, Palaeoecology and Preservation of the Lower Carboniferous Plant Deposits at Pettycur, Fife, Scotland. Geol. Mag. 124, 43–66.

Rey, J., 1972. Recherches géologiques sur le Crétacé inférieur de l’Estremadura (Portugal). Serviços Geologicos de Portugal 21, 1–477.

Richards, A.E., Gibbard, P.L., Pettit, M.E., 1999. The sedimentology and palaeoecology of the Westleton Member of the Norwich Crag Formation (Early Pleistodene) at Thorigton, Suffolk, England. Geological Magazine 136, 453–464.

Richardson, J.B., 1964. Middle Old Red Sandstone Spore Assemblages from the Orcadian Basin North-East Scotland. Palaeontology 7, 559–605.

Richardson, J.B., 1960. Spores from the Middle Old Red Sandstone of Cromarty, Scotland. Palaeontology 3, 45–63.

Rich, T.H., Giménez, O., Cúneo, R., Puerta, P.F., Vacca, R., Vickers-Rich, P.A., 1997. Primer registro de un camarasáurido primitivo en el Gondwana patagónico [First record of a primitive camarasaurid in Patagonian Gondwana]. Ameghiniana 34, 540.

Rich, T.H., Vickers-Rich, P., Gimenez, O., Cúneo, R., Puerta, P., Vacca, R., 1999. A new sauropod dinosaur from Chubut province, Argentina, Proceedings of the Second Gondwanan Dinosaur Symposium, National Science Museum Monographs.

Riek, E.F., 1973. Fossil insects from the Upper Permian of Natal, South Africa. Annals of the Natal Museum 21, 513–532.

Rigby, J.F., 1978. Jurassic plant fossils from the Walloon Coal Measures at Rosewood Consolidated Colliery. Queensland Government Mining Journal 526–529.

Rigby, J.F., 1970. The distribution of Lower Gondwana plants in the Parana Basin of Brazil. IUGS Commission on Stratigraphy, Sub-Commission on Gondwana Stratigraphy and Palaeontology; 2nd Gondwana Symposium, Proceedings and Papers 575–584.

Rigby, J.F., 1966. The Lower Gondwana floras of the Perth and Collie Basins, Western Australia. Palaeontographica Abteilung B 118, 113–152.

Ritenberb, M.I., Fadeeva, Z.I., 1961. Lower Mesozoic coal deposition in the Maykyuben’ basin area. Transactions of the Laboratory of Coal Geology. History of the Lower Mesozoic coal deposition in Kazakhstan. Part 1. 12, 253–298.

Rivett, W.H.E., 1956. On some reptilian bones from the Weald Clay of Surrey. Proceedings of the Geologists’ Association 67, 110–111.

Robison, C.R., Hunt, A., Woldberg, D.L., 1982. New Late Cretaceous leaf locality from lower Kirtland Shale member, Bisti area, San Juan Basin, New Mexico. New Mexico Geology.

Roghi, G., Kustatscher, E., Van Konijnenburg-van Cittert, J.H.A., 2006. Late Triassic Plants from the Julian Alps (Italy). Bollettino della Scietà Paleontologica Italiana 45, 133–140.

Rohn, R., Rosler, O., Pennatti, J.R., Czajkowski, S., Iannuzzi, R., Mendonca, E., Ferreira, A.R., Pereira, S.C.A., Quiterio, L., 1997a. Ocorrencias de macrofitofosseis nas Formacoes Teresina e Rio do Rasto (Permiano Superior da Bacia do Parana) na porcao meridional do Estado do Parana, Brasil. Revista Universidade Guarulhos - Geociencias II (no. especial) 58–68.

Rohn, R., Rosler, O., Pinnatti, J.R., Davies, H.P.K., Cavalheiro, M.C.T., 1997b. Ocorrencias de macrofitofosseis nas Formacoes Teresina e Rio do Rasto (Permiano Superior da Bacia do Parana) na regiao de Candido de Abreu-Reserva, Estado do Parana, Brasil. Revista Universidade Guarulhos - Geociencias II (no. especial) 69–75.

Romanova, E.V., 1961. Materials to the study of the Jurassic flora of the Borolday coal deposit. Materials on the history of fauna and flora of Kazakhstan 3, 104–120.

Romanova, E.V., 1958. About the Jurassic flora of Alakol’ depression. Materials on the history of fauna and flora of Kazakhstan 2, 143–148.

Romanov, L.F., Teslenko, Y.V., Yanovskaya, G.G., 1986. On paleontological characteristics of the Middle Jurassic deposits of the Lower Member of the Beshui suite, Crimea Mountain, Paleontogico-stratigraphic studies of Mesozoic and Cenozoic of Dnestr-Prust plain.

Romero, E.J., Fernandez, C.A., 1981. Palinología de paleosuelos del Cuaternario de los alrededores de Lobería (Provincia de Buenos Aires, República Argentina). Ameghiniana XVIII, 273–285.

Rose, J., Moorlock, B.S.P., Hamblin, R.J.O., 2001. Pre-Anglican fluvial and coastal deposits in Eastern England; lithostratigraphy and palaeoenvironments. Quaternary Internationat 79, 5–22.

Rosenkrantz, A., 1942. The Lower Jurassic Rocks of East Greenland Part II The Mesozoic Sediments of the Kap Hope Area Southern Liverpool Land. Meddelelser Om Gronland 110, 5–55.

Rosler, O., 1978. The Brazilian Eogondwanic floral succession. Boletim IG Instituto de Geociencias, Universidade de Sao Paulo 9, 85–91.

Rothwell, G.W., Stockey, R.A., 2006. Combining characters of Pteridaceae and tree ferns: Pterisorus radiata gen. et sp. nov., a permineralized Lower Cretaceous filicalean with radial sori. International Journal of Plant Sciences 167, 695–701. doi:10.1086/501035

Rowe, N., Galtier, J., 1989. A lower carboniferous plant assemblage from La Serre (Montagne Noire, France). Part I. Review of palaeobotany and palynology 61, 239–271.

Rowe, N.P., 1988a. A herbaceous lycophyte from the Lower Carboniferous drybrook sandstone of The Forest of Dean, Gloucestershire. Palaeontology 31, 69–83.

Rowe, N.P., 1988b. Two species of the lycophyte genus Eskdalia Kidston from the Drybrook sandstone (Visean) of Great Britain. Palaeontographica Abteilung B 208, 81–103.

Rozanov, V.I., 1968. On the age of the white quartz sandstone unit in Yarenga River basin. Sovetskaya Geologiya 121–122.

Rozefelds, A.C., Christophel, D.C., Alley, N.F., 1992. Tertiary occurrence of the fern Lygodium (Schizaeaceae) in Australia and New Zealand. Memoirs of The Queensland Museum 32, 203–222.

Ruiz, L.C., Quattrocchio, M.E., 1997. Estudio Palinológico de la Formación Pedro Luro (?Maastrichtiano-Paleoceno) en la Cuenca del Colorado, República Argentina. Parte 1: Esporas triletes, Laevigati, Murornati, Tricassati, Cingulati y Zonati. Revista Española de Micropaleontología 29, 13–29.

Rull, V., 1999. Palaeofloristic and palaeovegetational changes across the Paleocene/Eocene boundary in northern South America. Review of Palaeobotany and Palynology 107, 83–95.

Runnegar, B., 1969. A Lower Triassic Ammonoid Fauna from Southeast Queensland. Journal of Paleontology 43, 818–828.

Rushforth, S.R., 1971. A flora from the Dakota Sandstone Formation (Cenomanian) near Westwater, Grand County, Utah. Brigham Young University Science Bulletin 14, 1–44.

Saad, S.I., 1978. Plynological studie in the Egyptian Western Desert: Umbarka IX Borehole. Pollen et Spores 20, 261–301.

Sadovnikov, G.N., 1987. K stratigrafii Permo-Trisovikh otlozhenii Tungusskogo Basseina (Stratigraphy of Permian-Triassic deposits of Tungusska Basin). Sovetskaya Geologiya 2, 84–89.

Sakhanova, N.S., Il’ina, V.I., Yu. V. Teslenko, 1985. Kansk-Achinsk basin, Trudy Paleontologicheskogo Instituta. Jurassic continental biocoenosis of Southern Siberia and surrounding areas.

Saks, T.T., Zakharov, V., 1976. Stratigraphy of the Jurassic System of the Northern USSR, Nauka, Moscow.

Sal’menova, K.Z., 1982. On the floristic relationships between the floras of the southern and eastern parts of the Angara region in the Early Permian. Paleontologicheskii Zhurnal (Paleontological Journal) 16, 1–7.

Samylina, V.A., 1967. Mesozoic flora of the area to the west of the Kolyma River (Zyryanka coal basin). Part 2. Ginkgoales, Coniferales. General chapters, Botanical Institute. Academy of Sciences of the USSR. Trudy. Series 8. Paleobotanica.

Samylina, V.A., 1963a. Mesozoic flora of the Lower course of the Aldan River, Paleobotanica. Botanicheskii Institut. Akademiya Nauk SSSR.

Samylina, V.A., 1963b. Paleobotanical characteristics of continental Mesozoic deposits of Zyryanka-Silyap coal-bearing basin (left side of Kolyma River). Doklady Akademii Nauk SSSR 152, 1212–1214.

Samylina, V.A., Yefimova, A.F., 1968. First findings of Early Jurassic flora in the basin of Kolyma River. Doklady Akademii Nauk SSSR 179, 166–168.

Sander, P.M., 1987. Taphonomy of the Lower Permian Geraldine Bonebed in Archer County, Texas. Palaeogeography, Palaeoclimatology, Palaeoecology 61, 221–236.

Sanz, J.L., Ortega, F., Shibata, M., 2014a. , Dinosaurios Maravillosos de España [Marvelous Dinosaurs from Spain].

Sanz, J.L., Ortega, F., Shibata, M., 2014b. , Dinosaurios Maravillosos de España [Marvelous Dinosaurs from Spain].

Sanz, J.L., Wenz, S., Yebenes, A., Estes, R., Martinez-Delclos, X., Jiminez-Fuentes, E., Dieguez, C., Buscalioni, A.D., Barbadillo, L.J., Via, L., 1988. An Early Cretaceous faunal and floral continental assemblage: Las Hoyas fossil site (Cuenca, Spain). Géobios 21, 611–635.

Savitskaya, L.I., 1969. Late Jurassic flora of Fergana. Paleontologicheskii Zhurnal (Paleontological Journal) 135–138.

Scafati, L., Melendi, D.L., Volkheimer, W., 2009. A Danian subtropical lacustrine palynobiota from South America (Bororó Formation, San Jorge Basin, Patagonia - Argentina). Geological Acta 7, 35–61. doi:10.1344/105.000000270

Scasso, R.A., Del Valle, R.A., 1989. Nuevas observaciones sobre la Formacion Ameghino en la peninsula Sobral, Antartida. Contribuciones del Instituto Antartico Argentino 1–43.

Schaarschmidt, F., 1974. Mosellophyton hefteri n.g. n.sp. (?Psilophyta), ein sukkulenter Halophyt aus dem Unterdevon von Alken an der Mosel. Paläontologische Zeitschrift 48, 188–204.

Scheckler, S.E., 1986. Geology, Floristics and Paleoecology of Late Devonian Coal Swamps from Appalachian Laurentia (U.S.A.). Annales de la Societe geologique de Belgique 109, 209–222.

Schemel, M.P., 1950. Cretaceous Plant Microfossils from Iowa. American Journal of Botany 37, 750–754.

Schmidt, V.W., Teichmuller, M., 1954. Pflanzen-Reste aus dem Gedinne des Hogen Venns. Geology 69, 89–102.

Schneck, W.J., Fritz, W.J., 1985. An Amphibian Trackway (Cincosaurus cobbi) from the Lower Pennsylvanian (“Pottsville”) of Lookout Mountain, Georgia: A First Occurrence. Journal of Paleontology 59, 1243–1250.

Schneider, H., Schmidt, A.R., Heinrichs, J., 2016. Burmese amber fossils bridge the gap in the Cretaceous record of polypod ferns. Perspectives in Plant Ecology, Evolution and Systematics 18, 70–78.

Schneider, H., Schmidt, A.R., Nascimbene, P.C., Heinrichs, J., 2015. A new Dominican amber fossil of the derived fern genus Pleopeltis. Organisms Diversity & Evolution 15, 277–283.

Schopf, J.M., 1964. Middle Devonian plant fossils from Northern Maine. United States Geological Survey Professional Paper 501-D, D43–D49.

Schrank, E., 2010. Pollen and spores from the Tendaguru Beds, Upper Jurassic and Lower Cretaceous of southeast Tanzania: palynostratigraphical and paleoecological implications. Palynology 34, 3–42.

Schrank, E., Mahmoud, M.S., 2002. Barremian angiosperm pollen and associated palynomorphs from the Dakhla Oasis Area, Egypt. Palaeontology 45, 33–56.

Schweitzer, H., Heumann, G., 1993. Lower Devonian Flora of Roragen, in Southeast Norway. Palaeontographica 229, 1–52.

Scott, A.C., 1990. Preservation, evolution and extinction of plants in Lower Carboniferous volcanic sequences in Scotland, In: M.G. Lockley and A. Rice, eds. Volcanism and Fossil Biotas. Geological Society of America Special Paper.

Scott, A.C., Edwards, D., Rolfe, W.D.I., 1976. Fossiliferous Lower Old Red Sandstone near Cardross, Dumbartonshire. Proceedings of the Geological Society of Glasgow 117, 4–5.

Scott, A.C., Galtier, J., 1988. A new Lower Carboniferous flora from East Lothian, Scotland. Proceedings of the Geological Association 99, 141–151.

Scott, A.C., Galtier, J., Clayton, G., 1985. A new late Tournaisian (Lower Carboniferous) flora from the Kilpatrick Hills, Scotland. Review of palaeobotany and palynology 44, 81–99.

Scott, A.C., Galtier, J., Clayton, G., 1984. Distribution of anatomically-preserved floras in the Lower Carboniferous in Western Europe. Transactions of the Royal Society of Edinburgh 75, 311–340.

Scott, D.H., 1926. New Discoveries in the Middle Devonian Flora of Germany. New Phytologist 25, 373–379.

Scudder, S.H., 1880. The Devonian insects of New Brunswick. Anniversary Memoirs of the Boston Society of Natural History 1830-1880 1–41.

Scudder, S.H., 1875. Two new fossil cockroaches from the Carboniferous of Cape Breton. The Canadian Naturalist and Quarterly Journal of Science 7, 271–272.

Selim, M.M., Said, M.M., 1993. Contribution to the geology of Gabal Abraq, southeast of Aswan, Egypt. Annals of the Geological Survey of Egypt 19, 325–333.

Senkevitch, M.A., 1978. New Devonian psilophytes from Kazakhstan. Ezneg. Vses. Paleontol. Obschestva 21, 288–298.

Serbet, R., Rothwell, G.W., 2006. Anatomically preserved ferns from the Late Cretaceous of western North America. II. Blechnaceae and Dryopteridaceae. International Journal of Plant Sciences 167, 703–709.

Serbet, R., Rothwell, G.W., 2003. Anatomically preserved ferns from the Late Cretaceous of western North America: Dennstaedtiaceae. International Journal of Plant Sciences 164, 1041–1051.

Serbet, R., Stockey, R.A., 1991. Taxodiaceous pollen cones from the Upper Cretaceous (Horshoe Canyon Formation) of Drumheller, Alberta, Canada. Review of Palaeobotany and Palynology 70, 67–76.

Shaanxi Stratigraphic Group, 1983. The Stratigraphic Tables of Shaanxi, Geology Press.

Shah, S.C., 1972. Jurassic-Lower Cretaceous megaflora in India - a review. Records of the Geological Survey of India 109, 55–81.

Shandong Stratigraphic Group, 1978. The Stratigraphic Tables of Shandong, Geology Press.

Shanxi Stratigraphic Group, 1979. The Stratigraphic Tables of Shanxi, Geology Press.

Shevyrev, A.A., 1990. Ammonoidei i Khronostratigrafiya Triasa. Akademiya Nauk SSSR, Trudy Paleontologicheskogo Instituta 241, 1–179.

Shigeta, Y., Komatsu, T., Maekawa, T., Dang, H.T., 2014. Olenekian (Early Triassic) stratigraphy and fossil assemblages in northeastern Vietnam. National Museum of Nature and Science Monographs 45, 1–309.

Shulgina, N.I., Burdykina, M.D., Basov, V.A., Arhus, N., 1994. Distribution of ammonites, foraminifera and dinoflagellate cysts in the Lower Cretaceous reference sections of the Khatanga Basin, and Boreal Valanginian biogeography. Cretaceous Research 15, 1–16.

Sichuan Stratigraphic Group, 1976. The Stratigraphic Tables of Sichuan, Geology Press.

Silva-Pineda, A., 1987. Algunos elementos paleofloristicos del Permico de la region de Calnali, estado de Hidalgo. Revista de la Sociedad Mexicana de Paleontologia 1, 328–346.

Silva-Pineda, A., 1979. La Flora Triasica de Mexico. Univ. Nal. Auton. Mexico, Inst. Geologia, Revista 3, 138–145.

Silva-Pineda, A., 1978a. Paleobotanica del Jurasico de Mexico. Universidad Nacional Autonoma de Mexico. Instituto de Geologia, Paleontologia Mexicana 44, 1–16.

Silva-Pineda, A., 1978b. Plantas del Jurasico Medio del sur de Puebla y noroeste de Oaxaca. Universidad Nacional Autonoma de Mexico, Instituto de Geologia, Paleontologia Mexicana 44, 27–56.

Silverman, A.J., Harris, W.L., 1967. Stratigraphy and economic geology of the Great Falls-Lewistown coal field central Montana. State of Montana Bureau of Mines and Geology Bulletin 56 1–20.

Singh, C., 1983. Cenomanian microfloras of the Peace River area, northwestern Alberta. Bulletin of the Alberta Research Council 44, 1–322.

Singh, C., 1971. Lower Cretaceous Microfloras of the Peace River area, northwestern Alberta. Bulletin of the Alberta Research Council 28, 1–299.

Singh, C., 1964. Microflora of the Lower Cretaceous Mannville Group. Bulletin of the Alberta Research Council 15, 1–239.

Singh, K.J., Goswami, S., Singh, G., 2011. Palaeodiversity in the genus Glossopteris from the Lower Gondwana rocks of the Korba Coalfield, Chhattisgarh State, India. Journal of the Palaeontological Society of India 56, 45–64.

Singh, K.J., Singh, R., Cleal, C.J., Saxena, A., Chandra, S., 2013. Carboniferous floras in siliciclastic rocks of Kashmir Himalaya, India and the evolutionary history of the Tethyan Basin. Geological Magazine 150, 577–601.

Sinitsa, S.M., 1985a. Eastern TransBaykal, Trudy Paleontologicheskogo Instituta. Jurassic continental biocoenosis of Southern Siberia and surrounding areas.

Sinitsa, S.M., 1985b. Locality Dzhargalant. Locality Oshir-Boro-Udzyur-Ula, Trudy Paleontologicheskogo Instituta. Jurassic continental biocoenosis of Southern Siberia and surrounding areas.

Skoblo, V.M., 1978. New data about biostratigraphy and potential coal resourses of Jurassic deposits of the Western TransBaykal. Geologiia i Geofizika 144–149.

Skog, J.E., 1988. Reassignment of Aspidium heterophyllum to a new genus in the family Matoniaceae. American Journal of Botany 75, 1120–1129.

Skog, J.E., 1982. Pelletixia amelguita, a New Species of Fossil Fern in the Potomac Group (Lower Cretaceous). American Fern Journal 72, 115–121.

Skog, J.E., 1976. Loxsomopteris anasilla, a New Fossil Fern Rhizome from the Cretaceous of Maryland. American Fern Journal 66, 8–14.

Skog, J.E., Dilcher, D.L., 1994. Lower vascular plants of the Dakota Formation in Kansas and Nebraska, USA. Review of Palaeobotany and Palynology 80, 1–18.

Skog, J.E., Dilcher, D.L., 1992. A new species of Marsilea from the Dakota Formation in central Kansas. American Journal of Botany 79, 982–988.

Smith, J.P., 1932. Lower Triassic ammonoids of North America. United States Geological Survey Professional Paper 167.

Smith, M.A., Rothwell, G.W., Stockey, R.A., 2015. Mesozoic Diversity of Osmundaceae: Osmundacaulis whittlesii sp. nov. in the Early Cretaceous of Western Canada. International Journal of Plant Sciences 176, 245–258.

Smith, S.Y., Rothwell, G.W., Stockey, R.A., 2003. Cyathea cranhamii sp. nov. (Cyatheaceae), anatomically preserved tree fern sori from the Lower Cretaceous of Vancouver Island, British Columbia. American Journal of Botany 90, 755–760.

Smyshlyaeva, O.P., Zakharov, Y.D., 2013. New members of the family Flemingitidae (Ammonoidea) from the Lower Triassic of south Primorye. Paleontological Journal 47, 247–255.

Sniderman, J.M.K., 2011. Early Pleistocene vegetation change in upland south-eastern Australia. Journal of Biogeography 38, 1456–1470. doi:10.1111/j.1365-2699.2011.02518.x

Spalletti, L.A., Arrondo, O.G., Morel, E.M., Ganuza, D.G., 1988. Estudio sedimentologico y paleofloristico de la Formacion Paso Flores (Triasico Superior) en el sector occidental del Macizo Norpatagonico, Argentina. V Congreso Geologico Chileno 2, 395–413.

Speden, I.G., 1971. Geology of Papatowai subdivision, south-east Otago. New Zealand Geological Survey Bulletin 1–166.

Spicer, R.A., Ahlberg, A., Herman, A.B., Kelley, S.P., Raikevich, M.I., Rees, P.M., 2002. Palaeoenvironment and ecology of the middle Cretaceous Grebenka flora of northeastern Asia. Palaeogeography, Palaeoclimatology, Palaeoecology 184, 65–105.

Spicer, R.A., Herman, A.B., 2001. The Albian-Cenomanian flora of the Kukpowruk River, western North Slope, Alaska: stratigraphy, palaeofloristics, and plant communities. Cretaceous Research 22, 1–40.

Spicer, R.A., Hill, C.R., 1979. Principal Components and Correspondence Analyses of Quantitative Data from a Jurassic Plant Bed. Review of Palaeobotany and Palynology 28, 273–299.

Spurgeon, P.A., Jennings, J.R., 1985. Pennsylvanian plants of eastern Kentucky: A flora from the Breathitt Formation near Grannies Branch and Rocky Branch of Goose Creek, Clay County, Kentucky. Kentucky Geological Survey Rept. Inv. 3, Series 11.

Srebrodol’skaya, I.N., Kozubova, L.A., 1976. On age of the Ichetuy suite in Western TransBaykal. Geologiia i Geofizika 90–93.

Srivastava, R., 2011. Indian Upper Cretaceous-Tertiary flora before collision of Indian Plate: A reappraisal of central and western Indian flora. Memoir of the Geological Society of India 77, 281–292.

Srivastava, S.K., 1966. Upper Cretaceous microflora (Maestrichtian) from Scollard, Alberta, Canada. Pollen et Spores 8, 497–552.

Stamberg, S., 1997. New discoveries of palaeoniscoid fishes and other fauna and flora from the northern region of Boskovice Furrow, Czech Republic. Journal of the Czech Geological Society 42, 111–120.

Stanich, N.A., Rothwell, G.W., Stockey, R.A., 2009. Phylogenetic diversification of Equisetum (Equisetales) as inferred from Lower Cretaceous species of British Columbia, Canada. American Journal of Botany 96, 1289–1299.

Stanislavskii, F.A., 1987. Rhaetian flora of north-west part of the Donetskii Basin, Retskaia flora severozapadnoi okrainy Donbassa.

Stanislavskii, F.A., 1971. Flora and stratigraphy of Upper Triassic deposits of Donbass (Rhaetian flora of Raiskoie village).

Stanislavskii, F.A., 1957. Fossil flora of the Bathonian-Callovian deposits of the Donetzk basin and Dnepr-Donetzk depression.

Steadman, D.W., Franz, R., Morgan, G.S., Albury, N.A., Kakuk, B., Broad, K., Franz, S.E., Tinker_, K., Pateman_, M.P., Lott, T.A., Jarzen, D.M., Dilcher, D.L., 2007. Exceptionally well preserved late Quaternary plant and vertebrate fossils from a blue hole on Abaco, The Bahamas. Proceedings of the National Academy of Sciences 104, 19897–19902.

Steen, M.C., 1934. The amphibian fauna from the South Joggins, Nova Scotia. Proceedings of the Zoological Society of London 1934, 465–504.

Stein, W.E., 2002. SUNY-Binghamton Paleobiology Collection.

Stein, W.E., 1982. The Devonian Plant Reimannia, with A Discussion of the Class Progymnospermopsida. Palaeontology 25, 605–622.

Stein, W.E., Hueber, F.M., 1989. The anatomy of Pseudosporochnus: P. hueberi from the Devonian of New York. Review of Palaeobotany and Palynology 60, 311–359.

Stevanovic-Walls, I.M., 2001. A paleontological study of an Early Pennsylvanian forest preserved above the Blue Creek Coal seam, Pottsville, Formation, Northwestern Alabama.

Stipanicic, P.N., Bonetti, M.I.R., 1970a. Posiciones estratigraficas y edades de las principales floras jurasicas argentinas. I. Floras liasicas. Ameghiniana 7, 57–78.

Stipanicic, P.N., Bonetti, M.I.R., 1970b. Posiciones estratigraficas y edades de las principales floras jurasicas argentinas. II. Floras doggerianas y malmicas. Ameghiniana 7, 101–118.

Stockey, R.A., Rothwell, G.W., Little, S.A., 2006. Relationships among fossil and living Dipteridaceae: Anatomically preserved Hausmannia from the Lower Cretaceous of Vancouver Island. International Journal of Plant Sciences 167, 649–663. doi:10.1086/503268

Stockey, R.A., Smith, S.Y., 2000. A new species of Millerocaulis (Osmundaceae) from the Lower Cretaceous of California. International Journal of Plant Sciences 161, 159–166.

Stockmans, F., 1968. Vegetaux Mesodevoniens Recoltes Aux Confins Du Massif Du Brabant (Belgique). Institut Royal Des Sciences Naturelles De Belgique, Memoire 159, 3–49.

Stockmans, F., 1948. Vegetaux du Devonien Superieur de la Belgique. Memoires du Musee Royal D’Histoire Naturelle de Belgique 110, 3–85.

Stockmans, F., 1940. Vegetaux Eodevoniens de la Belgique. Musee Royal D’Histoire Naturelle De Belgique Rue Vautier 31.

Stone, J.F., Langston, W., Jr., 1975. Late Maestrichtian?–Paleocene palynomorphs associated with the sauropod dinosaur, ?Alamosaurus sanjuanensis. Geological Society of America, 9th Annual Meeting South-Central Section, Abstracts with Programs 7, 238–239.

Stopes, M.C., 1914. The “Fern Ledges” Carboniferous Flora of St. John, New Brunswick, Geologic Survey.

Stopes, M.C., 1907. The flora of the Inferior Oolite of Brora (Sutherland). Quarterly Journal of the Geological Society of London 63, 375–382.

Stuchlik, L., 1981. Tertiary pollen spectra from the Ezcurra Inlet Group of Admiralty Bay, King George Island (South Shetland Islands, Antartica). Studia Geologica Polonica 109–131.

Stuermer, W., Schaarschmidt, F., 1980. Pflanzen im Hunsrueckschiefer. Versteinertes Leben im Roentgenlicht 11, 19–25.

Sultan, I., 1978. Mid-Cretaceous plat microfossils from the northern part of the Western Desert of Egypt. Review of Palaeobotany and Palynology 25, 259–267.

Sultan, I., Aly, S.M., 1986. Palynological zonation of Albian-Cenomanian sediments in the northern part of the Western Desert of Egypt. Bulletin of the Faculty of Science of Alexandria University 26, 80–101.

Svanidze, T.I., 1970a. New data on the Middle Jurassic flora of Georgia. Bulletin of the Academy of Sciences of the Georgian SSR 60, 625–627.

Svanidze, T.I., 1970b. New data on the Upper Jurassic flora of Georgia. Bulletin of the Academy of Sciences of the Georgian SSR 59, 373–375.

Svanidze, T.I., 1960. On the age of the fossil flora of shales and coal suite of Okriba. Bulletin of the Academy of Sciences of the Georgian SSR 25, 561–564.

Svanidze, T.I., Shengeliya, F.K., 1987. Study of flora of the analcime horizon around Kutaisi. Bulletin of the Academy of Sciences of the Georgian SSR 125, 73–76.

Svanidze, T.I., Vashakidze, I.G., Yakobidze, E.B., 1983. New data on the Bathonian flora of Georgia. Bulletin of the Academy of Sciences of the Georgian SSR 111, 85–88.

Svanidze, T.S., Shengeliya, F.K., 1979. New data on flora and vegetation of the Middle Jurassic deposits of the Magana River. Bulletin of the Academy of Sciences of the Georgian SSR 93, 625–628.

Swartz, F.M., 1965. Guide to the Horse Shoe curve Section between Altoona and Gallitzin, Central Pennsylvania. General Geology Report G50 1–56.

Sweet, A.R., 1979. Jurassic and Cretaceous Megaspores. AASP Contributions Series Number 5B: Contributions of Stratigrphic Palynology (with emphasis on North America): Mesozoic Palynology 2, 1–30.

Sweet, A.R., Hills, L.V., 1974. A detailed study of the genus Azollopsis. Canadian Journal of Botany 52, 1625–1642.

Szafer, W., 1961. Miocenska Flora ze Starych Gliwic na Slasku (Miocene Flora of Stare Gliwice in Upper Silesia). Prace Instytut Geologiczny 33, 1–205.

Takahasi, E., 1958. Mesozoic floral succession of Nagato Mountainland, western Japan. The Palaeobotanist 7, 155–159.

Tanner, W.R., 1984. A Fossil Flora from the Beartooth Butte Formation of Northern Wyoming.

Tashi, S.M., Burago, V.I., 1974. Litologo-paleofloristicheskaya kharakteristika permskikh otlozhenii Yuzhnogo Primor’ya [Lithologo-paleofloral characteristics of the Permian deposits of South Primor’e]. Sovetskaya Geologiya 9, 40–48.

Taylor, D.W., 1986. Anatomical and Morphological Study of a New Species of Taeniocrada, a Devonian Tracheophyte from New York State. Review of Palaeobotany and Palynology 47, 63–87.

Taylor, G., Truswell, E.M., McQueen, K.G., Brown, M.C., 1990. Early Tertiary palaeogeography, landform evolution, and palaeoclimates of the Southern Monaro, N.S.W., Australia. Palaeogeography, Palaeoclimatology, Palaeoecology 78, 109–134. doi:10.1016/0031-0182(90)90207-N

Teichert, C., Schop, J.M., 1958. A Middle or Lower Devonian Psilophyte Flora from Central Arizona and its Paleogeographic Significance. Journal of Geology 66, 208–217.

Teslenko, Y.V., 1987. Materials to investigation of the Middle Jurassic floras of northeastern Bulgaria. Geologica Balcanica 17, 71–75.

Teslenko, Y.V., 1982. New species of Jurassic plants of the Crimea, Systematics and the evolution of ancient plants of the Ukraine; a collection of scientific works.

Teslenko, Y.V., 1975. On stratigraphy of the Jurassic deposits of Eastern TransBaykal. Geologiia i Geofizika 41–45.

Teslenko, Y.V., 1974. Plant remains from upper horizons of Shadoron series in the Eastern TransBaykal and their stratigraphic significance, Trudy. Institut Geologii i Geofiziki. Academy of Science of the USSR. Siberian division. Biostratigraphy of the Boreal Mesozoic.

Teslenko, Y.V., 1971. Data on the Toarcian flora of eastern Transbaikalia. Sib. Nauchno-Issled. Inst. Geol. Geofiz. Miner. Syr’ya, Trudy. In: Materialy po regional’noy geologii Sibiri 39–42.

Teslenko, Y.V., 1970. Stratigraphy and flora of the Jurassic deposits of the Western and Southern Siberia and Tuva, Trudy SNIIGGIMSa. Series “Paleontology and Stratigraphy”.

Teslenko, Y.V., 1968. About the Late Jurassic and Early Cretaceous floras of the Eastern TransBaykal. Doklady Akademii Nauk SSSR 183, 910–913.

Teslenko, Y.V., 1964. Materials on stratigraphy and correlation of the Jurassic coal-bearing deposits of Kuznetsk, Ulugkhem, and Irkutsk basins. Trudy SNIIGGIMSa. Materials on stratigraphy of Sayan-Altai folded area. 29, 125–141.

Teslenko, Y.V., Mogucheva, N.K., 1967. Discovery of Early Jurassic deposits in Tuva, Stratigraphy of the Mesozoic and Cenozoic of Middle Siberia.

Teslenko, Y.V., Mogucheva, N.K., 1965. New data for study of Jurassic flora of the Ulugkhem coal basin in Tuva. Geologiia i Geofizika 80–84.

Teslenko, Y.V., Mogucheva, N.K., 1964. Paleobotanic materials on stratigraphy of Jurassic coal deposits of Tuva. Doklady Akademii Nauk SSSR 155, 822–825.

Thomas, B.A., Cleal, C.J., 2001. A new early Westphalian D flora from Aberdulais Falls, South Wales. Proceedings of the Geologists’ Association 112, 373–377.

Thuy, B., Klompmaker, A.A., Jagt, J.W.M., 2012. Late Triassic (Rhaetian) ophiuroides from Winterswijk, the Netherlands; with comments on the systematic position of Aplocoma (Echinodermata, Ophiolepididae). Zoosymposia 7, 163–172.

Tidwell, W.D., 1990. Preliminary report on the megafossil flora of the Upper Jurassic Morrison Formation. Hunteria 2, 1–11.

Tidwell, W.D., 1988. A new Upper Pennsylvanian or Lower Permian flora from southeastern Utah. Brigham Young University Geological Studies 35, 33–56.

Tidwell, W.D., 1967. Flora of Manning Canyon Shale, Part I: A lowermost Pennsylvanian flora from the Manning Canyon Shale, Utah, and its stratigraphic significance. Brigham Young University Geological Studies 14, 3–66.

Tidwell, W.D., Britt, B.B., Ash, S.R., 1998. Preliminary floral analysis of the Mygatt-Moore Quarry in the Jurassic Morrison Formation, west-central Colorado. Modern Geology 22, 341–378.

Tidwell, W.D., Hebbert, N., 1992. Species of the Cretaceous tree fern Tempskya from Utah. International Journal of Plant Sciences 153, 513–528.

Tidwell, W.D., Jennings, J.R., Beus, S.S., 1992. A Carboniferous flora from the Surprise Canyon Formation in the Grand Canyon, Arizona. Journal of Paleontology 66, 1013–1021.

Tidwell, W.D., Kern, J.H., Kimura, T., 1987. Mid-Mesozoic leaves from near Ida Bay, southern Tasmania, Australia. Papers and Proceedings of the Royal Society of Tasmania 121, 159–170.

Tidwell, W.D., Nishida, H., Webster, N., 1989. Oguracaulis banksii gen. et sp. nov., a mid-Mesozoic tree-fern from Tasmania, Australia, in: Papers and Proceedings of the Royal Society of Tasmania. pp. 15–25.

Tidwell, W.D., Rozefelds, A.C., 1991. Yulebacaulis normanii gen. et sp. nov., a new fossil tree fern from south-eastern Queensland, Australia. Australian Systematic Botany 4, 421–432.

Tidwell, W.D., Skog, J.E., 1992. Two new fossil matoniaceous stem genera from Tasmania, Australia. Review of palaeobotany and palynology 70, 263–277.

Tidwell, W.D., Thayn, G.F., 1985. Flora of the Lower Cretaceous Cedar Mountain Formation of Utah and Colorado, Part IV. Palaeopiceoxylon thinosus (Protopinaceae). The Southwestern Naturalist 30, 525–532.

Tidwell, W.., Thayn, G.F., Roth, J.L., 1976. Cretaceous and Early Tertiary Floras of the Intermountain Area. Brigham Youg University Geology Studies 22, 77–98.

Tillyard, R.J., Dunstan, B., 1916. Mesozoic and Tertiary Insects of Queensland and New South Wales. Descriptions of the fossil Insects and stratigraphical features. Queensland Geological Survey 1–63.

Tims, J.., Chambers, T.C., 1984. Rhyniophytina and Trimerophytina from the Early Land Flora of Victoria, Australia. Palaeontology 27, 265–279.

Todesco, R., Wachtler, M., Kustatscher, E., Avanzini, M., 2008. Preliminary report on a new vertebrate track and flora site from Piz da peres (Anisian-Illyrian): Olang Dolomites, Northern Italy. Geo.Alp 5, 121–137.

Tokaryk, T.T., Bryant, H.N., 2004. The fauna from the Tyrannosaurus rex excavation, Frenchman Formation (Late Maastrichtian), Saskatchewan. Summary of Investigations 2004, Volume 1. Saskatchewan Geological Survey, Saskatchewan Industry Resources, Miscellaneous Report 2004-4 1, 1–12.

Tolstykh, A.N., 1974. Pozdnepaleozoiskaia flora Zapadnogo Verkhoian’ia (Upper Paleozoic flora of West Verkhonian’ia, Siberia)., Akademia Nauk SSSR, Siberian region. Novosibirsk, Nauka.

Tosolini, A.-M.P., McLoughlin, S., Drinnan, A.N., 2002. Early Cretaceous megaspore assemblages from southeastern Australia. Cretaceous Research 23, 807–844.

Townrow, J.A., 1964. Speculation of the Rhaeto-Liassic climate of Tasmania. Papers and Proceedings of the Royal Society of Tasmania 98, 113–118.

Tozer, E.T., Calon, T.J., 1990. Triassic ammonoids from Jabal Safra and Wadi Alwa, Oman, and their significance. In: Robertson, A. H. F., Searle, M. P. & Ries, A. C. (eds) The Geology and Tectonics of the oman Region Geological Society, London, Special Publication 203–211.

Trettin, H.P., 1969. Geology of Ordovician to Pennsylvanian rocks, M’Clintock Inlet, north coast of Ellesmere Island, Canadian Arctic Archipelago. Geological Survey of Canada Bulletin 183, 1–93.

Troncoso, A., Suárez, M., de la Cruz, R., Palma-Heldt, S., 2002. Paleoflora de la Formación Ligorio Márquez (XI Región, Chile) en su localidad tipo: sistemática, edad e implicancias paleoclimáticas. Revista Geológica de Chile 29, 113–135. doi:10.4067/S0716-02082002000100007

Trunko, L., Munk, W., 1990. Makrofloren aus dem Unterrotliegenden (Unterperm) von Alsenz bei Bad Kreuznach/Rheinland Pfalz. Carolinea 48, 21–30.

Truswell, E.M., Macphail, M.K., 2009. Polar forests on the edge of extinction: what does the fossil spore and pollen evidence from East Antarctica say?. Australian Systematic Botany 57–106. doi:10.1071/SB08046

Tschudy, R.H., 1966. Associated megaspores and microspores of the Cretaceous genus Ariadnaesporites Potonie, 1956, emend. United States Geological Survey Professional Paper 550-D, D76–D82.

Tuchkov, I.I., 1972. Stratigraphy of the USSR. Jurassic System.

Tweet, J., Chin, K., Murphy, N., 2005. An analysis of possible gut contents in a hadrosaurid dinosaur from the Upper Cretaceous (late Campanian) Judith River Formation of Montana. Journal of Vertebrate Paleontology 25, 125A.

Upchurch, G.R., Crane, P.R., Drinnan, A.N., 1994. The Megaflora from the Quantico Locality (Upper Albian), Lower Cretaceous Potomac Group of Virginia. Virginia Museum of Natural History 4, 1–57.

Ustritskii, V.I., et al., 1981. Permian plants, Novaya Zemlya’.

Vaillant-Couturier Treat, I., 1933. Paléontologie de Madagascar. XIX - Le Permo-Trias Marin. Annales de Paléontologie 22, 39–96.

Vakhrameev, V.A., 1983. Subdivision of Upper Triassic deposits of PriKumsk oil region. Sovetskaya Geologiya 54–63.

Vakhrameev, V.A., 1958. Stratigraphy and fossil flora of the Jurassic and Cretaceous deposits of the Vilui River basin and surrounding part of the PriVerkhoyansk marginal trough, Regional Stratigraphy of the USSR.

Vakhrameev, V.A., Dobruskina, I.A., Zhatikova, Z.A., Yaroshenko, O.P., 1977. Verkhetriasovie floromosnie otlozheniia Vostochnogo Predkavkaz’ia [Upper Triassic flora-bearing deposits of the eastern pre-Caucasus]. Izvestiya Akademiia Nauk SSSR. Seriia Geologicheskaia 3, 62–72.

Vakhrameev, V.A., Doludenko, M.P., 1961. Late Jurassic and Early Cretaceous flora of the Bureya basin and its significance for stratigraphy. Trudy Geologicheskogo Instituta 54.

Vakhrameev, V.A., Grigyalis, A.A., Mikhailov, I.A., 1971. Finding of the Middle Jurassic flora in the Papile suite of the Southern Prebaltics. Soviet Geology 3, 120–123.

Vakhrameev, V.A., Lebedev, E.L., 1985. Jurassic floras and climate of Pacific USSR, Special paper IGCP Project 171: Circum Pacific Jurassic. Jurassic climate of western Pacific area.

Vakhrameev, V.A., Petros’yants, M.A., 1973. Distribution of plant fossils, Ob’yasnitel’naya zapiska k stratigraphicheskoi skheme yurskikh otlozhenii Severnogo Kavkaza. (Explanatory notes for stratigraphic scheme of Jurassic deposits of the North Caucasus).

Vallati, P., 2013. Paleotropical pollen grains from the Neuquén Group, Patagonia, Argentina. Carnets de Géologie 273–279.

Vallati, P., 2010. Asociaciones palinológicas con Angiospermas en el Cretácico Superior de la Cuenca Neuquina, Argentina. Revista Brasileira de Paleontolgia 13, 143–158. doi:10.4072/rbp.2010.2.07

Vallati, P., 2006. Las primeras angiospermas en el Cretácico de la Cuenca Neuquina (Centro Oeste de Argentina): Aspectos geológicos relacionados. Revista Brasileira de Paleontologia 9, 83–92.

Vallati, P., 2001. Middle Cretaceous Microflora from the Huincul Formation (“Dinosaurian Beds”) in theNeuquén Basin, Patagonia, Argentina. Palynology 25, 179–197.

van Ameron, H.W.J., Boersma, M., Niedermayr, G., Scheriau-Niedermayr, E., 1976. Das permische Alter der “Karbon”-Flora von Kotschach (Karnten, Osterreich). Carinthia II 166/86, 93–101.

Van Beneden, P.-J., 1878. Sur la découverte de reptiles fossiles gigantesques dans le charbonnage de Bernissart près de Péruwelz [On the discovery of gigantic fossil reptiles in the Bernissart coal mine near Péruwelz]. Bulletin de l’Academie Royale des Sciences, des Lettres et des Beaux-Arts de Belgique, série 2 45, 578–579.

Van Boskirk, M.C., 1998. The flora of the Eagle Formation and its significance for Late Cretaceous floristic evolution.

Van der Burgh, J., Van Konijnenburg-Van Cittert, J.H.A., 1984. A drifted flora from the Kimmeridgian (Upper Jurassic) of Lothbeg Point, Sutherland, Scotland. Review of Palaeobotany and Palynology 43, 359–396.

Van Itterbeeck, J., Missiaen, P., Folie, A., Markevich, V.S., Van Damme, D., -Y. Guo, D., Smith, T., 2007. Woodland in a fluvio-lacustrine environment on the dry Mongolian Plateau during the late Paleocene: Evidence from the mammal bearing Subeng section (Inner Mongolia, P.R. China). Palaeogeography, Palaeoclimatology, Palaeoecology 243, 55–78.

van Konijnenburg-van Cittert, J.H.A., Bandel, K., 2001. Jurassic plants from Djebel Tih, Sinai. Mitt. Geol.-Paläont. Inst. Univ. Hamburg 85, 47–64.

Van Konijnenburg-van Cittert, J.H.A., Kustatscher, E., Wachtler, M., 2006. Middle Triassic (Anisian) ferns from Kühwiesenkopf (Monte Prá Della Vacca), Dolomites, Northern Italy. Palaeontology 49, 943–968.

van Konijnenburg-van Cittert, J.H.A., Morgans, H.S., 1999. The Jurassic Flora of Yorkshire. Palaeontological Association Field Guides to Fossils 8, 1–134.

Van Konijnenburg-van Cittert, J.H.A., Van der Burgh, J., 1996. Review of the Kimmeridgian flora of Sutherland, Scotland with reference to the ecology and in situ pollen. Geologists’ Association 107, 97–105.

Van Konijnenburg-Van Cittert, J., Van der Burgh, J., 1989. The flora from the Kimmeridgian (Upper Jurassic) of Culgower, Sutherland, Scotland. Review of Palaeobotany and Palynology 61, 1–51.

Van Neer, W., 1984. The use of fish remains in African archaeozoology. Centre de Recherches Archeol. Notes et Monogra. Tech. 16, 155–167.

Varlamov, V.A., Kiselev, A.I., et al., 1972. Geology of North-eastern Asia.

Varyukhina, L.M., Koloda, N.A., Molin, V.A., Fefilova, L.A., Chadyshev, V.F., 1975. Kompleksy rasteniy; sporovo-pyl’tsevyye kompleksy [Plant assemblages; spore-pollen assemblages.], Biogeograficheskoe raionirovanie Evropeiskogo Severa SSSR (permii trias). Akademia Nauk SSSR, Leningrad.

Vasina, R.A., Doludenko, M.P., 1968. Late Aalenian flora of Dagestan. Paleontologicheskii Zhurnal (Paleontological Journal) 90–98.

Vassilevskaja, I.D., 1984. First discovery of Triassic plants on Novaya Zemlya Island [in Russian]. Vsesoivznoe Paleontologicheskoe Obshchestvo (Ezhegodnik) 27, 109–119.

Vavrek, M.J., Stockey, R.A., Rothwell, G.W., 2006. Osmunda vancouverensis sp. nov. (Osmundaceae), perminerallized fertile frond segments from the lower Cretaceous of British Columbia, Canada. International Journal of Plant Sciences 167, 631–637.

Vega, F.J., García-Barrera, P., del Carmen Perrilliat, M., Coutiño, M.A., Mariño-Pérez, R., 2006. El Espinal, a new plattenkalk facies locality from the Lower Cretaceous Sierra Madre Formation, Chiapas, southeastern Mexico. Revista Mexicana de Ciencias Geológicas 23, 323–333.

Vera, E.I., 2015. Further evidence supporting high diversity of cyathealean tree ferns in the Early Cretaceous of Antarctica. Cretaceous Research 56, 141–154.

Vera, E.I., 2010. Estudios anatómicos en paleofloras del Aptiano de Antártida y Patagonia, y su comparación.

Vera, E.I., 2010. Oligocene ferns from the Rancahué Formation (Aluminé, Neuquén, Argentina): Cuyenopteris patagoniensis nov. gen., nov. sp.(Polypodiales: Blechnaceae/Dryopteridaceae) and Alsophilocaulis calveloi Menéndez emend. Vera (Cyatheales: Cyatheaceae). Geobios 43, 465–478.

Via Boada, L., Villalta, J.F., Esteban Cerda, M., 1977. Paleontologia y paleoecolgia de los yacimientos fosiliferos del Muschelkalk superior entre Alcover y Mont-Ral (Montañas de Prades, Provincia de Tarragona). Cuadernos Geología Ibérica 4, 247–256.

Villar de Seoane, L., 2005. Equisetites pusillus sp. nov. from the Aptian of Patagonia, Argentina. Revista del Museo Argentino de Ciencias Naturales, n. s 7, 43–49.

Villar de Seoane, L., Archangelsky, S., 2008. Taxonomy and biostratigraphy of Cretaceous megaspores from Patagonia, Argentina. Cretaceous Research 29, 354–372. doi:10.1016/j.cretres.2007.07.002

Vladimirov, A.Y., Mogucheva, N.K., Nikulov, L.P., Romanov, A.P., 1990. O raschlenenii permotriasovykh vulkanogennykh otlozheniy Zapadnogo Taymyra [Subdivision of Upper Permian and Lower Triassic volcanic deposits in western Taymyr]. Institut Geologii i Geofiziki, Trudy 767, 4–13.

Vladimirovich, V.P., 1985. Typical Kungurian flora of the Middle Urals. Viniti 377.

Vladimirovich, V.P., 1967. Biostratigraphy of Triassic and Jurassic continental deposits of eastern slope of the Urals, northern Kazakhstan and mountain part of western Siberia, Stratigrafiya i paleontologiya mezozoiskikh i paleogen-neogenovikh kontinental. Nikh otlozhenii Aziatskoi chasti.

Vladimirovich, V.P., 1959. New data on subdivision and correlation of coal deposits in different localities of the Turgay (Ubagan) basin. Informatsionnyi sbornik Vsesouznogo Nauchno-Issledovatel’skogo Geologicheskogo Instituta 15–23.

Vladimirovich, V.P., Lyufanov, L.E., 1969. On the question of stratigraphy of the Jurassic deposits of Podobass-Tutuyass trough of Kuznetsk basin. Trudy Vsesoyuznogo nauchno-issledovatel’skogo geologicheskogo instituta (VSEGEI) Novaya seriya. Collection of papers on biostratigraphy. Vypusk (vol.) 4 130, 22–29.

Vladimirovich, V.P., Teslenko, Y.V., 1969. About the Early Jurassic flora of Tuva. Trudy SNIIGGIMSa. Materialy po stratigrafii i paleontologii Sibiri. 84, 94–97.

Volkheimer, W., 1971. Algunos adelantos de la microbioestratigrafía del Jurásico en la Argentina y comparación con otras regiones del Hemisferio Austral. Ameghiniana VIII, 341–355.

Volkheimer, W., Novara, M.G., Narvaéz, P.L., Marquillas, R.A., 2006. Palynological and paleoenvironmental significance of the Tunal Formation (Danian) at its type locality, El Chorro creek (Salta, Argentina). Ameghiniana 43, 567–584.

Volkheimer, W., Scafati, L., PARSE ERROR, 2007. Palynology of a Danian warm climatic wetland in Central Northern Patagonia, Argentina. Revista Española de Micropaleontología 38, 117–134.

Voropinov, V.S., Ermolaev, D.I., 1966. Flora and fauna of Ust’-Balei Jurassic. Geologiia i Geofizika 30–37.

Waagen, W., 1895. Fossils from the Ceratite Formation. Salt Range Fossils, Palaeontologia Indica, Series 13 2, 1–323.

Wagner, R.H., 1985. Upper Stephanian stratigraphy and palaeontology of the Puertollano Basin, Ciudad Real, Spain. An. Fac. Cien., Porto, Suppl. (Papers on the Carboniferous of the Iberian Peninsula) 64, 171–231.

Wagner, R.H., 1983. A lower Rotliegend flora from Ayrshire. Scottish Journal of Geology 19, 135–155.

Wagner, R.H., 1971. The Westphalian D floras of the Olloniego and Esperanza formations in the central Asturian coalfield. Separata de Trabajos de Geologica 461–505.

Wagner, R.H., 1962a. An Upper Westphalian flora from teh mine Inés, near the pass of Pajares, on the Leonese-Asturian boundary (N.W. Spain). Extracto de Notas y Comunicaciones del Instituto Geológico y Minero de España 79–102.

Wagner, R.H., 1962b. On a mixed Cathaysia and Gondwana flora from SE. Anatolia (Turkey). Compte Rendu du Ive Congrés pour l’Avancement des Éstudes de Stratigraphie et de Géologie du Carbonifére 745–752.

Wagner, R.H., Lemos de Sousa, M.J., 1982. A middle Westphalian flora from the Alvarelhos strip near the Serra de Bougado, Santo Tirso, North Portugal. Communicações dos Serviços Geológicos de Portugal 68, 257–266.

Wagner, R.H., Martinez Garcia, E., 1982. Description of an Early Permian flora from Asturias and comments on similar occurrences in the Iberian Penisula. Trabajos de Geologia, Universidad de Oviedo 12, 273–287.

Wagner, R.H., Soper, N.J., Higgins, A.K., 1982. A Late Permian flora of Pechora affinity in North Greenland. Gronlands geol. Unders. 108, 5–13.

Wagner, R.H., Talens, J., Melendez, B., 1985. Upper Stephanian stratigraphy an megaflora of Henarejos (province of Cuenca) in the Cordillera Iberica, central Spain. An. Fac. Cienc., Porto, Suppl. (Papers on the Carboniferous of the Iberian Peninsula) 64, 445–480.

Wagner, R.H., Winkler Prins, C.., 1970. The stratigraphic succession, flora, and fauna of Cantabrian and Stephanian A rocks at Barruelo (prov. Palencia), N.W. Spain. Colloque sur la Stratigraphie du Carbonifère 55, 487–551.

Wagner, R.H., Winkler Prins, C.F., 1983. The Catabrian and Barruelian stratotypes: A summary of basin development and biostratigraphic information. An. Fac. Ciences, Porto, Suppl. 64, 359–410.

Waksmundzka, M., 1982. LOWER CRETACEOUS MEGASPORES FROM NORTHERN POLAND. Acta Palaeontologica Polonica 27, 147–156.

Walkom, A.B., 1932. Fossil Plants from Mount Piddington and Clarence Siding. Proceedings of the Linnean Society of New South Wales 57, 123–126.

Wang, D.X., He, B., Zhang, S.L., 1984. On a mixed Cathaysia and Angara flora from Qilianshan region. Scientific Papers on Geology for International Exchange 1, 13–24.

Wang, L., Xie, Z., Wang Ziqiang, 1978. On the occurrence of Pleuromeia from the Qinshui Basin in Shanxi Province. Acta Palaeontologica Sinica 17, 195–212.

Wang, X., Kellner, A.W.A., Jiang, S., Meng, X., 2009. An unusual long-tailed pterosaur with elongated neck from western Liaoning of China. Anais da Academia Brasileira de Ciências 81, 793–812.

Wang, Y.G., Zheng, Z.G., Chen, G.L., 1979. Cephalopoda, Paleontological Atlas of Northwest China, Qinghai Volume.

Ward, L.F., 1899. The Cretaceous formation of the Black Hills as as indicated by the fossil plants, Cretaceous Formation of the Black Hills.

Ware, W.N., 2001. A paleoecological study of an in situ peat-forming Carboniferous forest in the Black Warrior Basin, Alabama.

Wartiti, M. El, Broutin, J., Freytet, P., Larhrib, M., Toutin-Morin, N., 1990. Continental deposits in Permian basins of the Mesetian Morocco, geodynamic history. Journal of African Earth Sciences. Major African continental Phanerozoic complexes and dynamics of sedimentation. 10, 361–368.

Weber, R., 2013. Salvinia coahuilensis nov. sp. del Cretácico Superior de México. Ameghiniana 10, 173–190.

Weber, R., 1973. Salvinia coahuilensis nov. sp. del Cretácico Superior de México. Ameghiniana 10, 173–190.

Weber, R., Trjo-Cruz, R., Tores-Romo, A., Garcia-Padilla, A., 1980. Hipotesis de trabajo acerca de la paleoecologia de comunidades de la tafoflora Santa Clara del Triasico Tardio de Sonora. Univ. Nal. Auton. Mexico, Inst. Geologia, Revista 4, 138–154.

Weems, R.E., 1980. An unusual newly discovered archosaur from the Upper Triassic of Virginia, U.S.A. Transactions of the American Philosophical Society, New Series 70, 1–53.

Welles, S.P., 1947. Vertebrates from the Upper Moenkopi Formation of northern Arizona. University of California Publications in Geological Sciences 27, 241–294.

Wellman, C.H., Habgood, K., Jenkins, G., Richardson, J.B., 2000. A new plant assemblage (microfossil and megafossil) from the Lower Old Red Sandstone of the Anglo-Welsh Basin: its implications for the palaeoecology of early terrestrial ecosystems. Review of Palaeobotany and Palynology 109, 161–196.

Wesley, A., 1966. The fossil flora of the Grey Limestones of Veneto, Northern Italy, and its relationships to the other European floras of similar age. The Palaeobotanist 14, 124–130.

Westgate, J.W., 2001. Paleoecology and biostratigraphy of marginal marine gulf coast Eocene vertebrate localities, Eocene Biodiversity: Unusual Occurrences and Rarely Sampled Habitats.

Westgate, J.W., 1988. Biostratigraphic implications of the first Eocene land-mammal fauna from the North American coastal plain. Geology 16, 995–998.

White, D., 1929. Flora of the Hermit Shale, Grand Canyon, Arizona. Carnegie Institution of Washington Publications.

White, D., 1912. The characters of the fossil plant Gigantopteris Schenk and its occurrence in North America. Proceedings of the United States National Museum 41, 493–516.

White, D., 1893. Flora of the Outlying Carboniferous Basins of Southwestern Missouri. United States Geological Survey 1–137.

Whitehead, D.R., 1983. Pollen analysis of the Peat Member from the Lee Creek Mine. Smitsonian Contributions to Paleobiology 53, 265–285.

White, M.E., 1981. Revision of the Talbragar Fish Bed Flora (Jurassic) of New South Wales. Records of the Australian Museum 33, 695–721.

Whitmore, F.C., 1987. Cetacea from the Sahabi Formation, Libya, Neogene Paleontology and Geology of Sahabi.

Wignall, P.B., 1987. A biofacies analysis of the Gastroceras cumbriense marine band (Namurian) of the central Pennines. Proceedings of the Yorkshire Geological Society 46, 111–121.

Wilde, V., Frankenhauser, H., 1998. The Middle Eocene plant taphocoenosis from Eckfeld (Eifel, Germany). Review of Palaeobotany and Palynology 101, 7–28.

Wilf, P., 2000. Late Paleocene-early Eocene climate changes in southwestern Wyoming: Paleobotanical analysis. Geological Society America Bulletin 112, 292–307.

Wilf, P., Johnson, K.R., Cúneo, N.R., Smith, M.E., Singer, B.S., Gandolfo, M.A., 2005. Eocene Plant Diversity at Laguna del Hunco and Río Pichileufú, Patagonia, Argentina. The American Naturalist 165, 634–650. doi:10.1086/430055

Willard, D.A., 1992. Early Virgilian palynofloras from the Kinney Quarry, Manzanita Mountains, New Mexico. New Mexico Bureau of Mines and Mineral Resources Bulletin 138, 49–60.

Willard, D.A., DiMichele, W.A., Eggert, D.L., Hower, J.C., Rexroad, C.B., C Scott, A., 1995. Paleoecology of the Springfield Coal Member (Desmoinesian, Illinois Basin) near the Leslie Cemetery paleochannel, southwestern Indiana. International Journal of Coal Geology 27, 59–98.

Wilson, L.R., 1962. Permian plant microfossils from the Flowerpot Formation, Greer County, Oklahoma. Oklahoma Geological Survey 49.

Wing, S.L., 1990. personal research. personal research.

Wing, S.L., Alroy, J., Hickey, L.J., 1995. Plant and mammal diversity in the Paleocene to Early Eocene of the Bighorn Basin. Palaeogeography, Palaeoclimatology, Palaeoecology 115, 117–155.

Wing, S.L., Herrera, F., Jaramillo, C.A., Gómez-Navarro, C., Wilf, P., and C. C. Labandeira, 2009. Late Paleocene fossils from the Cerrejón Formation, Columbia, are the earliest record of Neotropical rainforest. Proceedings of the National Academy of Sciences. doi:10.1073/pnas.0905130106

Winston, R.B., 1983. A Late Pennsylvanian upland flora in Kansas: Systematics and environmental implications. Rev. Palaeobotany. Palynol. 40, 5–31.

Witham, H.T.M., 1833. the Internal Structure of Fossil Vegetables Found in the Carboniferous and Oolitic Deposits of Great Britain.

Wittry, J., Glasspool, I.J., Béthoux, O., Koll, R., Cleal, C.J., 2015. A revision of the Pennsylvanian marattialean fern Lobatopteris vestita auct. and related species. Journal of Systematic Palaeontology 13, 615–643.

Wolfart, R., Wittekindt, H., 1980. Geologie Von Afghanistan.

Woodhams, K.E., Hines, J.S., 1989. Dinosaur footprints from the Lower Cretaceous of East Sussex, England, Dinosaur Tracks and Traces. Cambridge University Press, Cambridge.

Wood, J.M., 1963. The Stanley Cemetery Flora (Early Pennsylvanian) of Greene County, Indiana. Indiana Department of Conservation Geological Survey Bulletin 29.

Woodward, A.S., 1895. The fossil fishes of the Talbragar Beds (Jurassic?). Memoirs of the Geological Survey of New South Wales 9, 1–31.

Woodward, H., 1885. On Iguanodon mantelli, Meyer. Geological Magazine, decade 3 2, 10–15.

Wu, X.W., 1982a. Fossil plants from the Upper Triassic Tumaingela Formation in Amdo-Baqen area, northern Xizang, Paleontology of Tibet.

Wu, X.W., 1982b. Late Triassic plants from eastern Xizang, Paleontology of Tibet.

Xinjiang Stratigraphic Group, 1981. The Stratigraphic Tables of Xinjiang, Geology Press.

Xu, K., Li, Y., Li, R., Wang, R., 1998. Discovery of the Dinosaur fossil in Heishan, Liaoning and its stratigraphical significance. Journal of stratigraphy (=Dicengxue-zazhi) 22, 227–231.

Yakobidze, E.B., 1980. New data on flora of the Bathonian deposits of Western Georgia. Bulletin of the Academy of Sciences of the Georgian SSR 99, 393–396.

Yang, G.X., Chen, Z.H., Zhang, S.L., 1983. The floras characteristic of Taiyuan Formation and Shansi Formation of the eastern Shungeerqi, Inner Mongol. Journal of the Wuhan College of Geology 22, 69–83.

Yang, Z.Y., Yin, H.F., Wu, S.B., Yang, F.Q., Ding, M.H., Xu, G.R., 1987. , Permian-Triassic Boundary Stratigraphy and Fauna of South China.

Yolkin, E.A., Gratsianova, R.T., Bakharev, N.K., Izokh, N.E., Yu. Yazikov, A., V’yushkova, L.V., Zheltonogova, V.A., Petrosyan, N.M., 1988. Facies and faunal associations of the Telengitian (Emsian) in its type locality, in N. J. McMillan, A. F. Embry, and D. J. Glass, eds. Devonian of the World, Volume III. Canadian Society of Petroleum Geologists. Calgary, Alberta, Canada.

Yunnan Stratigraphic Group, 1978. The Stratigraphic Tables of Yunnan, Geology Press.

Yurina, A.L., 1969. Devonskaia flora Tsentral’nogo Kazakhstana. Materialy po geologii Tsentral’nogo Kazakhstana 8, 1–207.

Zakharevich, A.N., 1980. Flora from the Stratotype of the Kozyrev and Korkino Suite of the Chelyabinsk Basin, News on Stratigraphy of the Triassic of the PaleoUrals.

Zakharov, Y.D., Ehiro, M., 2006. Systematic description: Early Triassic (Olenekian) ammonoids from Khentey Province, Mongolia, and their paleobiogeographic significance. Bulletin of the Tohoku University Museum 5, 83–97.

Zakharov, Y.D., Popov, A.M., Buryi, G.I., 2004a. Triassic Ammonoid succession in South Primorye: 2. Middle Olenekian Tirolites - Amphistephanites Zone. Albertiana 29, 29–37.

Zakharov, Y.D., Popov, A.M., Buryi, G.I., 2004b. Triassic Ammonoid succession in South Primorye: 3. Late Olenekian - Early Anisian Zones (Neocolumbites insignis, Subcolumbites multiformis, Ussuriphyllites amurensis and Leiophyllites pradyumna). Albertiana 31, 54–64.

Zakharov, Y.D., Shigeta, Y., 2000. Gyronautilus, a new genus of Triassic Nautilida from South Primorye, Russia. Paleontological Research 4, 231–234.

Zakharov, Y.D., Shigeta, Y., Popov, A.M., Buryi, G.I., Oleinikov, A.V., Dorukhovskaya and T. M. Mikhalik, E.A., 2002. Triassic Ammonoid succession in South Primorye: 1. Lower Olenekian Hedenstroemia bosphorensis and Anasibirites nevolini Zones. Albertiana 27, 42–64.

Zamaloa, M.C., 2004. Miocene algae and spores from Tierra del Fuego, Argentina. Alcheringa 28, 205–227. doi:10.1080/03115510408619282

Zamaloa, M.C., 2000. Palinoflora y ambiente en el Terciario del nordeste de Tierra del Fuego, Argentina. Revista del Museo Argentino de Ciencias Naturales 43–51.

Zamaloa, M.C., Romero, E.J., 1990. Some Spores and Pollen from the Cullen Formation (Upper Eocene to Middle Oligocene), Tierra Del Fuego, Argentina 14, 123–133.

Zambrano Garcia, A., Weber, R., 1985. Nuevo ensayo de un panorama de la paleoecologia de comunidades de la tafoflora Santa Clara (Triasico Tardio, Sonora). III Congreso Latinoamericano de Paleontologia. Mexico. Simposio sobre Floras del Triasico Tardio, Su Fitogeografia y Paleoecologia. Memoria. 153–163.

Zetter, R., Hofmann, C.C., Draxler, I., Durango de Cabrera, J., Vergel and F. Vervoorst, M.M., 1999. A rich Middle Eocene microflora at Arroyo de los Mineros, near Cañadón Beta, NE Tierra del Fuego Province, Argentina. Abhandlungen der Geologischen Bundesanstalt 436–460.

Zhejiang Stratigraphic Group, 1979. The Stratigraphic Tables of Zhejiang, Geology Press.

Zherikhin, V.V., 1985. Verkhnee PriAmur’e and Amur-Zeya depression, Trudy Paleontologicheskogo Instituta. Jurassic continental biocoenosis of Southern Siberia and surrounding areas.

Zhou, Z., 1983. Quaternary record of Azolla pinnata from China and its sporoderm ultrastructure. Review of Palaeobotany and Palynology 39, 109–129.

Zhou, Z., Crepet, W.L., Nixon, K.C., 2001. The earliest fossil evidence of the Hamamelidaceae: Late Cretaceous (Turonian) inflorescences and fruits of Altingioideae. American Journal of Botany 88, 753–766.

Zhou, Z.-Y., Li, B.-X., 1979. A preliminary study of the Early Triassic plants from the Qionghai District, Hainan Island. Acta Palaeontologica Sinica 18, 444–464.

Ziegler, A.M., Rees, P.M., Naugolnykh, S.V., 2002. The Early Permian floras of Prince Edward Island, Canada: differentiating global from local effects of climate change. Canadian Journal of Earth Sciences 39, 223–238.

Zimina, V.G., 1977. Flora Rannei i Nachala Pozdnei Permi Yuzhnogo Primor’ya [Flora of Early to early Late Permian of Southern Primor’ya], Dafnevost. nauch. tesntr. Moskva.

Ziquiang, W., 1983. Osmundacaulis hebeiensis, a new species of fossil rhizomes from the Middle Jurassic of China. Review of Palaeobotany and Palynology 39, 87–107.

Zobaa, M., Sanchez-Botero, C., Browne, C., Oboh-Ikuenobe, F.E., Ibrahim, M.I., 2008. Kerogen and palynomorph analyses of the mid-Cretaceous Bahariya Formation and Abu Roash “G” Member, North Western Desert, Egypt. Gulf Coast Association of Geological Societies Transactions 58, 933–943.
